# Enantioselective Synthesis of 3‑Alkyl-1,5-Functionalized 1,4-Diynes via Isoxazol-5(4*H*)‑ones

**DOI:** 10.1021/acs.orglett.5c04457

**Published:** 2026-01-14

**Authors:** Ricardo Torán, Sergi Vercher, Pablo López, Amparo Sanz-Marco, Marc Montesinos-Magraner, Carlos Vila, Gonzalo Blay

**Affiliations:** Departament de Química Orgànica, Facultat de Química, 16781 Universitat de València, C. Dr. Moliner 50, 46100 Burjassot, Spain

## Abstract

An enantioselective strategy for the synthesis of chiral 3-alkyl-1,5-functionalized 1,4-diynes is reported. The method involves a highly enantioselective organocatalytic conjugate addition of isoxazol-5­(4*H*)-ones to nitroenynes followed by a Zard-type transformation under one-pot conditions yielding chiral skipped diynes in good overall yields and with excellent enantioselectivities. This study highlights the synthetic versatility of the isoxazol-5­(4*H*)-one scaffold and its value as a platform for accessing structurally diverse and synthetically relevant building blocks.

## Graphical abstract

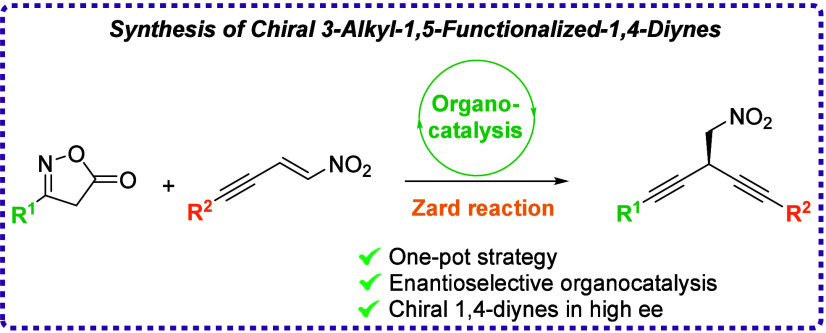

Diyne-containing molecules are of great importance as they are widely distributed in natural products, often display significant biological activities, and are important building blocks.^1–5^ This has inspired extensive research devoted to their synthesis and functionalization. Among the different structural motifs, particular attention has been given to 1,4-diynes (skipped diynes), which show a unique structure involving two alkyne groups linked to a doubly propargylic carbon. With focus on asymmetric strategies, the synthesis of skipped diynes bearing a propargylic stereogenic center remains elusive, probably due to the similar shape of the alkyne substituents and their linear structure, which hampers effective enantiodiscrimination. Consequently, a very reduced number of examples leading to skipped diynes possessing a heteroatom bonded to the stereogenic carbon have been reported so far (Scheme 1A).^6–9^

**Scheme 1:**
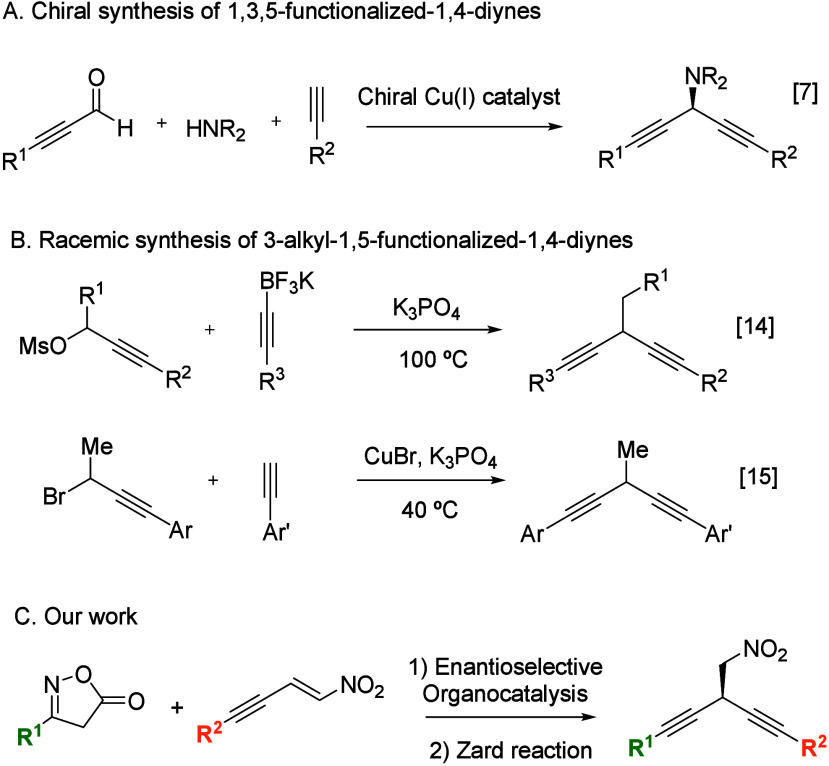
Representative Examples of the Synthesis of 1,3,5-Functionalized 1,4-Diynes

In contrast, despite the recognized importance of 3-alkyl-1,5-functionalized 1,4-diynes,^10–12^ their synthesis has been relatively limited compared to their analogues bearing a heteroatom at the C3 position. This is largely due to the intrinsic synthetic challenges they present since the absence of a heteroatom in the diyne core reduces the number of reliable disconnections and available strategies. Nevertheless, in 2020, a synthesis of symmetric 3-alkyl-1,5-functionalized 1,4-diynes was reported via a nickel-catalyzed, chemodivergent 1,1-difunctionalization of unactivated α-olefins using alkynyl electrophiles and B_2_pin_2_.^13^ For unsymmetrical 3-alkyl-1,5-functionalized 1,4-diynes, only two racemic strategies have been reported (Scheme 1B). In 2022, a valuable approach was disclosed based on the nucleophilic substitution of sp^3^-carbon electrophiles with alkynyltrifluoroborates, which allowed efficient access to a range of unsymmetrical diynes.^14^ More recently, an alternative copper-catalyzed transformation has been developed. Specifically, the CuBr-catalyzed coupling of propargyl bromides with terminal alkynes was demonstrated to deliver a diverse set of 3-methyl-1,5-functionalized 1,4-diynes with good yields and functional group tolerance.^15^ However, the synthesis of chiral 3-alkyl-1,5-functionalized 1,4-diynes remains a major challenge, and no methodology has been reported to date.

Isoxazol-5­(4*H*)-ones are five-membered heterocycles derived from isoxazole, characterized by a carbonyl group at the 5 position. These compounds exist in equilibrium among three tautomeric forms, CH, NH, and OH forms, depending on the nature of substituents, solvent, and other factors.^16^ Their unique reactivity arises from several features: (i) an acidic hydrogen at C4, (ii) a relatively weak N–O bond, (iii) the presence of three nucleophilic sites at N2, C4, and exocyclic oxygen, and (iv) facile release of CO_2_. These properties make isoxazol-5­(4*H*)-ones versatile intermediates in organic synthesis. Notably, they have been employed as nucleophiles in asymmetric organocatalysis^17–19^ and can be transformed into a broad range of functionalized scaffolds.^20–23^ Among the diverse transformations, the conversion of isoxazol-5­(4*H*)-one into alkyne represents a particularly interesting reaction, first explored by Zard and his research team in 2002.^24^ This transformation was later successfully carried out by Jurberg, who demonstrated that, following the organocatalyzed conjugate addition of ketones to alkylidene isoxazol-5­(4*H*)-ones, the isoxazole ring could be converted into an alkyne, thereby affording chiral α-propargyl ketones with good yields.^25^

Herein, we propose the first synthesis of chiral 3-alkyl-1,5-functionalized 1,4-diynes by exploiting the unique reactivity of the isoxazol-5­(4*H*)-one ring (Scheme 1C). Our strategy focuses on the development of a one-pot methodology involving an enantioselective conjugate addition, followed by a Zard-type reaction, using isoxazol-5­(4*H*)-ones as nucleophiles and nitroenynes as electrophiles. In fact, nitroenyne represents an attractive electrophile due to the simultaneous presence of an alkyne and a nitro group, which enables the synthesis of highly functionalized compounds.^26^ Despite the promising reactivity and high functionalization of these substrates, the enantioselective addition of isoxazol-5­(4*H*)-ones to nitroenynes has not yet been reported. This conjugate addition provides access to a versatile, chiral, and highly functionalized intermediate that can subsequently undergo a Zard reaction to furnish chiral 3-alkyl-1,5-functionalized 1,4-diynes.

To achieve this goal, we initially investigated the organocatalyzed 1,4-addition of isoxazol-5­(4*H*)-one **1a** to nitroenyne **2a** as the key step toward the formation of desired chiral intermediate **A** (Table 1). Initially, quinine-derived squaramide **I** and thiourea **II** were evaluated in dichloromethane at room temperature (entries 1 and 2). Interestingly, product formation was observed; however, an additional acetylation step was required to determine the enantioselectivity since the addition product **A** existed in several tautomeric forms.^27^ Following this protocol, compound **3aa** was obtained as a promising result with catalyst **II** (69% ee, entry 2). Subsequently, squaramide **III** and thiourea **IV**, derived from chiral cyclohexanediamine, were also investigated (entries 3 and 4), affording an increase in enantioselectivity up to 88% in the case of **IV**. Moreover, the use of commercially available catalyst **V** improved the yield of **3aa** while maintaining enantioselectivity, and thus, this catalyst was selected for further optimization (entry 5). Remarkably, lowering the reaction temperature to 0 °C provided **3aa** in 63% yield with 92% ee (entry 6). Other chlorinated solvents were then explored (entries 7 and 8), with chloroform affording the product in both a high yield and enantioselectivity (entry 8). In contrast, when a non-chlorinated solvent such as toluene was tested, a significant decrease in enantioselectivity was observed (entry 9). Therefore, the optimal conditions were established as those of entry 8, under which **3aa** was obtained in 70% yield with 92% ee. Notably, when the reaction was performed on a 1 mmol scale under these conditions, product **3aa** was obtained in over 80% yield with 92% enantiomeric excess, demonstrating the scalability and robustness of the protocol (entry 10).

**Table 1:**
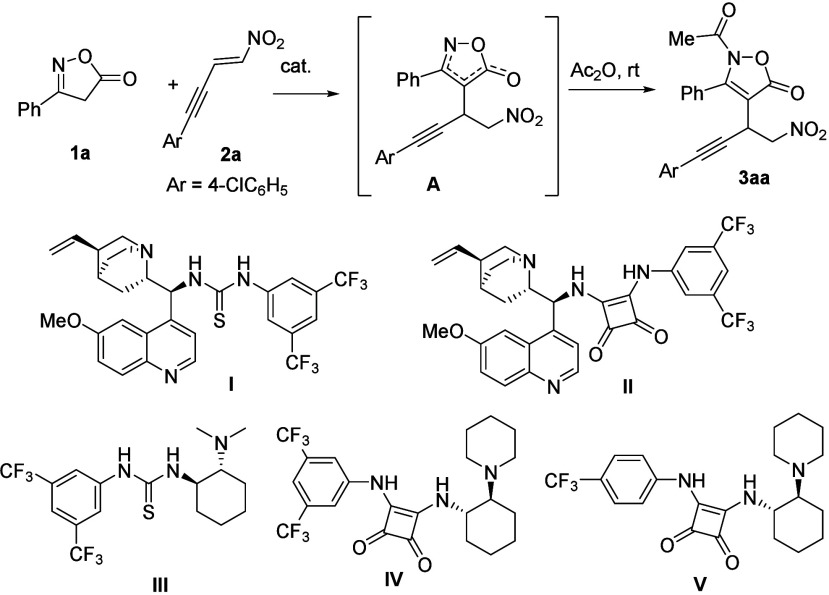
Enantioselective Conjugate Addition of 3-Phenylisoxazol-5­(4*H*)-one **1a** to Nitroenyne **2a**: Optimization Process^a^

entry	catalyst	solvent	*T* (°C)	**3aa** yield (%)^b^	**3aa** ee (%)^c^
1	**I**	CH_2_Cl_2_	rt	36	35
2	**II**	CH_2_Cl_2_	rt	64	69
3	**III**	CH_2_Cl_2_	rt	64	36
4	**IV**	CH_2_Cl_2_	rt	48	88
5	**V**	CH_2_Cl_2_	rt	73	87
6	**V**	CH_2_Cl_2_	0	63	92
7	**V**	(ClCH_2_)_2_	0	40	92
8	**V**	CHCl_3_	0	70	92
9	**V**	toluene	0	79	88
10^d^	**V**	CHCl_3_	0	80	92

a Reaction conditions: **1a** (0.1 mmol), **2a** (0.1 mmol), catalyst (0.01 mmol), and solvent (1 mL) for 24 h.

b Yield of the isolated product.

c Determined by chiral HPLC.

d On a 1 mmol scale reaction.

Next, the scope of this key step was examined to probe the robustness of the reaction (Scheme 2). Guided by our final objective, this study was carried out by coupling isoxazol-5­(4*H*)-ones **1** with nitroenynes **2** bearing different substituents, thereby enabling access to chiral skipped diynes in the subsequent step. First, several isoxazol-5­(4*H*)-ones **1** were reacted with nitroenyne **2a**. Aromatic substituents at the C4 position of **1** were well-tolerated, affording the corresponding products **3aa**–**3da** in good overall yields with excellent enantioselectivities. In addition, an aliphatic substituent was examined, providing compound **3ea**, which displayed high reactivity and moderate enantiomeric excess. Subsequently, other nitroenynes **2** were evaluated with different isoxazol-5­(4*H*)-ones **1**. *meta*-Chloro-substituted nitroenyne **2b** was first examined, affording products **3ab**, **3bb**, and **3fb** in good yields and high enantioselectivities, regardless of the electronic nature of 3-phenylisoxazol-5­(4*H*)-one derivative **1**. In addition, nitroenyne **2c** bearing a phenyl substituent delivered the corresponding products **3bc**, **3gc**, and **3fc** with excellent results. Moreover, substituents with different electronic features on the aromatic ring, such as a methoxy group in the *para* and *meta* positions, were explored, providing compounds **3ad**, **3fd** and **3ae** in high yields and with enantiomeric excesses above 90%. Aliphatic nitroenynes **2f** and **2g** were also investigated, affording products **3af**, **3bf**, **3ff**, and **3ag** in moderate yields but with high enantioselectivities.^28^ Finally, silyl-substituted nitroenyne **2h** furnished the expected product **3ah** in 71% yield and 90% ee.

**Scheme 2:**
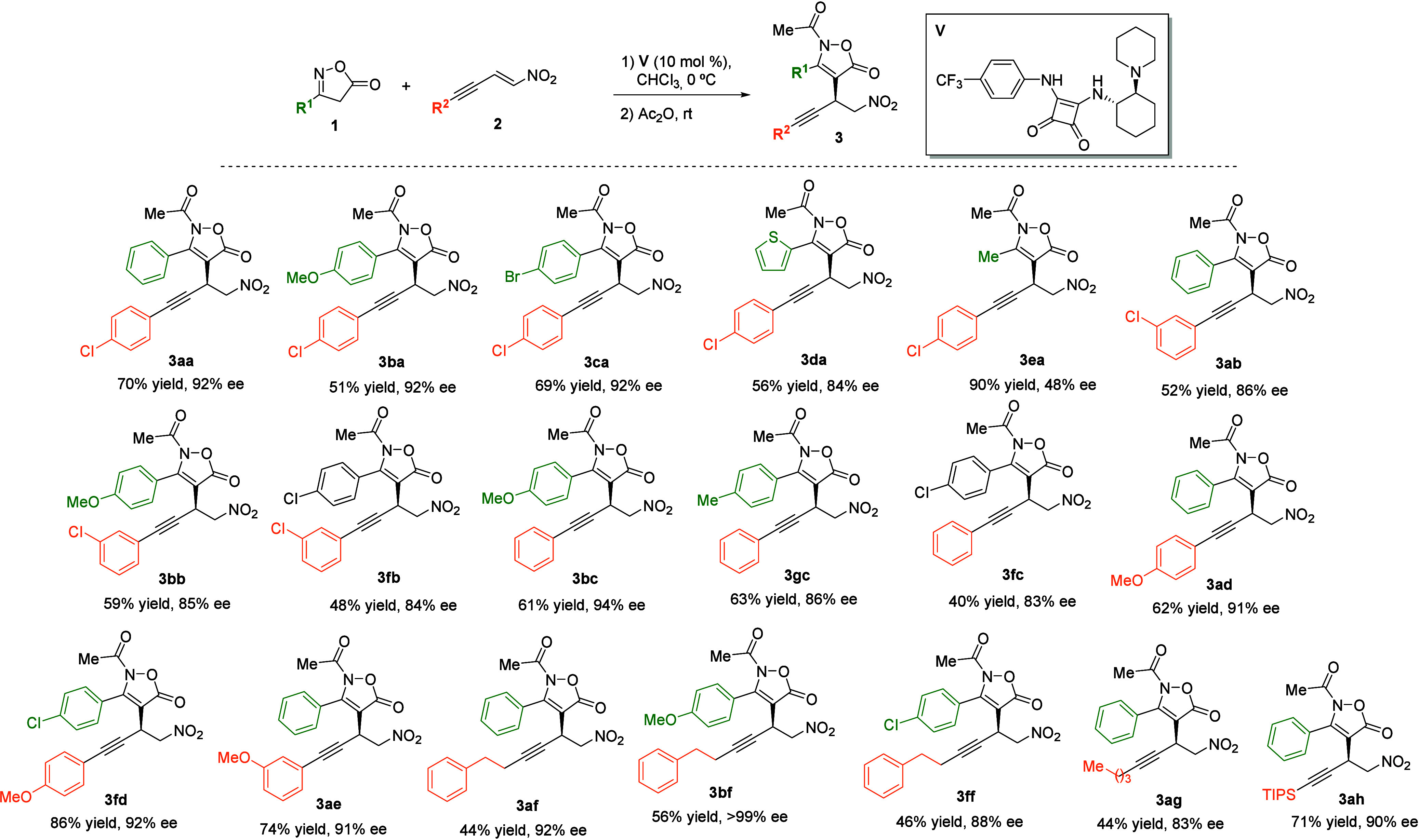
Conjugate Addition/Acetylation of Isoxazolinones **1** to Nitroenynes **2**: Reaction Scope^a^

a Reaction conditions: (1) **1** (0.1 mmol), **2** (0.1 mmol), **V** (0.010 mmol), and CHCl_3_ (1 mL) at 0 °C and (2) Ac_2_O (0.2 mmol) at rt for 30 min, with overall isolated yields. Enantiomeric excesses of compounds **3** were determined by chiral HPLC analysis after column chromatography.

Once we had demonstrated the broad scope of the enantioselective conjugate addition of isoxazolones **1** to enynes **2**, we undertook the synthesis of chiral skipped diynes by subjecting compound **3aa** to the Zard reaction conditions that involve treatment with FeSO_4_ and NaNO_2_ in aqueous acetic acid (see the Supporting Information). Under these conditions, compound **3aa** was converted into diyne **4aa** in an almost quantitative yield without erosion in the enantiomeric excess (Scheme 3). Then, we explored the feasibility of performing the conjugate addition and the Zard reaction in a one-pot fashion avoiding the acylataion step. Thus, after completion of the reaction of isoxazolinone **1a** with nitroenyne **2a**, chloroform was removed under reduced pressure, and the resulting crude mixture was treated under Zard conditions. In this way, the expected skipped diyne **4aa** was obtained in both the yield and enantioselectivity comparable to those of product **4aa** prepared via the acetylation approach (70% yield and 91% ee vs 92% ee, respectively). Following this strategy, a series of chiral 3-alkyl-1,5-functionalized 1,4-diynes **4** were prepared (Scheme 4).

**Scheme 3:**
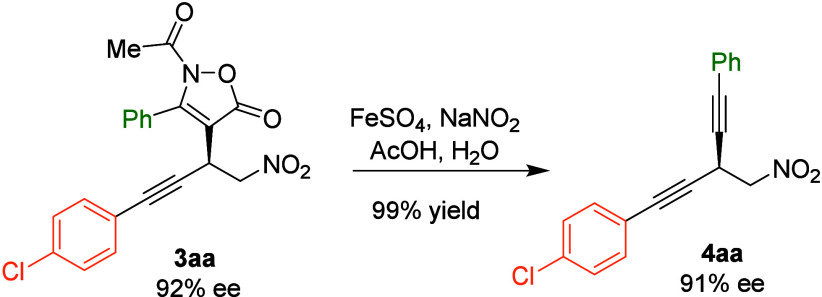
Zard Reaction of Compound **3aa** Leading to Diyne **4aa**

**Scheme 4:**
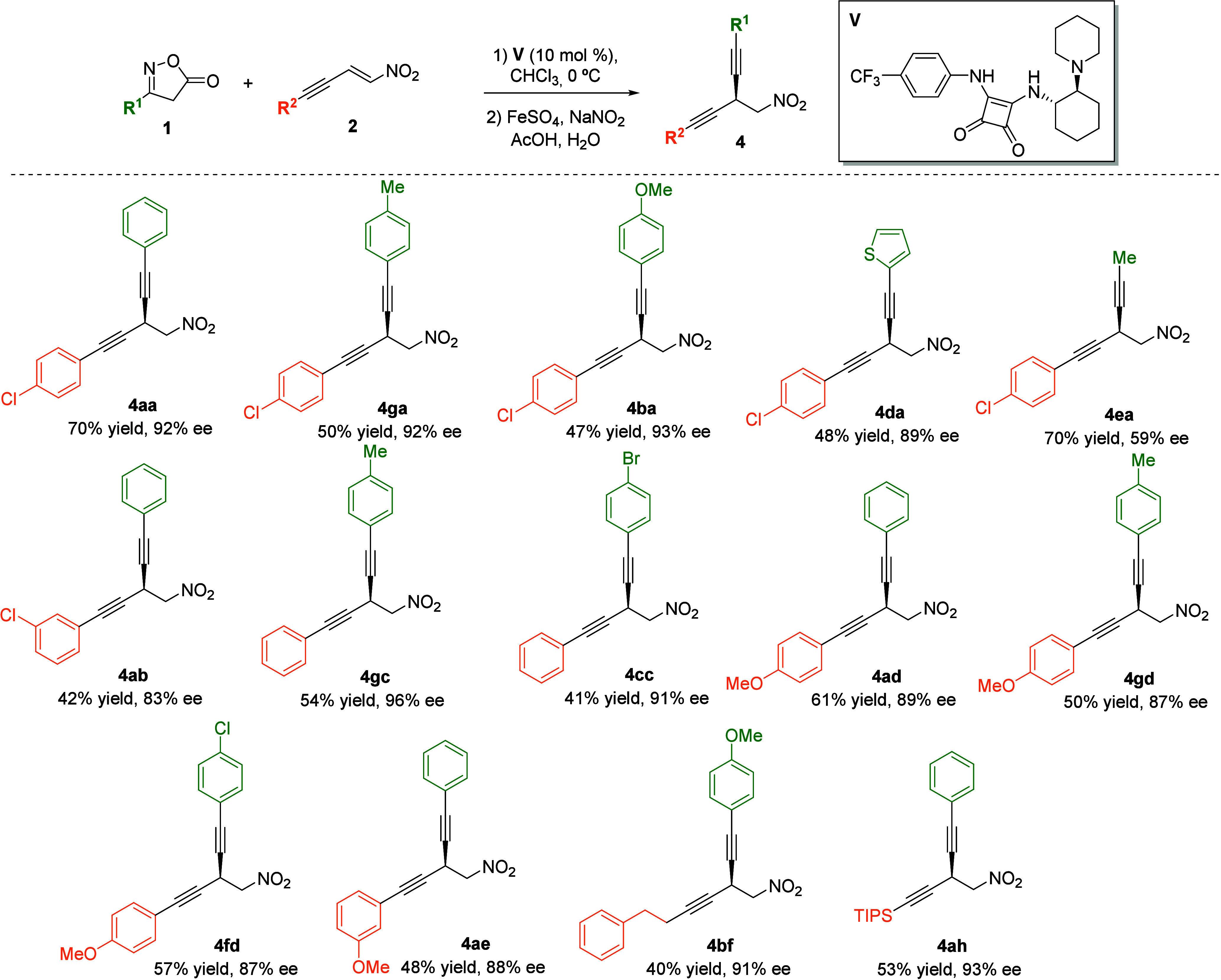
Synthesis of Chiral Skipped Diynes **4** via One-Pot Conjugate Addition/Zard Reaction of Isoxazolinones **1** and Nitroenynes **2**: Reaction Scope^a^

a Reaction conditions: (1) **1** (0.1 mmol), **2** (0.1 mmol), **V** (0.010 mmol), and CHCl_3_ (1 mL) at 0 °C and (2) NaNO_2_ (1 mmol), FeSO_4_ (0.55 mmol), AcOH (2 mL), and H_2_O (1 mL) at rt for 45 min, with overall isolated yields. Enantiomeric excesses for final compounds **4** were determined by chiral HPLC analysis.

First, various skipped diynes **4** were synthesized by reacting different isoxazol-5­(4*H*)-ones **1** with nitroenyne **2a**. Isoxazol-5­(4*H*)-ones bearing either aromatic or heteroaromatic substituents at the C3 position of **1** afforded the desired diynes **4aa**, **4ga**, **4ba**, and **4da** in good yields and with excellent enantioselectivities. Notably, 3-methylisoxazol-5­(4*H*)-one (**1e**), presenting an alkyl group at C3, was tolerated, thereby affording compound **4ea**, which incorporates both alkyl- and aryl-substituted alkynes within its structure. Then, we explored different alkynes. *m*-Chlorophenylnitroenye **2b** and isoxazole-5­(4*H*)-one **1a** provided the expected diyne **4ab** in a moderate yield and with somehow lower enantioselectivity compared to nitroenyne **2a** (92% ee vs 83% ee). Nitroenyne **2c**, bearing an unsubstituted phenyl ring, reacted with isoxazole-5­(4*H*)-one **1c** and **1g** to give compounds **4gc** and **4cc** with consistent 87% ee. Moreover, nitroenynes bearing an electron-releasing methoxy group at either the *meta* or *para* position in the phenyl group could also be employed with a range of isoxazol-5­(4*H*)-ones **1**, providing the corresponding skipped diynes **4ad**, **4gd**, **4fd**, and **4ae** in moderate yields but consistently high enantioselectivities. Finally, nitroenynes bearing aliphatic or silyl substituents (**2f** and **2h**) were likewise well-tolerated under the optimized conditions, affording corresponding products **4bf** and **4ah** with enantioselectivities above 90%.

To determine the absolute configuration of the obtained compounds, a one-pot sequence was performed with compounds **1a** and **2a**, consisting of an enantioselective conjugate addition followed by reductive N–O bond cleavage with concomitant decarboxylation of the isoxazol-5­(4*H*)-one ring to give ketone **5** (Scheme 5).^29^ Ketone **5** obtained in this way showed identical spectroscopic features and optical rotation sign as those reported in the literature for (*R*)-**5**.^30^ Accordingly, ketone **5** synthesized by our procedure was assigned to have the *R* configuration. Accordingly, we assigned the sterochemistry of compounds **3aa** and **4aa** by chemical correlation with those of (*R*)-**5**. The absolute stereochemistry of the remaining compounds **3** and **4** was assigned upon the assumption of a uniform mechanistic pathway involving the approach of isoxazolinone from the *Re* face of the nitroenyne double bond during the Michael addition step (see the Supporting Information for a stereochemical model). This transformation, together with the other reactions described herein, highlights both the synthetic versatility of the isoxazol-5­(4*H*)-one scaffold and its importance as a key platform for the preparation of valuable building blocks.

**Scheme 5:**
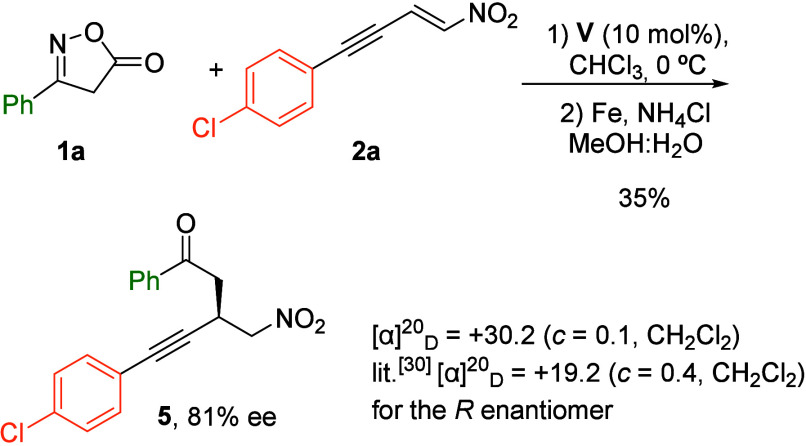
Synthesis of Compound **5**: Determination of Absolute Stereochemistry

In conclusion, we have developed the first synthesis of chiral 3-alkyl-1,5-functionalized 1,4-diynes through a one-pot efficient strategy combining an enantioselective conjugate addition with a subsequent Zard-type reaction. The enantioselective organocatalytic conjugate addition of isoxazol-5­(4*H*)-ones to nitroenynes served as the key step, providing compounds **3** in good yields and with high enantioselectivities. With the advantage of the versatility of the isoxazol-5­(4*H*)-one scaffold, the resulting chiral intermediates underwent a one-pot Zard-type transformation to deliver the desired chiral skipped diynes **4** for the first time, in good overall yields and excellent enantioselectivities.

## Supplementary Material



## Data Availability

The data underlying this study are available in the published article, in its Supporting Information, and openly available in Zenodo at 10.5281/zenodo.17417754

## Notes

Experimental procedures, characterization data, NMR spectra, and HPLC traces (PDF)
