# Supplementary material for: Enantioselective Synthesis of 3‑Alkyl-1,5-Functionalized 1,4-Diynes via Isoxazol-5(4H)‑ones
Source: Org Lett. 2026 Jan 14;28(4):1121–6. doi: 10.1021/acs.orglett.5c04457 (PMC12865758; doi:10.1021/acs.orglett.5c04457)
Supplement: Supplementary file 1 [file ol5c04457_si_001.pdf]

SUPPORTING INFORMATION

Enantioselective Synthesis of 3-Alkyl-1,5-Functionalized-1,4-Diynes via Isoxazol-5(4*H*)-ones

Ricardo Torán, Sergi Vercher, Pablo López, Amparo Sanz-Marco,* Marc Montesinos-Magraner, Carlos Vila and Gonzalo Blay*

Departament de Química Orgànica, Facultat de Química, Universitat de València, C. Dr. Moliner 50, 46100-Burjassot, Spain

Index:

Materials and methods	S2
Synthesis and characterization data of products **3**	S2
Synthesis of compound **3aa** at one mmol scale	S12
Synthesis and characterization data of products **4**	S12
Synthesis of compound **4aa** from **3aa**	S19
Synthesis and characterization data for compound **5**	S19
Mechanism of the Zard reaction (nitrosative cleavage of isoxazolinones) and role of $FeSO_4$	S21
NMR spectra for compounds **3**, **4** and **5**	S22
HPLC traces for compounds **3**, **4** and **5**	S56
Stereochemical model for the enantioselective Michael addition of isoxazolinones to nitroenynes	S90
Computational methods	S90
References	S158

Materials and methods

All reagents were purchased from commercial suppliers and used without further purification. All solvents employed in the reactions were distilled from appropriate drying agents prior to use. Reactions were monitored by TLC analysis using Merck Silica Gel 60 F-254 thin layer plates. Flash column chromatography was performed on Merck silica gel 60, 0.040-0.063 mm. Melting points were determined in capillary tubes. NMR spectra were run at 300 MHz and 500 MHz for ^{1}H and at 75 MHz and 125 MHz for ^{13}C NMR using residual nondeuterated solvent ($CHCl_3$) as internal standard (δ 7.26 and 77.0 ppm, respectively). Chemical shifts are given in ppm. The carbon type was determined by DEPT experiments. High resolution mass spectra (ESI) were recorded on a Q-TOF spectrometer equipped with an electrospray source with a capillary voltage of 3.3 kV (ESI). Specific optical rotations were measured using sodium light (D line 589 nm). Chiral HPLC analyses were performed in a chromatograph equipped with a UV diode-array detector using chiral stationary phase columns from Daicel or Phenomenex. Isoxazol-5(4*H*)-ones[1] **1** and nitroenynes[2] **2** were prepared according to literature procedures.

Synthesis and characterization data of products 3

General procedure for the enantioselective synthesis of 3

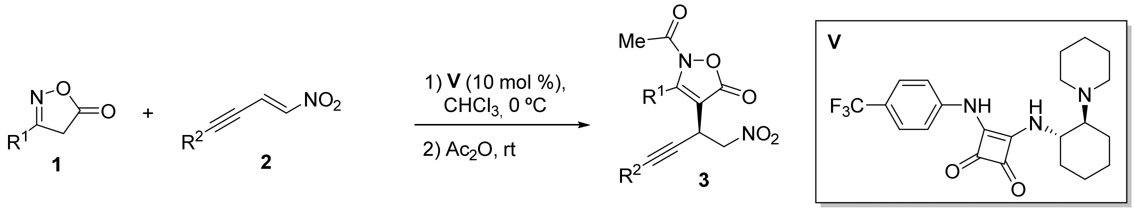

In a test tube, chiral catalyst **V** (4.2 mg, 0.01 mmol), isoxazol-5(4*H*)-one **1** (0.1 mmol), and nitroenyne **2** (0.1 mmol) were introduced. Then, chloroform (1 mL) is added under a nitrogen atmosphere, and the mixture is stirred at 0 ºC until isoxazol-5(4*H*)-one **1** is consumed. Acetic anhydride (18.9 μL, 0.2 mmol) was added, and the reaction was allowed to proceed for 30 minutes at room temperature. Finally, column chromatography was performed using a mixture of Hexane:EtOAc as the eluent to obtain compound **3**. Racemic compounds were prepared by following a similar procedure with an achiral squaramide.

(*S*)-2-Acetyl-4-(4-(4-chlorophenyl)-1-nitrobut-3-yn-2-yl)-3-phenylisoxazol-5(2*H*)-one (3aa)

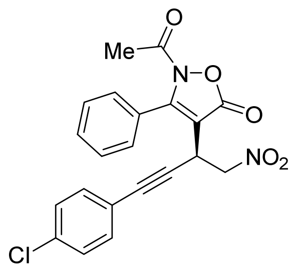

From isoxazol-5(4*H*)-one **1a** (16.1 mg) and nitroenyne **2a** (20.7 mg), 23.4 mg (70%) of compound **3aa** were obtained. The enantiomeric excess (92%) was determined by HPLC (Chiralpak IC), hexane:^{i}PrOH 80:20, 1.0 mL min^{-1}, major enantiomer: t_r = 19.7 min, minor enantiomer: t_r = 16.7 min.

Yellow oil; $[\alpha]_D^{25}$ = -186.5 (c = 0.47, $CHCl_3$); ^{1}H NMR (300 MHz, $CDCl_3$) δ 7.56-7.44 (m, 5H), 7.29-7.26 (m, 4H), 5.07-5.00 (dd, J = 13.2, 9.1 Hz, 1H), 4.81-4.75 (dd, J = 13.2, 6.1 Hz, 1H), 4.38-4.33 (dd, J = 9.1, 6.1 Hz, 1H), 2.45 (s, 3H); ^{13}C NMR (75 MHz, $CDCl_3$) δ 164.5 (C), 164.2 (C), 156.5 (C), 135.0 (C), 133.1 (CH), 131.4 (CH), 129.2 (CH), 128.8 (CH),128.7 (CH), 128.4 (CH), 126.6 (CH), 126.1 (C), 121.3 (CH), 120.1 (C), 102.9 (C), 84.0 (C), 83.1 (C), 74.2 (CH_2), 25.9 (CH_3), 22.9 (CH); HRMS (ESI) *m/z*: 411.0740 $[M+H]^+$, $C_{21}H_{16}ClN_2O_5^+$ requires 411.0742.

(*S*)-2-Acetyl-4-(4-(4-chlorophenyl)-1-nitrobut-3-yn-2-yl)-3-(4-methoxyphenyl)isoxazol-5(2*H*)-one (3ba)

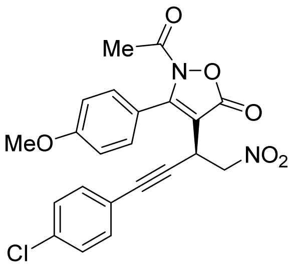

From isoxazol-5(4*H*)-one **1b** (19.1 mg) and nitroenyne **2a** (20.8 mg), 22.6 mg (51%) of compound **3ba** were obtained. The enantiomeric excess (92%) was determined by HPLC (Chiralpak AD-H), hexane:^{i}PrOH 80:20, 1.0 mL min^{-1}, major enantiomer: t_r = 22.4 min, minor enantiomer: t_r = 26.7 min.

Yellow oil; $[\alpha]_D^{25}$ = -279.9 (c = 0.47, $CHCl_3$); ^{1}H NMR (300 MHz, $CDCl_3$) δ 7.42 (d, J = 8.9 Hz, 2H), 7.32-7.29 (m, 4H), 7.03 (d, J = 8.9 Hz, 2H), 5.03 (dd, J = 13.2, 8.9 Hz, 1H), 4.81 (dd, J = 13.2, 6.3 Hz, 1H), 4.40 (dd, J = 8.9, 6.3 Hz, 1H), 3.87 (s, 3H), 2.45 (s, 3H); ^{13}C NMR (75 MHz, $CDCl_3$) δ 164.7 (C), 164.6 (C), 162.0 (C), 156.8 (C), 135.0 (C), 133.1 (2CH), 130.4 (2CH), 128.7 (2CH), 120.2 (C), 117.8 (C), 114.3 (2CH), 102.2 (C), 83.9 (C), 83.4 (C), 74.3 (CH_2), 55.4 (CH_3), 26.1 (CH_3), 23.1 (CH); HRMS (ESI) *m/z*: 441.0841 $[M+H]^+$, $C_{22}H_{18}ClN_2O_6^+$ requires 441.0848.

(S)-2-Acetyl-3-(4-bromophenyl)-4-(4-(4-chlorophenyl)-1-nitrobut-3-yn-2-yl)isoxazol-5(2H)-one (3ca)

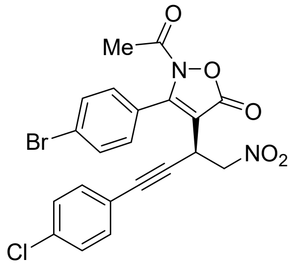

From isoxazol-5(4H)-one **1c** (24.0 mg) and nitroenyne **2a** (20.8 mg), 33.5 mg (69%) of compound **3ca** were obtained. The enantiomeric excess (92%) was determined by HPLC (Chiralpak AD-H), hexane:^{i}PrOH 80:20, 1.0 mL min^{-1}, major enantiomer: t_r = 16.6 min, minor enantiomer: t_r = 19.6 min.

Yellow oil; **[α]$_D^{25}$** = -280.0 (c = 0.47, $CHCl_3$); ^{1}H NMR (300 MHz, $CDCl_3$) δ 7.68-7.65 (d, 2H), 7.35-7.32 (d, 2H), 7.28 (s, 4H), 5.11-5.03 (dd, J = 13.3, 9.2 Hz, 1H), 4.80-4.74 (dd, J = 13.3, 5.7 Hz, 1H), 4.35-4.30 (dd, J = 9.2, 5.7 Hz, 1H), 2.46 (s, 3H); ^{13}C NMR (75 MHz, $CDCl_3$) δ 164.4 (C), 155.4 (C), 135.2 (C), 133.1 (2CH), 132.1 (2CH), 130.0 (2CH), 128.7 (2CH), 126.2 (C), 124.9 (C), 119.9 (C), 103.2 (C), 96.6 (C), 84.3 (C), 82.7 (C), 74.1 (CH_2), 26.0 (CH_3), 22.9 (CH); HRMS (ESI) m/z: 488.9847 [M+H]$^+$, $C_{21}H_{15}BrClN_2O_5^+$ requires 488.9847.

2-acetyl-4-(4-(4-chlorophenyl)-1-nitrobut-3-yn-2-yl)-3-(thiophen-2-yl)isoxazol-5(2H)-one (3da)

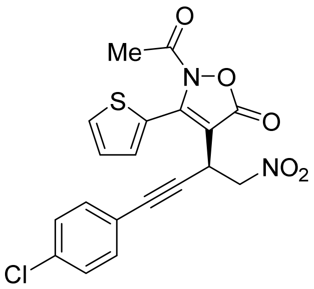

From isoxazol-5(4H)-one **1d** (16.7 mg) and nitroenyne **2a** (20.8 mg), 23.5 mg (56%) of compound **3da** were obtained. The enantiomeric excess (84%) was determined by HPLC (Chiralpak IC), hexane:^{i}PrOH 90:10, 1.0 mL min^{-1}, major enantiomer: t_r = 37.0 min, minor enantiomer: t_r = 30.6 min.

Yellow oil; [α]$_D^{25}$ = -164.7 (c = 0.17, $CHCl_3$); ^{1}H NMR (300 MHz, $CDCl_3$) δ 7.73-7.70 (dd, J= 5.1, 1.2 Hz, 1H), 7.46-7.44 (dd, J= 3.7, 1.2 Hz, 1H), 7.35-7.28 (m, 4H), 7.23-7.20 (dd, J = 5.1, 3.7 Hz, 1H), 5.10-5.03 (dd, J = 13.3, 8.9 Hz, 1H), 4.88-4.81 (dd, J= 13.3, 6.3 Hz, 1H), 4.57-4.52 (dd, J= 8.8, 6.2 Hz, 1H), 2.47 (s, 3H); ^{13}C NMR (75 MHz, $CDCl_3$) δ 164.7 (C), 164.3 (C), 150.7 (C), 135.1 (C), 133.1 (2CH), 132.1 (CH), 131.5 (CH), 128.7 (2CH), 127.6 (CH), 124.9 (C), 120.1 (C), 103.8 (C), 84.0 (C), 83.1 (C), 74.2 (CH_2), 26.4 (CH_3), 23.1 (CH); HRMS (ESI) m/z: 439.0121 [M+Na]$^+$, $C_{19}H_{13}ClN_2NaO_5S^+$ requires 439.0126.

2-Acetyl-4-(4-(4-chlorophenyl)-1-nitrobut-3-yn-2-yl)-3-methylisoxazol-5(2*H*)-one (3ea)

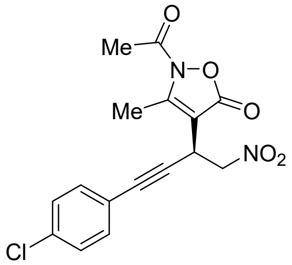

From isoxazol-5(4*H*)-one **1e** (9.9 mg) and nitroenyne **2a** (20.8 mg), 31.2 mg (90%) of compound **3ea** were obtained. The enantiomeric excess (48%) was determined by HPLC (Chiralpak AD-H), hexane:^{i}PrOH 80:20, 1.0 mL min^{-1}, major enantiomer: t_r = 16.7 min, minor enantiomer: t_r = 15.7 min.

Yellow oil; $[\alpha]_D^{25}$ = -54.3 (*c* = 0.26, $CHCl_3$); ^{1}H NMR (300 MHz, $CDCl_3$) δ 7.35-7.32 (m, 2H), 7.30-7.27 (m, 2H), 4.99-4.92 (dd, *J*= 13.4, 8.5 Hz, 1H), 4.82-4.76 (dd, *J*= 13.4, 6.1 Hz, 1H), 4.55-4.50 (dd, *J*= 8.4, 6.1 Hz, 1H), 2.72 (s, 3H), 2.45 (s, 3H); ^{13}C NMR (125 MHz, $CDCl_3$) δ 165.1 (C), 164.5 (C), 155.7 (C), 135.1 (C), 133.1 (2CH), 128.8 (2CH), 120.1 (C), 101.1 (C), 95.0 (C), 84.0 (C), 82.7 (C), 74.8 (CH_2), 25.6 (CH_3), 22.6 (CH_3), 13.7 (CH); HRMS (ESI) *m/z*: 349.0583 [M+Na]$^+$, $C_{16}H_{14}ClN_2O_5^+$ requires 349.0586. Compound **3ea** was obtained slightly contaminated with a by-product resulting from the acetylation of unreacted isoxazolinone **1e**

(*S*)-2-Acetyl-4-(4-(3-chlorophenyl)-1-nitrobut-3-yn-2-yl)-3-phenylisoxazol-5(2*H*)-one (3ab)

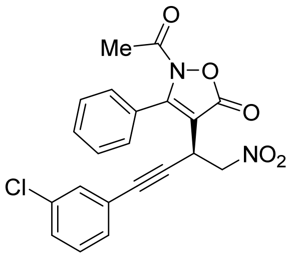

From isoxazol-5(4*H*)-one **1a** (16.1 mg) and nitroenyne **2b** (20.8 mg), 21.7 mg (52%) of compound **3ab** were obtained. The enantiomeric excess (86%) was determined by HPLC (Chiralpak IC), hexane:^{i}PrOH 80:20, 1.0 mL min^{-1}, major enantiomer: t_r = 19.0 min, minor enantiomer: t_r = 16.2 min.

Yellow oil; $[\alpha]_D^{25}$ = -255.1 (*c* = 0.43, $CHCl_3$); ^{1}H NMR (300 MHz, $CDCl_3$) δ 7.60-7.42 (m, 5H),7.38-7.29 (m, 2H), 7.28-7.20 (m, 2H), 5.04 (dd, *J* = 13.2, 9.1 Hz, 1H), 4.78 (dd, *J* = 13.2, 6.1 Hz, 1H), 4.36 (dd, *J* = 9.0, 6.0 Hz, 1H), 2.45 (s, 3H); ^{13}C NMR (75 MHz, $CDCl_3$) δ 164.5 (C), 164.2 (C), 156.6 (C), 134.1 (C), 131.8 (CH), 131.4 (CH), 130.0 (CH), 129.5 (CH), 129.2 (CH), 128.8 (2CH), 128.4 (2CH), 126.0 (C), 123.3 (C), 102.8 (C), 83.6 (C), 83.4 (C), 74.2 (CH_2), 25.9 (CH_3), 23.0 (CH); HRMS (ESI) *m/z*: 411.0728 [M+H]$^+$, $C_{21}H_{16}ClN_2O_5^+$ requires 411.0743.

(*S*)-2-Acetyl-4-(4-(3-chlorophenyl)-1-nitrobut-3-yn-2-yl)-3-(4-methoxyphenyl)isoxazol-5(2*H*)-one (3bb)

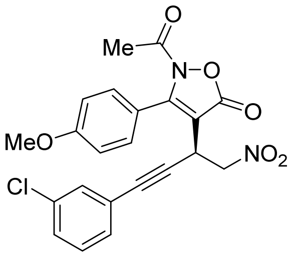

From isoxazol-5(4*H*)-one **1b** (19.1 mg) and nitroenyne **2b** (20.8 mg), 26.0 mg (59%) of compound **3bb** were obtained. The enantiomeric excess (85%) was determined by HPLC (Chiralpak IC), hexane:^{i}PrOH 80:20, 1.0 mL min^{-1}, major enantiomer: t_r = 28.1 min, minor enantiomer: t_r = 25.2 min.

Yellow oil; $[\alpha]_D^{25}$ = -195.4 (*c* = 0.52, $CHCl_3$); ^{1}H NMR (300 MHz, $CDCl_3$) δ 7.42 (d, *J* = 8.8 Hz, 2H), 7.37-7.34 (m, 1H), 7.31 (dt, *J* = 7.1, 2.1 Hz, 1H), 7.26-7.19 (m, 3H), 7.03 (d, *J* = 8.8 Hz, 2H), 5.04 (dd, *J* = 13.2, 8.8 Hz, 1H), 4.81 (dd, *J* = 13.2, 6.3 Hz, 1H), 4.41 (dd, *J* = 8.8, 6.3 Hz, 1H), 3.88 (s, 3H), 2.45 (s, 3H); ^{13}C NMR (75 MHz, $CDCl_3$) δ 164.6 (C), 162.0 (C), 156.8 (C), 134.2 (C), 131.8 (CH), 130.4 (2CH), 130.0 (CH), 129.6 (CH), 129.2 (CH), 123.4 (C), 117.8 (C), 114.3 (2CH), 102.1 (C), 83.7 (C), 83.6 (C), 74.2 (CH_2), 55.4 (CH_3), 26.0 (CH_3), 23.1 (CH); HRMS (ESI) *m/z*: 441.0835 [M+Na]$^+$, $C_{22}H_{18}ClN_2O_6^+$ requires 441.0848.

(*S*)-2-Acetyl-3-(4-chlorophenyl)-4-(4-(3-chlorophenyl)-1-nitrobut-3-yn-2-yl)isoxazol-5(2*H*)-one (3fb)

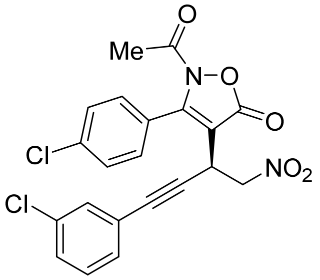

From isoxazol-5(4*H*)-one **1f** (19.6 mg) and nitroenyne **2b** (20.7 mg), 21.5 mg (48%) of compound **3fb** were obtained. The enantiomeric excess (84%) was determined by HPLC (Chiralpak IC), hexane:^{i}PrOH 90:10, 1.0 mL min^{-1}, major enantiomer: t_r = 30.3 min, minor enantiomer: t_r = 28.8 min.

Yellow oil; $[\alpha]_D^{25}$ = -254.2 (*c* = 0.43, $CHCl_3$); ^{1}H NMR (500 MHz, $CDCl_3$) δ 7.51 (d, *J* = 8.7 Hz, 2H), 7.41-7.40 (m, 2H), 7.35-7.32 (m, 2H), 7.25-7.23 (m, 2H), 5.08 (dd, *J* = 13.3, 9.2 Hz, 1H), 4.79 (dd, *J* = 13.3, 5.7 Hz, 1H), 4.33 (dd, *J* = 9.2, 5.7 Hz, 1H), 2.46 (s, 3H); ^{13}C NMR (125 MHz, $CDCl_3$) δ 164.4 (C), 164.3 (C), 155.4 (C), 137.9 (C), 134.2 (C), 131.7 (CH), 130.0 (CH), 129.8 (2CH), 129.6 (CH), 129.4 (CH), 129.2 (2CH), 124.4 (C), 123.1 (C),103.2 (C), 84.0 (C), 83.0 (C), 74.0 (CH_2), 25.9 (CH_3), 22.9 (CH); HRMS (ESI) *m/z*: 445.0346 [M+H]$^+$, $C_{21}H_{15}Cl_2N_2O_5^+$ requires 445.0353.

(*S*)-2-Acetyl-3-(4-methoxyphenyl)-4-(1-nitro-4-phenylbut-3-yn-2-yl)isoxazol-5(2*H*)-one (3bc)

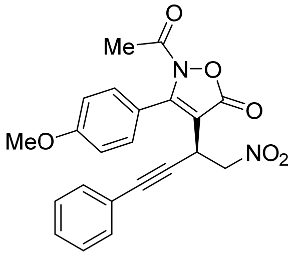

From isoxazol-5(4*H*)-one **1b** (19.1 mg) and nitroenyne **2c** (17.3 mg), 24.8 mg (61%) of compound **3bc** were obtained. The enantiomeric excess (94%) was determined by HPLC (Chiralpak AD-H), hexane:^{i}PrOH 80:20, 1.0 mL min^{-1}, major enantiomer: t_r = 17.9 min, minor enantiomer: t_r = 20.0 min.

Yellow oil; $[\alpha]_D^{25}$ = -235.7 (*c* = 0.50, $CHCl_3$); ^1H NMR (300 MHz, $CDCl_3$) δ 7.44 (d, *J* = 8.9 Hz, 2H), 7.41-7.37 (m, 2H), 7.34-7.29 (m, 3H), 7.03 (d, *J* = 8.9 Hz, 2H), 5.06 (dd, *J* = 13.2, 9.1 Hz, 1H), 4.81 (dd, *J* = 13.2, 6.1 Hz, 1H), 4.41 (dd, *J* = 9.0, 6.1 Hz, 1H), 3.87 (s, 3H), 2.45 (s, 3H); ^{13}C NMR (75 MHz, $CDCl_3$) δ 164.7 (C), 164.6 (C), 162.0 (C), 131.9 (2CH), 130.4 (2CH), 128.9 (CH), 128.3 (2CH), 121.7 (C), 117.9 (C), 114.2 (2CH), 102.5 (C), 84.9 (C), 82.4 (C), 74.3 (CH_2), 55.4 (CH_3), 26.1 (CH_3), 23.1 (CH); HRMS (ESI) *m/z*: 407.1228 $[M+H]^+$, $C_{22}H_{19}N_2O_6^+$ requires 407.1238.

(*S*)-2-Acetyl-4-(1-nitro-4-phenylbut-3-yn-2-yl)-3-(*p*-tolyl)isoxazol-5(2H)-one (3gc)

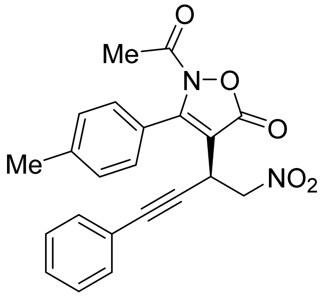

From isoxazol-5(4*H*)-one **1g** (17.5 mg) and nitroenyne **2c** (17.3 mg), 24.6 mg (63%) of compound **3gc** were obtained. The enantiomeric excess (86%) was determined by HPLC (Chiralpak IC), hexane:^{i}PrOH 80:20, 1.0 mL min^{-1}, major enantiomer: t_r = 22.6 min, minor enantiomer: t_r = 20.2 min.

Yellow oil; $[\alpha]_D^{25}$ = -28.8 (*c* = 0.08, $CHCl_3$); ^1H NMR (300 MHz, $CDCl_3$) δ 7.33-7.22 (m, 9H), 4.98 (dd, *J* = 13.2, 9.1 Hz, 1H), 4.72 (dd, *J* = 13.2, 6.1 Hz, 1H), 4.31 (dd, *J* = 9.1, 6.1 Hz, 1H), 2.38 (s, 3H), 2.37 (s, 3H); ^{13}C NMR (125 MHz, $CDCl_3$) δ 164.6 (C), 164.4 (C), 156.8 (C), 141.9 (C), 131.9 (2CH), 129.5 (2CH), 128.9 (CH), 128.4 (2CH), 128.3 (2CH), 123.1 (C), 121.7 (C), 102.9 (C), 85.0 (C), 82.3 (C), 74.3 (CH_2), 26.0 (CH_3), 23.0 (CH), 21.6 (CH_3); HRMS (ESI) *m/z*: 391.1273 $[M+H]^+$, $C_{22}H_{19}N_2O_5^+$ requires 391.1288.

(*S*)-2-Acetyl-3-(4-chlorophenyl)-4-(1-nitro-4-phenylbut-3-yn-2-yl)isoxazol-5(2*H*)-one (3fc)

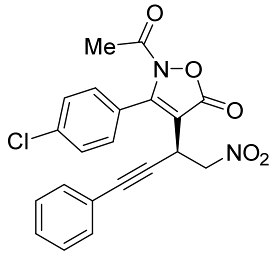

From isoxazol-5(4*H*)-one **1f** (19.5 mg) and nitroenyne **2c** (17.3 mg), 16.5 mg (40%) of compound **3fc** were obtained. The enantiomeric excess (83%) was determined by HPLC (Chiralpak AD-H), hexane:^{i}PrOH 95:5, 1.0 mL min^{-1}, major enantiomer: t_r = 39.5 min, minor enantiomer: t_r = 42.6 min.

Yellow oil; $[\alpha]_D^{25}$ = -246.7 (c = 0.21, $CHCl_3$); ^{1}H NMR (300 MHz, $CDCl_3$) δ 7.51 (d, J = 8.7 Hz, 1H), 7.42 (d, J = 8.8 Hz, 1H), 5.09 (dd, J = 13.3, 9.4 Hz, 1H), 4.77 (dd, J = 13.3, 5.7 Hz, 1H), 4.34 (dd, J = 9.4, 5.6 Hz, 1H), 2.45 (s, 3H); ^{13}C NMR (75 MHz, $CDCl_3$) δ 164.4 (C), 164.3 (C), 155.3 (C), 137.7 (C), 131.8 (2CH), 129.9 (2CH), 129.2 (2CH), 129.0 (CH), 128.3 (2CH), 124.5 (C), 121.5 (C), 103.5 (C), 74.1 (CH_2), 25.9 (CH_3), 22.9 (CH); HRMS (ESI) *m/z*: 411.0722 $[M+H]^+$, $C_{21}H_{16}ClN_2O_5^+$ requires 411.0743.

(*S*)-2-Acetyl-4-(4-(4-methoxyphenyl)-1-nitrobut-3-yn-2-yl)-3-phenylisoxazol-5(2*H*)-one (3ad)

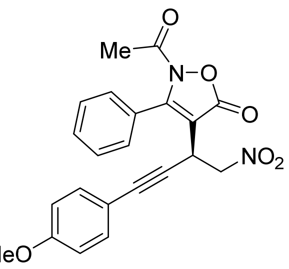

From isoxazol-5(4*H*)-one **1a** (16.1 mg) and nitroenyne **2d** (20.3 mg), 25.3 mg (62%) of compound **3ad** were obtained. The enantiomeric excess (91%) was determined by HPLC (Chiralpak AD-H), hexane:^{i}PrOH 80:20, 1.0 mL min^{-1}, major enantiomer: t_r = 31.2 min, minor enantiomer: t_r = 24.6 min.

Yellow oil; $[\alpha]_D^{25}$ = -290.3 (c = 0.51, $CHCl_3$); ^{1}H NMR (300 MHz, $CDCl_3$) δ 7.57-7.45 (m, 5H), 7.33-7.30 (m, 2H), 6.83-6.80 (m, 2H), 5.09-5.02 (dd, J = 13.2, 9.3 Hz, 1H), 4.79-4.73 (dd, J = 13.2, 5.9 Hz, 1H), 4.37-4.32 (dd, J = 9.3, 5.9 Hz, 1H), 3.80 (s, 3H), 2.44 (s, 3H); ^{13}C NMR (75 MHz, $CDCl_3$) δ 164.6 (C), 164.3 (C), 160.0 (C), 156.4 (C), 133.4 (2CH), 131.3 (CH), 128.7 (2CH), 128.5 (2CH), 126.2 (C), 113.9 (2CH), 113.7 (C), 103.4 (C), 85.0 (C), 80.8 (C), 74.3 (CH_2), 55.3 (CH_3), 26.0 (CH_3), 22.9 (CH); HRMS (ESI) *m/z*: 407.1229 $[M+H]^+$, $C_{22}H_{19}N_2O_6^+$ requires 407.1238.

(*S*)-2-Acetyl-3-(4-chlorophenyl)-4-(4-(4-methoxyphenyl)-1-nitrobut-3-yn-2-yl)isoxazol-5(2*H*)-one (3fd)

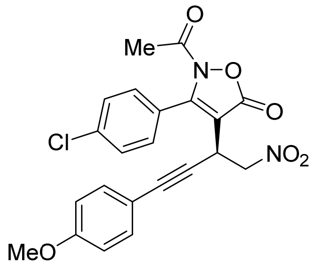

From isoxazol-5(4*H*)-one **1f** (19.5 mg) and nitroenyne **2d** (20.3 mg), 37.7 mg (86%) of compound **3fd** were obtained. The enantiomeric excess (92%) was determined by HPLC (Chiralpak AD-H), hexane:^{i}PrOH 95:5, 1.0 mL min^{-1}, major enantiomer: t_r = 64.8 min, minor enantiomer: t_r = 59.4 min.

White solid; m.p. = 122.3-123.4 ºC; $[\alpha]_D^{25}$ = -247.9 (c = 0.75, $CHCl_3$). ^{1}H NMR (300 MHz, $CDCl_3$) δ 7.52-7.48 (m, 2H), 7.44-7.40 (m, 2H), 7.31-7.28 (m, 2H), 6.85-6.81 (m, 2H), 5.12-5.05 (dd, J = 13.3, 9.4 Hz, 1H), 4.79-4.73 (dd, J = 13.3, 5.6 Hz, 1H), 4.34-4.29 (dd, J = 9.4, 5.6 Hz, 1H), 3.81 (s, 3H), 2.45 (s, 3H); ^{13}C NMR (75 MHz, $CDCl_3$) δ 164.4 (C), 164.4 (C), 160.1 (C), 155.2 (C), 137.7 (C), 133.4 (2CH), 129.9 (CH), 129.2 (2CH), 124.6 (C), 114.0 (2CH), 113.5 (C), 103.8 (C), 85.4 (C), 80.4 (C), 74.23 (CH_2), 55.3 (CH_3), 26.0 (CH_3), 22.9 (CH); HRMS (ESI) *m/z*: 441.0837 $[M+H]^+$, $C_{22}H_{18}ClN_2O_6^+$ requires 441.0848.

(*S*)-2-Acetyl-4-(4-(3-methoxyphenyl)-1-nitrobut-3-yn-2-yl)-3-phenylisoxazol-5(2*H*)-one (3ae)

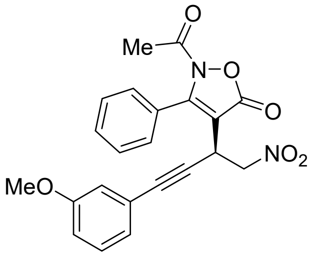

From isoxazol-5(4*H*)-one **1a** (16.1 mg) and nitroenyne **2e** (20.8 mg), 30.1 mg (74%) of compound **3ae** were obtained. The enantiomeric excess (91%) was determined by HPLC (Chiralpak IG), hexane:^{i}PrOH 80:20, 1.0 mL min^{-1}, major enantiomer: t_r = 30.2 min, minor enantiomer: t_r = 27.2 min.

Yellow oil; $[\alpha]_D^{25}$ = -231.6 (c = 0.60, $CHCl_3$); ^{1}H NMR (300 MHz, $CDCl_3$) δ 7.61-7.41 (m, 5H), 7.25-7.15 (m, 1H), 6.98 (dt, J = 7.6, 1.2 Hz, 1H), 6.91-6.86 (m, 2H), 5.07 (dd, J = 13.2, 9.3 Hz, 1H), 4.78 (dd, J = 13.3, 5.9 Hz, 1H), 4.36 (dd, J = 9.3, 5.9 Hz, 1H), 3.79 (s, 3H), 2.45 (s, 3H); ^{13}C NMR (75 MHz, $CDCl_3$) δ 164.5 (C), 164.3 (C), 159.3 (C), 156.5 (C), 131.4 (CH), 129.4 (CH), 128.8 (2CH), 128.5 (2CH), 126.1 (C), 124.4 (CH), 122.6 (C), 116.7 (CH), 115.5 (CH), 103.1 (C), 84.9 (C), 82.0 (C), 74.2 (CH_2), 55.3 (CH_3), 25.9 (CH_3), 22.9 (CH); HRMS (ESI) *m/z*: 407.1220 $[M+H]^+$, $C_{22}H_{19}N_2O_6^+$ requires 407.1238.

(*S*)-2-Acetyl-4-(1-nitro-6-phenylhex-3-yn-2-yl)-3-phenylisoxazol-5(2*H*)-one (3af)

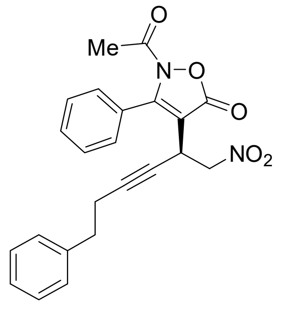

From isoxazol-5(4*H*)-one **1a** (16.1 mg) and nitroenyne **2f** (20.1 mg), 17.7 mg (44%) of compound **3af** were obtained. The enantiomeric excess (92%) was determined by HPLC (Chiralpak AS-H), hexane:^{i}PrOH 80:20, 1.0 mL min^{-1}, major enantiomer: t_r = 20.6 min, minor enantiomer: t_r = 26.4 min.

Yellow oil; $[\alpha]_D^{25}$ = -128.8 (*c* = 0.35, $CHCl_3$); ^1H NMR (300 MHz, $CDCl_3$) δ 7.56-7.46 (m, 3H), 7.38-7.35 (m, 2H), 7.31-7.26 (m, 2H), 7.24-7.17 (m, 3H), 4.97-4.90 (dd, *J* = 13.2, 9.3 Hz, 1H), 4.63-4.57 (dd, *J* = 13.2, 5.9 Hz, 1H), 4.08-4.03 (m, 1H), 2.80-2.75 (m, 2H), 2.47-2.42 (m, 5H); ^{13}C NMR (75 MHz, $CDCl_3$) δ 164.5 (C), 164.2 (C), 156.2 (C), 140.2 (C), 131.2 (CH), 128.6 (2CH), 128.5 (2CH), 128.4 (2CH), 128.3 (2CH), 126.3 (2CH), 126.1 (C), 103.6 (C), 85.0 (2C), 74.3 (CH_2), 34.5 (CH_2), 25.3 (CH_3), 22.9 (CH), 20.8 (CH_2); HRMS (ESI) *m/z*: 405.1432 $[M+H]^+$, $C_{23}H_{21}N_2O_5^+$ requires 405.1445.

(*S*)-2-Acetyl-3-(4-methoxyphenyl)-4-(1-nitro-6-phenylhex-3-yn-2-yl)isoxazol-5(2*H*)-one (3bf)

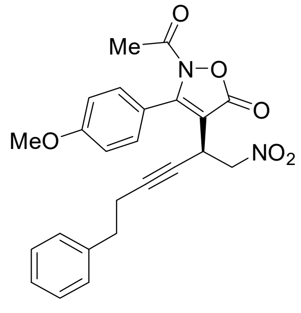

From isoxazol-5(4*H*)-one **1b** (19.1 mg) and nitroenyne **2f** (20.1 mg), 24.3 mg (56%) of compound **3bf** were obtained. The enantiomeric excess (>99%) was determined by HPLC (Chiralpak IC), hexane:^{i}PrOH 80:20, 1.0 mL min^{-1}, major enantiomer: t_r = 31.6 min.

Yellow oil; $[\alpha]_D^{25}$ = -140.9 (*c* = 0.49, $CHCl_3$); ^1H NMR (300 MHz, $CDCl_3$) δ 7.34-7.39 (m, 3H), 7.24-7.17 (m, 4H), 6.99 (d, *J* = 8.7 Hz, 2H), 4.93 (dd, *J* = 13.2, 9.2 Hz, 1H), 4.63 (dd, *J* = 13.2, 6.1 Hz, 1H), 4.15-4.04 (m, 1H), 3.90 (s, 3H), 2.81-2.76 (m, 2H), 2.51-2.40 (m, 5H); ^{13}C NMR (75 MHz, $CDCl_3$) δ 164.7 (C), 164.6 (C), 161.9 (C), 156.4 (C), 140.2 (C), 130.4 (2CH), 128.5 (2CH), 128.4 (2CH), 126.3 (CH), 117.9 (C), 114.1 (2CH), 103.0 (C), 84.9 (C), 74.5 (C), 74.3 (CH_2), 55.4 (CH_3), 34.6 (CH_2), 25.5 (CH_3), 23.1 (CH), 20.8 (CH_2); HRMS (ESI) *m/z*: 435.1543 $[M+H]^+$, $C_{24}H_{23}N_2O_6^+$ requires 435.1551.

(*S*)-2-Acetyl-3-(4-chlorophenyl)-4-(1-nitro-6-phenylhex-3-yn-2-yl)isoxazol-5(2*H*)-one (3ff)

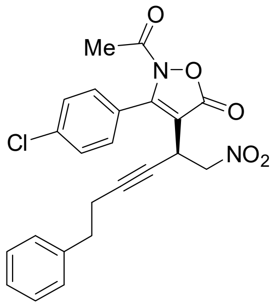

From isoxazol-5(4*H*)-one **1f** (19.6 mg) and nitroenyne **2f** (20.1 mg), 20.2 mg (46%) of compound **3ff** were obtained. The enantiomeric excess (88%) was determined by HPLC (Chiralpak IC), hexane:^{i}PrOH 80:20, 1.0 mL min^{-1}, major enantiomer: t_r = 16.6 min, minor enantiomer: t_r = 16.1 min.

Yellow oil; $[\alpha]_D^{25}$ = -125.7 (c = 0.40, $CHCl_3$); ^{1}H NMR (300 MHz, $CDCl_3$) δ 7.46 (d, J = 8.5 Hz, 2H), 7.32-7.28 (m, 3H), 7.25-7.15 (m, 4H), 4.96 (dd, J = 13.4, 9.5 Hz, 1H), 4.59 (dd, J = 13.4, 5.7 Hz, 1H), 4.14-3.89 (m, 1H), 2.79-2.75 (m, 2H), 2.47-2.41 (m, 5H); ^{13}C NMR (75 MHz, $CDCl_3$) δ 164.4 (C), 164.3 (C), 155.0 (C), 140.1 (C), 137.6 (C), 129.9 (2CH), 129.1 (2CH), 128.5 (2CH), 128.4 (2CH), 126.4 (CH), 124.5 (C), 104.0 (C), 85.3 (C), 74.1 (CH_2), 74.0 (C), 34.5 (CH_2), 25.3 (CH_3), 22.9 (CH), 20.7 (CH_2); HRMS (ESI) *m/z*: 439.1040 $[M+H]^+$, $C_{23}H_{20}ClN_2O_5^+$ requires 439.1056.

(*S*)-2-Acetyl-4-(1-nitrohex-3-yn-2-yl)-3-phenylisoxazol-5(2*H*)-one (3ag)

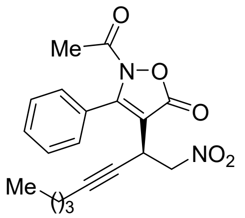

From isoxazol-5(4*H*)-one **1a** (10.6 mg) and nitroenyne **2g** (10.1 mg), 10.5 mg (44%) of compound **3ag** were obtained. The enantiomeric excess (83%) was determined by HPLC (Chiralpak IC) hexane:^{i}PrOH 80:20, 1.0 mL min^{-1}, major enantiomer: t_r = 13.8 min, minor enantiomer: t_r = 11.6 min.

Yellow oil; $[\alpha]_D^{25}$ = -86.1 (c = 0.20, $CHCl_3$); ^{1}H NMR (300 MHz, $CDCl_3$) δ 7.60-7.46 (m, 3H), 7.45-7.40 (m, 2H), 4.94 (dd, J = 13.1, 9.2 Hz, 1H), 4.65 (dd, J = 13.1, 6.0 Hz, 1H), 4.10 (dd, J = 9.2, 6.0 Hz, 1H), 2.43 (s, 3H), 2.13 (td, J = 6.9, 2.3 Hz, 2H), 1.49-1.29 (m, 4H), 0.90 (t, J = 7.2 Hz, 3H); ^{13}C NMR (75 MHz, $CDCl_3$) δ 164.6 (C), 164.3 (C), 156.2 (C), 131.2 (CH), 128.7 (2CH), 128.5 (2CH), 126.2 (C), 103.8 (C), 86.0 (C), 74.6 (CH_2), 73.1 (C), 30.4 (CH_2), 25.5 (CH_3), 22.9 (CH), 21.9 (CH_2), 18.3 (CH_2), 13.5 (CH_3); HRMS (ESI) *m/z*: 357.1433 $[M+H]^+$, $C_{19}H_{21}N_2O_5^+$ requires 357.1445.

(*R*)-2-Acetyl-4-(1-nitro-4-(triisopropylsilyl)but-3-yn-2-yl)-3-phenylisoxazol-5(2*H*)-one (3ah)

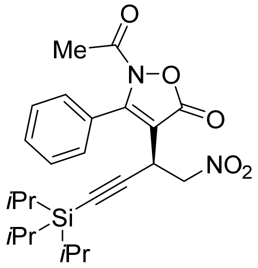

From isoxazol-5(4*H*)-one **1a** (10.6 mg) and nitroenyne **2h** (25.3 mg), 32.5 mg (71%) of compound **3ah** were obtained. The enantiomeric excess (90%) was determined by HPLC (Chiralpak AD-H) hexane:^{i}PrOH 90:10, 1.0 mL min^{-1}, major enantiomer: t_r = 6.2 min, minor enantiomer: t_r = 5.6 min

Yellow oil; $[\alpha]_D^{25}$ = -356.5 (*c* = 0.17, $CHCl_3$); ^{1}H NMR (500 MHz, $CDCl_3$) δ 7.64 – 7.49 (m, 3H), 7.47 – 7.37 (m, 2H), 4.99 (dd, *J* = 13.4, 9.3 Hz, 1H), 4.70 (dd, *J* = 13.3, 6.1 Hz, 1H), 4.18 (dd, *J* = 9.3, 6.0 Hz, 1H), 2.44 (s, 3H), 1.05 (s, 21H); ^{13}C NMR (125 MHz, $CDCl_3$) δ 164.28 (C), 164.25 (C), 156.3 (C), 131.2 (CH), 128.7 (2CH), 128.4 (2CH), 126.1 (C), 103.2 (C), 100.2 (C), 87.0 (C), 74.1 (CH_2), 26.3 (CH_3), 22.9 (CH), 18.5 ($3CH_3$), 11.0 (3CH); HRMS (ESI) *m/z*: 457.2137 $[M+H]^+$, $C_{24}H_{33}N_2O_5Si^+$ requires 457.2153.

Synthesis of compound 3aa at one mmol scale

In a round bottom flask, chiral catalyst **V** (42 mg, 0.1 mmol), isoxazol-5(4*H*)-one **1a** (161 mg, 1 mmol), and nitroenyne **2a** (208 mg, 1 mmol) were introduced. Then, chloroform (10 mL) was added under a nitrogen atmosphere, and the mixture was stirred until isoxazol-5(4*H*)-one **1a** was consumed. Acetic anhydride (189 μL, 0.2 mmol) was added, and the reaction was allowed to proceed for 30 minutes at room temperature. Finally, column chromatography was performed using a mixture of Hexane:EtOAc as the eluent to obtain compound **3aa** (329 mg, 80%) with 92% ee.

Synthesis and characterization data of products 4

General procedure for the enantioselective synthesis of 4

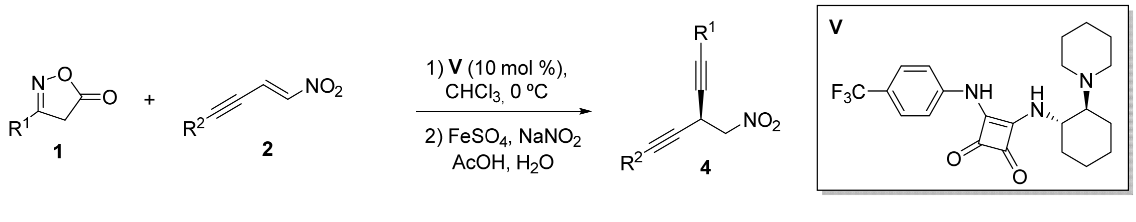

In a test tube, the catalyst **V** (4.2 mg, 0.01 mmol), isoxazol-5(4*H*)-one **1** (0.1 mmol), and nitroenyne **2** (0.1 mmol) are introduced. Chloroform (1 mL) is added under a nitrogen atmosphere, and the mixture is stirred at 0 ºC until the isoxazol-5(4*H*)-one is consumed. The reaction mixture is then concentrated in a 25 mL round-bottom flask. The

concentrated is dissolved in degassed acetic acid (1 mL) and is added via syringe to a solution prepared by adding $NaNO_2$ (69.0 mg, 1 mmol) in degassed water (1 mL) to a solution of heptahydrated iron(II) sulfate (152 mg, 0.55 mmol) in degassed acetic acid (1 mL) under nitrogen. After completion, it was neutralised with std. aqueous $NaHCO_3$ and extracted with ethyl acetate. After drying ($MgSO_4$), filtering, and concentrating, column chromatography eluting with hexane:AcOEt mixtures gave compound **4.** Racemic compounds were prepared by following a similar procedure with an achiral squaramide.

(*R*) -1-Chloro-4-(3-(nitromethyl)-5-phenylpenta-1,4-diyn-1-yl)benzene (4aa)

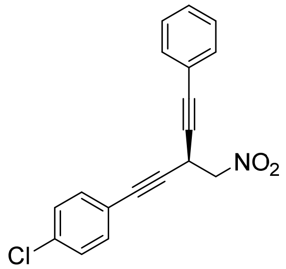

From isoxazol-5(4*H*)-one **1a** (16.1 mg, 0.1 mmol) and nitroenyne **2a** (20.7 mg, 0.1 mmol), 21.7 mg (70%) of **4aa** were obtained. The enantiomeric excess (92%) was determined by HPLC (Chiralpak AD-H), hexane:iPrOH 95:05, 1.0 mL·min^{-1}, major enantiomer: t_r = 11.3 min, minor enantiomer: t_r = 10.6 min.

Yellow oil. $[\alpha]_D^{25}$ = +2.5 (*c* = 0.43, $CHCl_3$); ^{1}H NMR (300 MHz, $CDCl_3$) δ 7.47-7.44 (m, 2H), 7.41-7.27 (m, 7H), 4.77-4.68 (m, 3H); ^{13}C NMR (75 MHz, $CDCl_3$) δ 135.2 (C), 133.3 (2CH), 132.0 (2CH), 129.1 (CH), 128.9 (2CH), 128.5 (2CH), 121.8 (C), 120.4 (C), 84.3 (C), 83.1 (C), 82.8 (C), 81.4 (C), 77.6 (CH_2), 24.3 (CH); HRMS (ESI) m/z: 310.0614 $[M+H]^+$, $C_{18}H_{13}ClNO_2^+$ requires 310.0630.

(*R*)-1-Chloro-4-(3-(nitromethyl)-5-(*p*-tolyl)penta-1,4-diyn-1-yl)benzene (4ga)

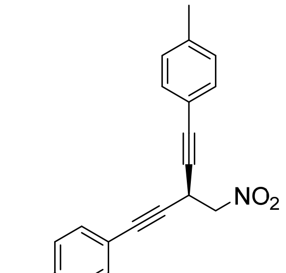

From isoxazol-5(4*H*)-one **1g** (17.5 mg, 0.1 mmol) and nitroenyne **2a** (20.7 mg, 0.1 mmol), 16.1 mg (50%) of **4ga** were obtained. The enantiomeric excess (92%) was determined by HPLC (Chiralcel OD-H), hexane:iPrOH 90:10, 1.0 mL·min^{-1}, major enantiomer: t_r = 14.2 min, minor enantiomer: t_r = 12.2 min.

Yellow oil. $[\alpha]_D^{25}$ = +7.8 (*c* = 0.15, $CHCl_3$); ^{1}H NMR (300 MHz, $CDCl_3$) δ 7.39-7.28 (m, 6H), 7.12 (d, *J* = 9 Hz, 2H), 4.76-4.67 (m, 3H), 2.35 (s, 3H); ^{13}C NMR (75 MHz, $CDCl_3$) δ 139.3 (C), 135.1 (C), 133.6 (C), 133.3 (2CH), 131.9 (2CH), 129.3 (C), 129.3 (2CH), 128.8 (2CH), 120.5 (C), 118.7 (C), 84.5 (C), 83.0 (C), 80.7 (C), 77.6 (CH_2), 77.4 (C), 24.3 (CH), 21.7 (CH_3); HRMS (ESI) m/z: 324.0772 $[M+H]^+$, $C_{19}H_{15}ClNO_2^+$ requires 324.0786.

(*R*)-1-Chloro-4-(5-(4-methoxyphenyl)-3-(nitromethyl)penta-1,4-diyn-1-yl)benzene (4ba)

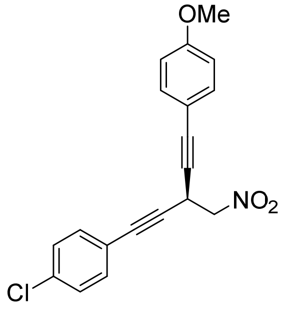

From isoxazol-5(4*H*)-one **1b** (19.1 mg, 0.1 mmol) and nitroenyne **2a** (20.7 mg, 0.1 mmol), 16.0 mg (47%) of **4ba** were obtained. The enantiomeric excess (93%) was determined by HPLC (Chiralpak AD-H), hexane:iPrOH 90:10, 1.0 mL·min^{-1}, major enantiomer: t_r = 14.3 min, minor enantiomer: t_r = 13.3 min.

Yellow oil. $[\alpha]_D^{25}$ = -16.2 (*c* = 0.12, $CHCl_3$); ^{1}H NMR (300 MHz, $CDCl_3$) δ 7.38 (dd, *J* = 9, 3 Hz, 4H), 7.29 (d, *J* = 6 Hz, 2H), 6.84 (d, *J* = 6 Hz, 2H), 4.75-4.68 (m, 3H), 3.81 (s, 3H); ^{13}C NMR (75 MHz, $CDCl_3$) δ 160.2 (C), 135.1 (C), 133.5 (2CH), 133.3 (2CH), 128.8 (2CH), 120.5 (C), 114.1 (2CH), 113.9 (C), 84.3 (C), 83.0 (C), 82.9 (C), 80.0 (C), 77.6 (CH_2), 55.5 (CH_3), 24.3 (CH); HRMS (ESI) m/z: 340.0742 $[M+H]^+$, $C_{19}H_{15}ClNO_3^+$ requires 340.0735.

(*S*)-2-(5-(4-Chlorophenyl)-3-(nitromethyl)penta-1,4-diyn-1-yl)thiophene (4da)

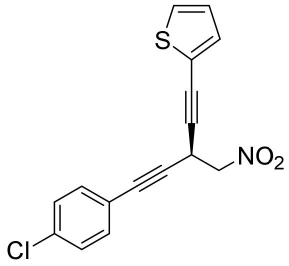

From isoxazol-5(4*H*)-one **1d** (16.7 mg, 0.1 mmol) and nitroenyne **2a** (20.7 mg, 0.1 mmol), 15.2 mg (48%) of **4da** were obtained. The enantiomeric excess (89%) was determined by HPLC (Chiralpak AD-H), hexane:iPrOH 90:10, 1.0 mL·min^{-1}, major enantiomer: t_r = 11.9 min, minor enantiomer: t_r = 11.1 min.

Yellow oil. $[\alpha]_D^{25}$ = +17.4 (*c* = 0.10, $CHCl_3$); ^{1}H NMR (300 MHz, $CDCl_3$) δ 7.39-7.24 (m, 6H), 6.98 (dd, *J* = 3.6, 5.1 Hz, 1H), 4.74 (s, 3H); ^{13}C NMR (75 MHz, $CDCl_3$) δ 135.2 (C), 133.3 (2CH), 133.2 (2CH), 128.9 (CH), 128.0 (CH), 127.2 (CH), 121.7 (C), 120.3 (C), 85.2 (C), 83.2 (C), 82.4 (C), 77.8 (C), 77.3 (CH_2), 24.5 (CH); HRMS (ESI) m/z: 316.0181 $[M+H]^+$, $C_{16}H_{11}ClNO_2S^+$ requires 316.0194.

(*R*)-1-Chloro-4-(3-(nitromethyl)hexa-1,4-diyn-1-yl)benzene (4ea)

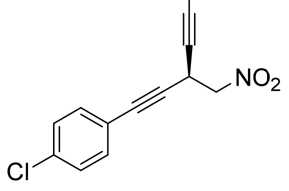

From isoxazol-5(4*H*)-one **1e** (10.0 mg, 0.1 mmol) and nitroenyne **2a** (20.7 mg, 0.1 mmol), 17.4 mg (70%) of **4ea** were obtained. The enantiomeric excess (59%) was determined by HPLC (Chiralpak IJ), hexane:iPrOH 90:10, 1.0 mL·min^{-1}, major enantiomer: t_r = 14.4 min, minor enantiomer: t_r = 16.1 min.

Yellow oil. $[\alpha]_D^{25}$ = +100 (c = 0.02, $CHCl_3$); ^{1}H NMR (500 MHz, $CDCl_3$) δ 7.36-7.34 (m, 2H), 7.29-7.27 (m, 2H), 4.62-4.60 (m, 2H), 4.45-4.41 (m, 1H), 1.84 (d, J = 2.4 Hz, 3H); ^{13}C NMR (125 MHz, $CDCl_3$) δ 134.9 (C), 133.1 (2CH), 128.7 (2CH), 120.4 (C), 83.3 (C), 82.4 (C), 80.6 (C), 77.6 (CH_2), 71.4 (C), 23.5 (CH), 3.6 (CH_3). HRMS (ESI) m/z: 248.0461 $[M+H]^+$, $C_{13}H_{11}ClNO_2^+$ requires 248.0473. The NMR spectra of compound **4ea** exhibit residual signals corresponding to the solvent used during its purification. Despite repeated attempts, these solvent traces could not be completely removed.

(*R*)-1-Chloro-3-(3-(nitromethyl)-5-phenylpenta-1,4-diyn-1-yl)benzene (4ab)

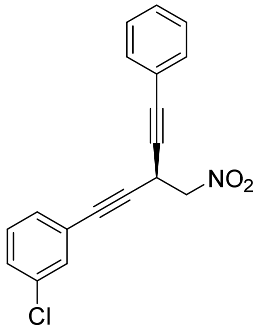

From isoxazol-5(4*H*)-one **1a** (16.1 mg, 0.1 mmol) and nitroenyne **2b** (20.7 mg, 0.1 mmol), 13.0 mg (42%) of **4ab** were obtained. The enantiomeric excess (83%) was determined by HPLC (Chiralcel OD-H), hexane:iPrOH 90:10, 1.0 mL·min^{-1}, major enantiomer: t_r = 23.6 min, minor enantiomer: t_r = 16.3 min.

Yellow oil. $[\alpha]_D^{25}$ = +21.2 (c = 0.08, $CHCl_3$); ^{1}H NMR (300 MHz, $CDCl_3$) δ 7.47-7.44 (m, 3H), 7.36-7.30 (m, 5H), 7.27-7.22 (m, 1H), 4.77-4.69 (m, 3H); ^{13}C NMR (125 MHz, $CDCl_3$) δ 134.2 (C), 131.9 (2CH), 131.8 (CH), 130.0 (CH), 129.6 (CH), 129.2 (CH), 129.0 (CH), 128.4 (2CH), 123.5 (C), 121.6 (C), 84.2 (C), 82.9 (C), 82.6 (C), 81.1 (C), 77.3 (CH_2), 24.1 (CH). HRMS (ESI) m/z: 310.0623 $[M+H]^+$, $C_{18}H_{13}ClNO_2^+$ requires 310.0630.

(*S*)-1-Methyl-4-(3-(nitromethyl)-5-phenylpenta-1,4-diyn-1-yl)benzene (4gc)

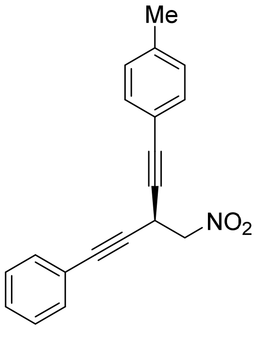

From isoxazol-5(4*H*)-one **1g** (17.5 mg, 0.1 mmol) and nitroenyne **2c** (17.3 mg, 0.1 mmol), 15.6 mg (54%) of **4gc** were obtained. The enantiomeric excess (96%) was determined by HPLC (Chiralcel OD-H), hexane:iPrOH 90:10, 1.0 mL·min^{-1}, major enantiomer: t_r = 15.5 min, minor enantiomer: t_r = 20.2 min.

Yellow oil. $[\alpha]_D^{25}$ = +39.2 (c = 0.12, $CHCl_3$); ^{1}H NMR (300 MHz, $CDCl_3$) δ 7.47-7.42 (m, 2H), 7.38-7.28 (m, 5H), 7.12 (d, J = 7.5 Hz, 2H), 4.77-4.69 (m, 3H), 2.35 (s, 3H); ^{13}C NMR (125 MHz, $CDCl_3$) δ 139.1 (C), 131.9 (2CH), 131.8 (2CH), 129.1 (2CH), 128.8 (CH), 128.3 (2CH), 121.8 (C), 118.7 (C), 84.1 (C), 83.9 (C), 81.7 (C), 80.8 (C), 77.6 (CH_2), 24.2 (CH), 21.5 (CH_3); HRMS (ESI) m/z: 290.1168 $[M+H]^+$, $C_{19}H_{16}NO_2^+$ requires 290.1176.

(*S*)-1-Bromo-4-(3-(nitromethyl)-5-phenylpenta-1,4-diyn-1-yl)benzene (4cc)

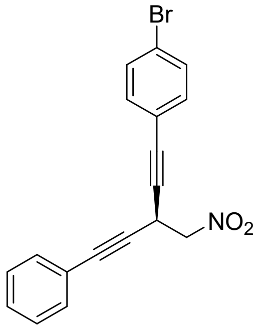

From isoxazol-5(4*H*)-one **1c** (24.0 mg, 0.1 mmol) and nitroenyne **2c** (17.3 mg, 0.1 mmol), 14.5 mg (41%) of **4cc** were obtained. The enantiomeric excess (91%) was determined by HPLC (Chiralpak AD-H), hexane:iPrOH 90:10, 1.0 mL·min^{-1}, major enantiomer: t_r = 9.1 min, minor enantiomer: t_r = 9.6 min.

Yellow oil. $[\alpha]_D^{25}$ = +24.1 (*c* = 0.17, $CHCl_3$); ^{1}H NMR (300 MHz, $CDCl_3$) δ 7.47-7.44 (m, 4H), 7.36-7.30 (m, 5H), 4.77-4.71 (m, 3H); ^{13}C NMR (125 MHz, $CDCl_3$) δ 131.3 (2CH), 131.9 (2CH), 131.6 (2CH), 129.0 (CH), 128.3 (2CH), 123.3 (C), 121.7 (C), 120.7 (C), 84.2 (C), 83.0 (C), 82.8 (C), 81.2 (C), 77.3 (CH_2), 24.1 (CH); HRMS (ESI) m/z: 354.0113 [M+H]$^+$, $C_{18}H_{13}BrNO_2^+$ requires 354.0124.

(*R*)-1-Methoxy-4-(3-(nitromethyl)-5-phenylpenta-1,4-diyn-1-yl)benzene (4ad)

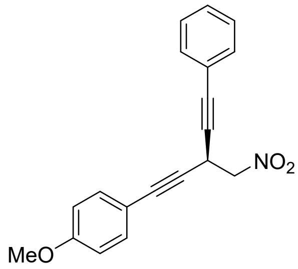

From isoxazol-5(4*H*)-one **1a** (16.1 mg, 0.1 mmol) and nitroenyne **2d** (20.3 mg, 0.1 mmol), 18.6 mg (61%) of **4ad** were obtained. The enantiomeric excess (89%) was determined by HPLC (Chiralcel OD-H) hexane:^{i}PrOH 90:10, 1.0 mL min^{-1}, major enantiomer t_r = 26.2 min, minor enantiomer t_r = 21.1 min.

Yellow oil. $[\alpha]_D^{25}$ = +6.2 (*c* = 0.27, $CHCl_3$); ^{1}H NMR (300 MHz, $CDCl_3$) δ 7.47-7.43 (m, 2H), 7.39 (d, *J* = 8.8 Hz, 2H), 7.35-7.29 (m, 3H), 6.84 (d, *J* = 8.8 Hz, 2H), 4.76-4.69 (m, 3H), 3.81 (s, 3H); ^{13}C NMR (75 MHz, $CDCl_3$) δ 160.0 (C), 133.4 (2CH), 131.9 (2CH), 128.8 (2CH), 128.3 (2CH), 121.9 (C), 113.9 (CH), 113.8 (C), 83.9 (C), 83.8 (C), 81.8 (C), 81.1 (C), 77.6 (CH_2), 55.3 (CH_3), 24.2 (CH). HRMS (ESI) m/z: 306.1131 [M+H]$^+$, $C_{19}H_{16}NO_3^+$ requires 306.1125.

(*R*)-1-Chloro-4-(3-(nitromethyl)-5-(*p*-tolyl)penta-1,4-diyn-1-yl)benzene (4gd)

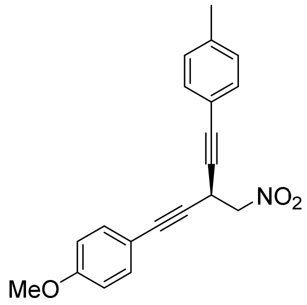

From isoxazol-5(4*H*)-one **1g** (17.5 mg, 0.1 mmol) and nitroenyne **2d** (20.3 mg, 0.1 mmol), 15.2 mg (50%) of **4gd** were obtained. The enantiomeric excess (87%) was determined by HPLC (Chiralpak AS-H), hexane:iPrOH 90:10, 1.0 mL·min^{-1}, major enantiomer: t_r = 16.0 min, minor enantiomer: t_r = 12.0 min.

Yellow oil. $[\alpha]_D^{25}$ = +62.8 (c = 0.07, $CHCl_3$); ^1H NMR (500 MHz, $CDCl_3$) δ 7.40-7.32 (m, 4H), 7.13-7.10 (m, 2H), 6.85-6.82 (m, 2H), 4.75-4.70 (m, 3H), 3.81 (s, 3H), 2.35 (s, 3H); ^{13}C NMR (125 MHz, $CDCl_3$) δ 160.0 (C), 139.0 (C), 133.4 (2CH), 131.8 (2CH), 129.1 (2CH), 118.8 (C), 113.92 (2CH), 113.88 (C), 84.0 (C), 83.9 (C), 81.1 (C), 80.3 (C), 77.7 (CH_2), 55.3 (CH_3), 24.2 (CH), 21.5 (CH_3); HRMS (ESI) m/z: 320.1271 $[M+H]^+$, $C_{20}H_{18}NO_3^+$ requires 320.1281.

(*S*)-1-Chloro-4-(5-(4-methoxyphenyl)-3-(nitromethyl)penta-1,4-diyn-1-yl)benzene (4fd)

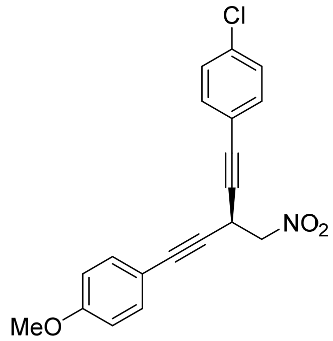

From isoxazol-5(4*H*)-one **1f** (19.6 mg, 0.1 mmol) and nitroenyne **2d** (20.3 mg, 0.1 mmol), 19.4 mg (57%) of **4fd** were obtained. The enantiomeric excess (87%) was determined by HPLC (Chiralpak AD-H), hexane:iPrOH 90:10, 1.0 mL·min^{-1}, major enantiomer: t_r = 13.4 min, minor enantiomer: t_r = 14.6 min.

Yellow oil. $[\alpha]_D^{25}$ = +5.6 (c = 0.09, $CHCl_3$); ^1H NMR (300 MHz, $CDCl_3$) δ 7.40-7.26 (m, 6H), 6.84 (d, J = 8.9 Hz, 2H), 4.75-4.70 (m, 3H), 3.81 (s, 3H). Data according with compound **4ba**.

(*R*)-1-Methoxy-3-(3-(nitromethyl)-5-phenylpenta-1,4-diyn-1-yl)benzene (4ae)

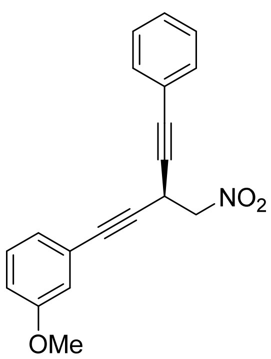

From isoxazol-5(4*H*)-one **1a** (16.1 mg, 0.1 mmol) and nitroenyne **2e** (20.3 mg, 0.1 mmol), 14.6 mg (48%) of **4ae** were obtained. The enantiomeric excess (88%) was determined by HPLC (Chiralcel OD-H), hexane:iPrOH 90:10, 1.0 mL·min^{-1}, major enantiomer: t_r = 27.6 min, minor enantiomer: t_r = 37.0 min.

Yellow oil. $[\alpha]_D^{25}$ = -10.8 (c = 0.12, $CHCl_3$); ^1H NMR (300 MHz, $CDCl_3$) δ 7.47-7.43 (m, 2H), 7.36-7.30 (m, 3H), 7.25-7.20 (m, 1H), 7.06-6.88 (m, 3H), 4.78-4.72 (m, 3H), 3.80 (s, 3H); ^{13}C NMR (125 MHz, $CDCl_3$) δ 159.3 (C), 131.9 (2CH), 129.4 (CH), 128.9 (CH), 128.3 (2CH), 124.4 (CH), 122.8 (C), 121.8 (C), 116.7 (CH), 115.6 (CH), 84.0 (C), 83.9 (C), 81.5 (C), 81.3 (C), 77.5 (CH_2), 55.3 (CH_3), 24.1 (CH); HRMS (ESI) m/z: 306.1122 $[M+H]^+$, $C_{19}H_{16}NO_3^+$ requires 306.1125.

(*S*)-1-Methoxy-4-(3-(nitromethyl)-7-phenylhepta-1,4-diyn-1-yl)benzene (4bf)

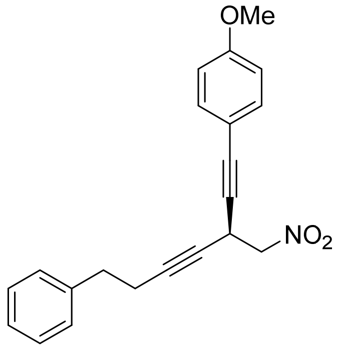

From isoxazol-5(4*H*)-one **1b** (19.1 mg, 0.1 mmol) and nitroenyne **2f** (20.1 mg, 0.1 mmol), 13.3 mg (40%) of **4bf** were obtained. The enantiomeric excess (91%) was determined by HPLC (Chiralpak AS-H), hexane:iPrOH 90:10, 1.0 mL·min^{-1}, major enantiomer: t_r = 11.0 min, minor enantiomer: t_r = 16.5 min.

Yellow oil. $[\alpha]_D^{25}$ = +5.8 (*c* = 0.12 mg, $CHCl_3$); ^{1}H NMR (500 MHz, $CDCl_3$) δ 7.37-7.34 (m, 2H), 7.30-7.27 (m, 2H), 7.24-7.19 (m, 3H), 6.85-6.82 (m, 2H), 4.60-4.55 (m, 2H), 4.46-4.40 (m, 1H), 3.81 (s, 3H), 2.82 (t, *J* = 7.5 Hz, 2H), 2.49 (td, *J* = 7.4, 2.2 Hz, 2H); ^{13}C NMR (75 MHz, $CDCl_3$) δ 159.9 (C), 140.3 (C), 133.3 (2CH), 128.5 (2CH), 128.4 (2CH), 126.4 (CH), 114.0 (C), 113.9 (2CH), 83.8 (C), 83.5 (C), 80.7 (C), 77.9 (CH_2), 73.7 (C), 55.3 (CH_3), 34.7 (CH_2), 23.6 (CH), 20.9 (CH_2); HRMS (ESI) m/z: 334.1422 $[M+H]^+$, $C_{21}H_{20}NO_3^+$ requires 334.1438.

(*R*)-Triisopropyl(3-(nitromethyl)-5-phenylpenta-1,4-diyn-1-yl)silane (4ah)

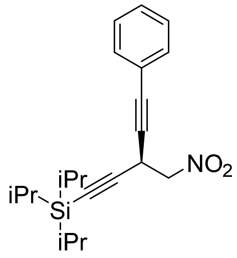

From isoxazol-5(4*H*)-one **1a** (16.1 mg, 0.1 mmol), nitroenyne **2h** (25.3 mg, 0.1 mmol), 18.8 mg (53%) of **4ah** were obtained. The enantiomeric excess (93%) was determined by HPLC (Chiralcel OD-H), hexane:iPrOH 90:10, 1.0 mL·min^{-1}, major enantiomer: t_r = 11.5 min, minor enantiomer: t_r = 6.2 min.

Yellow oil. $[\alpha]_D^{25}$ = +7.9 (*c* = 0.19, $CHCl_3$). ^{1}H NMR (300 MHz, $CDCl_3$) δ 7.43-7.40 (m, 2H), 7.34-7.30 (m, 3H), 4.68-4.64 (m, 2H), 4.53 (dd, *J* = 3.6, 5.1 Hz, 1H), 1.07 (s, 21H). ^{13}C NMR (75 MHz, $CDCl_3$) δ 131.8 (2CH), 128.8 (CH), 128.3 (2CH), 121.9 (C), 99.3 (C), 86.0 (C), 83.9 (C), 81.8 (C), 77.6 (C), 24.5 (CH), 18.5 (CH_3), 11.0 (CH). HRMS (ESI) m/z: 373.2290 $[M+NH_4]^+$, $C_{21}H_{33}N_2O_2Si^+$ requires 373.2306.

Synthesis of compound 4aa from 3aa

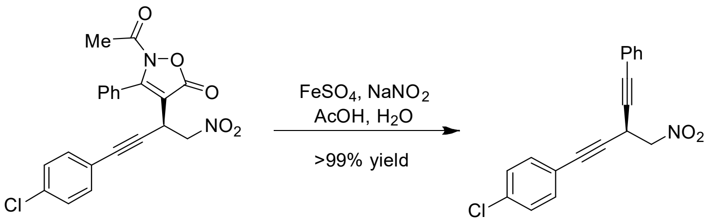

3aa
92% ee

4aa
91% ee

Compound **3aa** (23.4 mg, 0.057 mmol) was introduced in a round-bottom flask under a nitrogen atmosphere and dissolved in degassed acetic acid (0.6 mL). Simultaneously, a solution of $NaNO_2$ (39.3 mg, 0.57 mmol) in degassed water (0.6 mL) and a solution of heptahydrated iron(II) sulfate (88.9 mg, 0.32 mmol) in degassed acetic acid (0.6 mL) were prepared and kept under nitrogen atmosphere. Then, the $NaNO_2$ and **3aa** solutions were added, in that order, to the iron(II) sulfate. When the reaction was complete, it was neutralized with std. aqueous $NaHCO_3$ and extracted with ethyl acetate. After drying with $MgSO_4$, filtering, and concentrating, column chromatography eluting with hexane:AcOEt mixtures gave 17.6 mg (99%) and 92% ee of diyne **4aa**.

Synthesis and characterization data for compound 5

(*R*)-5-(4-Chlorophenyl)-3-(nitromethyl)-1-phenylpent-4-yn-1-one (5)[3]

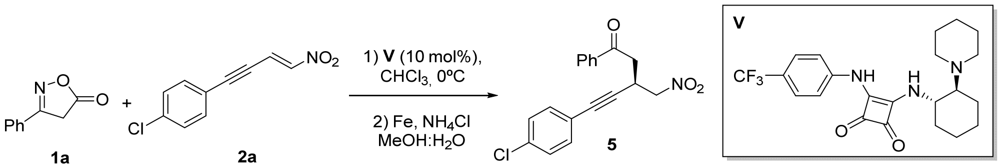

1a **2a** **5**

In a test tube, isoxazol-5(4*H*)-one **1a** (16.1 mg, 0.1 mmol), nitroenyne **2a** (20.7 mg, 0.1 mmol) and catalyst **V** (4.2 mg, 0.01 mmol) were introduced. The flask was purged with nitrogen and chloroform (1 mL) was added at 0 ºC. Once the starting materials were consumed, the reaction mixture was concentrated under reduced pressure. Then, the mixture was dissolved in 1 mL of H_2O:MeOH (1:1), iron (55.9 mg, 1 mmol) and NH_4Cl (53.5 mg, 1 mmol) were added, and the mixture was stirred for 24 hours. After this time, the reaction mixture was filtered through celite, washed with MeOH and dichloromethane. The filtrate was concentrated under reduced pressure and purified by column chromatography using Hexane:EtOAc eluent mixtures to give 11.5 mg (35%) of ketone **5**. The enantiomeric excess (81%) was determined by HPLC (Phenomenex Lux® 5μm *i*-Amylose-1), hexane:^{i}PrOH 90:10, 1.0 mL min^{-1}, major enantiomer: t_r = 17.5 min,

minor enantiomer: t_r = 15.5 min. Yellow solid; m.p. = 88.2-90.2 ºC; $[\alpha]_D^{25}$ = +30.2 (*c* = 0.10, CH_2Cl_2), Lit.[3] $[\alpha]_{20}^{D}$ = +19.2 (*c* = 0.4, CH_2Cl_2) for the *R* enantiomer; ^1H NMR (300 MHz, $CDCl_3$) δ 8.04-7.95 (m, 1H), 7.66-7.58 (m, 1H), 7.55-7.46 (m, 4H), 7.33-7.21 (m, 4H), 4.77 (dd, *J* = 12.4, 5.8 Hz, 1H), 4.66 (dd, *J* = 12.4, 6.7 Hz, 1H), 4.13-4.09 (m, 1H), 3.48 (d, *J* = 6.7 Hz, 2H).

Mechanism of the Zard reaction (nitrosative cleavage of isoxazolinones) and role of $FeSO_4$

The mechanism of this transformation[4] is proposed to start by the in situ generation of NO^+ from the mixture of $NaNO_2$ and AcOH, which is subsequently trapped by the isoxazolinone. Nitrosation of the isoxazolinone can take place at the N or C atoms giving intermediates A or B, respectively. Intermediate A can then undergo N-O bond cleavage to give zwitterionic intermediate C, which after loss of carbon dioxide (CO_2) and nitrous oxide (N_2O) would provide the desired diyne. Intermediate B, which is formed majorly, is not productive but interconversion between intermediates B and A can take place via a radical mechanism involving homolytic cleavage of the N-O bond yielding isoxazolinone radical D and nitric oxide. At this point, a potential competing pathway is the dimerization of the radical to give a dimeric by-product. To avoid this, it is important to keep the concentration of nitric oxide high, which is achieved by the reaction of sodium nitrite and ferrous sulfate ($FeSO_4$). This excess of nitric oxide displaces the equilibrium to generate more of the N-nitroso isoxazolinone intermediate A leading to the formation of more diyne

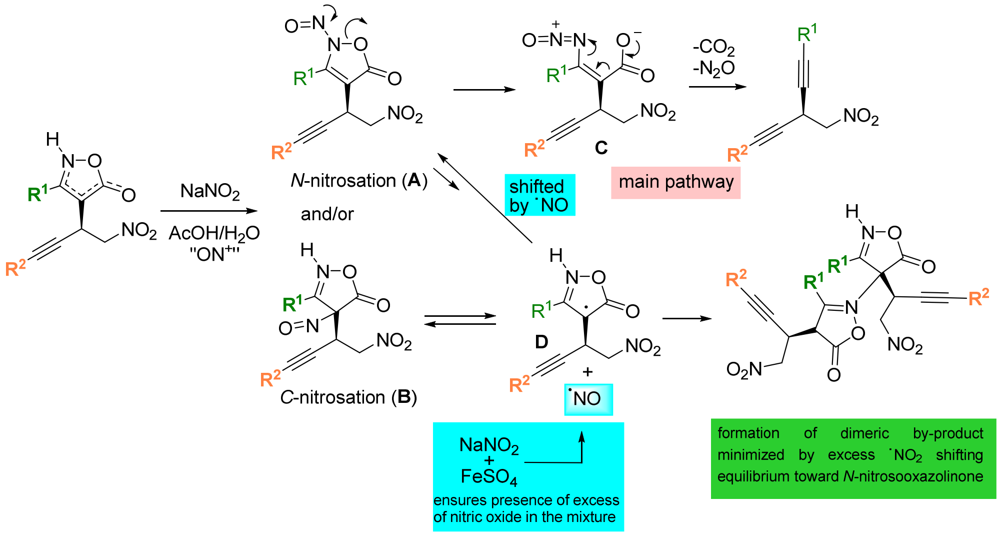

NMR SPECTRA

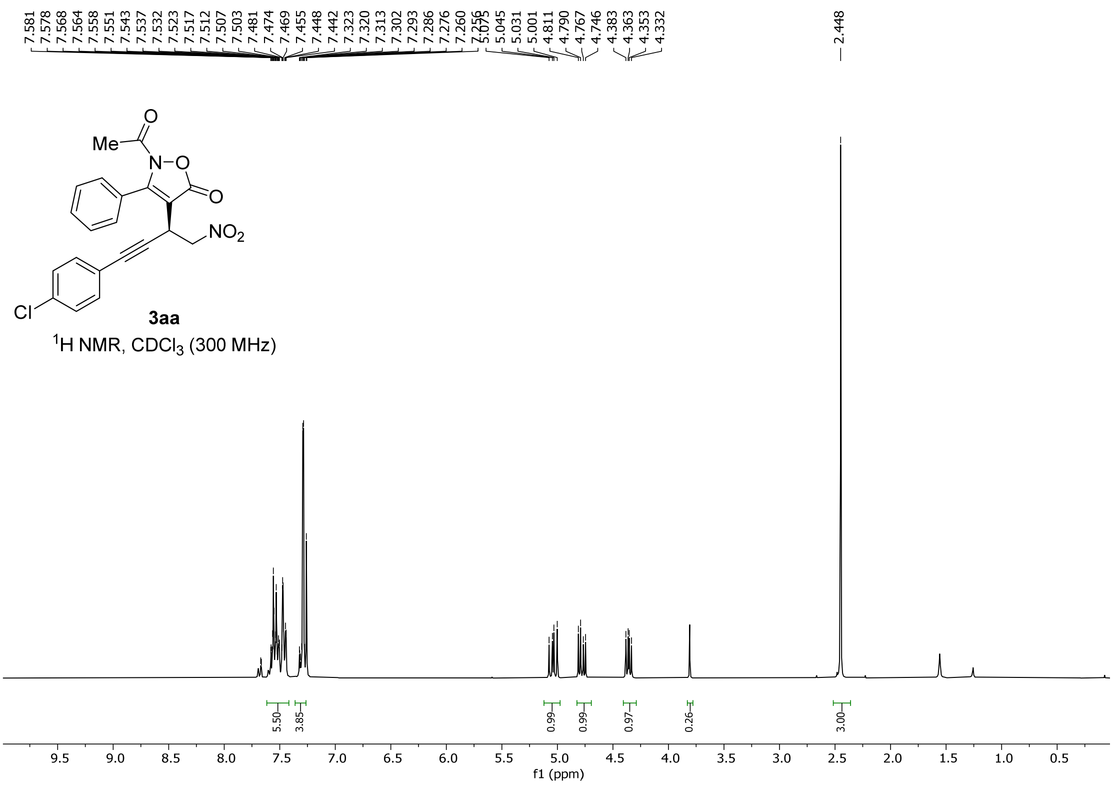

7.581
7.578
7.568
7.564
7.558
7.551
7.543
7.537
7.532
7.523
7.517
7.512
7.507
7.503
7.481
7.474
7.469
7.455
7.448
7.442
7.323
7.320
7.313
7.302
7.293
7.286
7.276
7.260
5.045
5.031
5.001
4.811
4.790
4.767
4.746
4.383
4.363
4.353
4.332
2.448
Me
N–O
O
NO2
Cl
3aa
1H NMR, CDCl3 (300 MHz)
5.50
3.85
0.99
0.99
0.97
0.26
3.00
f1 (ppm)

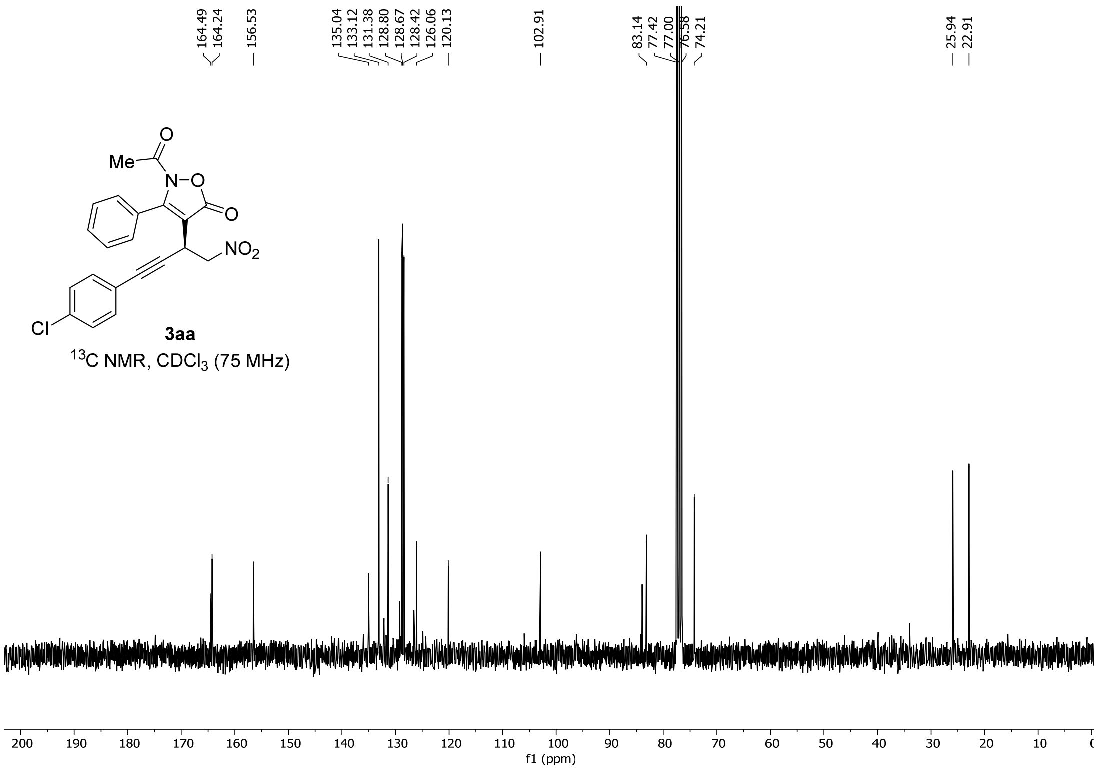

164.49
164.24
156.53
135.04
133.12
131.38
128.80
128.67
128.42
126.06
120.13
102.91
83.14
77.42
77.00
76.58
74.21
25.94
22.91
Me
N–O
O
NO2
Cl
3aa
13C NMR, CDCl3 (75 MHz)
f1 (ppm)

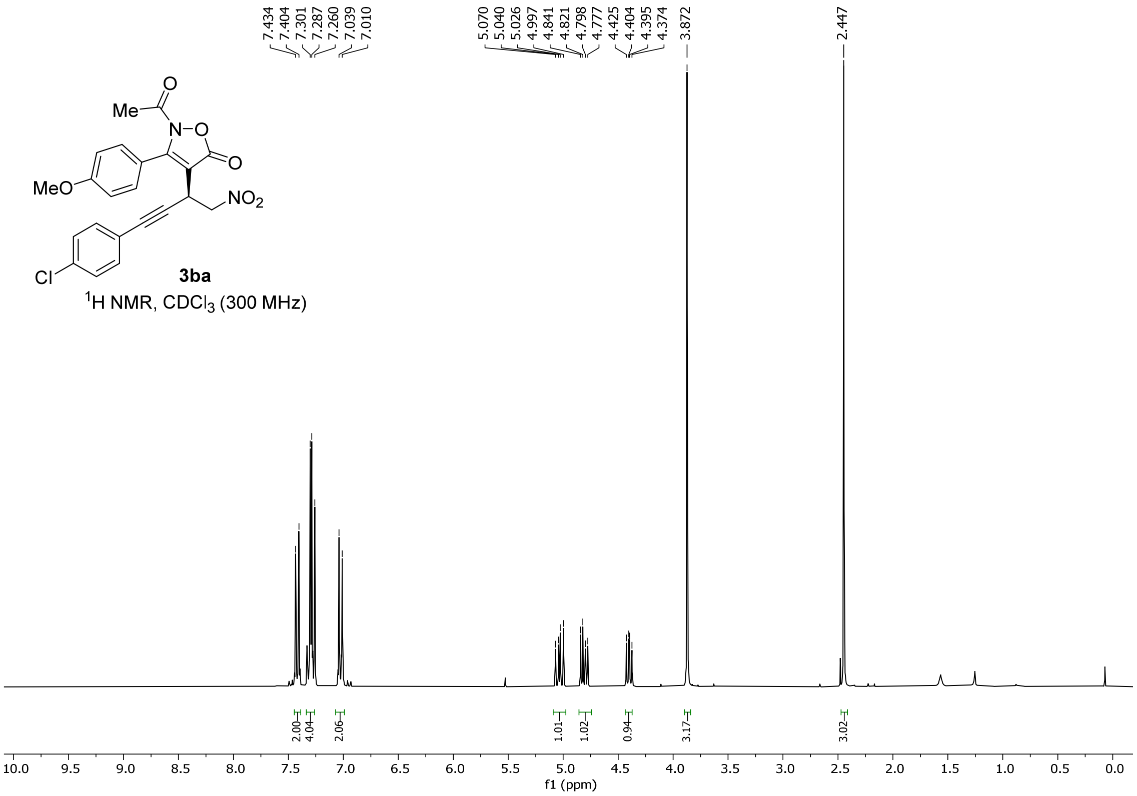
7.434
7.404
7.301
7.287
7.260
7.039
7.010
5.070
5.040
5.026
4.997
4.841
4.821
4.798
4.777
4.425
4.404
4.395
4.374
3.872
2.447
Me
N
O
O
MeO
NO2
Cl
3ba
1H NMR, CDCl3 (300 MHz)
2.00
4.04
2.06
1.01
1.02
0.94
3.17
3.02
f1 (ppm)

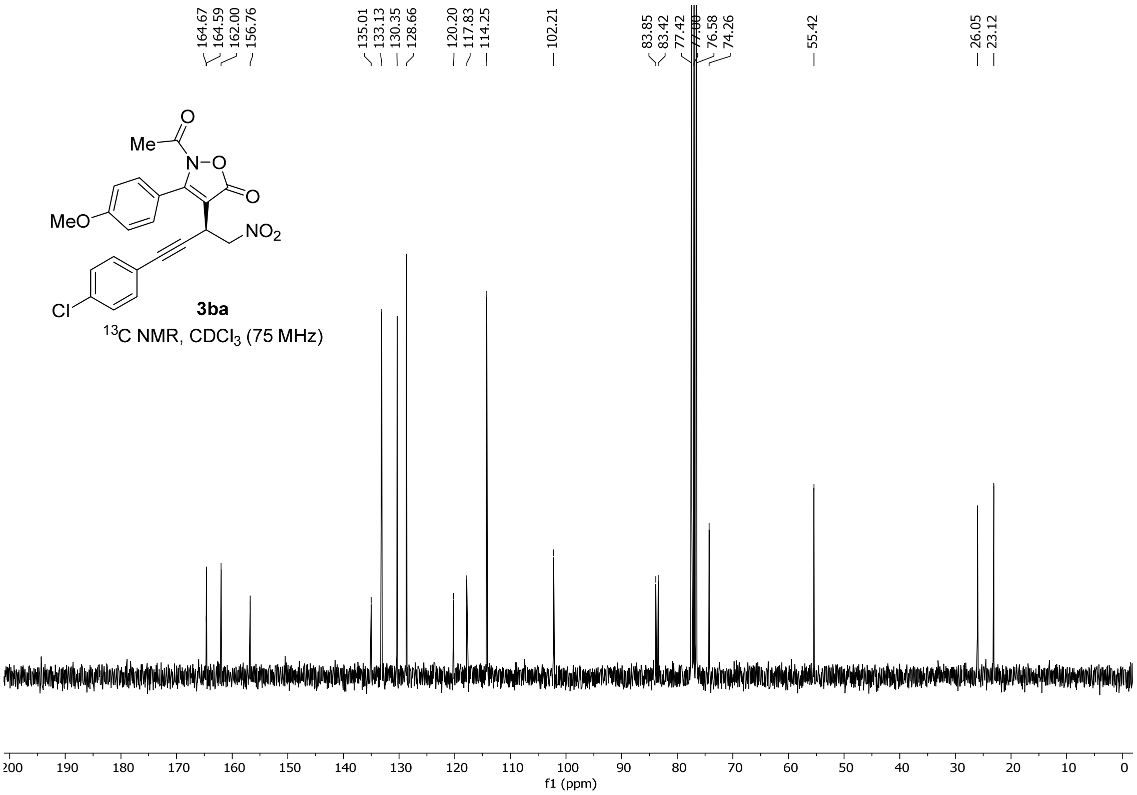
164.67
164.59
162.00
156.76
135.01
133.13
130.35
128.66
120.20
117.83
114.25
102.21
83.85
83.42
77.42
77.00
76.58
74.26
55.42
26.05
23.12
Me
N
O
O
MeO
NO2
Cl
3ba
13C NMR, CDCl3 (75 MHz)
f1 (ppm)

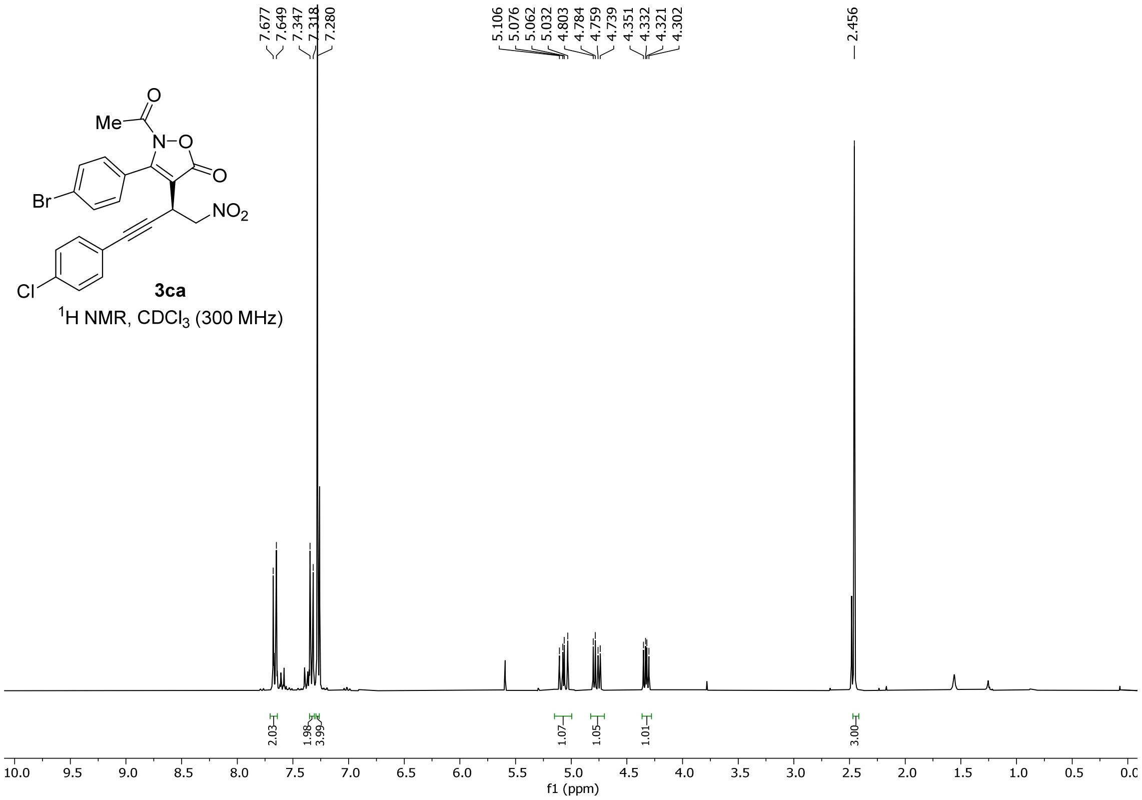
7.677
7.649
7.347
7.318
7.280
5.106
5.076
5.062
5.032
4.803
4.784
4.759
4.739
4.351
4.332
4.321
4.302
2.456
Me
N–O
O
O
Br
NO2
Cl
3ca
1H NMR, CDCl3 (300 MHz)
2.03
1.98
3.99
1.07
1.05
1.01
3.00
f1 (ppm)

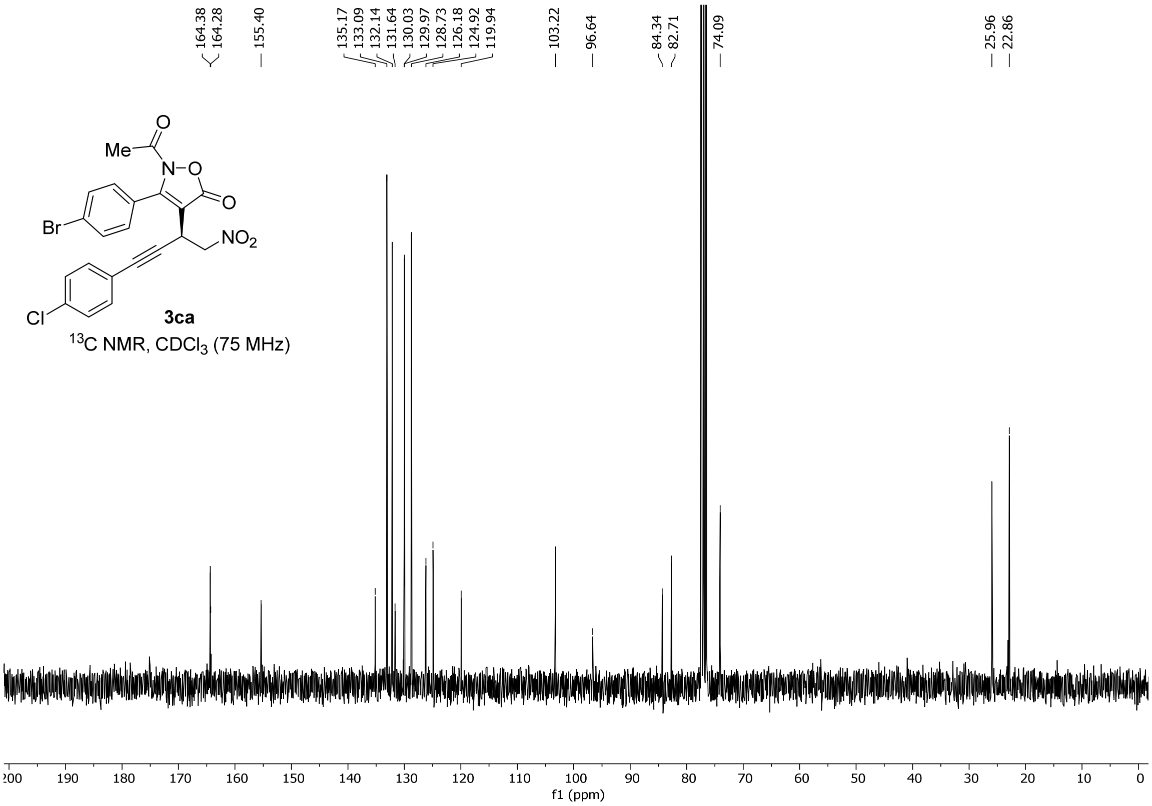
164.38
164.28
155.40
135.17
133.09
132.14
131.64
130.03
129.97
128.73
126.18
124.92
119.94
103.22
96.64
84.34
82.71
74.09
25.96
22.86
Me
N–O
O
O
Br
NO2
Cl
3ca
13C NMR, CDCl3 (75 MHz)
f1 (ppm)

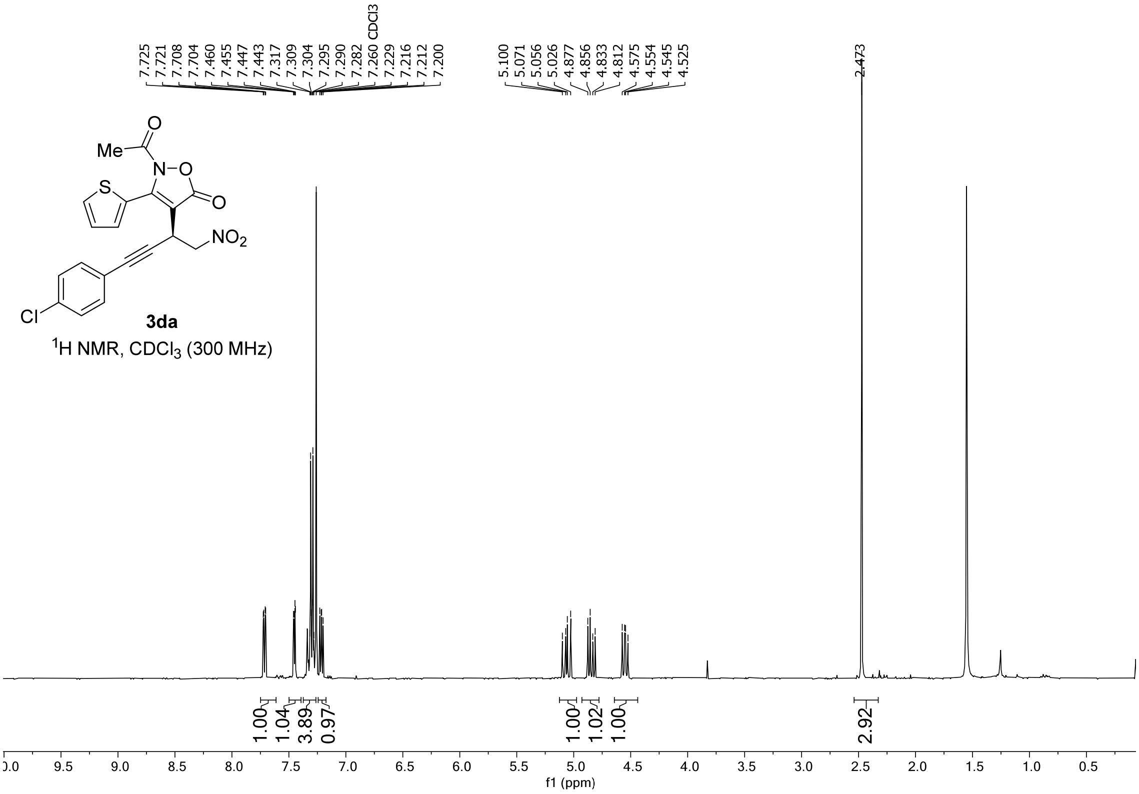
7.725 7.721 7.708 7.704 7.460 7.455 7.447 7.443 7.317 7.309 7.304 7.295 7.290 7.282 7.260 CDCl3 7.229 7.216 7.212 7.200
5.100 5.071 5.056 5.026 4.877 4.856 4.833 4.812 4.575 4.554 4.545 4.525
2.473
Me
N
O
S
NO2
Cl
3da
1H NMR, CDCl3 (300 MHz)
1.00 1.04 3.89 0.97
1.00 1.02 1.00
2.92
f1 (ppm)

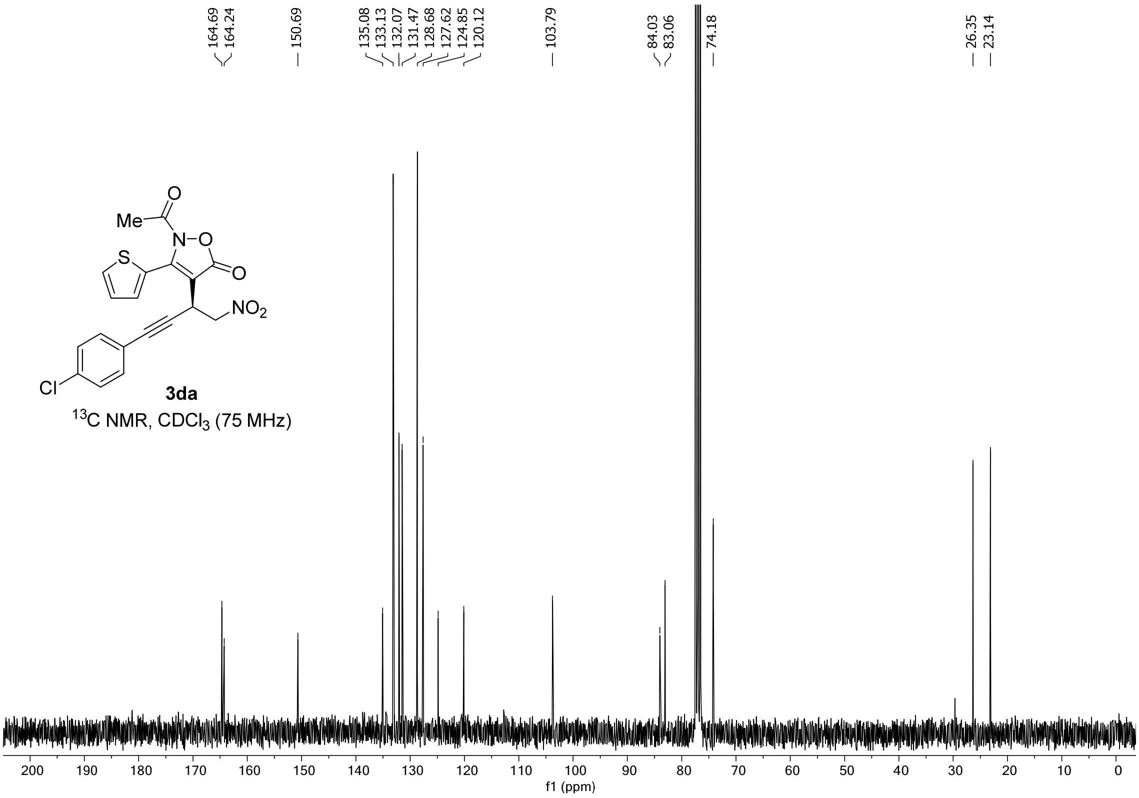
164.69 164.24
150.69
135.08 133.13 132.07 131.47 128.68 127.62 124.85 120.12
103.79
84.03 83.06
74.18
26.35 23.14
Me
N
O
S
NO2
Cl
3da
13C NMR, CDCl3 (75 MHz)
f1 (ppm)

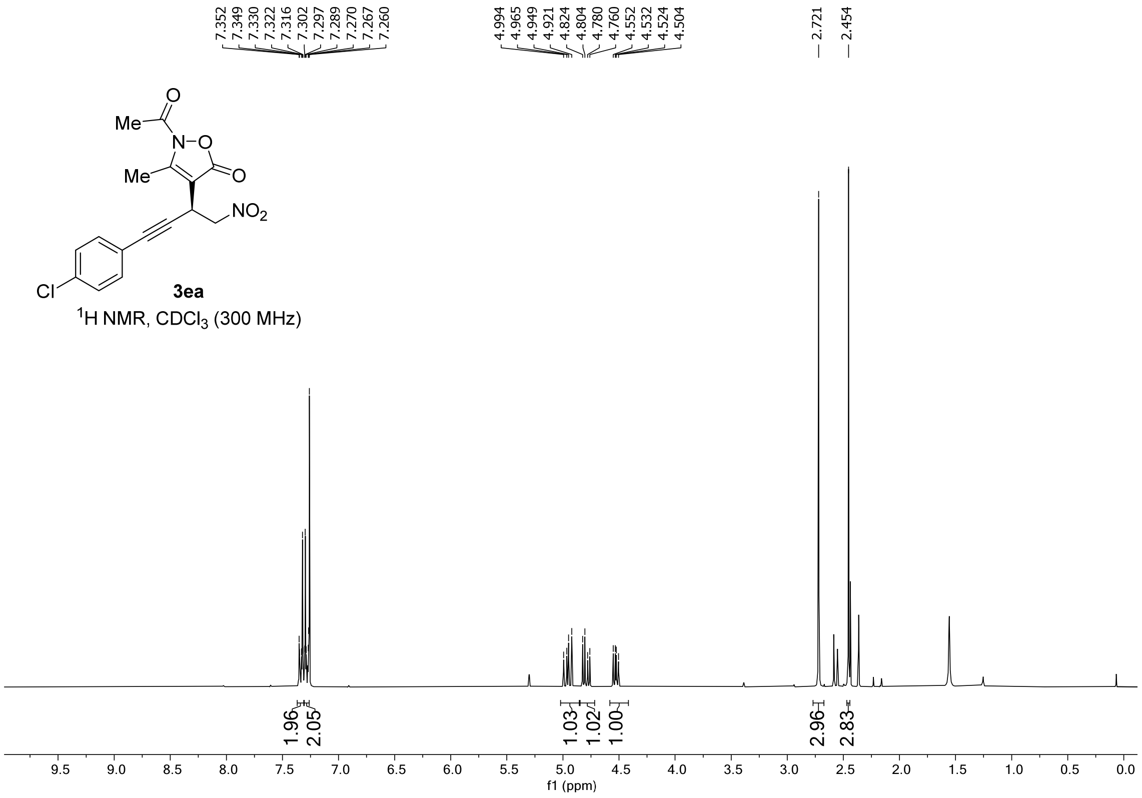
7.352
7.349
7.330
7.322
7.316
7.302
7.297
7.289
7.270
7.267
7.260
4.994
4.965
4.949
4.921
4.824
4.804
4.780
4.760
4.552
4.532
4.524
4.504
2.721
2.454
Me
N–O
O
Me
O
NO2
Cl
3ea
1H NMR, CDCl3 (300 MHz)
1.96
2.05
1.03
1.02
1.00
2.96
2.83
f1 (ppm)

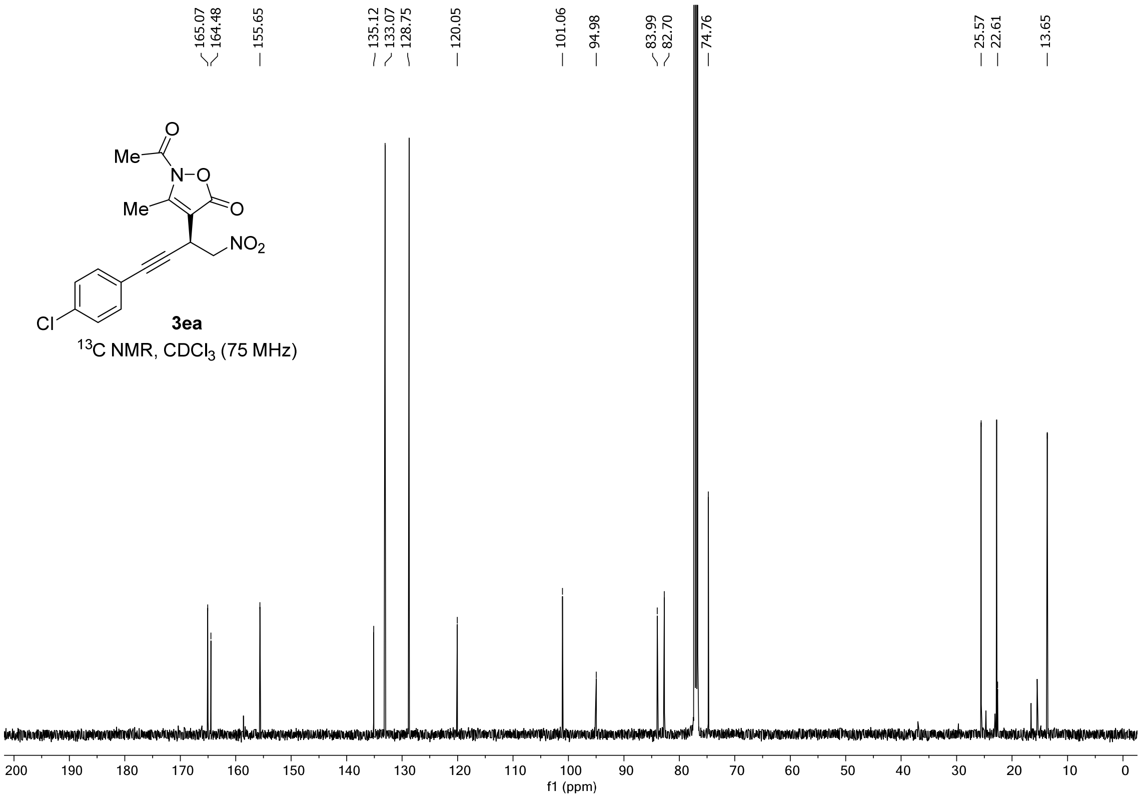
165.07
164.48
155.65
135.12
133.07
128.75
120.05
101.06
94.98
83.99
82.70
74.76
25.57
22.61
13.65
Me
N–O
O
Me
O
NO2
Cl
3ea
13C NMR, CDCl3 (75 MHz)
f1 (ppm)

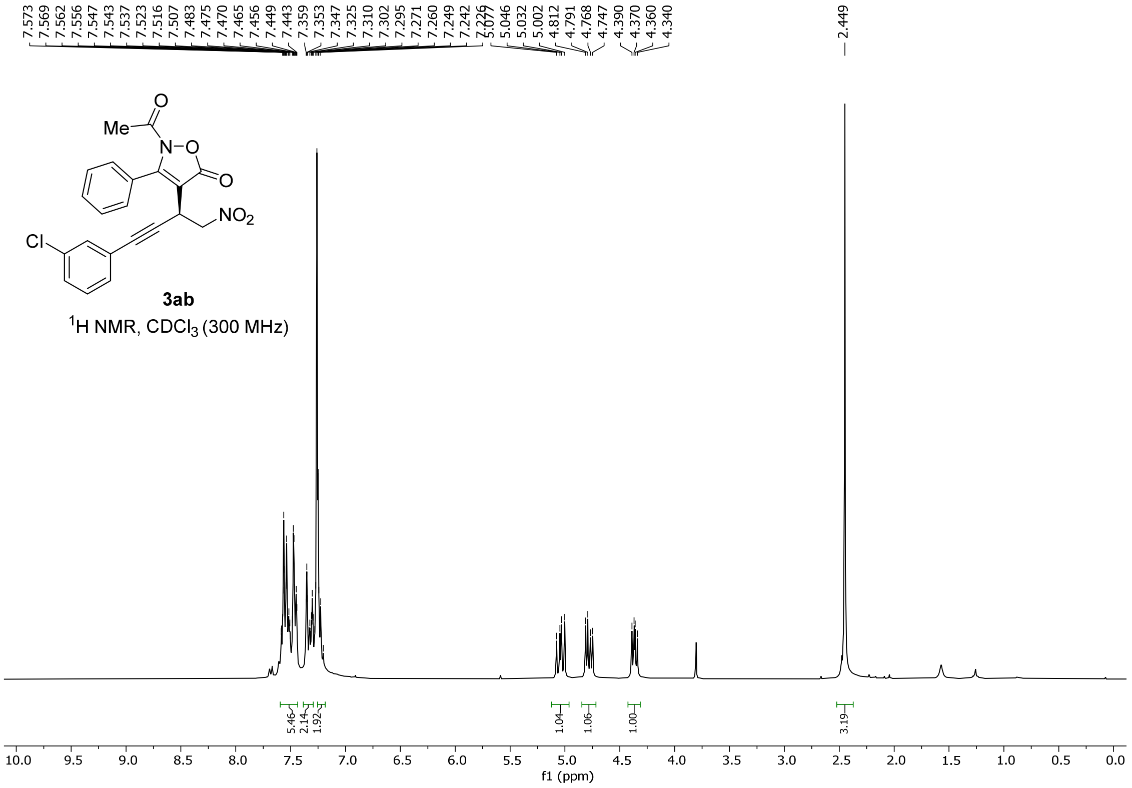
7.573 7.569 7.562 7.556 7.547 7.543 7.537 7.523 7.516 7.507 7.483 7.475 7.470 7.465 7.456 7.449 7.443 7.359 7.353 7.347 7.325 7.310 7.302 7.295 7.271 7.260 7.249 7.242 7.226 5.077 5.046 5.032 5.002 4.812 4.791 4.768 4.747 4.390 4.370 4.360 4.340
2.449
Me
N–O
O
NO2
Cl
3ab
1H NMR, CDCl3 (300 MHz)
5.46 2.14 1.92 1.04 1.06 1.00 3.19
10.0 9.5 9.0 8.5 8.0 7.5 7.0 6.5 6.0 5.5 5.0 4.5 4.0 3.5 3.0 2.5 2.0 1.5 1.0 0.5 0.0
f1 (ppm)

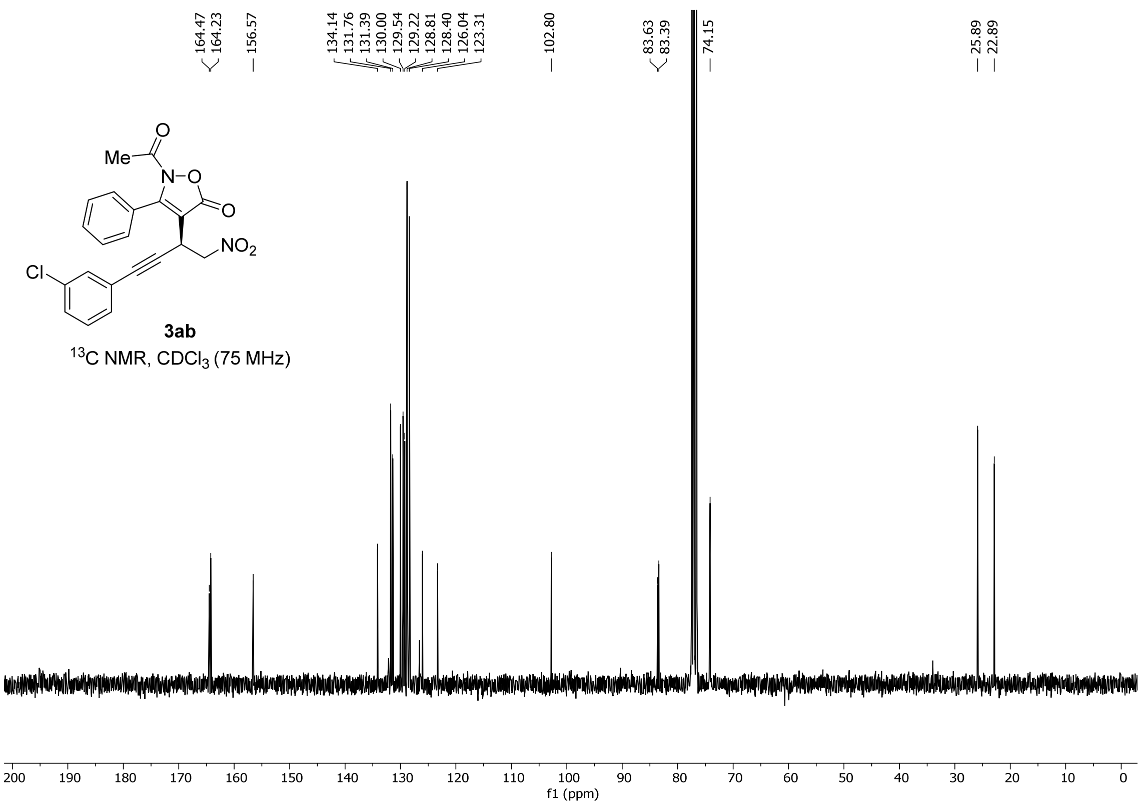
164.47 164.23 156.57
134.14 131.76 131.39 130.00 129.54 129.22 128.81 128.40 126.04 123.31
102.80
83.63 83.39 74.15
25.89 22.89
Me
N–O
O
NO2
Cl
3ab
13C NMR, CDCl3 (75 MHz)
200 190 180 170 160 150 140 130 120 110 100 90 80 70 60 50 40 30 20 10 0
f1 (ppm)

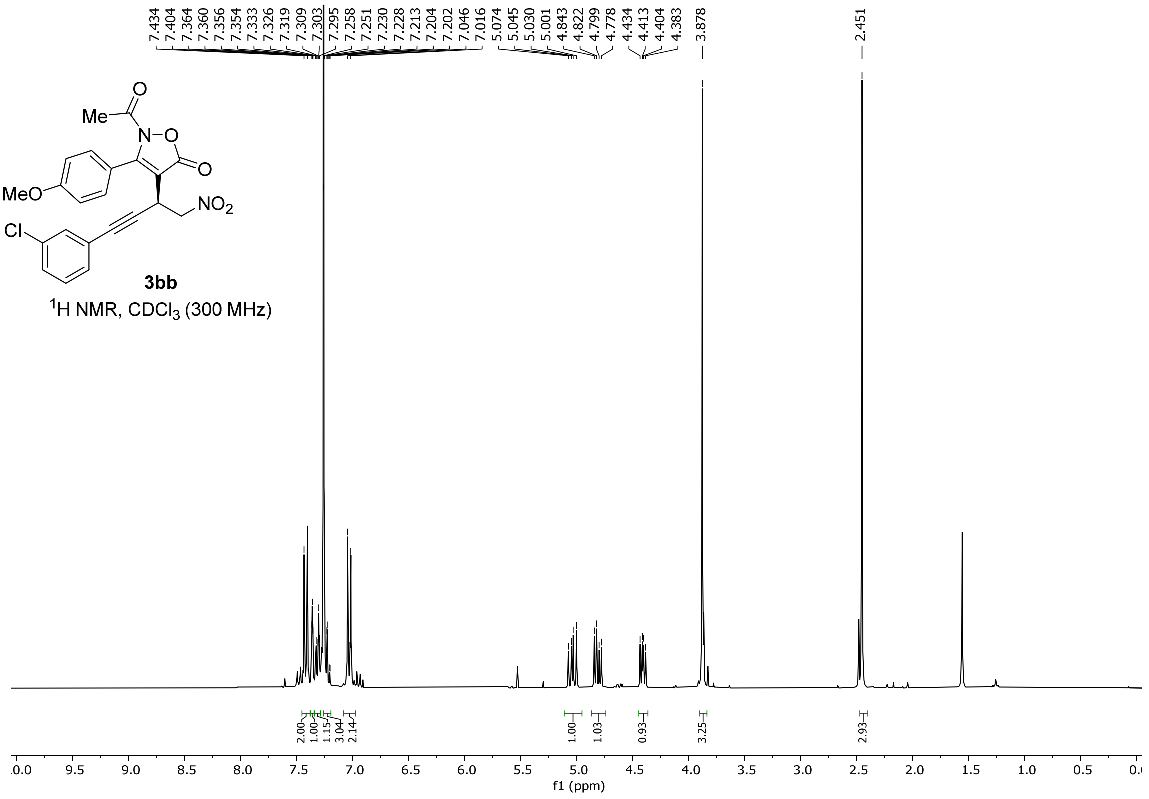
7.434
7.404
7.364
7.360
7.356
7.354
7.333
7.326
7.319
7.309
7.303
7.295
7.258
7.251
7.230
7.228
7.213
7.204
7.202
7.046
7.016
5.074
5.045
5.030
5.001
4.843
4.822
4.799
4.778
4.434
4.413
4.404
4.383
3.878
2.451
Me
N
O
O
MeO
NO2
Cl
3bb
1H NMR, CDCl3 (300 MHz)
2.00
1.00
1.15
3.04
2.14
1.00
1.03
0.93
3.25
2.93
f1 (ppm)

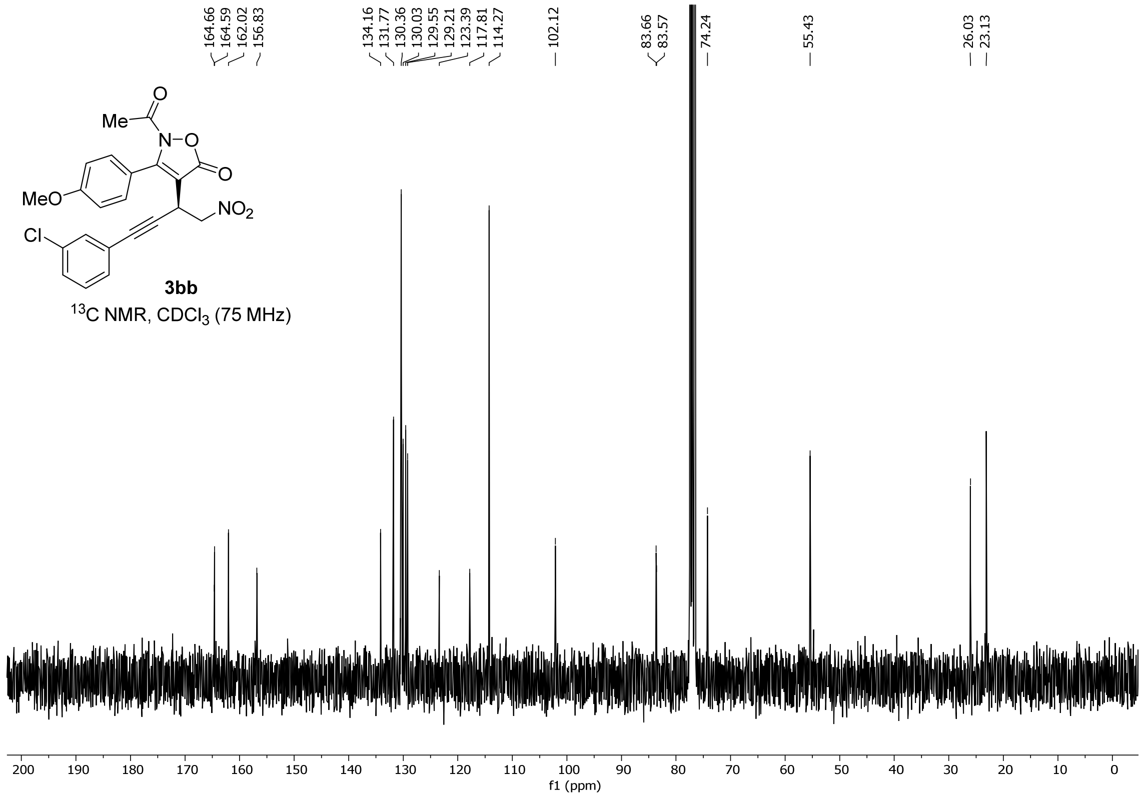
164.66
164.59
162.02
156.83
134.16
131.77
130.36
130.03
129.55
129.21
123.39
117.81
114.27
102.12
83.66
83.57
74.24
55.43
26.03
23.13
Me
N
O
O
MeO
NO2
Cl
3bb
13C NMR, CDCl3 (75 MHz)
f1 (ppm)

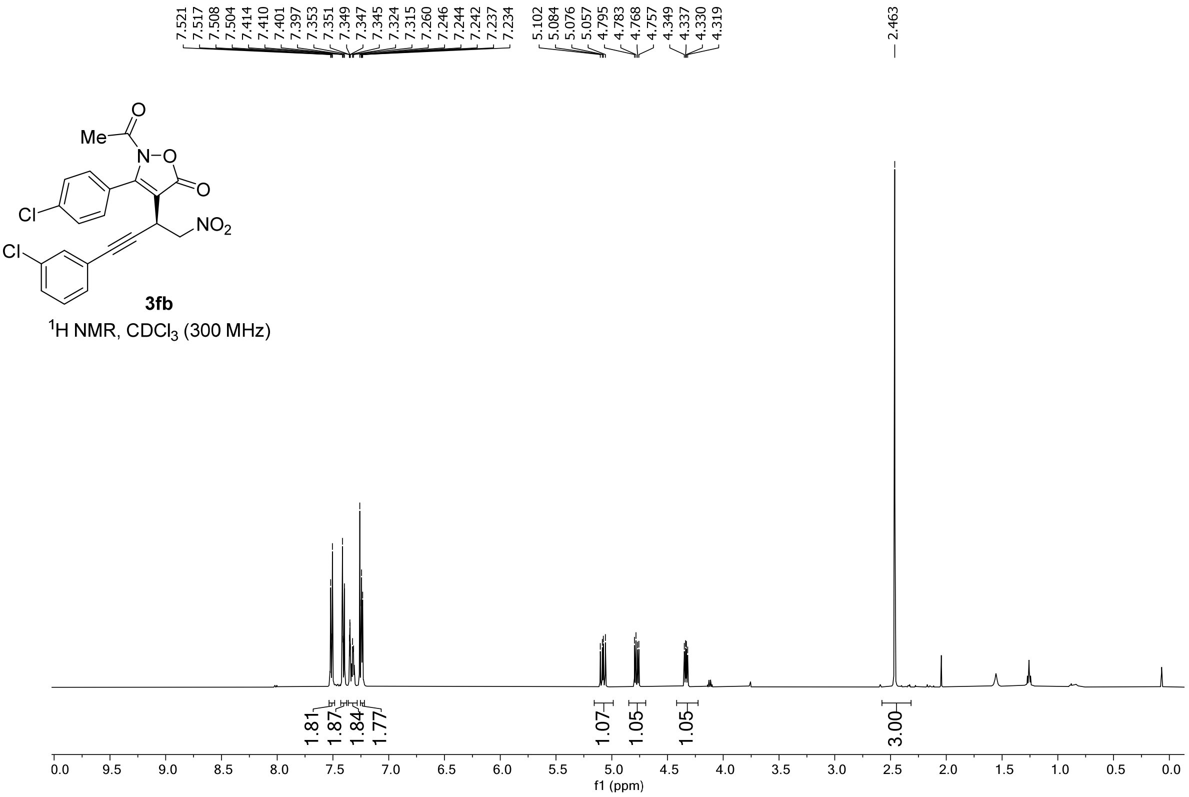

Me
N
O
O
O
Cl
NO2
Cl
3fb
1H NMR, CDCl3 (300 MHz)
7.521 7.517 7.508 7.504 7.414 7.410 7.401 7.397 7.353 7.351 7.349 7.347 7.345 7.324 7.315 7.260 7.246 7.244 7.242 7.237 7.234
5.102 5.084 5.076 5.057 4.795 4.783 4.768 4.757 4.349 4.337 4.330 4.319
2.463
1.81 1.87 1.84 1.77 1.07 1.05 1.05 3.00
f1 (ppm)

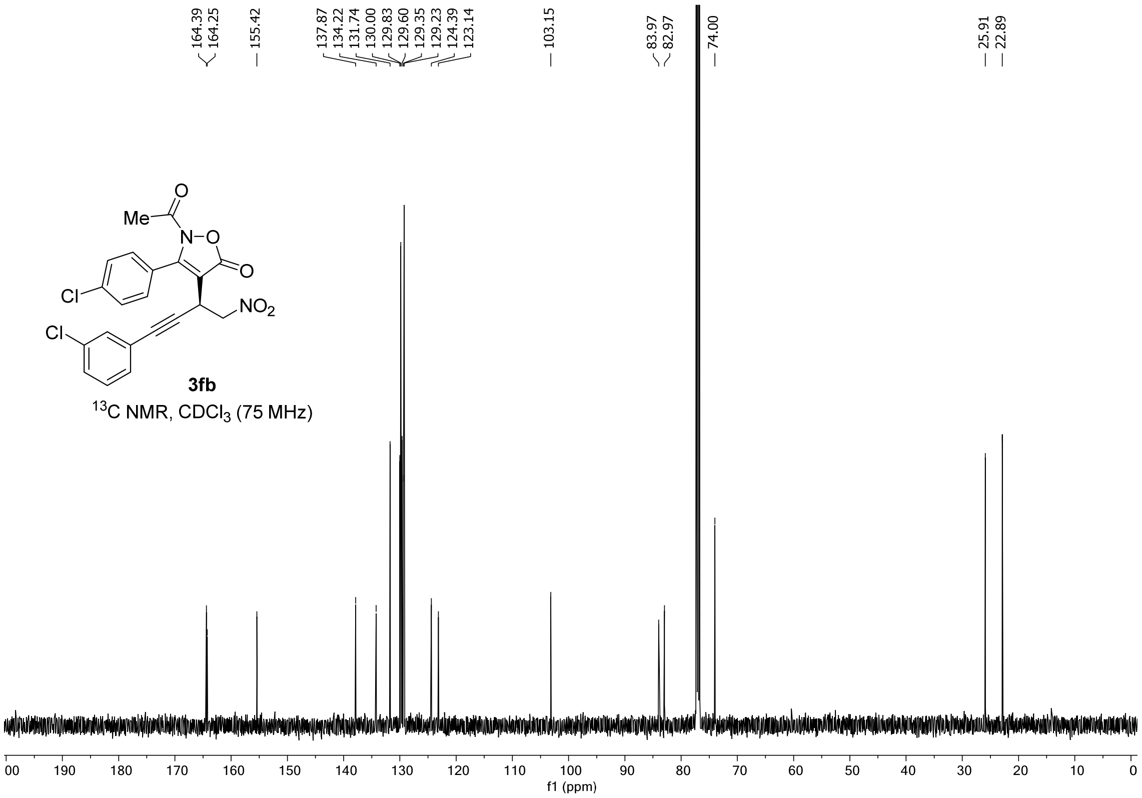

Me
N
O
O
O
Cl
NO2
Cl
3fb
13C NMR, CDCl3 (75 MHz)
164.39 164.25
155.42
137.87 134.22 131.74 130.00 129.83 129.60 129.35 129.23 124.39 123.14
103.15
83.97 82.97
74.00
25.91 22.89
f1 (ppm)

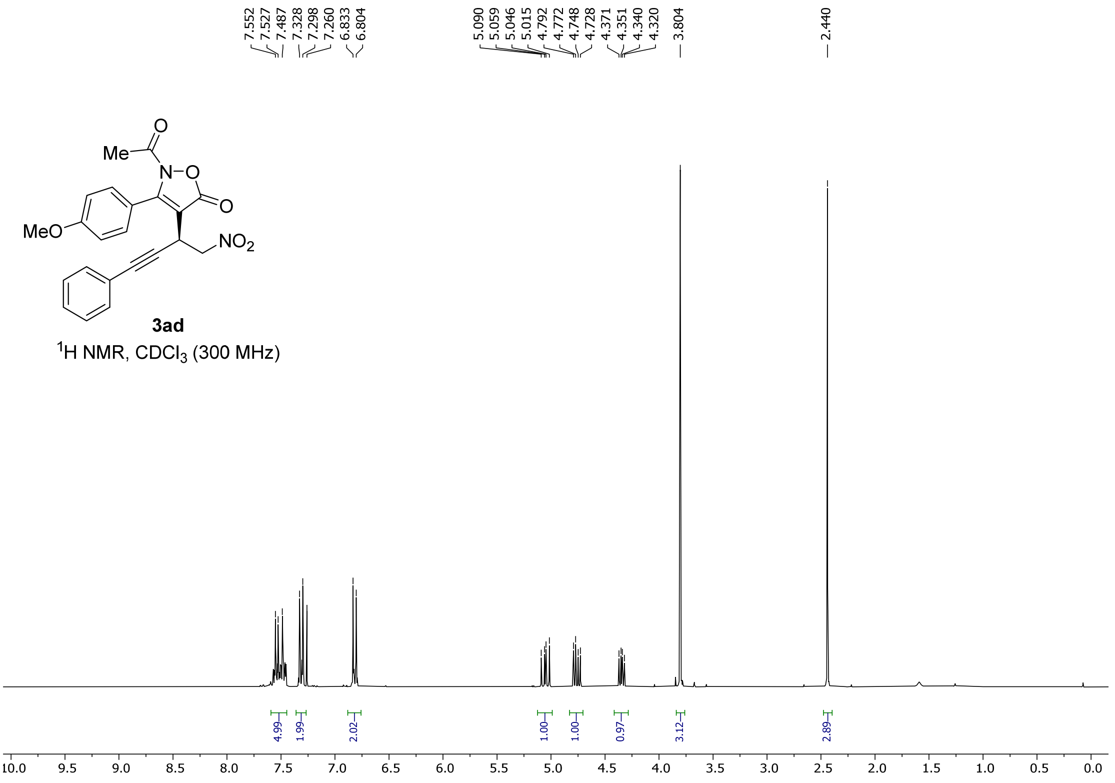

3ad

^{1}H NMR, $CDCl_3$ (300 MHz)

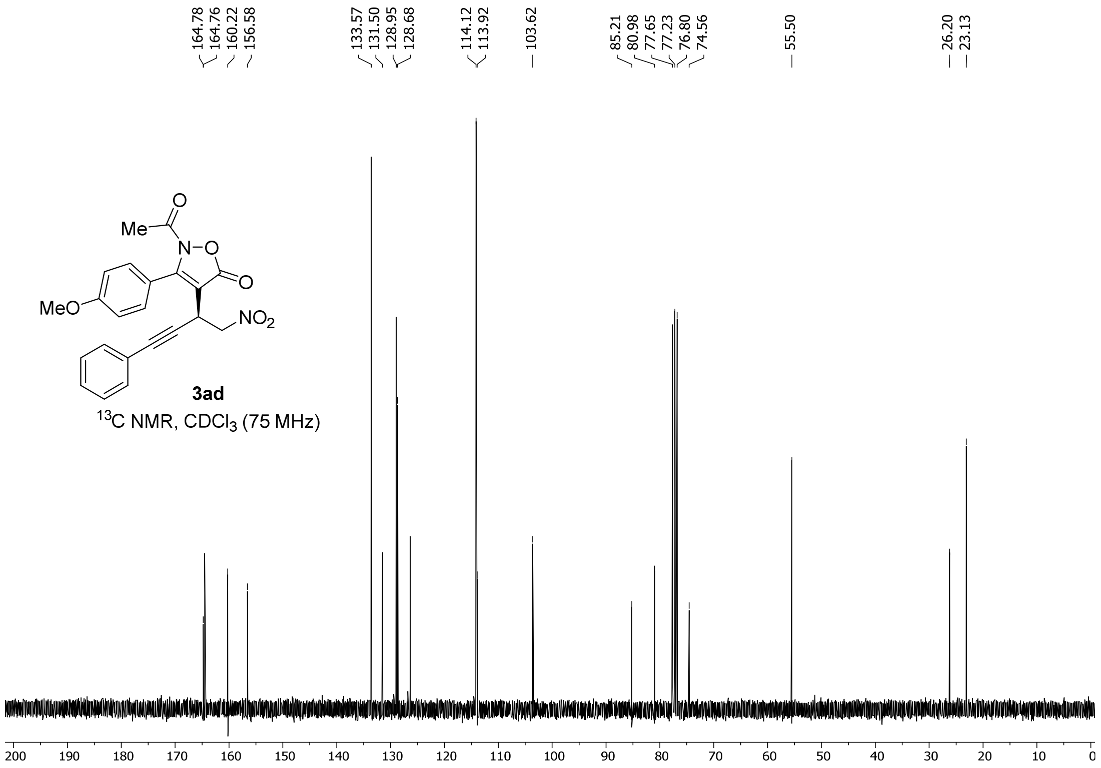

3ad

^{13}C NMR, $CDCl_3$ (75 MHz)

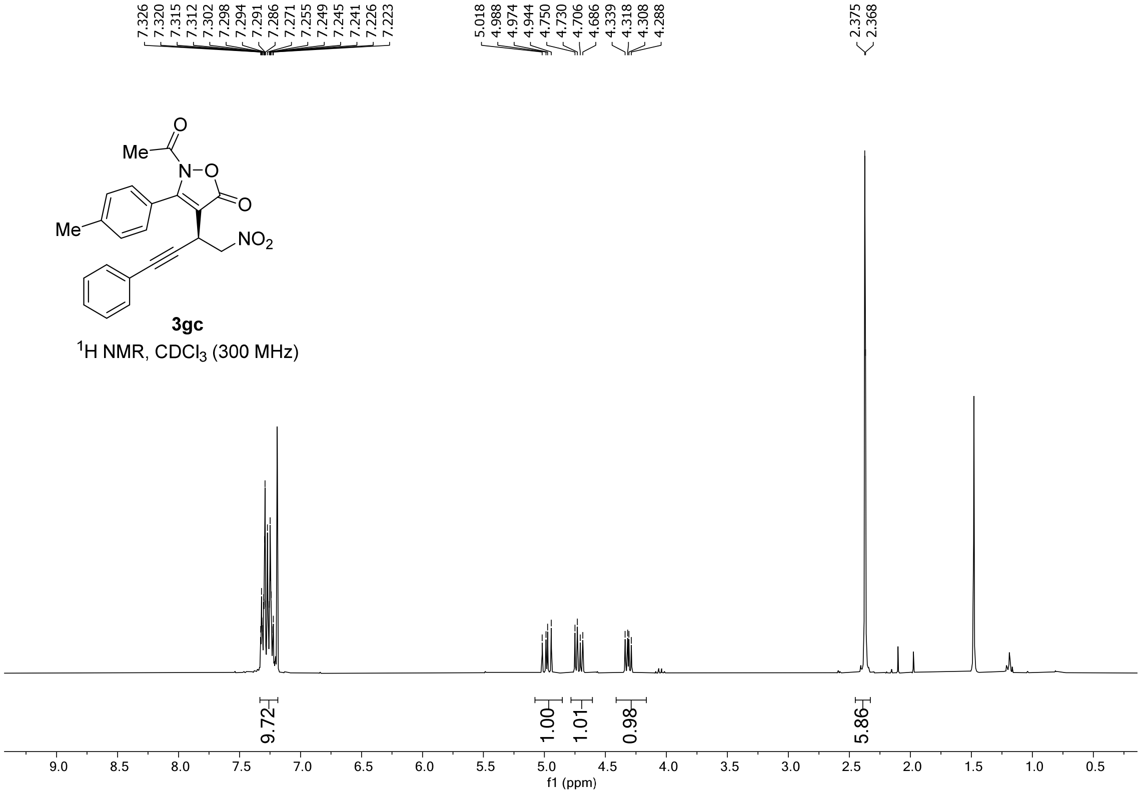
7.326 7.320 7.315 7.312 7.302 7.298 7.294 7.291 7.286 7.271 7.255 7.249 7.245 7.241 7.226 7.223
5.018 4.988 4.974 4.944 4.750 4.730 4.706 4.686 4.339 4.318 4.308 4.288
2.375 2.368
Me
N–O
O
NO2
3gc
1H NMR, CDCl3 (300 MHz)
9.72
1.00
1.01
0.98
5.86
f1 (ppm)

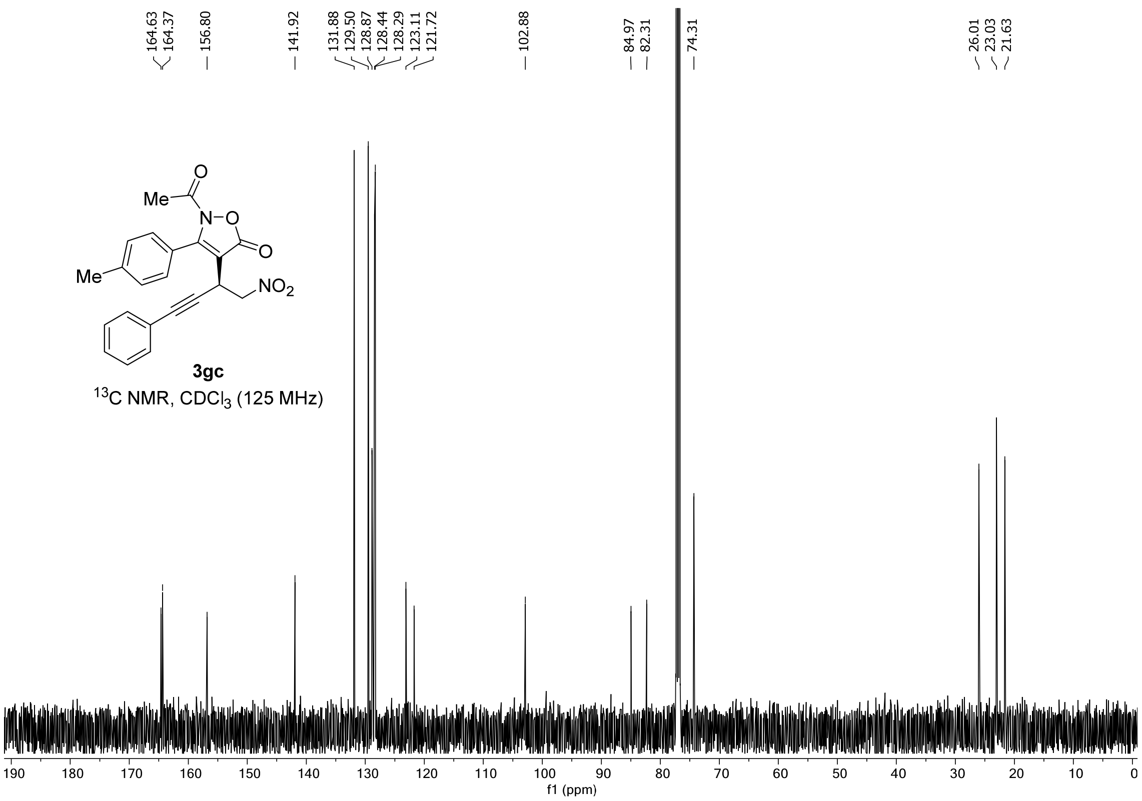
164.63 164.37 156.80 141.92 131.88 129.50 128.87 128.44 128.29 123.11 121.72 102.88 84.97 82.31 74.31 26.01 23.03 21.63
Me
N–O
O
NO2
3gc
13C NMR, CDCl3 (125 MHz)
f1 (ppm)

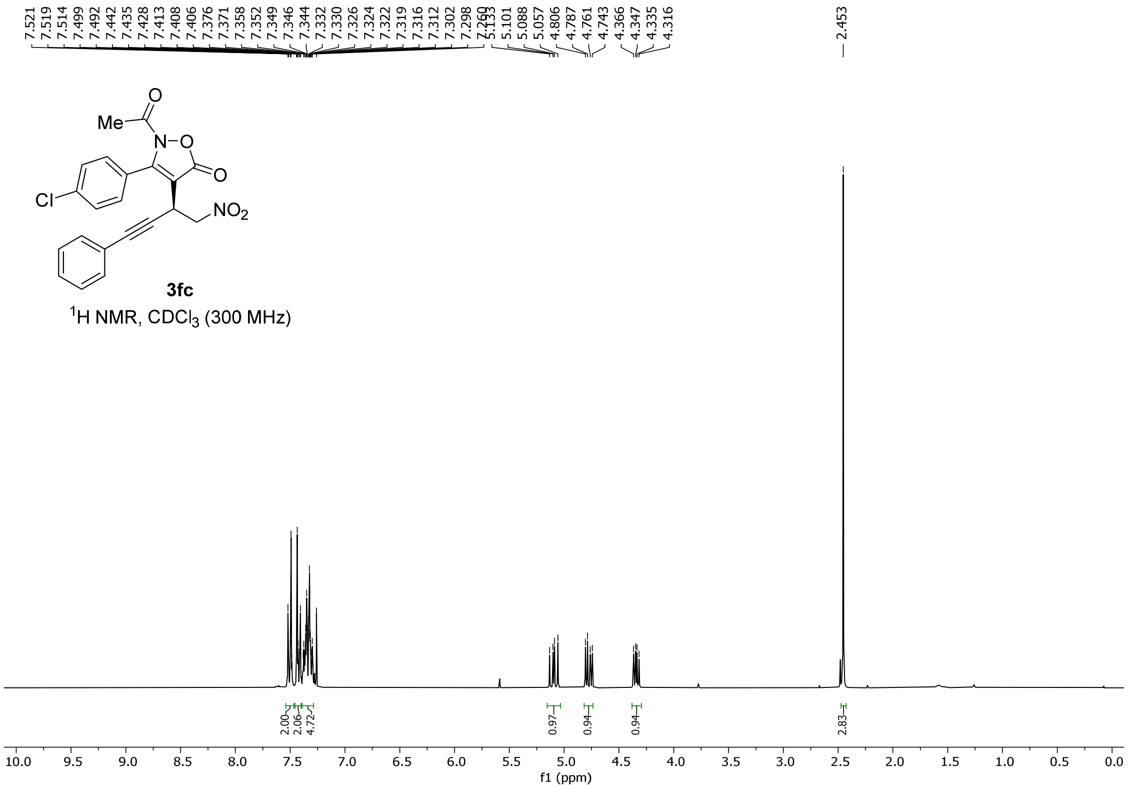

3fc

^{1}H NMR, $CDCl_3$ (300 MHz)

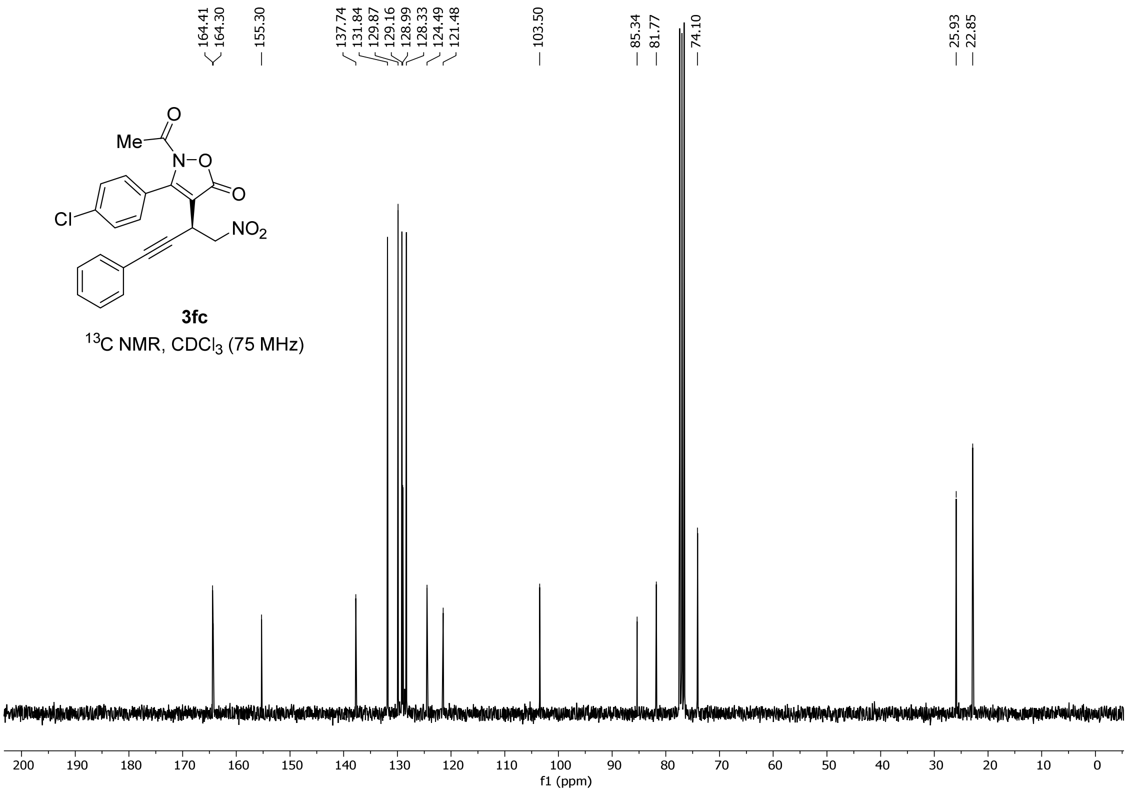

3fc

^{13}C NMR, $CDCl_3$ (75 MHz)

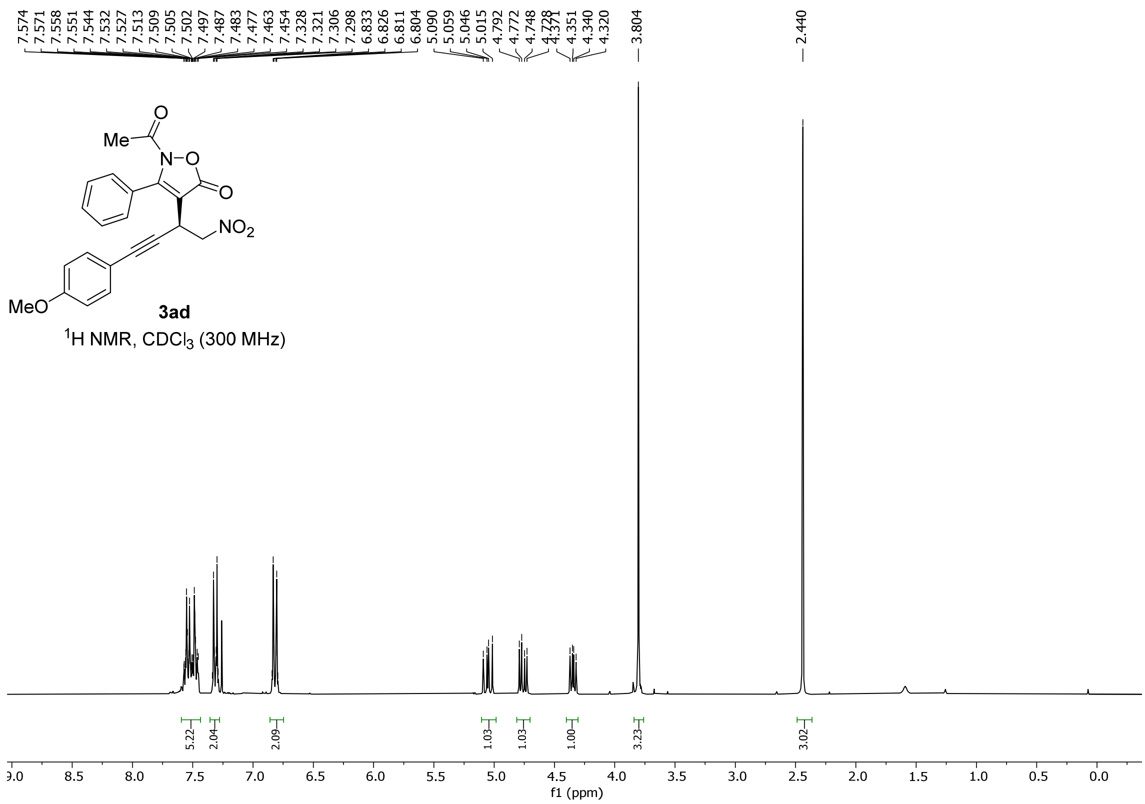
7.574
7.571
7.558
7.551
7.544
7.532
7.527
7.513
7.509
7.505
7.502
7.497
7.487
7.483
7.477
7.463
7.454
7.328
7.321
7.306
7.298
6.833
6.826
6.811
6.804
5.090
5.059
5.046
5.015
4.792
4.772
4.748
4.728
4.371
4.351
4.340
4.320
3.804
2.440
Me
O
N
O
O
NO2
MeO
3ad
1H NMR, CDCl3 (300 MHz)
5.22
2.04
2.09
1.03
1.03
1.00
3.23
3.02
f1 (ppm)

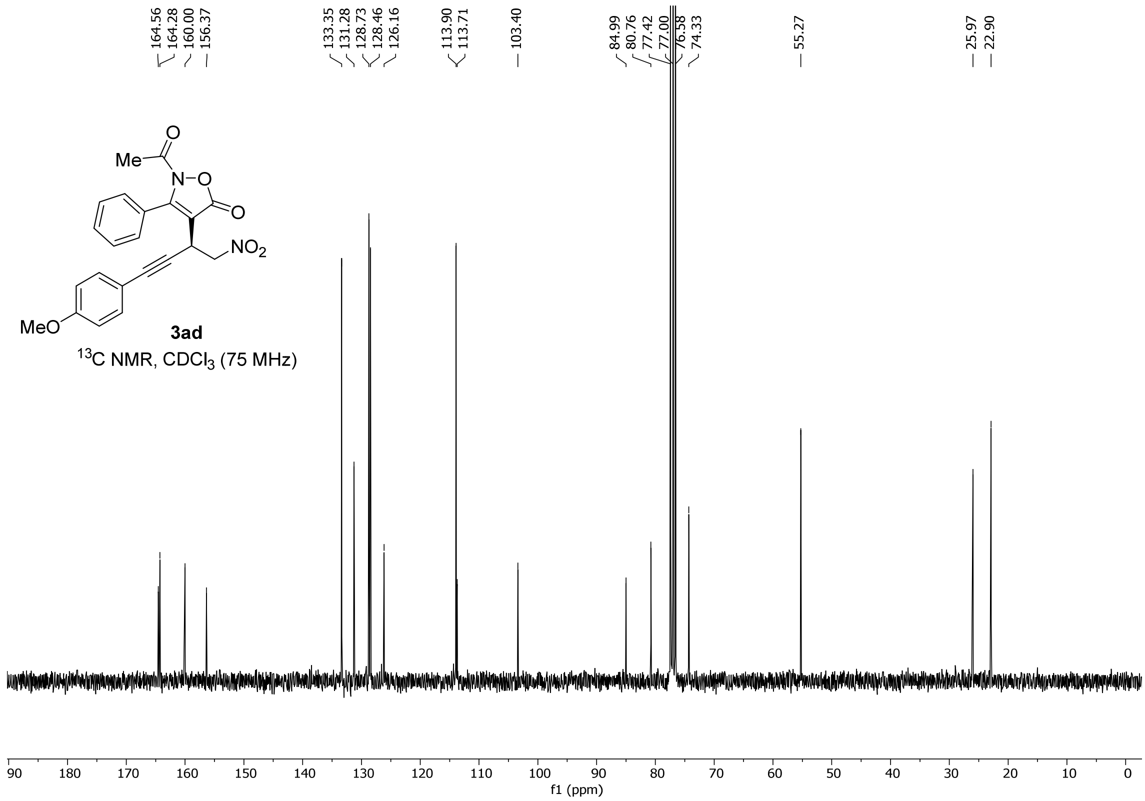
164.56
164.28
160.00
156.37
133.35
131.28
128.73
128.46
126.16
113.90
113.71
103.40
84.99
80.76
77.42
77.00
76.58
74.33
55.27
25.97
22.90
Me
O
N
O
O
NO2
MeO
3ad
13C NMR, CDCl3 (75 MHz)
f1 (ppm)

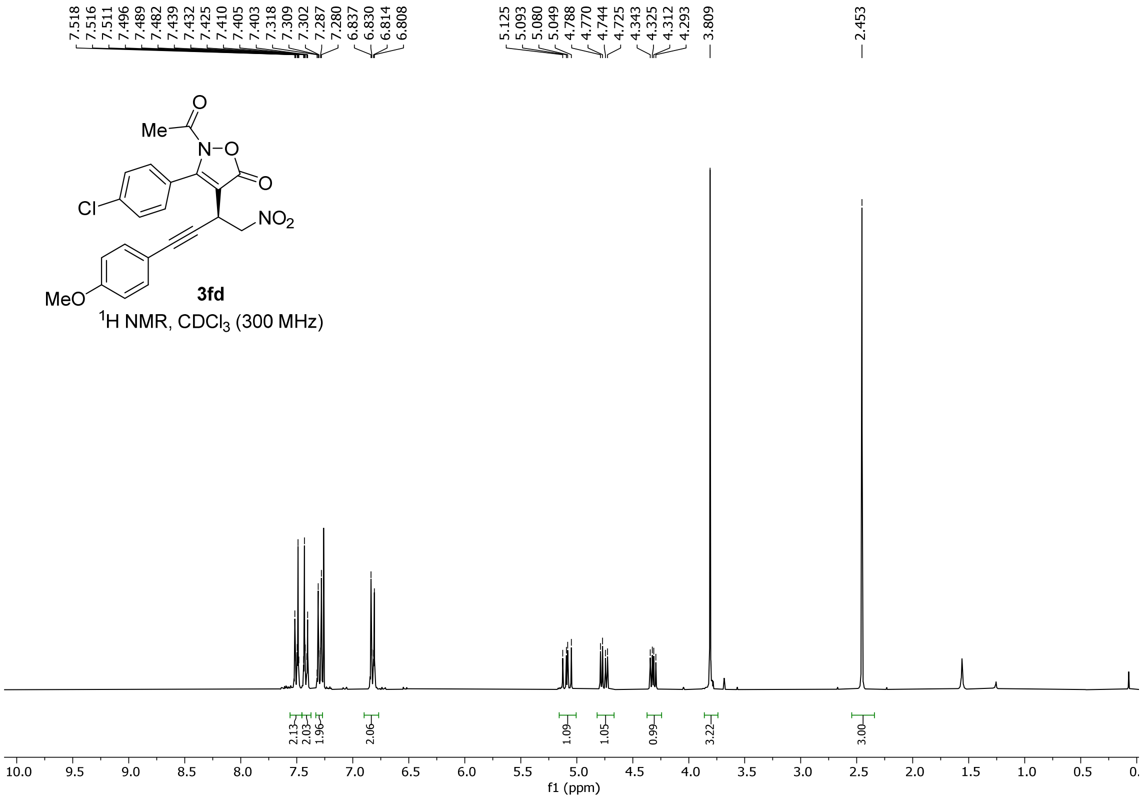
3fd
^{1}H NMR, CDCl$_3$ (300 MHz)
f1 (ppm)

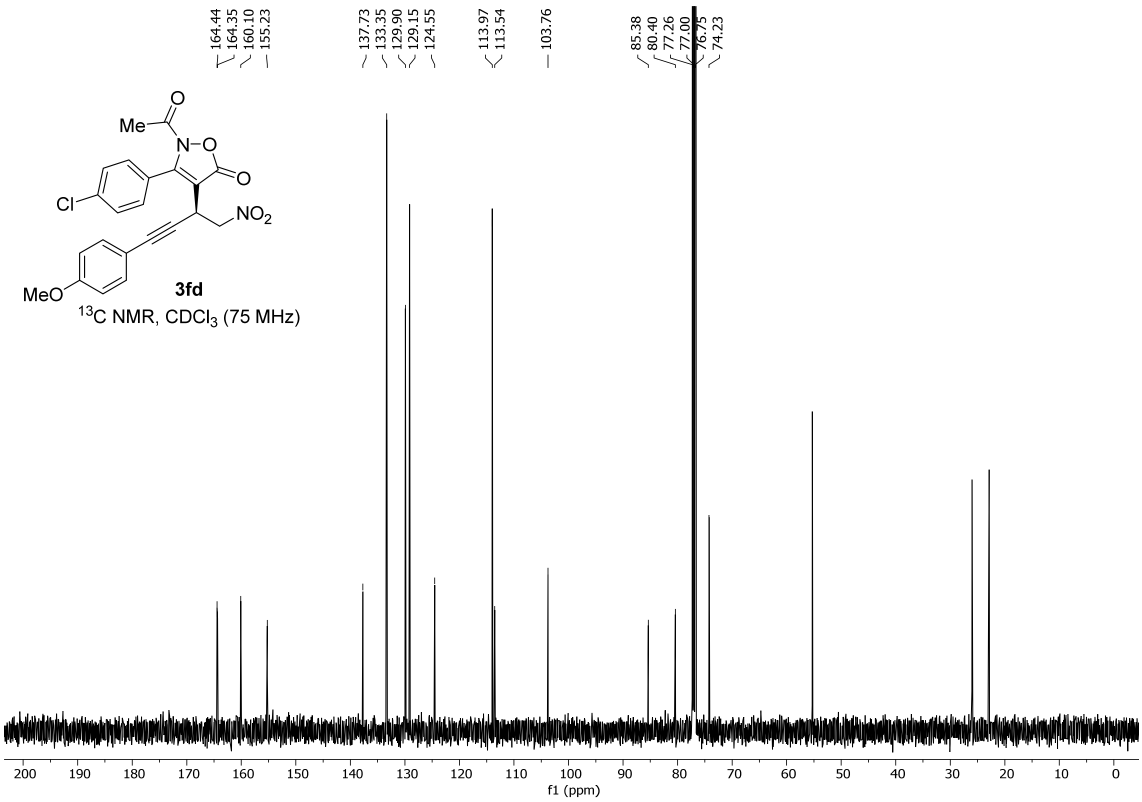
3fd
^{13}C NMR, CDCl$_3$ (75 MHz)
f1 (ppm)

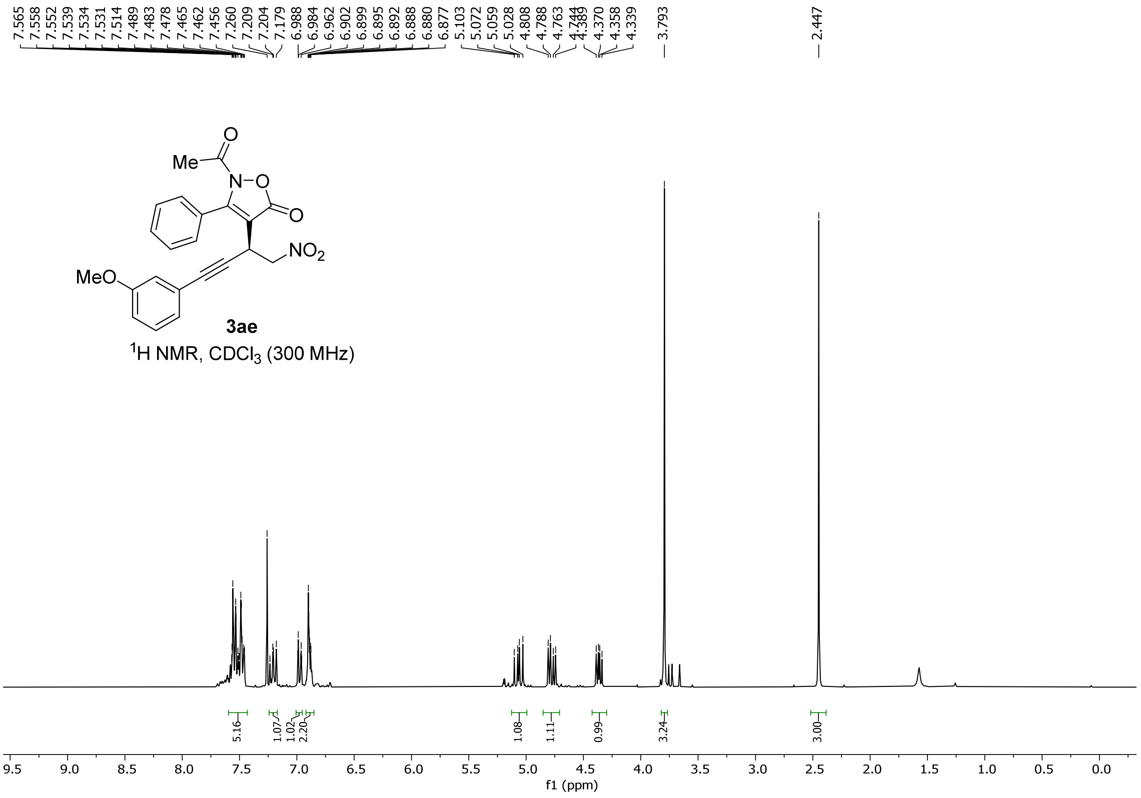
7.565
7.558
7.552
7.539
7.534
7.531
7.514
7.489
7.483
7.478
7.465
7.462
7.456
7.260
7.209
7.204
7.179
6.988
6.984
6.962
6.902
6.899
6.895
6.892
6.888
6.880
6.877
5.103
5.072
5.059
5.028
4.808
4.788
4.763
4.744
4.389
4.370
4.358
4.339
3.793
2.447
Me
O
N
O
O
NO2
MeO
3ae
1H NMR, CDCl3 (300 MHz)
5.16
1.07
1.02
2.20
1.08
1.11
0.99
3.24
3.00
f1 (ppm)

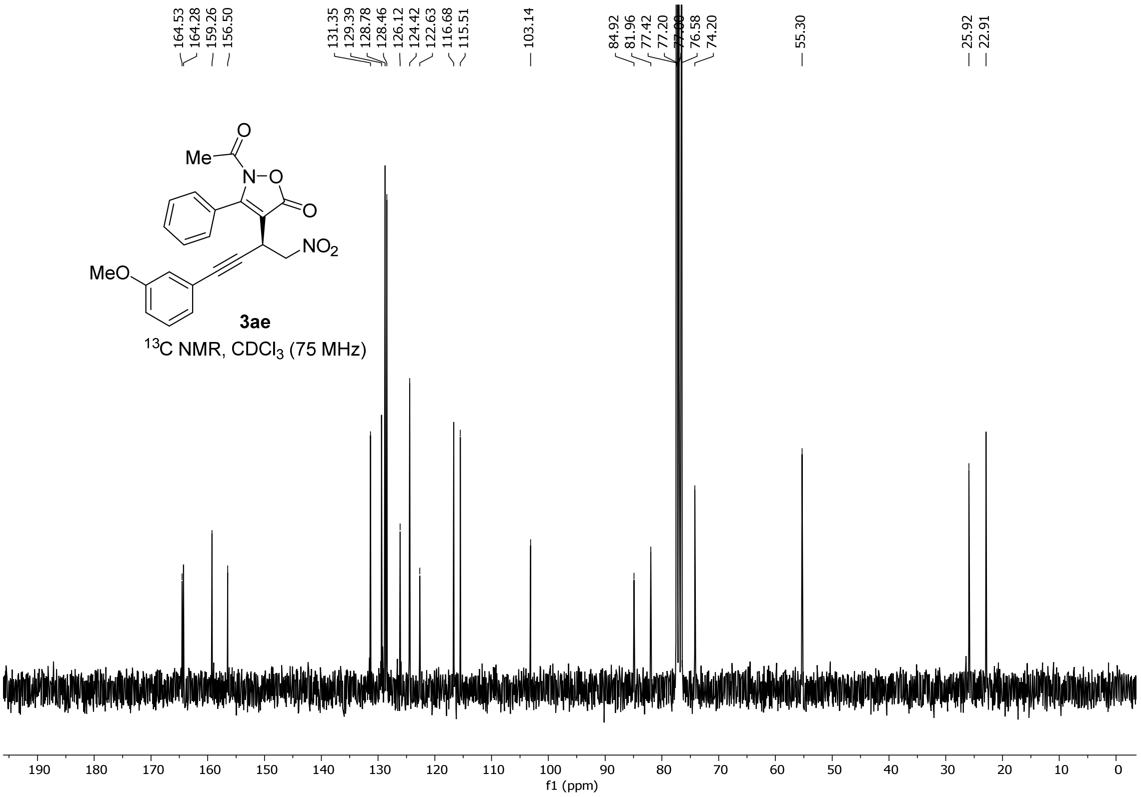
164.53
164.28
159.26
156.50
131.35
129.39
128.78
128.46
126.12
124.42
122.63
116.68
115.51
103.14
84.92
81.96
77.42
77.20
77.00
76.58
74.20
55.30
25.92
22.91
Me
O
N
O
O
NO2
MeO
3ae
13C NMR, CDCl3 (75 MHz)
f1 (ppm)

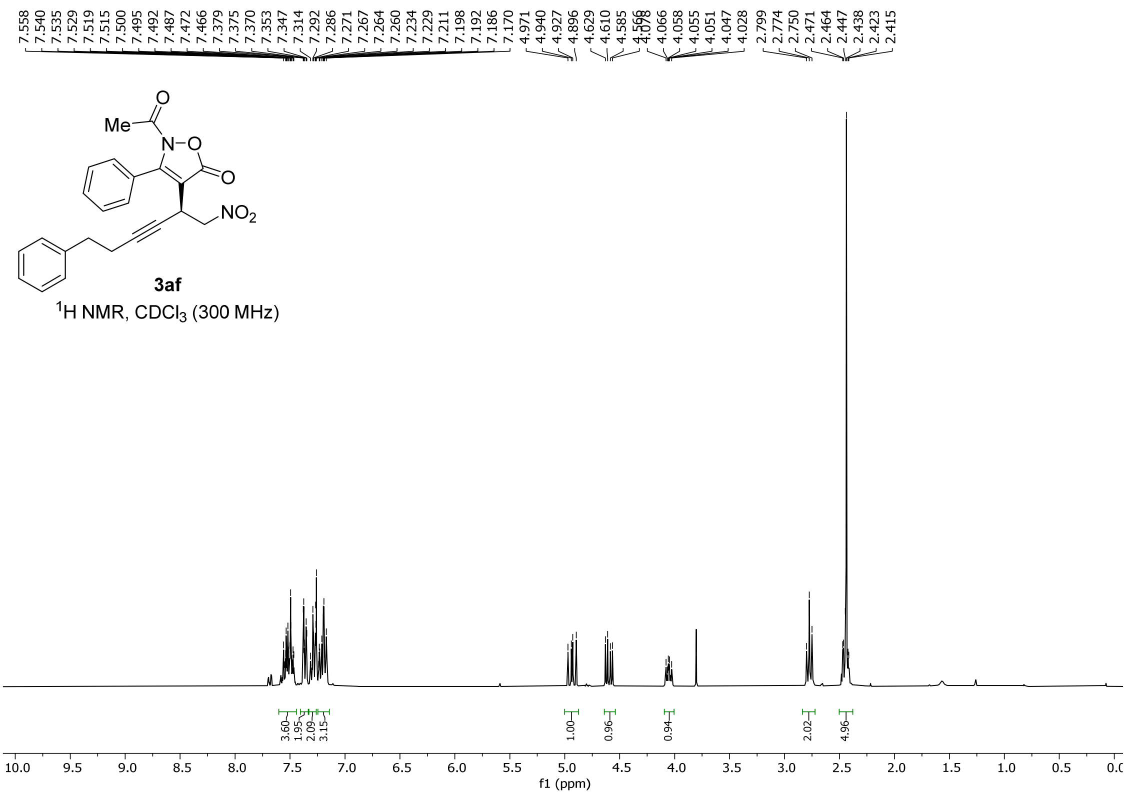
7.558 7.540 7.535 7.529 7.519 7.515 7.500 7.495 7.492 7.487 7.472 7.466 7.379 7.375 7.370 7.353 7.347 7.314 7.292 7.286 7.271 7.267 7.264 7.260 7.234 7.229 7.211 7.198 7.192 7.186 7.170 4.971 4.940 4.927 4.896 4.629 4.610 4.585 4.566 4.078 4.066 4.058 4.055 4.051 4.047 4.028 2.799 2.774 2.750 2.471 2.464 2.447 2.438 2.423 2.415
Me
O
N
O
O
NO2
3af
1H NMR, CDCl3 (300 MHz)
3.60 1.95 2.09 3.15 1.00 0.96 0.94 2.02 4.96
f1 (ppm)

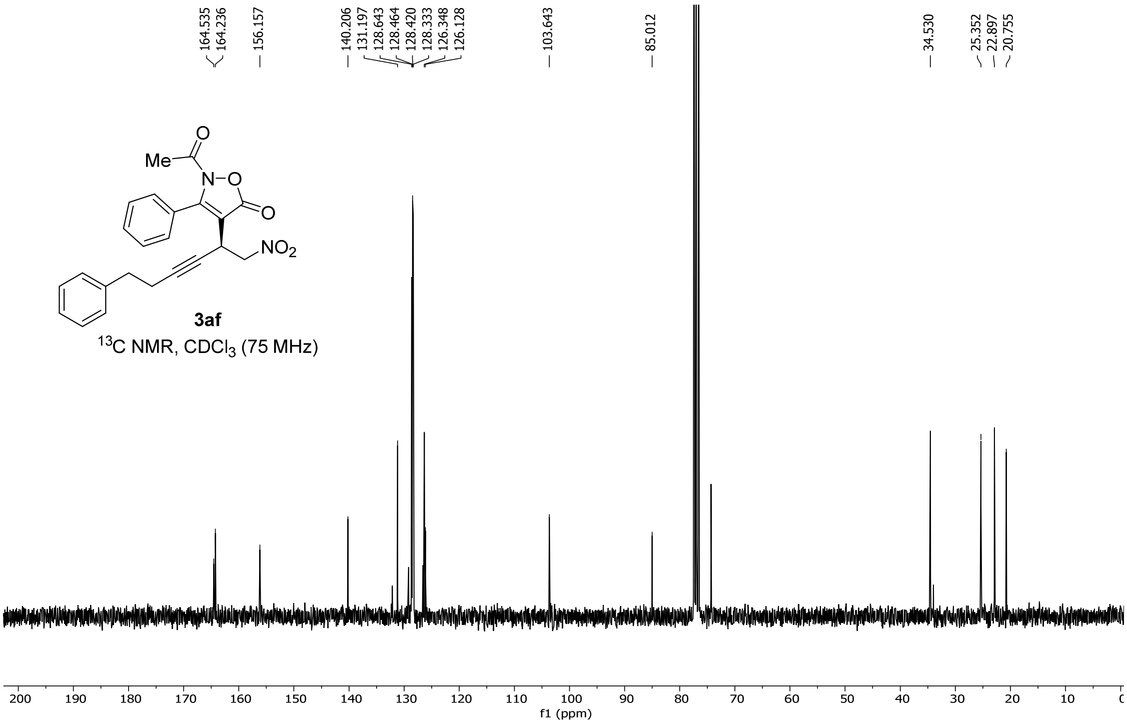
164.535 164.236 156.157 140.206 131.197 128.643 128.464 128.420 128.333 126.348 126.128 103.643 85.012 34.530 25.352 22.897 20.755
Me
O
N
O
O
NO2
3af
13C NMR, CDCl3 (75 MHz)
f1 (ppm)

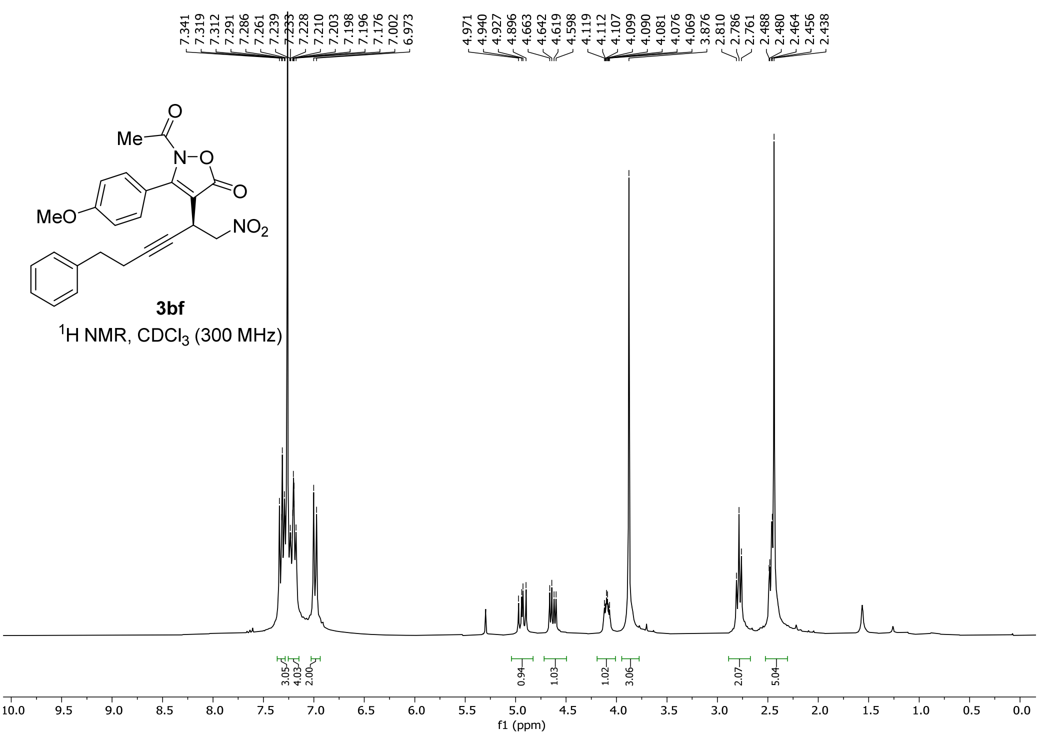
7.341
7.319
7.312
7.291
7.286
7.261
7.239
7.233
7.228
7.210
7.203
7.198
7.196
7.176
7.002
6.973
4.971
4.940
4.927
4.896
4.663
4.642
4.619
4.598
4.119
4.112
4.107
4.099
4.090
4.081
4.076
4.069
3.876
2.810
2.786
2.761
2.488
2.480
2.464
2.456
2.438
Me
N–O
O
O
MeO
NO2
3bf
1H NMR, CDCl3 (300 MHz)
3.05
4.03
2.00
0.94
1.03
1.02
3.06
2.07
5.04
10.0 9.5 9.0 8.5 8.0 7.5 7.0 6.5 6.0 5.5 5.0 4.5 4.0 3.5 3.0 2.5 2.0 1.5 1.0 0.5 0.0
f1 (ppm)

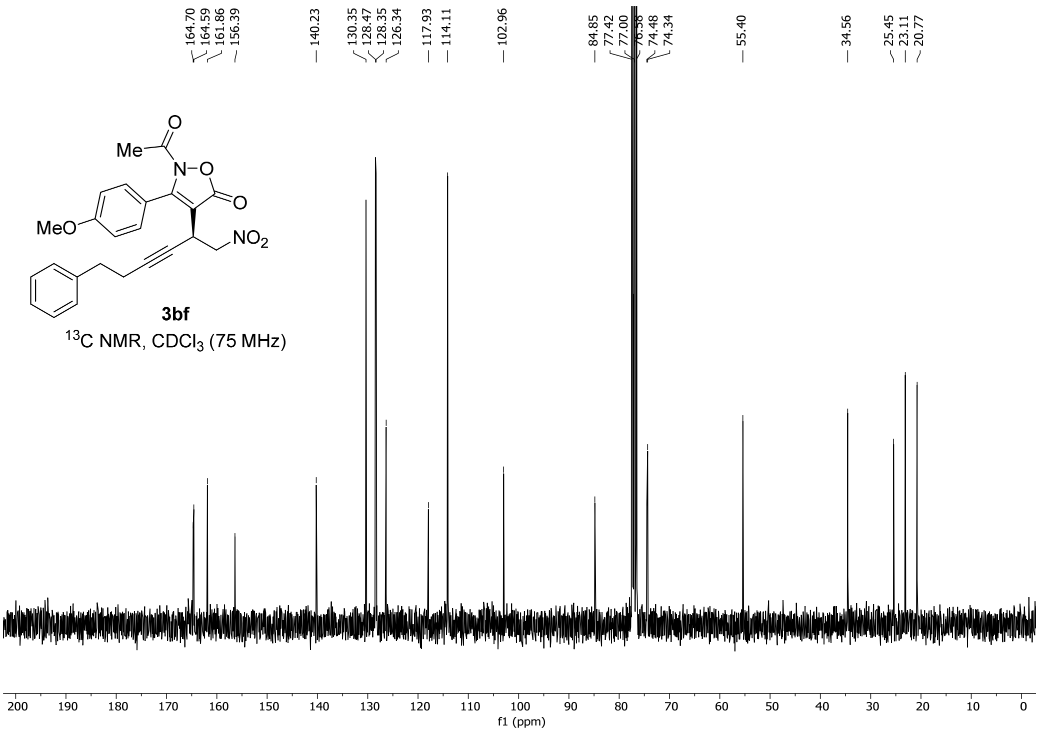
164.70
164.59
161.86
156.39
140.23
130.35
128.47
128.35
126.34
117.93
114.11
102.96
84.85
77.42
77.00
76.58
74.48
74.34
55.40
34.56
25.45
23.11
20.77
Me
N–O
O
O
MeO
NO2
3bf
13C NMR, CDCl3 (75 MHz)
200 190 180 170 160 150 140 130 120 110 100 90 80 70 60 50 40 30 20 10 0
f1 (ppm)

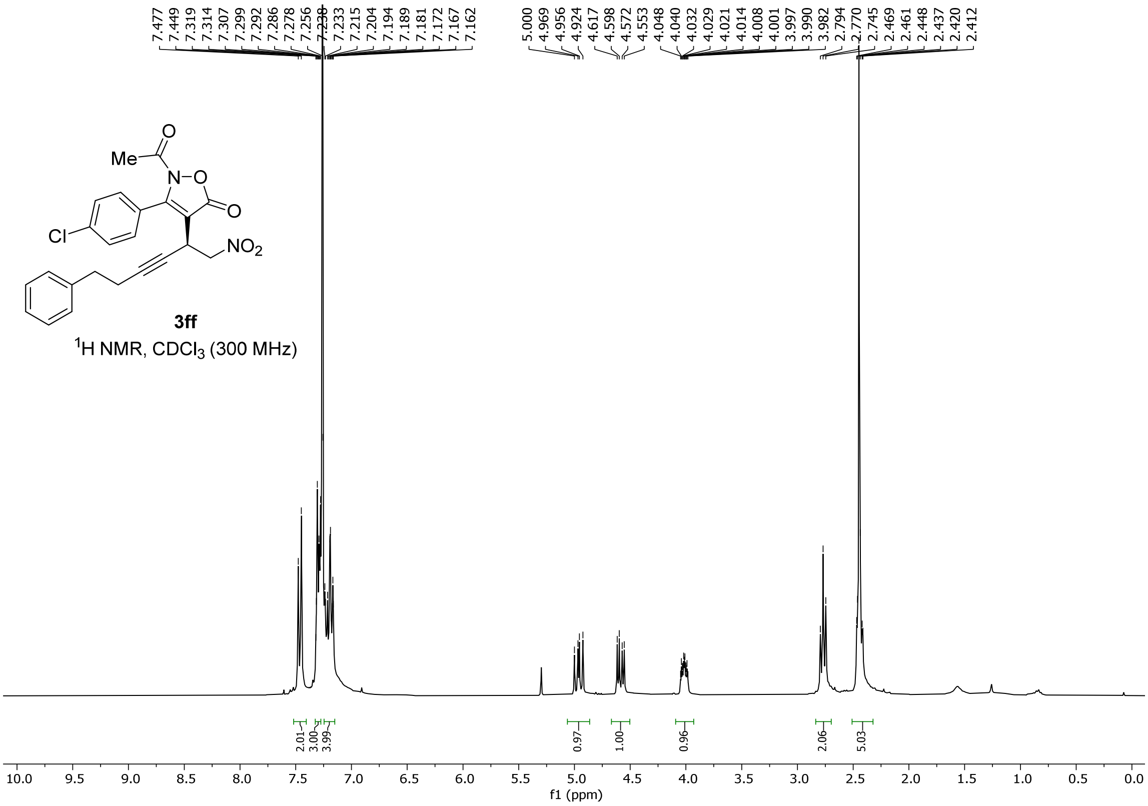

3ff

^{1}H NMR, $CDCl_3$ (300 MHz)

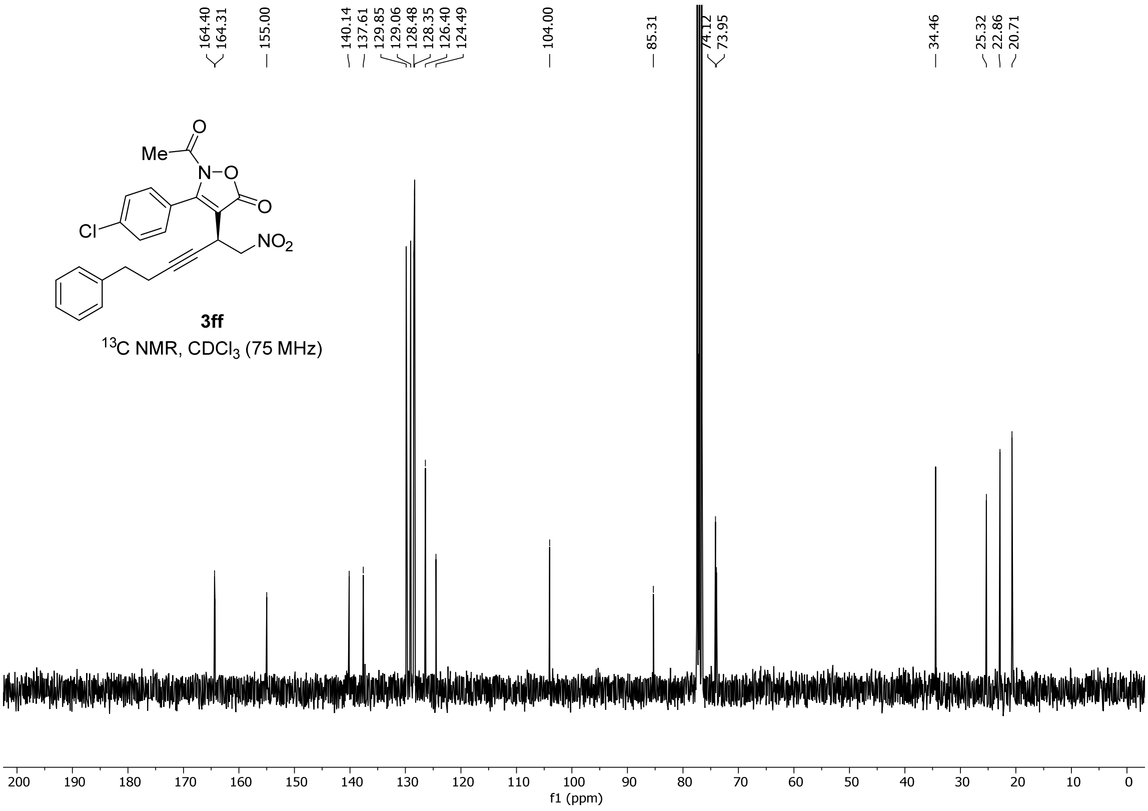

3ff

^{13}C NMR, $CDCl_3$ (75 MHz)

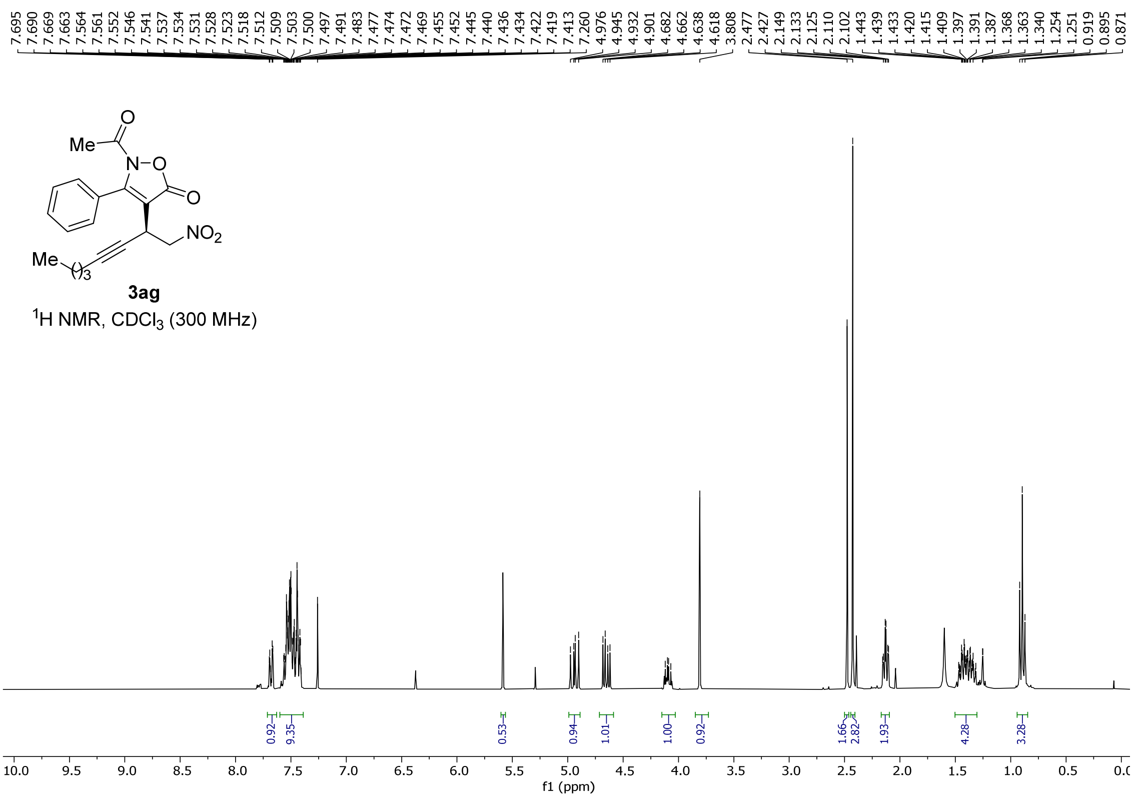

3ag

^{1}H NMR, $CDCl_3$ (300 MHz)

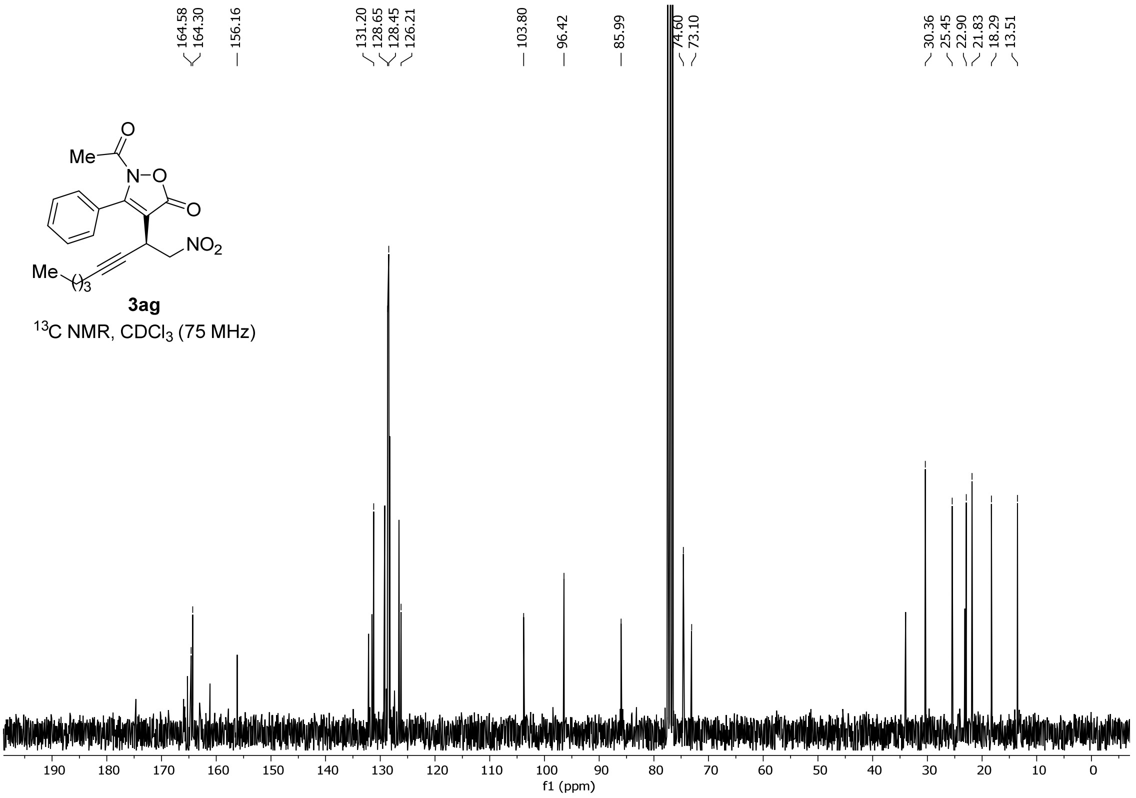

3ag

^{13}C NMR, $CDCl_3$ (75 MHz)

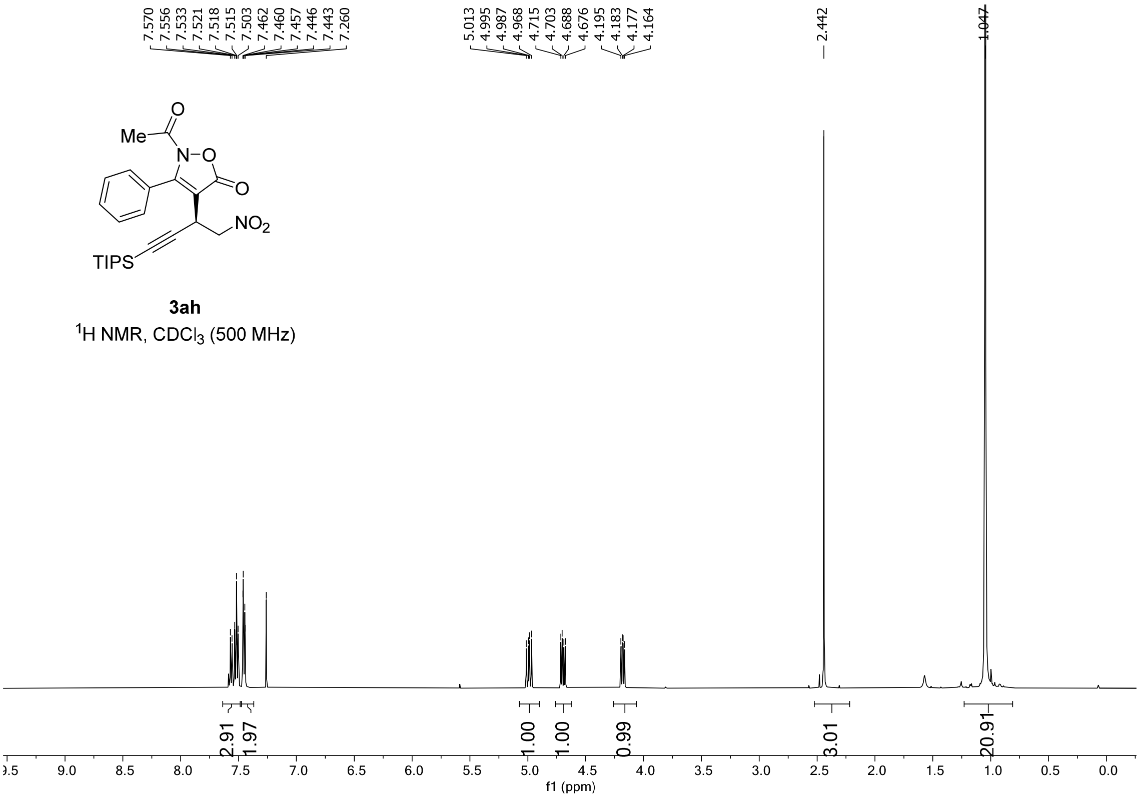
7.570 7.556 7.533 7.521 7.518 7.515 7.503 7.462 7.460 7.457 7.446 7.443 7.260
5.013 4.995 4.987 4.968 4.715 4.703 4.688 4.676 4.195 4.183 4.177 4.164
2.442
1.047
Me
N–O
O
NO2
TIPS
3ah
1H NMR, CDCl3 (500 MHz)
2.91 1.97 1.00 1.00 0.99 3.01 20.91
f1 (ppm)

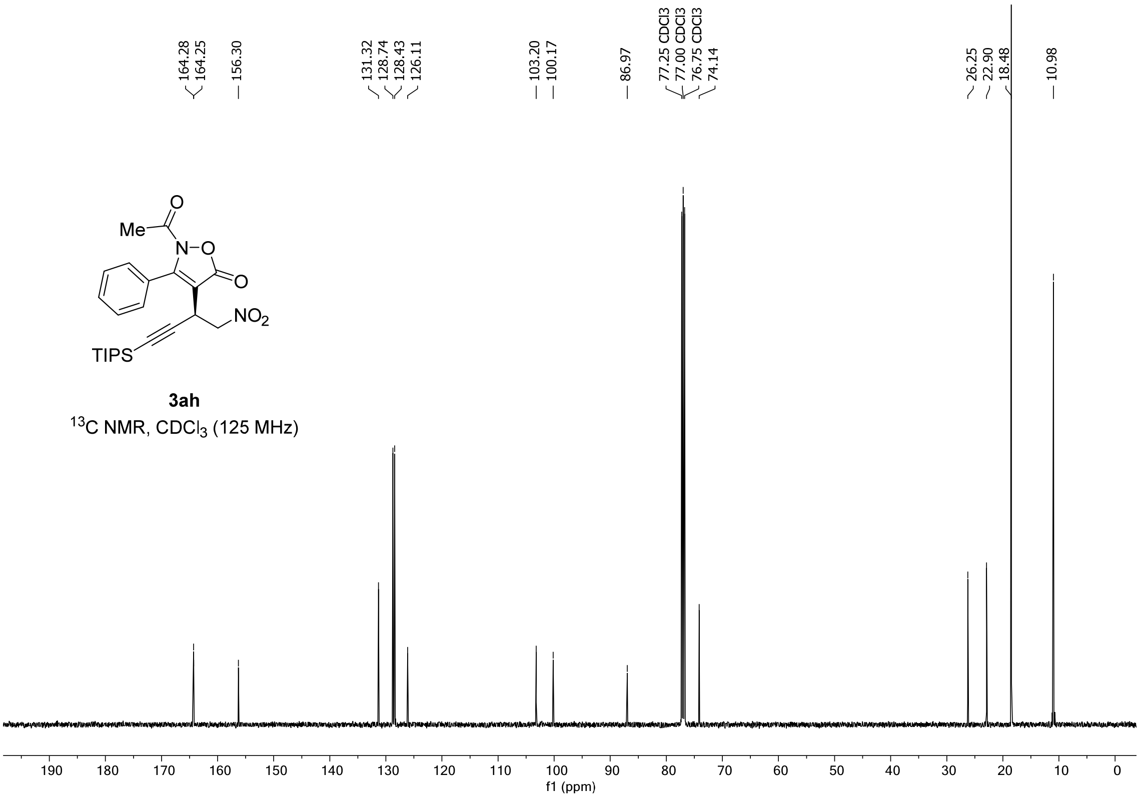
164.28 164.25 156.30
131.32 128.74 128.43 126.11
103.20 100.17
86.97
77.25 CDCl3 77.00 CDCl3 76.75 CDCl3 74.14
26.25 22.90 18.48 10.98
Me
N–O
O
NO2
TIPS
3ah
13C NMR, CDCl3 (125 MHz)
f1 (ppm)

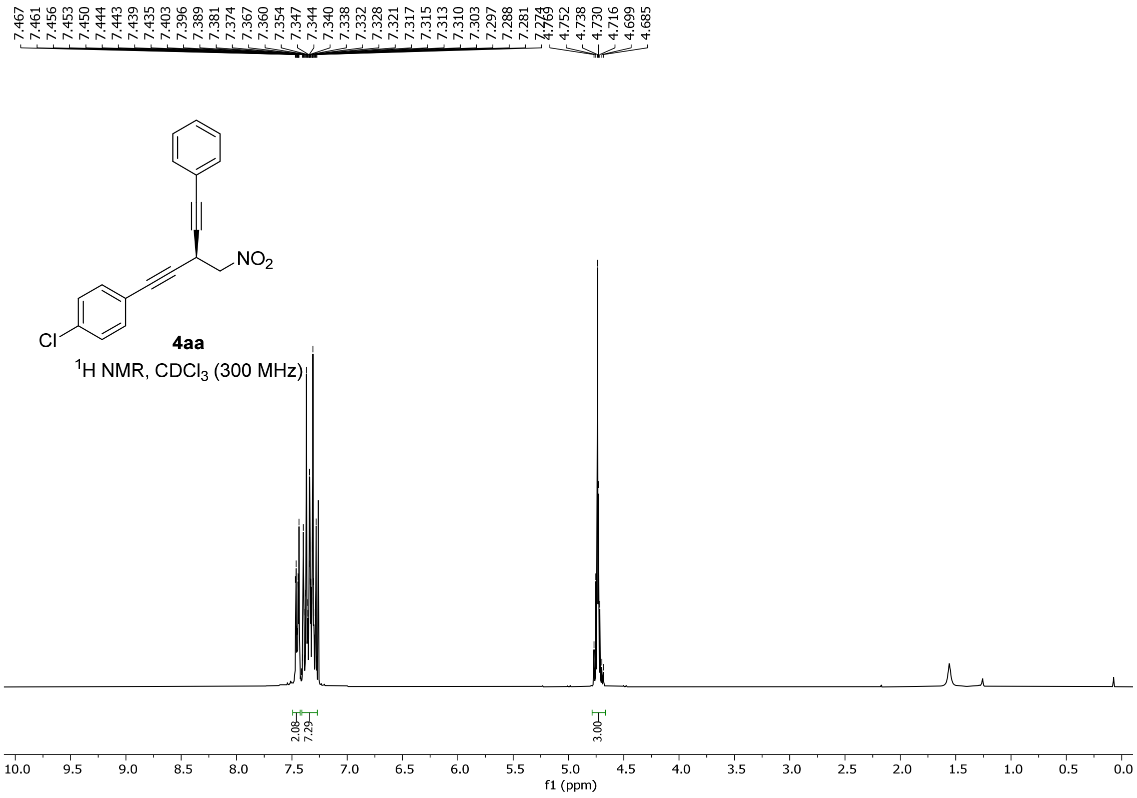
7.467 7.461 7.456 7.453 7.450 7.444 7.443 7.439 7.435 7.403 7.396 7.389 7.381 7.374 7.367 7.360 7.354 7.347 7.344 7.340 7.338 7.332 7.328 7.321 7.317 7.315 7.313 7.310 7.303 7.297 7.288 7.281 7.274 4.769 4.752 4.738 4.730 4.716 4.699 4.685
NO2
Cl
4aa
1H NMR, CDCl3 (300 MHz)
2.08 7.29 3.00
10.0 9.5 9.0 8.5 8.0 7.5 7.0 6.5 6.0 5.5 5.0 4.5 4.0 3.5 3.0 2.5 2.0 1.5 1.0 0.5 0.0
f1 (ppm)

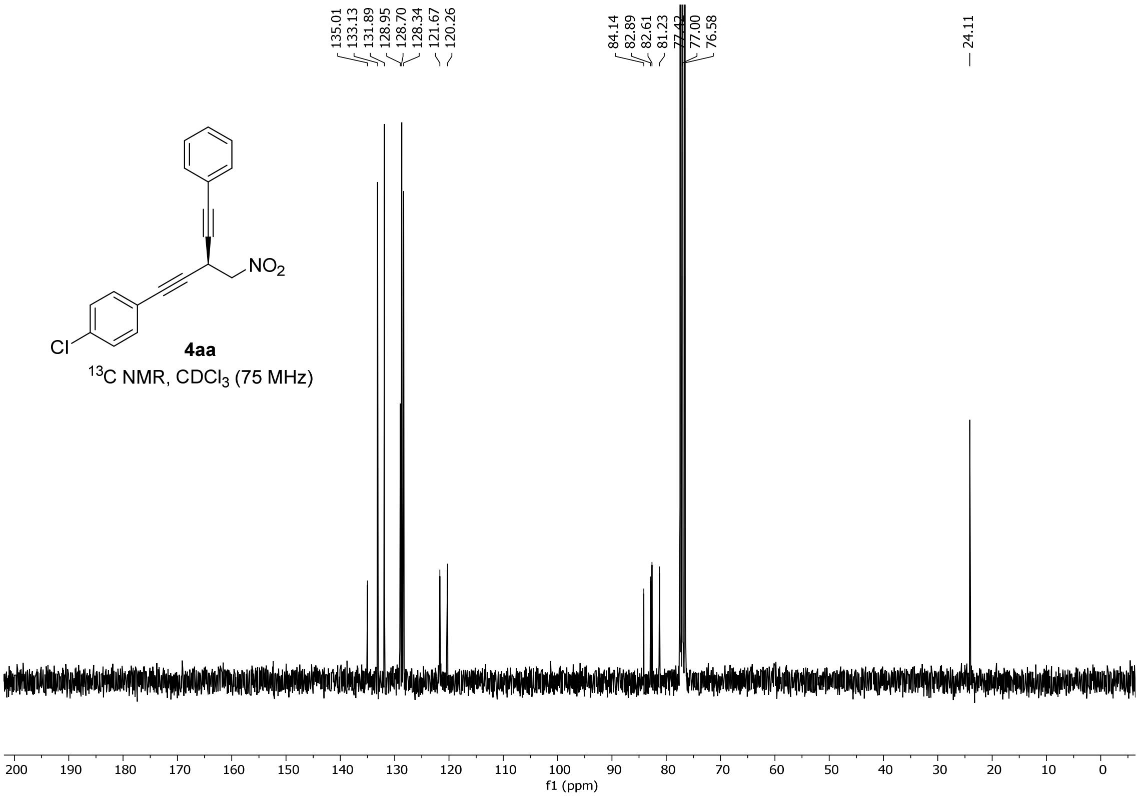
135.01 133.13 131.89 128.95 128.70 128.34 121.67 120.26
84.14 82.89 82.61 81.23 77.42 77.00 76.58
24.11
NO2
Cl
4aa
13C NMR, CDCl3 (75 MHz)
200 190 180 170 160 150 140 130 120 110 100 90 80 70 60 50 40 30 20 10 0
f1 (ppm)

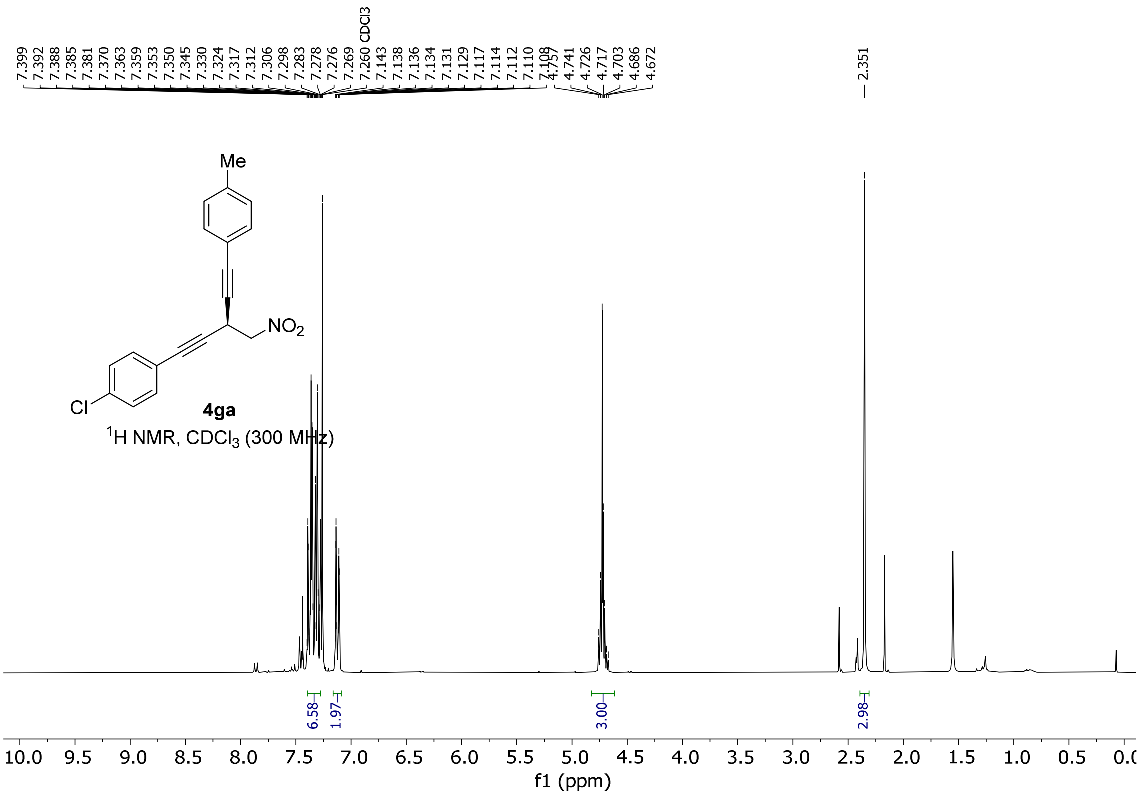

7.399
7.392
7.388
7.385
7.381
7.370
7.363
7.359
7.353
7.350
7.345
7.330
7.324
7.317
7.312
7.306
7.298
7.283
7.278
7.276
7.269
7.260 CDCl3
7.143
7.138
7.136
7.134
7.131
7.129
7.117
7.114
7.112
7.110
4.741
4.726
4.717
4.703
4.686
4.672
2.351
Me
NO2
Cl
4ga
1H NMR, CDCl3 (300 MHz)
6.58
1.97
3.00
2.98
f1 (ppm)

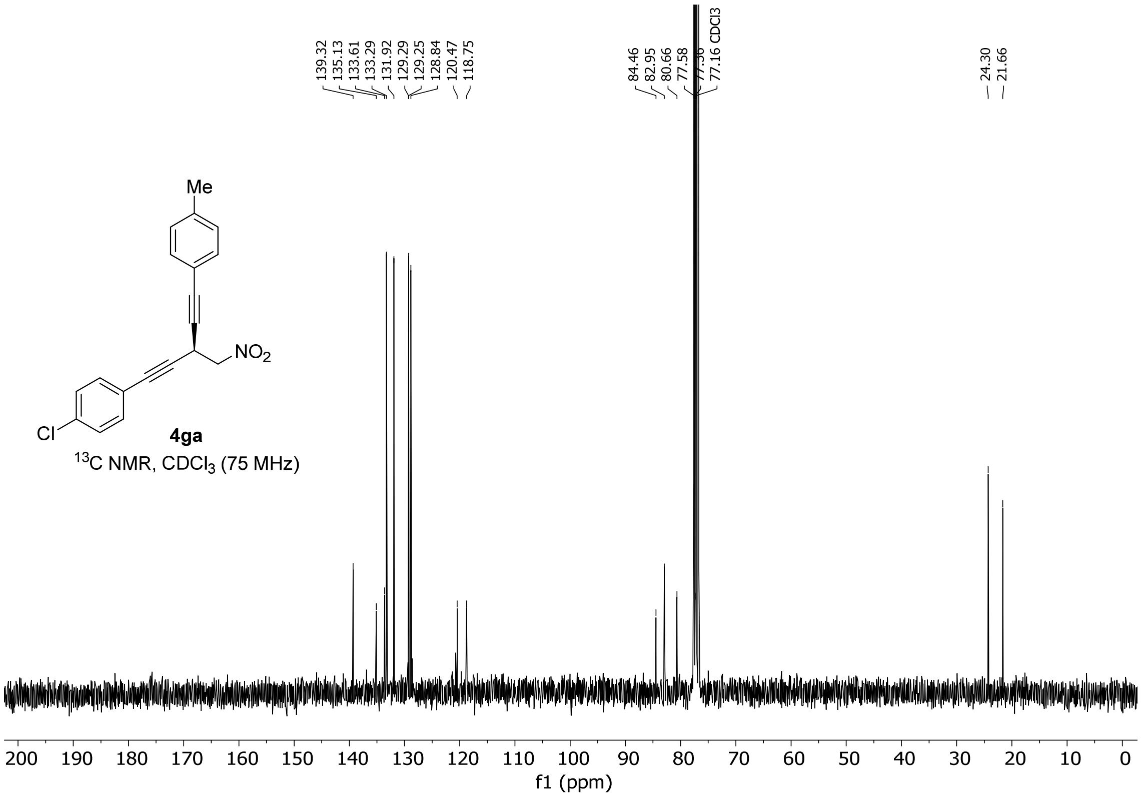

139.32
135.13
133.61
133.29
131.92
129.29
129.25
128.84
120.47
118.75
84.46
82.95
80.66
77.58
77.16 CDCl3
24.30
21.66
Me
NO2
Cl
4ga
13C NMR, CDCl3 (75 MHz)
f1 (ppm)

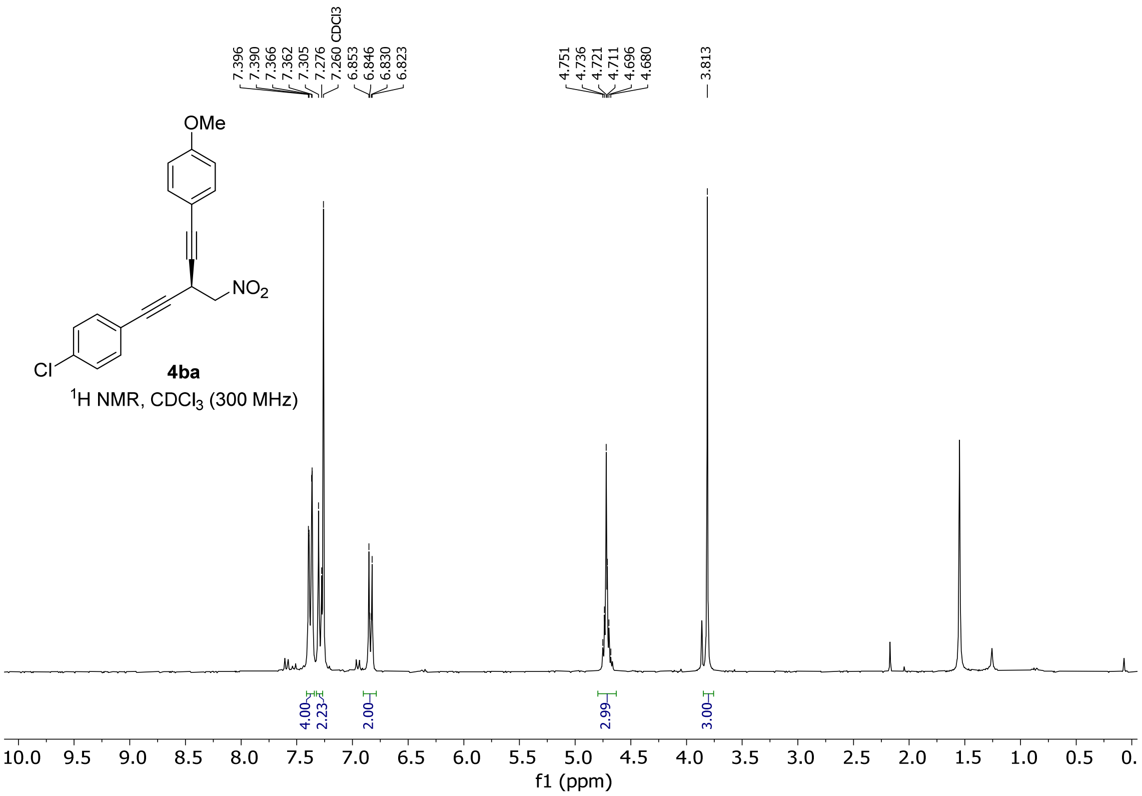
7.396
7.390
7.366
7.362
7.305
7.276
7.260 CDCl3
6.853
6.846
6.830
6.823
4.751
4.736
4.721
4.711
4.696
4.680
3.813
OMe
NO2
Cl
4ba
1H NMR, CDCl3 (300 MHz)
4.00
2.23
2.00
2.99
3.00
10.0 9.5 9.0 8.5 8.0 7.5 7.0 6.5 6.0 5.5 5.0 4.5 4.0 3.5 3.0 2.5 2.0 1.5 1.0 0.5 0.
f1 (ppm)

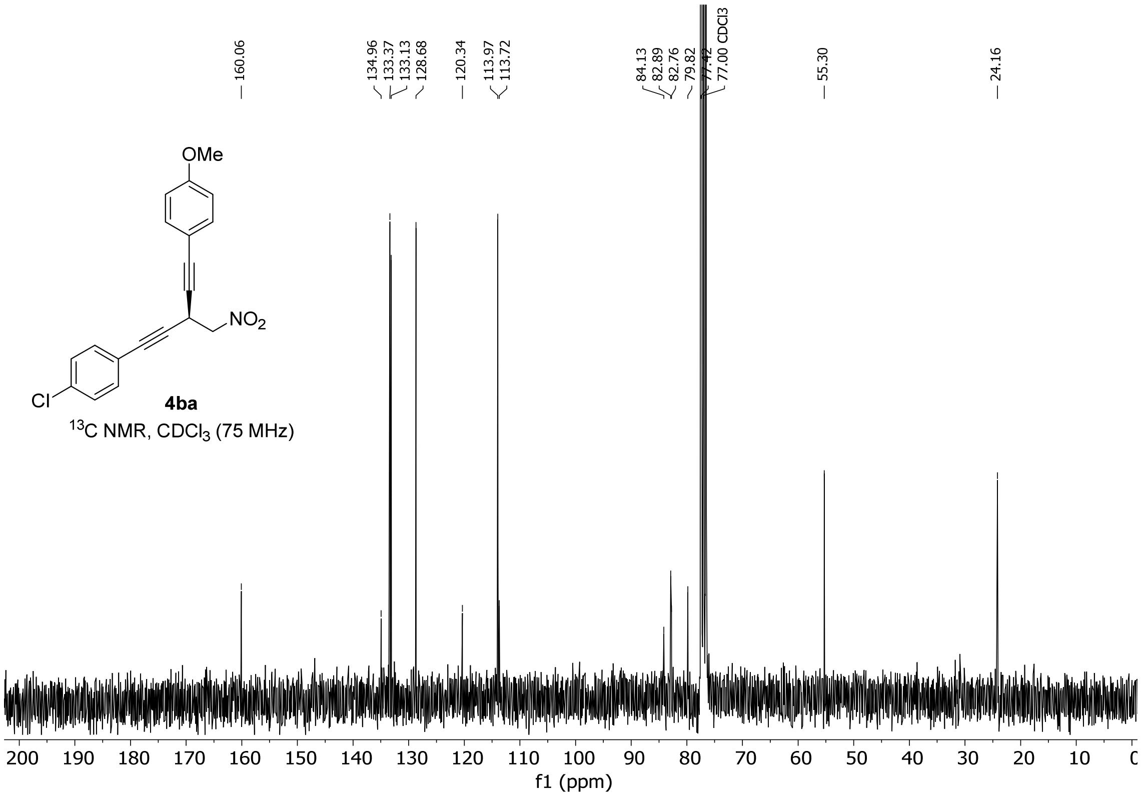
160.06
134.96
133.37
133.13
128.68
120.34
113.97
113.72
84.13
82.89
82.76
79.82
77.42
77.00 CDCl3
55.30
24.16
OMe
NO2
Cl
4ba
13C NMR, CDCl3 (75 MHz)
200 190 180 170 160 150 140 130 120 110 100 90 80 70 60 50 40 30 20 10 0
f1 (ppm)

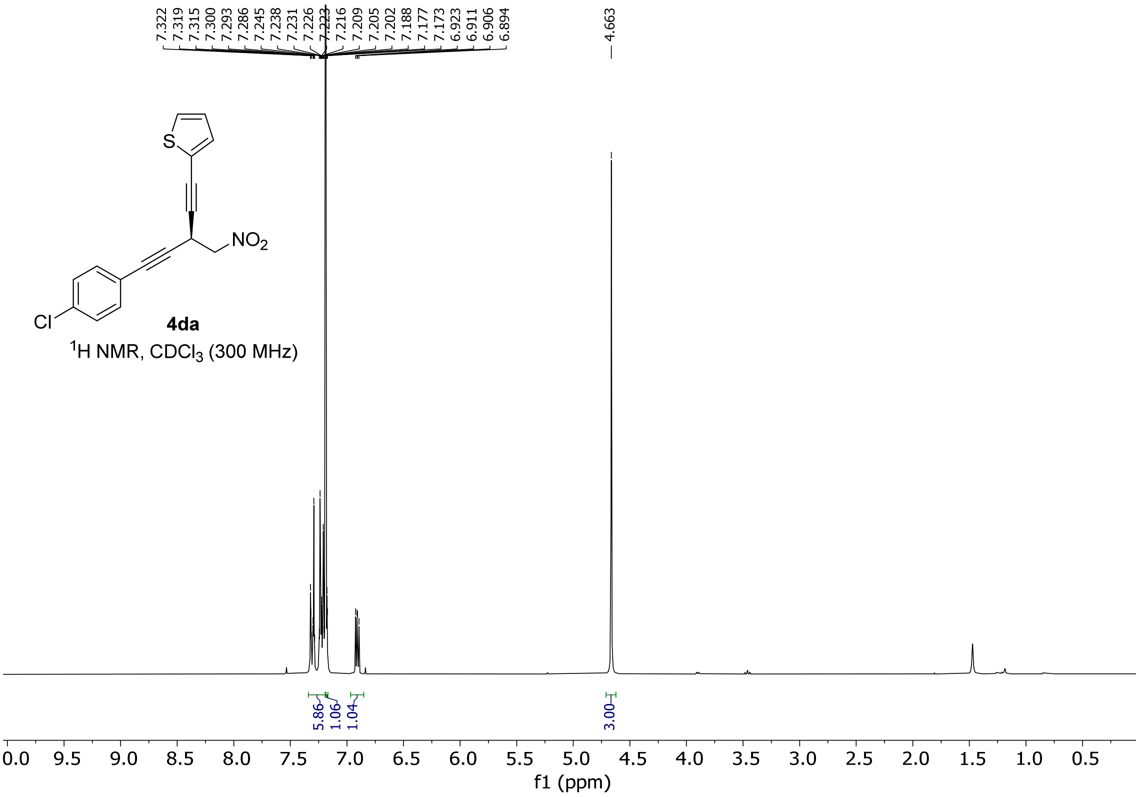
7.322
7.319
7.315
7.300
7.293
7.286
7.245
7.238
7.231
7.226
7.223
7.216
7.209
7.205
7.202
7.188
7.177
7.173
6.923
6.911
6.906
6.894
4.663
S
NO2
Cl
4da
1H NMR, CDCl3 (300 MHz)
5.86
1.06
1.04
3.00
f1 (ppm)

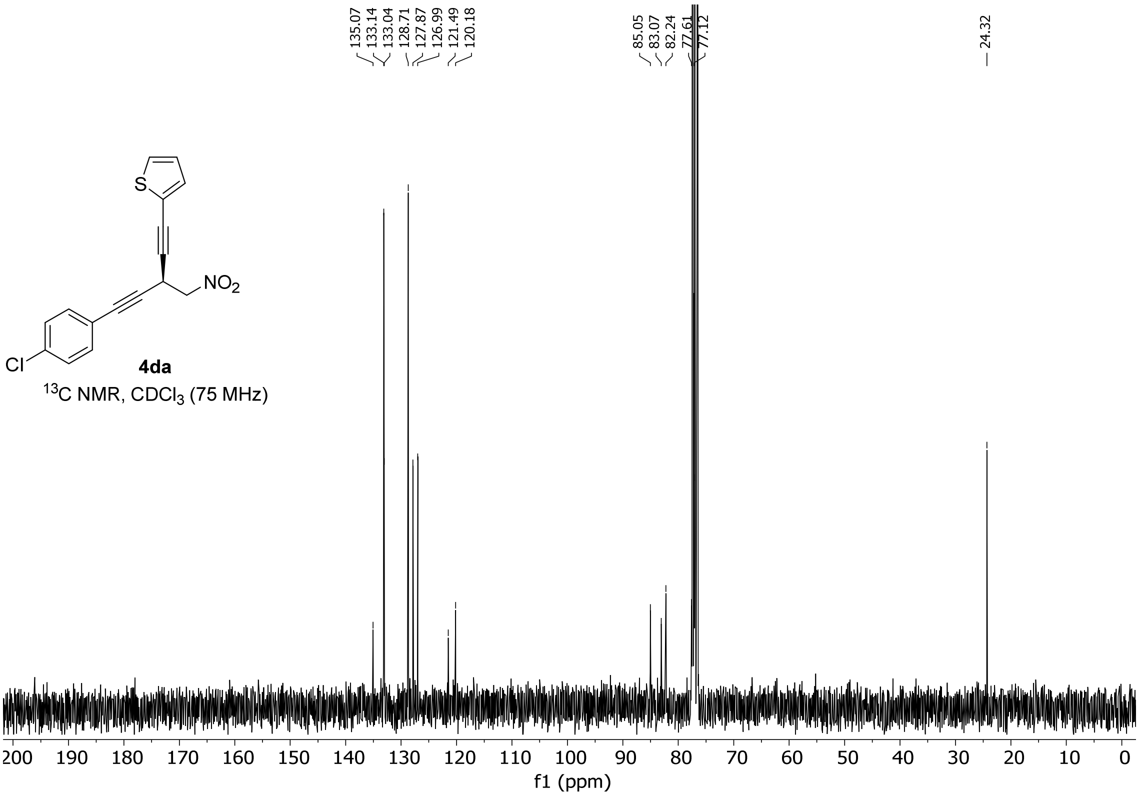
135.07
133.14
133.04
128.71
127.87
126.99
121.49
120.18
85.05
83.07
82.24
77.61
77.12
24.32
S
NO2
Cl
4da
13C NMR, CDCl3 (75 MHz)
f1 (ppm)

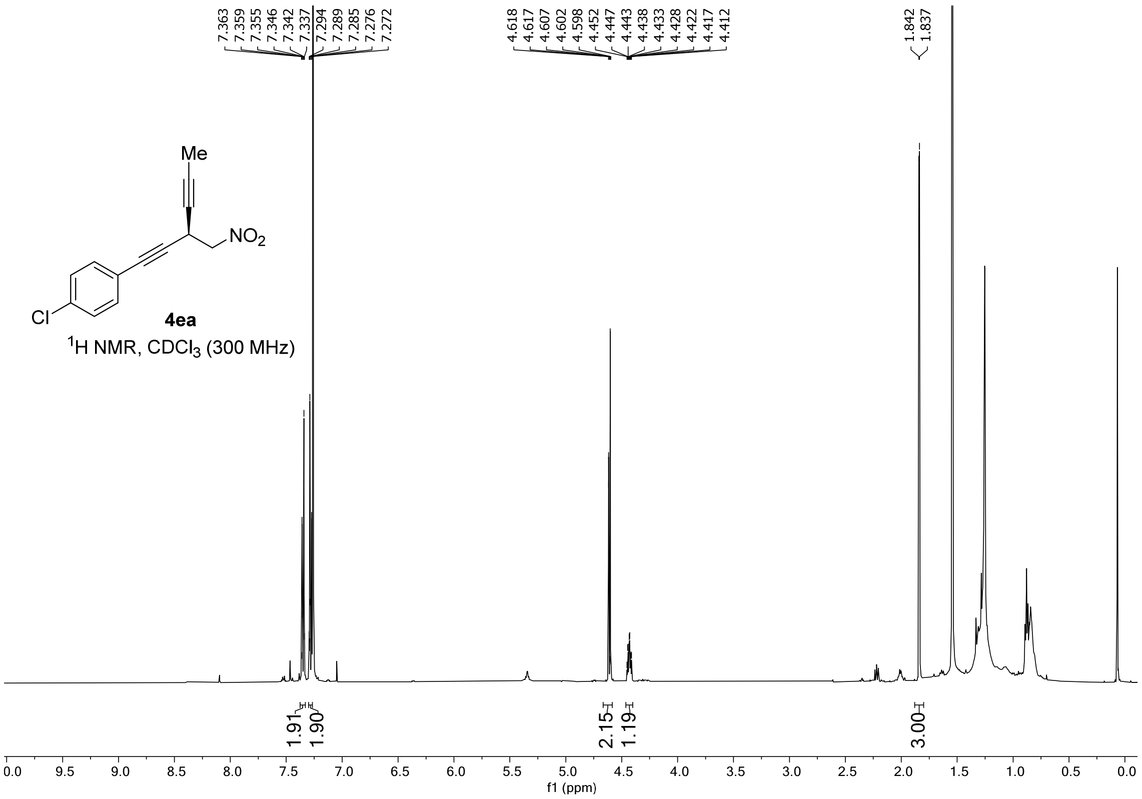

4ea
^{1}H NMR, $CDCl_3$ (300 MHz)

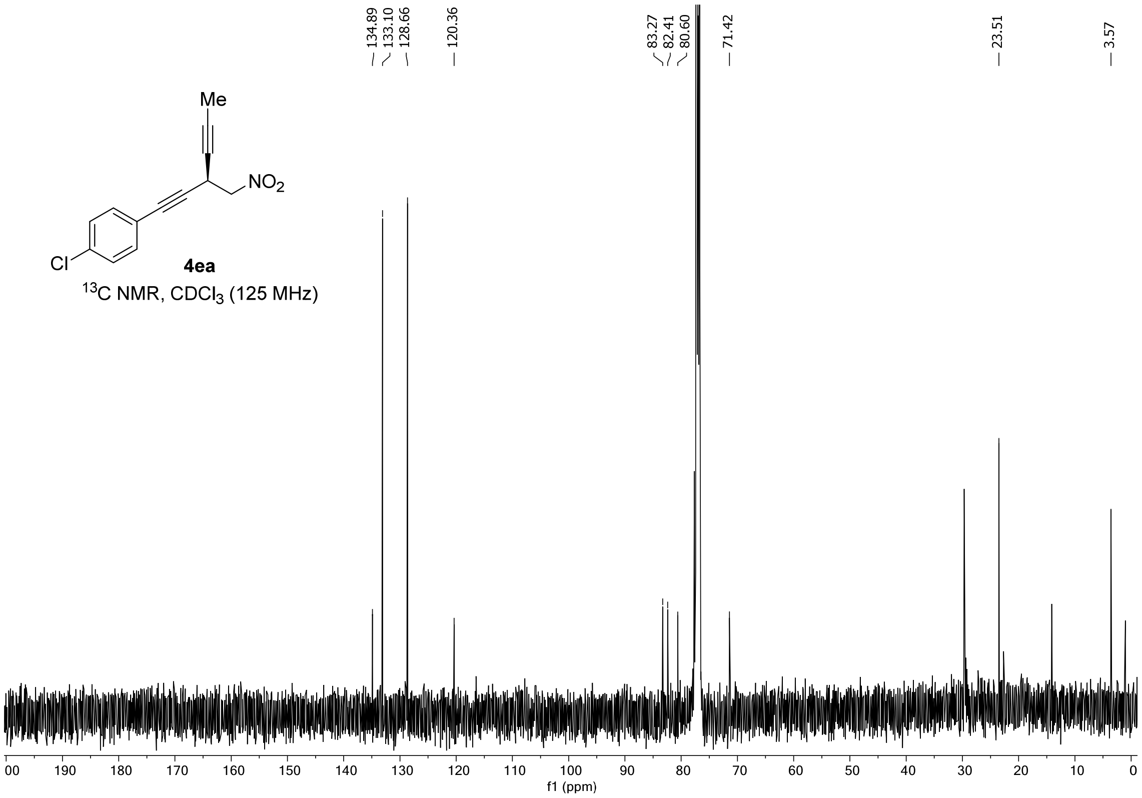

4ea
^{13}C NMR, $CDCl_3$ (125 MHz)

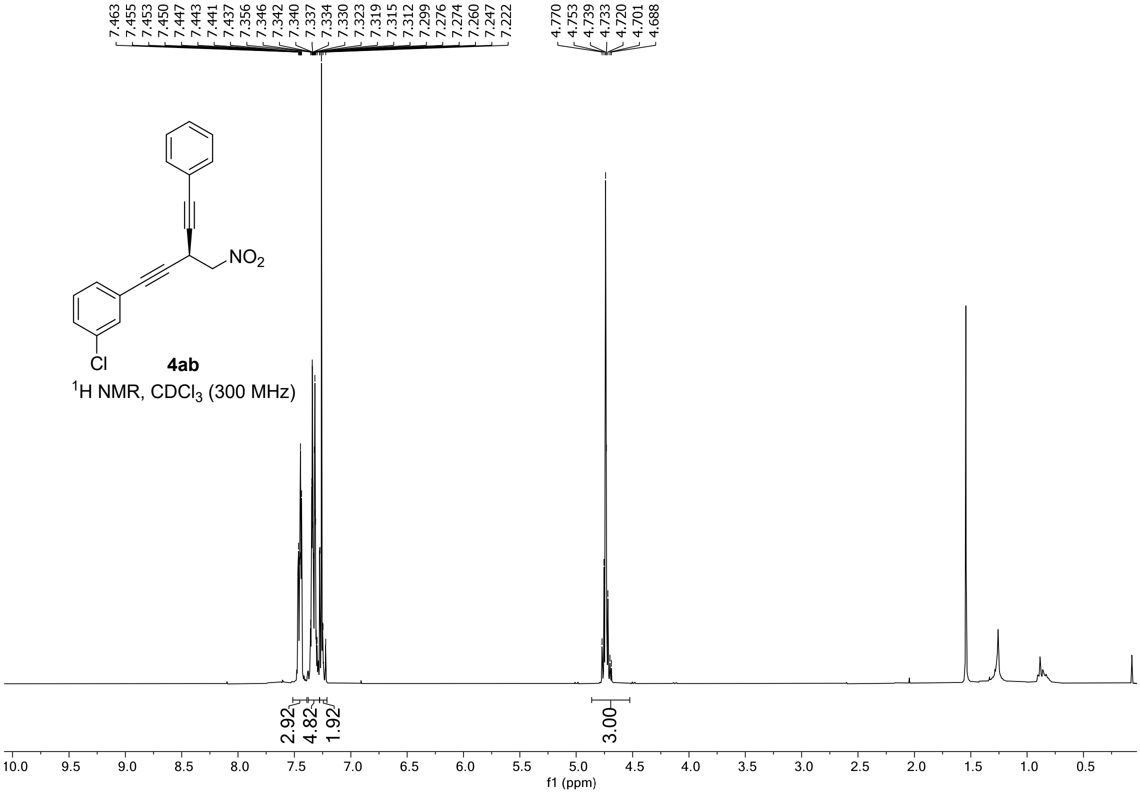
7.463 7.455 7.453 7.450 7.447 7.443 7.441 7.437 7.356 7.346 7.342 7.340 7.337 7.334 7.330 7.323 7.319 7.315 7.312 7.299 7.276 7.274 7.260 7.247 7.222
4.770 4.753 4.739 4.733 4.720 4.701 4.688
NO_2
Cl
4ab
^{1}H NMR, $CDCl_3$ (300 MHz)
2.92
4.82
1.92
3.00
10.0 9.5 9.0 8.5 8.0 7.5 7.0 6.5 6.0 5.5 5.0 4.5 4.0 3.5 3.0 2.5 2.0 1.5 1.0 0.5
f1 (ppm)

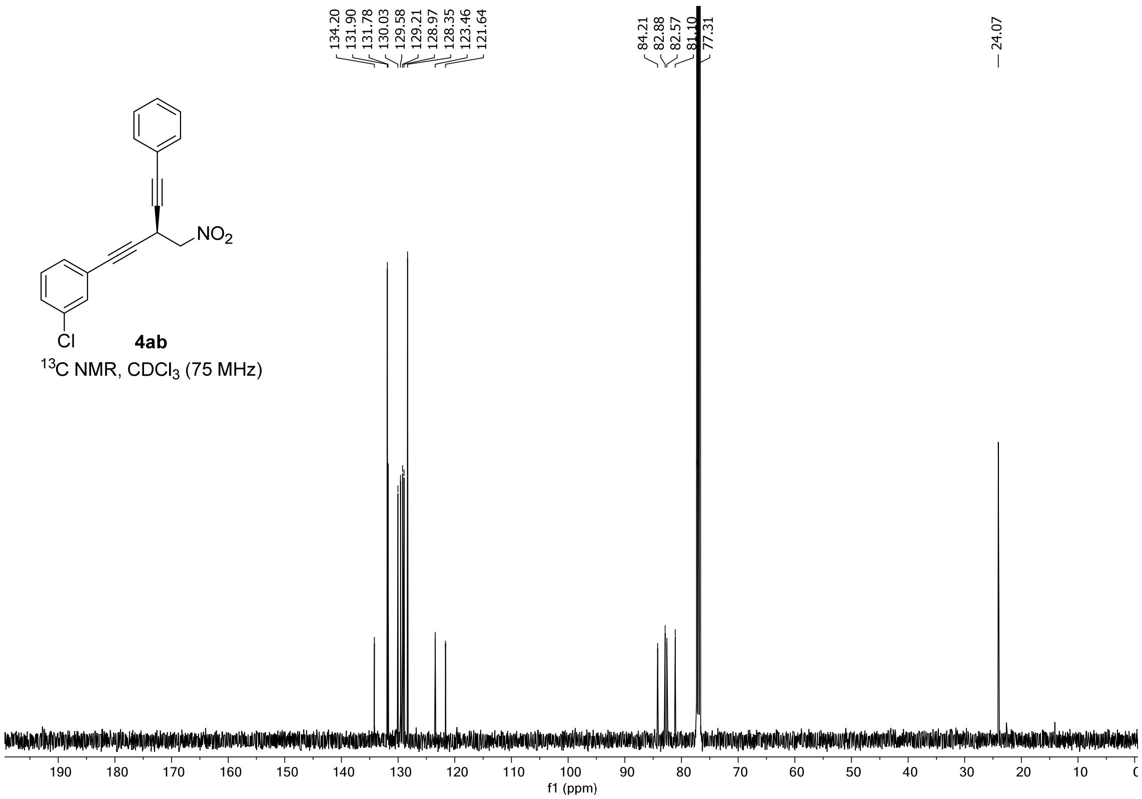
134.20 131.90 131.78 130.03 129.58 129.21 128.97 128.35 123.46 121.64
84.21 82.88 82.57 81.10 77.31
24.07
NO_2
Cl
4ab
^{13}C NMR, $CDCl_3$ (75 MHz)
190 180 170 160 150 140 130 120 110 100 90 80 70 60 50 40 30 20 10
f1 (ppm)

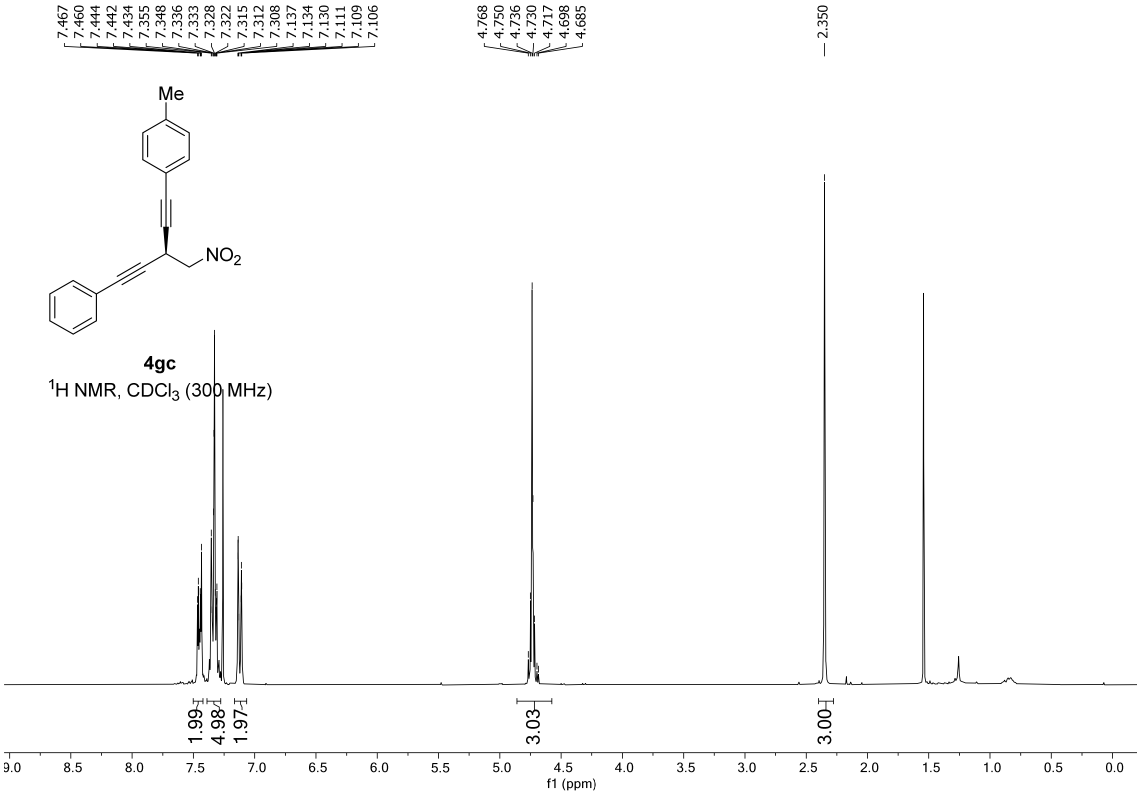
7.467
7.460
7.444
7.442
7.434
7.355
7.348
7.336
7.333
7.328
7.322
7.315
7.312
7.308
7.137
7.134
7.130
7.111
7.109
7.106
4.768
4.750
4.736
4.730
4.717
4.698
4.685
2.350
Me
NO2
4gc
1H NMR, CDCl3 (300 MHz)
1.99
4.98
1.97
3.03
3.00
f1 (ppm)

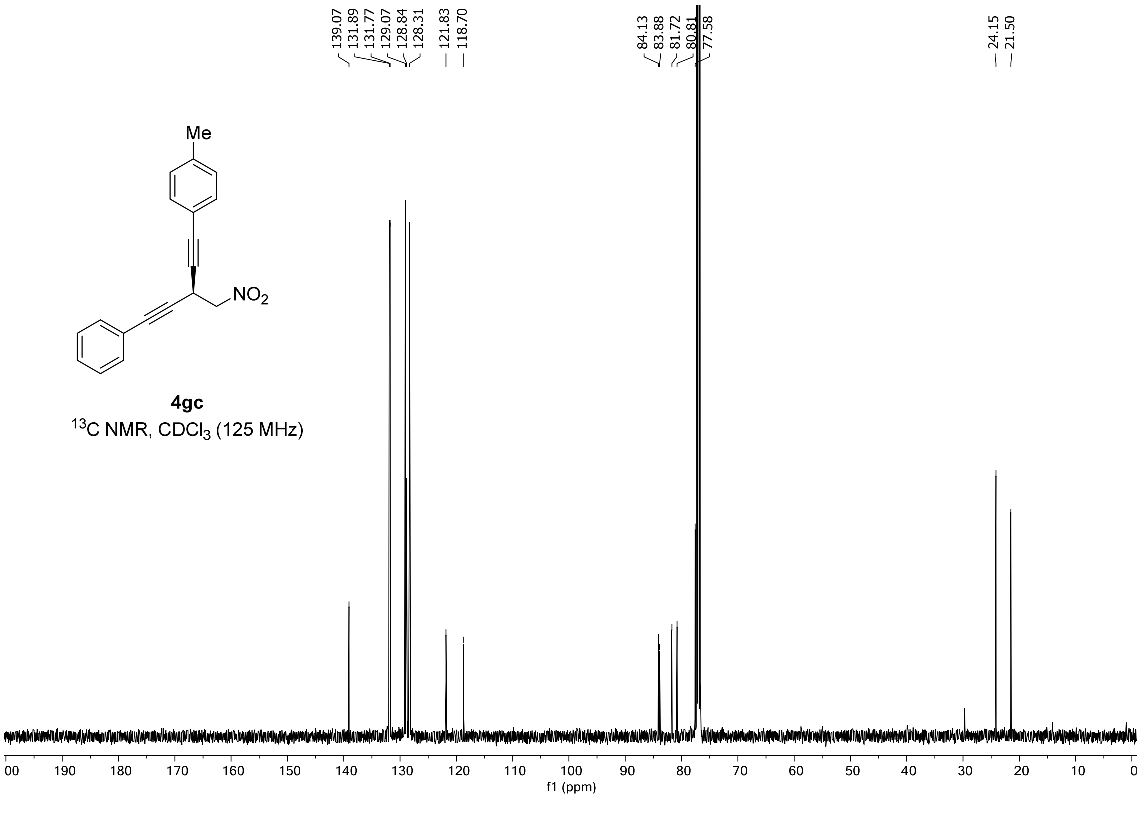
139.07
131.89
131.77
129.07
128.84
128.31
121.83
118.70
84.13
83.88
81.72
80.81
77.58
24.15
21.50
Me
NO2
4gc
13C NMR, CDCl3 (125 MHz)
f1 (ppm)

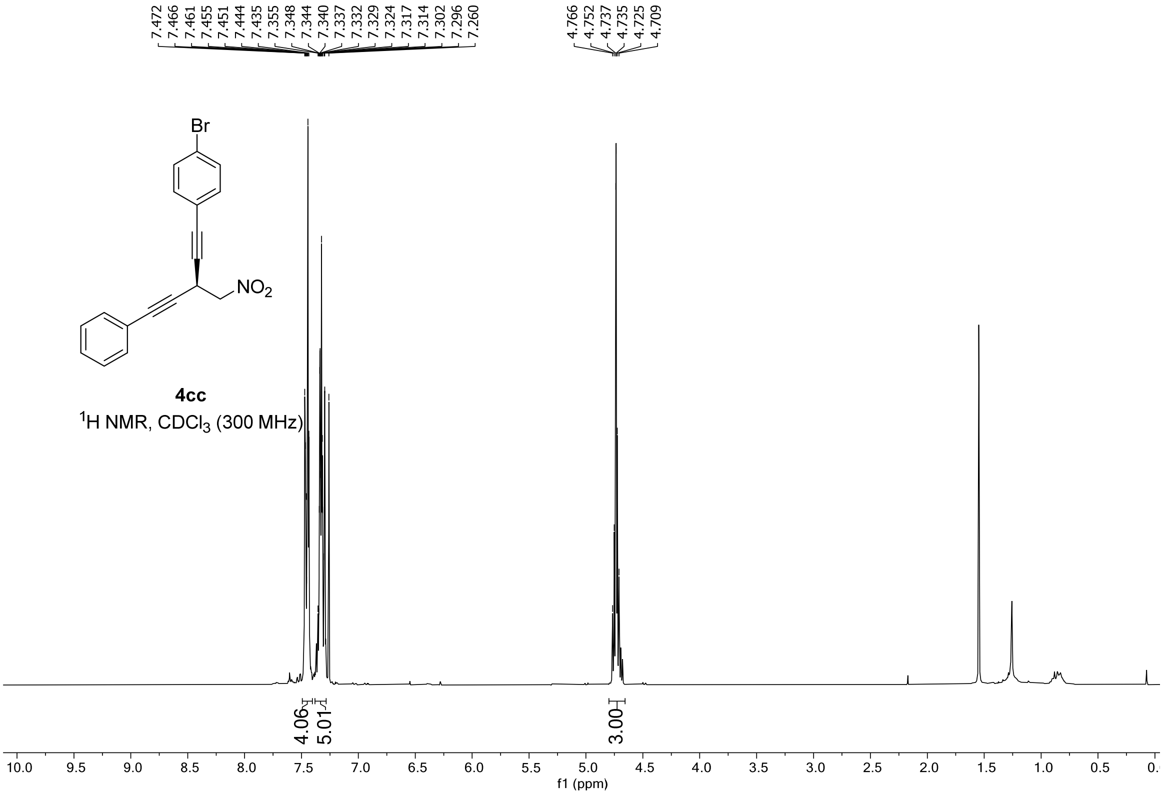
7.472
7.466
7.461
7.455
7.451
7.444
7.435
7.355
7.348
7.344
7.340
7.337
7.332
7.329
7.324
7.317
7.314
7.302
7.296
7.260
4.766
4.752
4.737
4.735
4.725
4.709
Br
NO2
4cc
1H NMR, CDCl3 (300 MHz)
4.06
5.01
3.00
f1 (ppm)

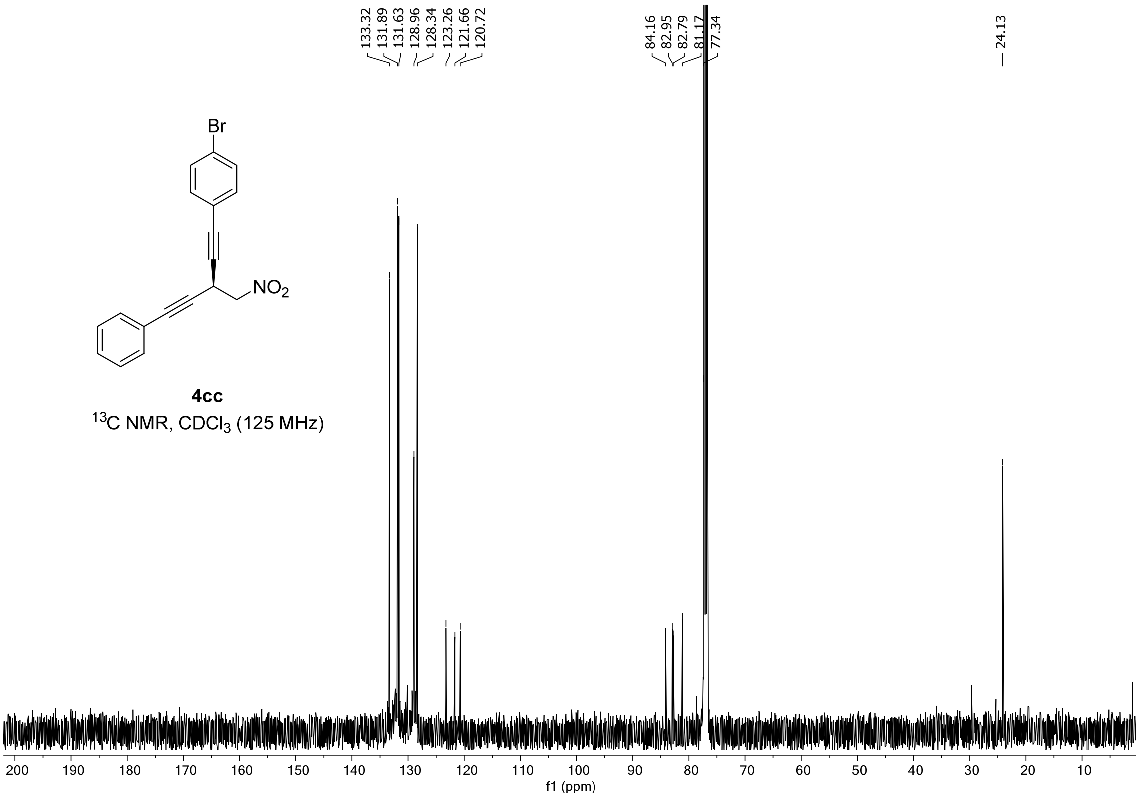
133.32
131.89
131.63
128.96
128.34
123.26
121.66
120.72
84.16
82.95
82.79
81.17
77.34
24.13
Br
NO2
4cc
13C NMR, CDCl3 (125 MHz)
f1 (ppm)

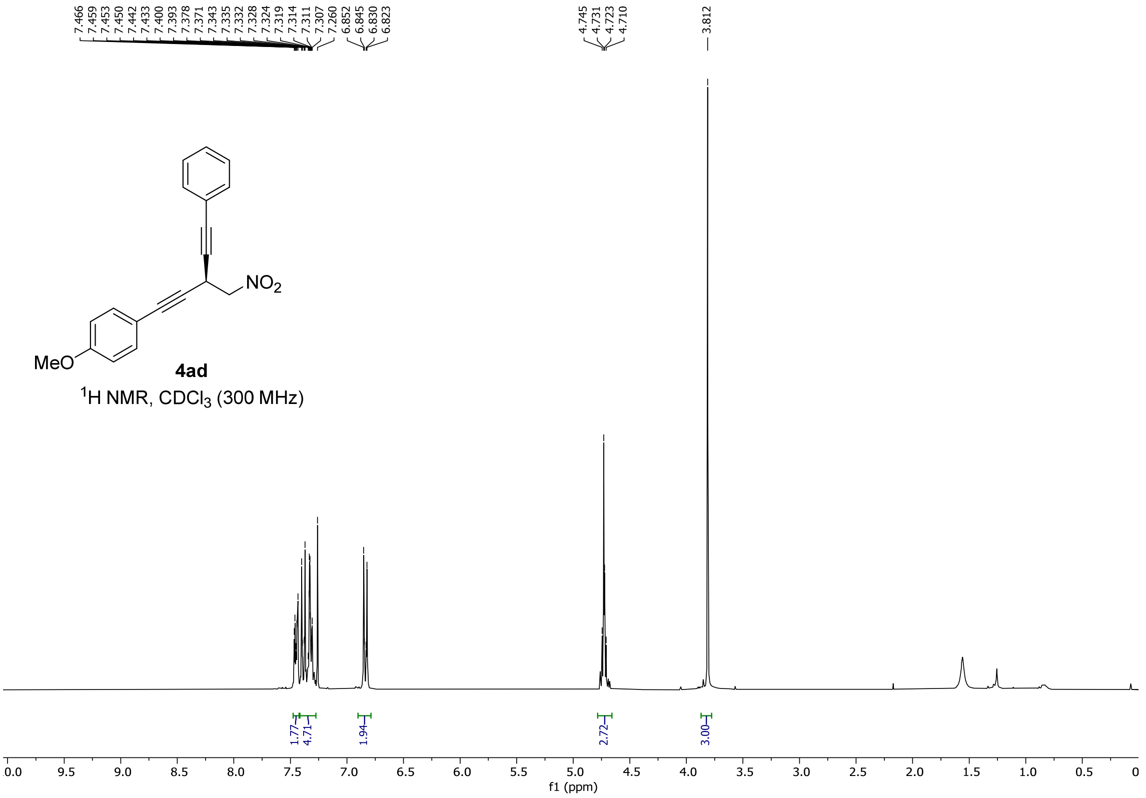
7.466
7.459
7.453
7.450
7.442
7.433
7.400
7.393
7.378
7.371
7.343
7.335
7.332
7.328
7.324
7.319
7.314
7.311
7.307
7.260
6.852
6.845
6.830
6.823
4.745
4.731
4.723
4.710
3.812
NO2
MeO
4ad
1H NMR, CDCl3 (300 MHz)
1.77
4.71
1.94
2.72
3.00
f1 (ppm)

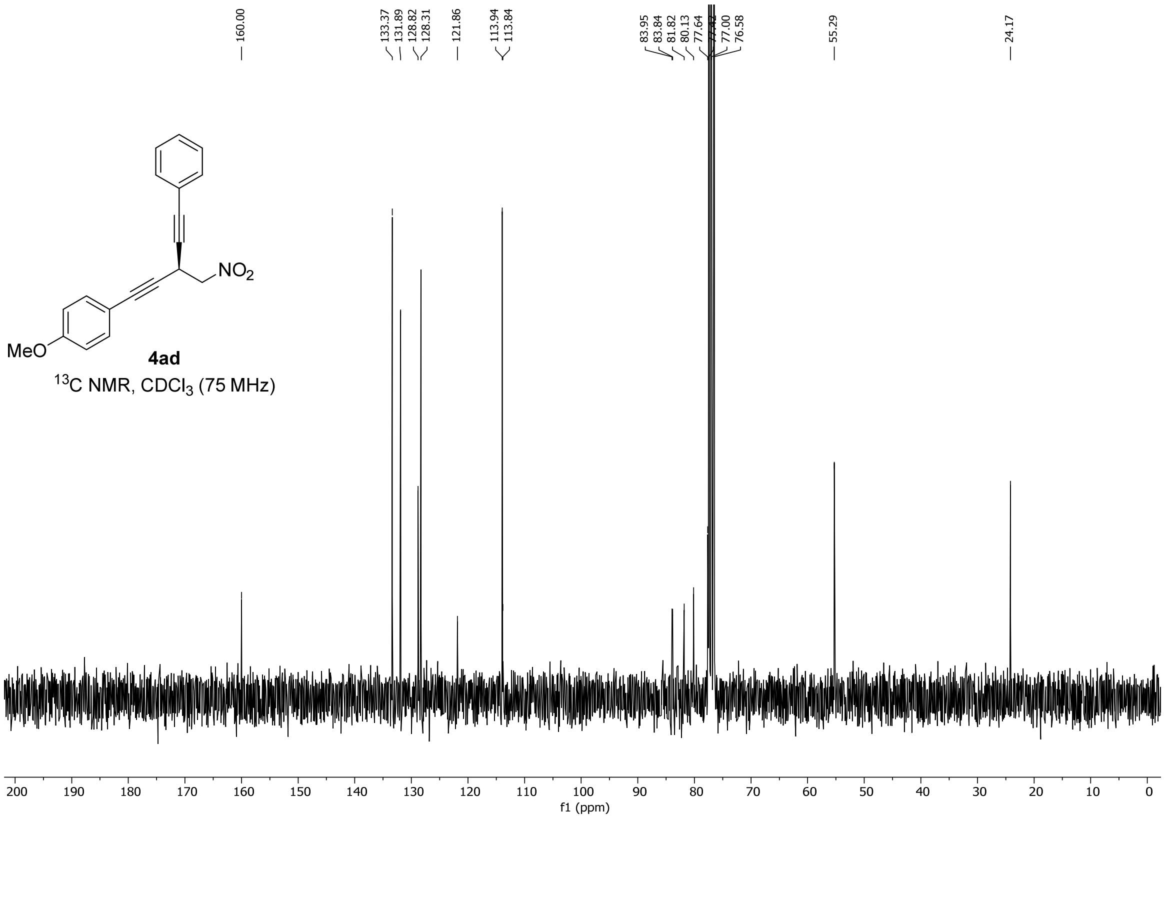
160.00
133.37
131.89
128.82
128.31
121.86
113.94
113.84
83.95
83.84
81.82
80.13
77.64
77.00
76.58
55.29
24.17
NO2
MeO
4ad
13C NMR, CDCl3 (75 MHz)
f1 (ppm)

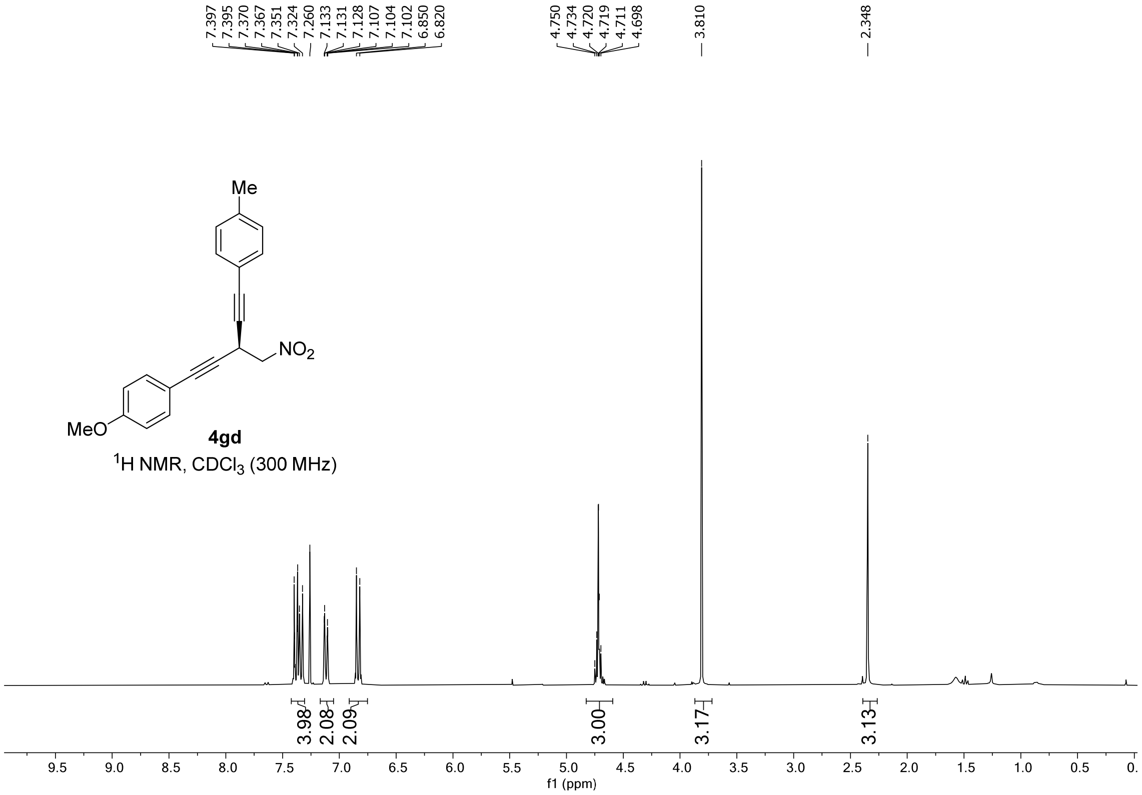

Me
NO2
MeO
4gd
1H NMR, CDCl3 (300 MHz)
7.397 7.395 7.370 7.367 7.351 7.324 7.260 7.133 7.131 7.128 7.107 7.104 7.102 6.850 6.820
4.750 4.734 4.720 4.719 4.711 4.698
3.810
2.348
3.98 2.08 2.09 3.00 3.17 3.13
f1 (ppm)

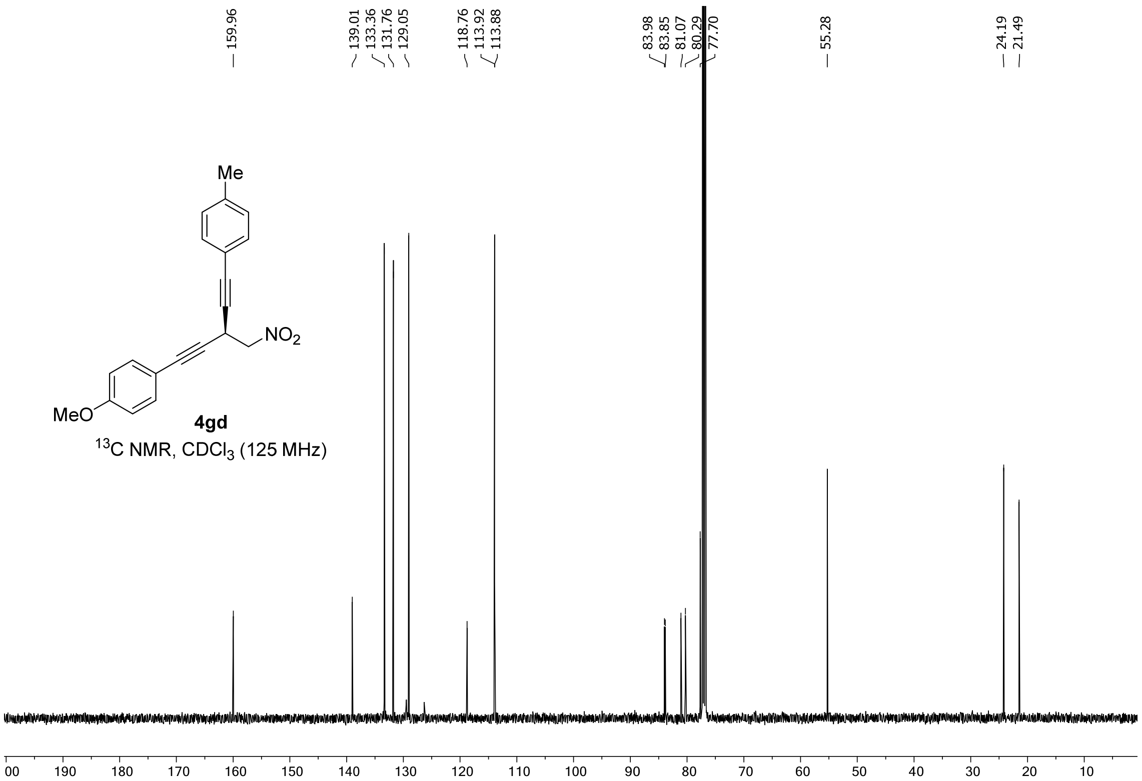

Me
NO2
MeO
4gd
13C NMR, CDCl3 (125 MHz)
159.96
139.01 133.36 131.76 129.05
118.76 113.92 113.88
83.98 83.85 81.07 80.29 77.70
55.28
24.19 21.49
f1 (ppm)

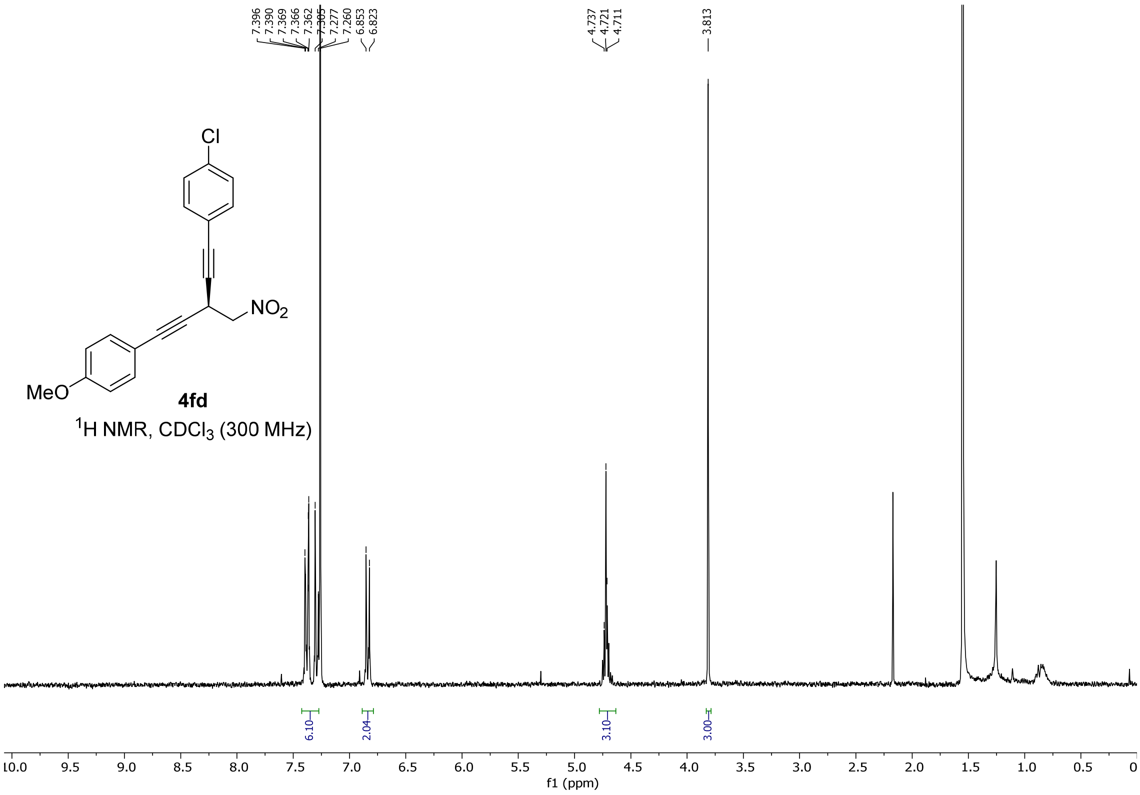

4fd

^{1}H NMR, $CDCl_3$ (300 MHz)

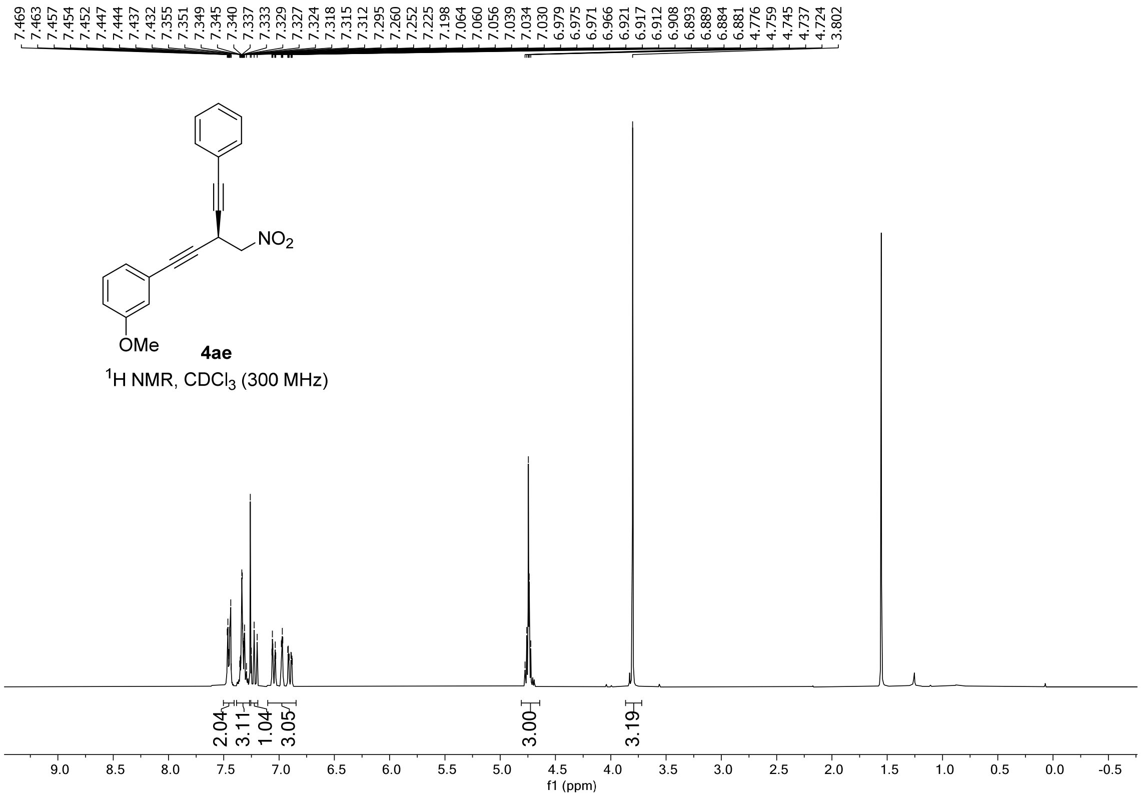
7.469 7.463 7.457 7.454 7.452 7.447 7.444 7.437 7.432 7.355 7.351 7.349 7.345 7.340 7.337 7.333 7.329 7.327 7.324 7.318 7.315 7.312 7.295 7.260 7.252 7.225 7.198 7.064 7.060 7.056 7.039 7.034 7.030 6.979 6.975 6.971 6.966 6.921 6.917 6.912 6.908 6.893 6.889 6.884 6.881 4.776 4.759 4.745 4.737 4.724 3.802
NO2
OMe
4ae
1H NMR, CDCl3 (300 MHz)
2.04
3.11
1.04
3.05
3.00
3.19
f1 (ppm)

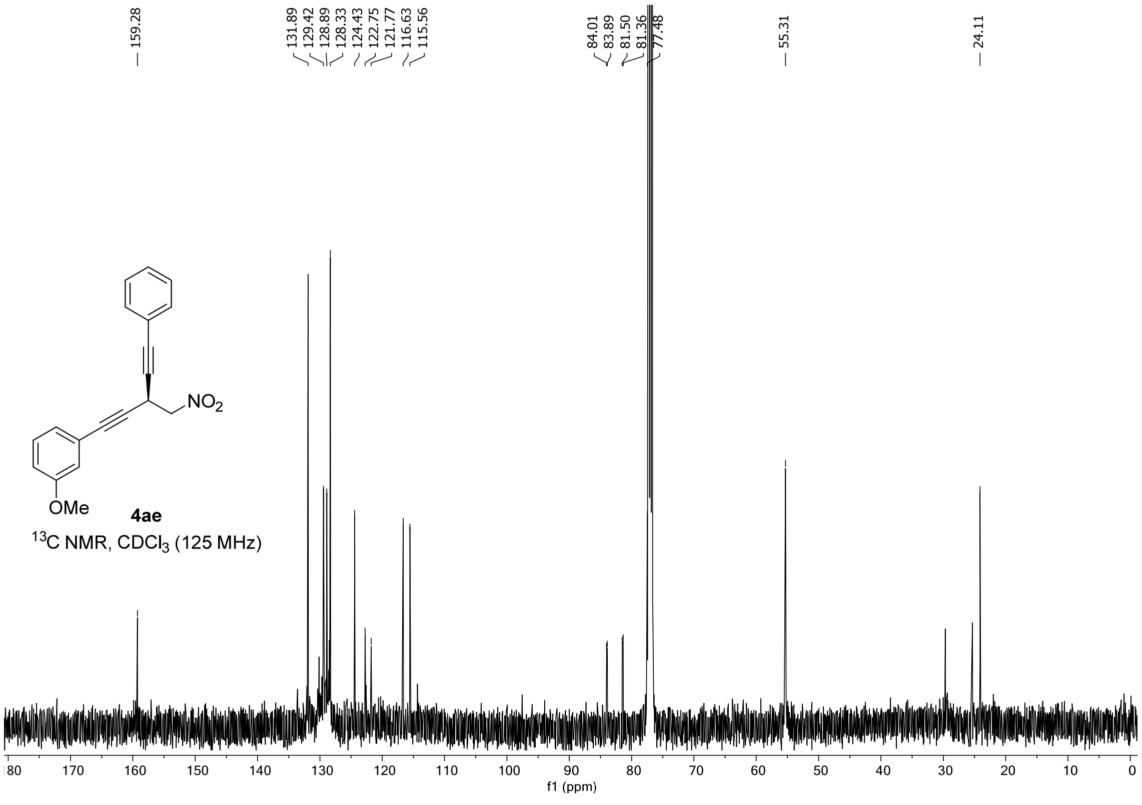
159.28
131.89 129.42 128.89 128.33 124.43 122.75 121.77 116.63 115.56
84.01 83.89 81.50 81.36 77.48
55.31
24.11
NO2
OMe
4ae
13C NMR, CDCl3 (125 MHz)
f1 (ppm)

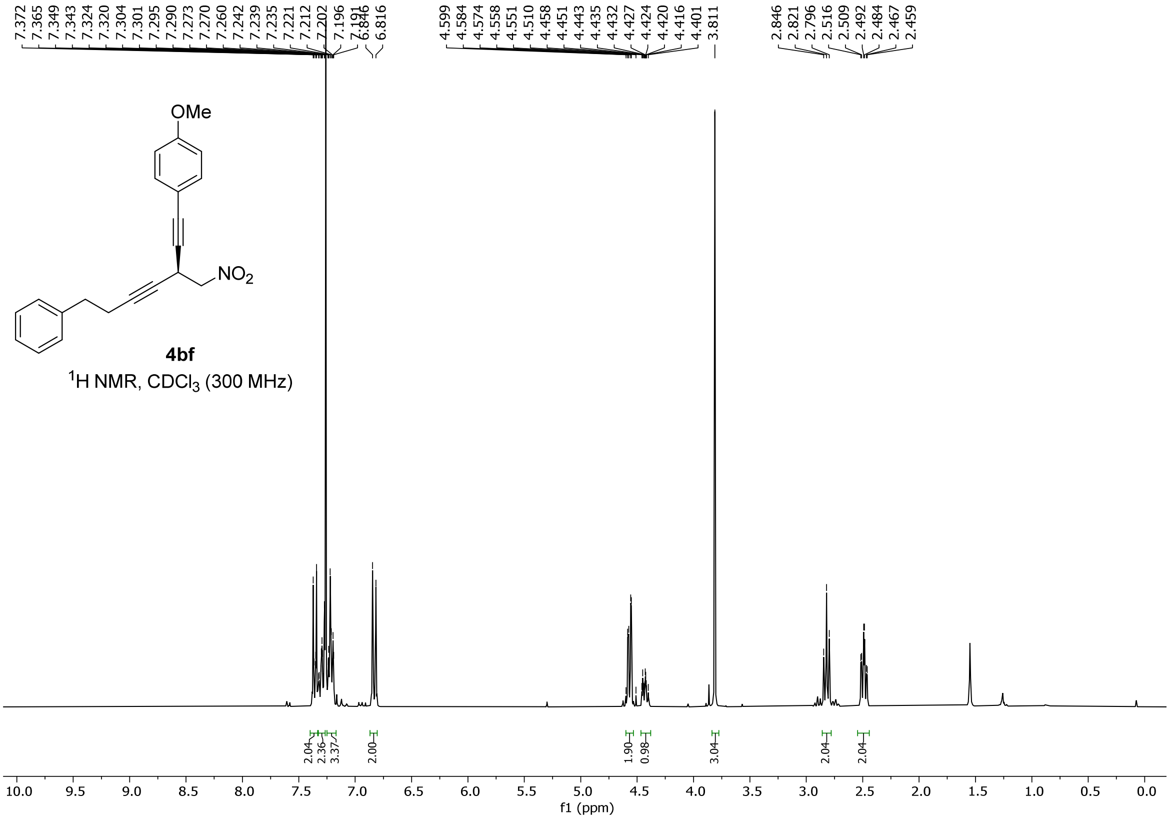
7.372
7.365
7.349
7.343
7.324
7.320
7.304
7.301
7.295
7.290
7.273
7.270
7.260
7.242
7.239
7.235
7.221
7.212
7.202
7.196
7.191
6.846
6.816
4.599
4.584
4.574
4.558
4.551
4.510
4.458
4.451
4.443
4.435
4.432
4.427
4.424
4.420
4.416
4.401
3.811
2.846
2.821
2.796
2.516
2.509
2.492
2.484
2.467
2.459
OMe
NO2
4bf
1H NMR, CDCl3 (300 MHz)
2.04
2.36
3.37
2.00
1.90
0.98
3.04
2.04
2.04
10.0
9.5
9.0
8.5
8.0
7.5
7.0
6.5
6.0
5.5
5.0
4.5
4.0
3.5
3.0
2.5
2.0
1.5
1.0
0.5
0.0
f1 (ppm)

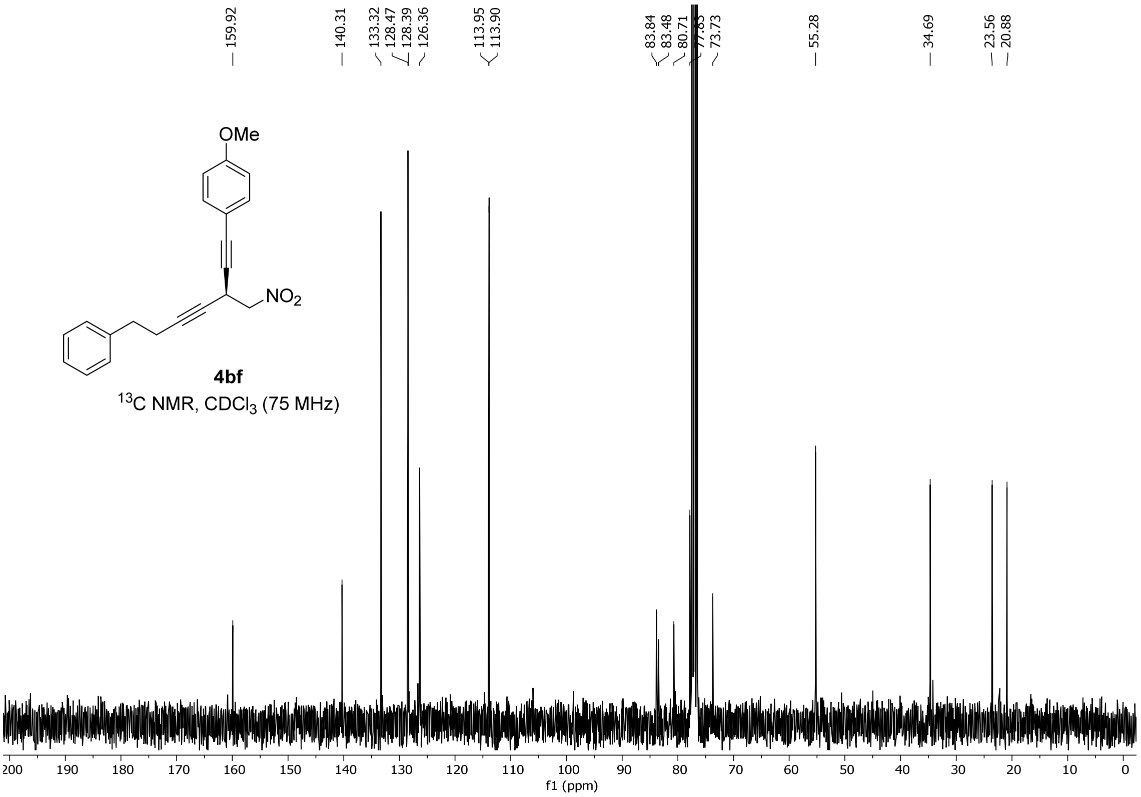
159.92
140.31
133.32
128.47
128.39
126.36
113.95
113.90
83.84
83.48
80.71
77.85
73.73
55.28
34.69
23.56
20.88
OMe
NO2
4bf
13C NMR, CDCl3 (75 MHz)
200
190
180
170
160
150
140
130
120
110
100
90
80
70
60
50
40
30
20
10
0
f1 (ppm)

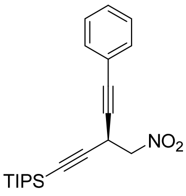

4ah

^{1}H NMR, $CDCl_3$ (300 MHz)

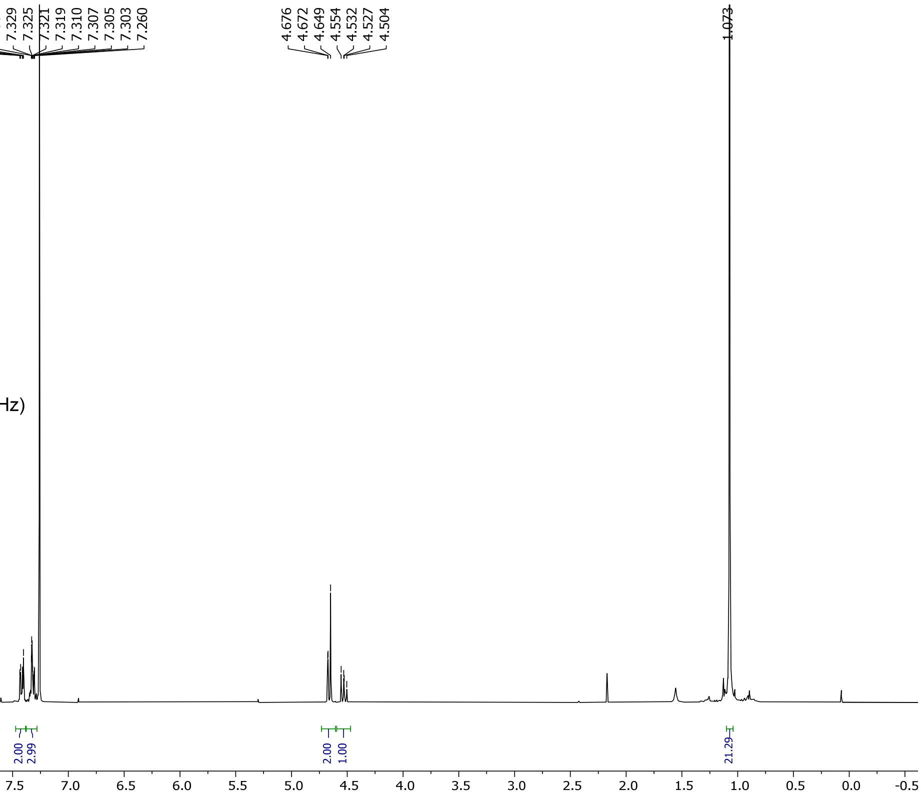

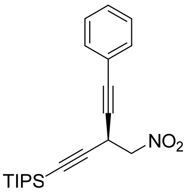

4ah

^{13}C NMR, $CDCl_3$ (75 MHz)

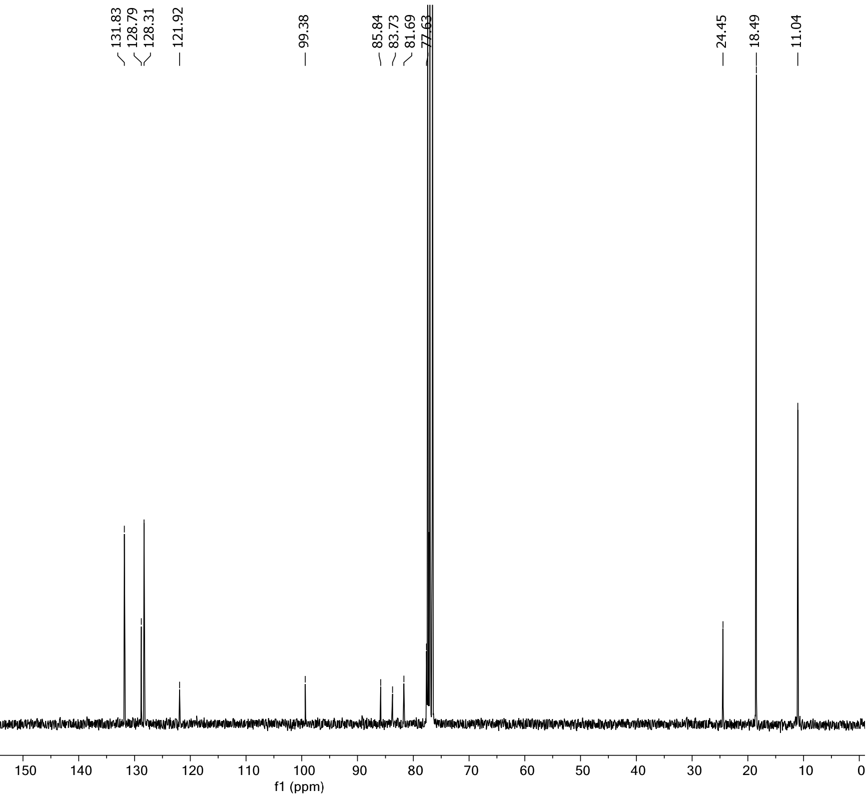

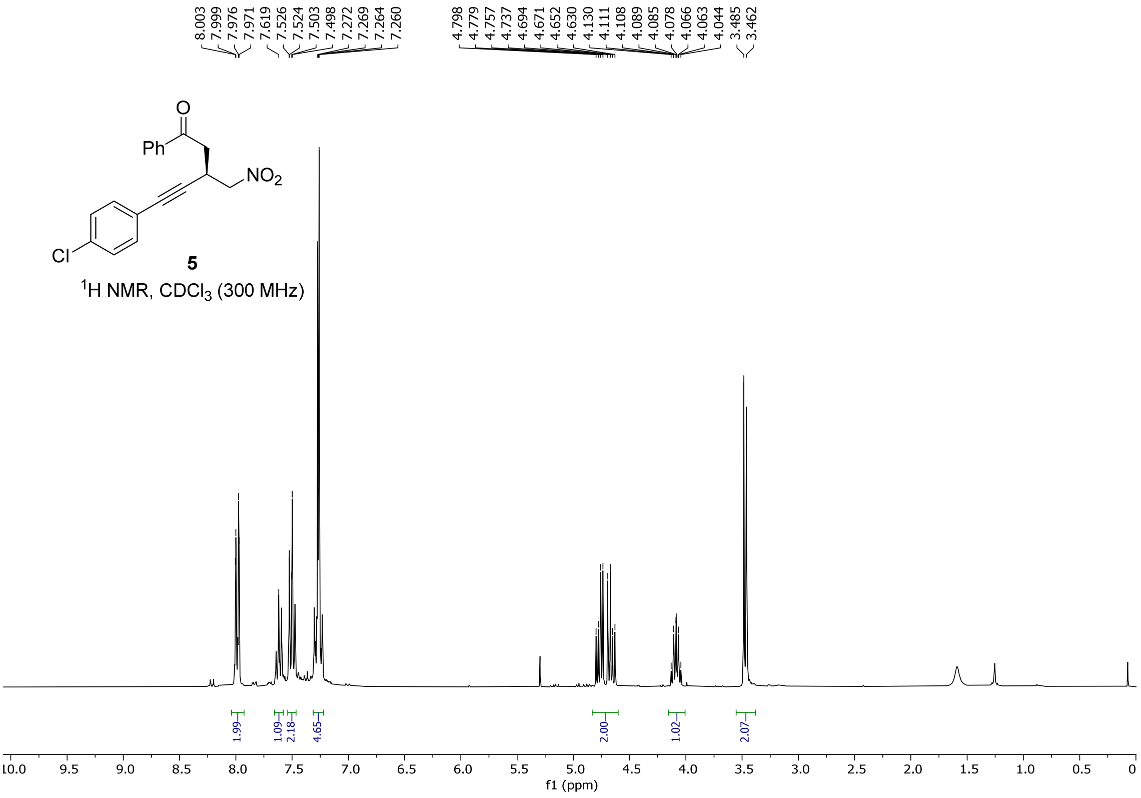

5

^{1}H NMR, $CDCl_3$ (300 MHz)

HPLC Traces

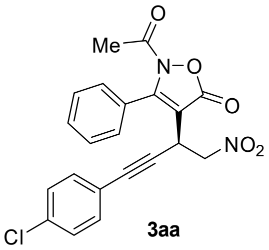

3aa

Non-enantioselective reaction

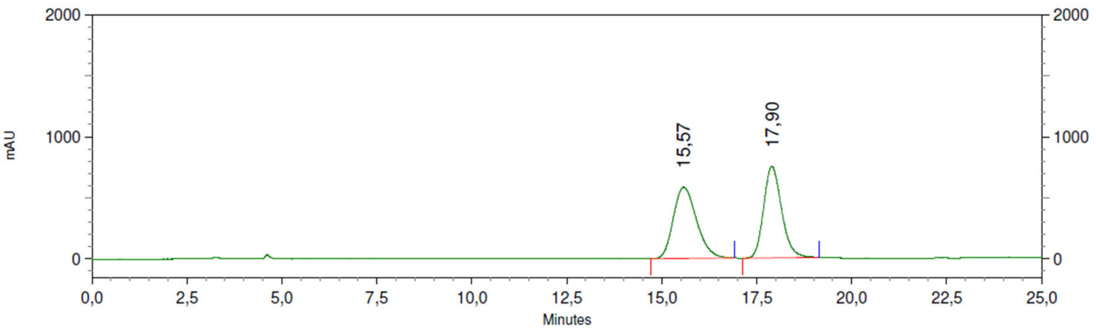

31: 250 nm, 4 nm
Results

Retention Time	Area	Area Percent
15,57	99526914	50,262
17,90	98487475	49,738

Enantioselective reaction

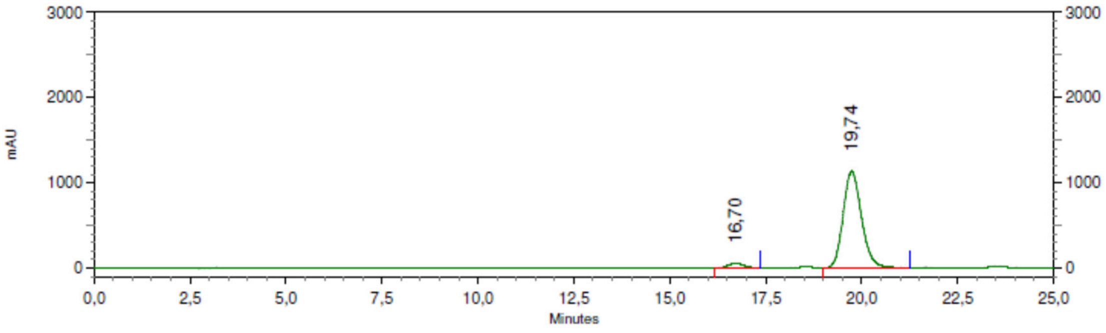

31: 250 nm, 4 nm
Results

Retention Time	Area	Area Percent
16,70	6241604	4,000
19,74	149794597	96,000

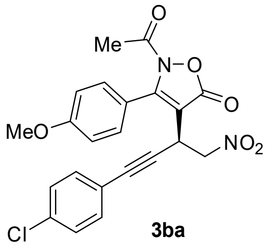

3ba

Non-enantioselective reaction

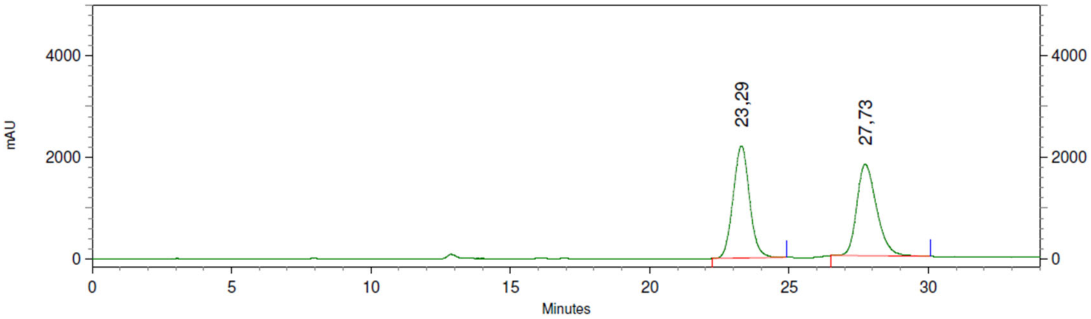

32: 253 nm, 4 nm
Results

Retention Time	Area	Area Percent
23,29	361862273	50,372
27,73	356524599	49,628

Enantioselective reaction

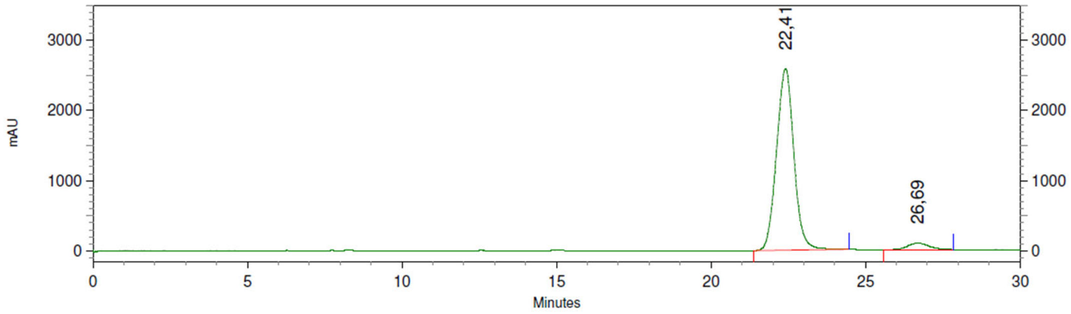

31: 250 nm, 4 nm
Results

Retention Time	Area	Area Percent
22,41	404609995	95,913
26,69	17240141	4,087

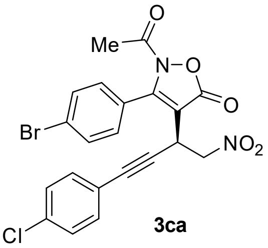

3ca

Non-enantioselective reaction

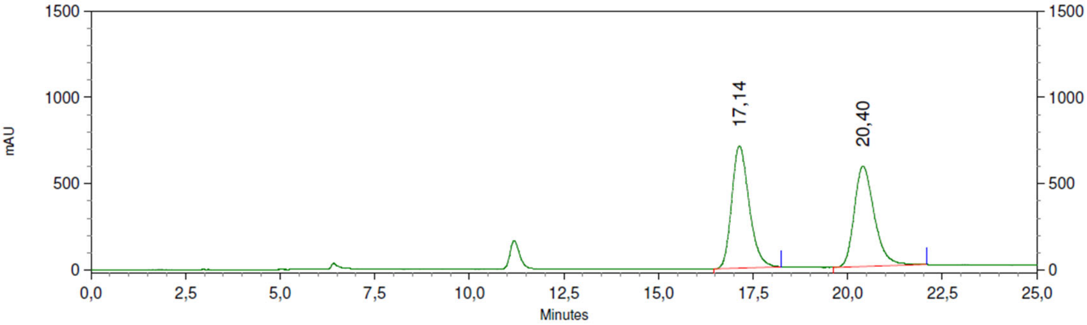

31: 250 nm, 4 nm
Results

Retention Time	Area	Area Percent
17,14	91291437	51,421
20,40	86247401	48,579

Enantioselective reaction

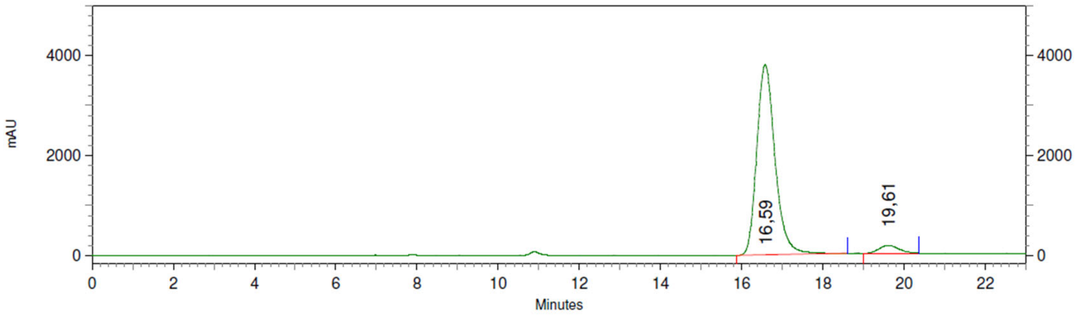

31: 250 nm, 4 nm
Results

Retention Time	Area	Area Percent
16,59	476448865	95,857
19,61	20591790	4,143

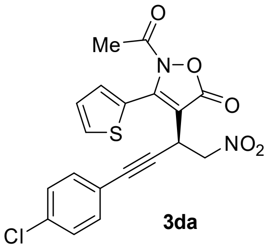

3da

Non-enantioselective reaction

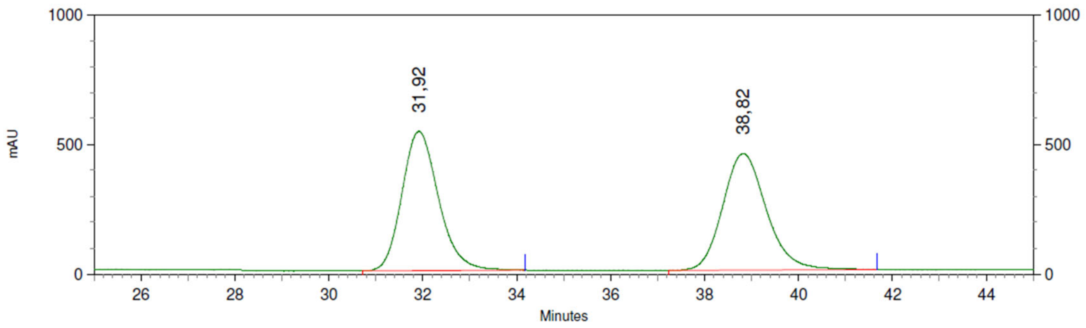

30: 248 nm, 4 nm
Results

Retention Time	Area	Area Percent
31,92	116440082	50,032
38,82	116291026	49,968

Enantioselective reaction

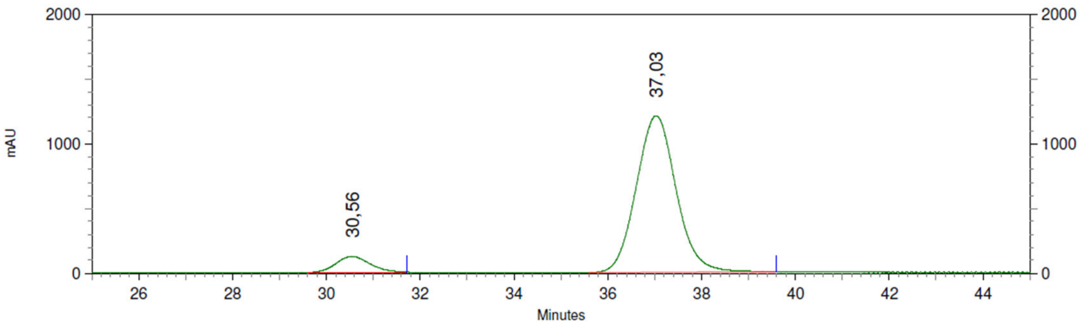

34: 250 nm, 4 nm
Results

Retention Time	Area	Area Percent
30,56	24440557	7,890
37,03	285334706	92,110

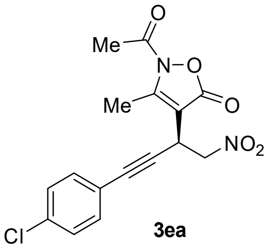

3ea

Non-enantioselective reaction

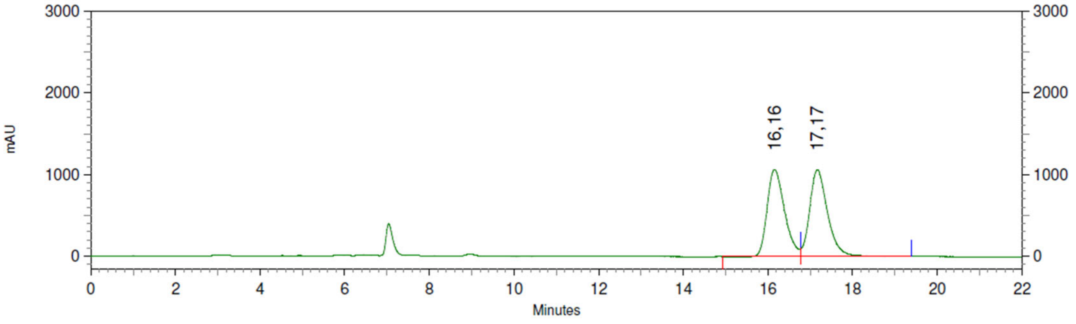

32: 253 nm, 4 nm
Results

Retention Time	Area	Area Percent
16,16	121259282	49,299
17,17	124706500	50,701

Enantioselective reaction

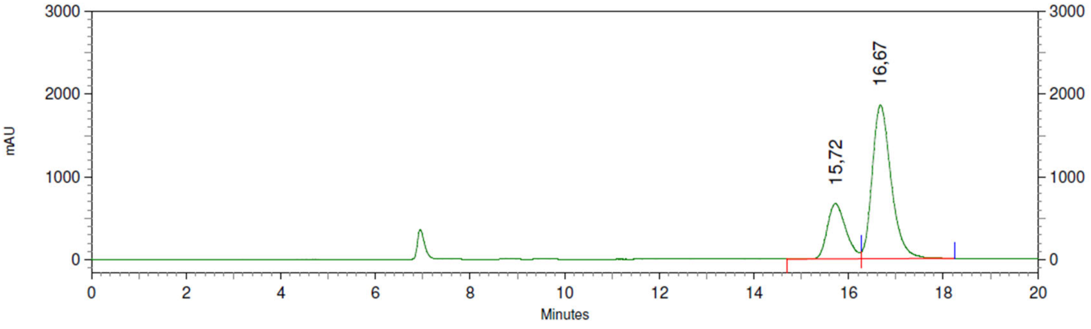

32: 253 nm, 4 nm
Results

Retention Time	Area	Area Percent
15,72	72493597	25,982
16,67	206522883	74,018

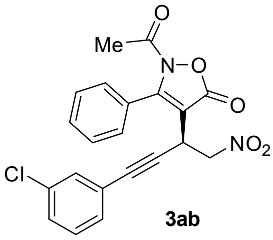

3ab

Non-enantioselective reaction

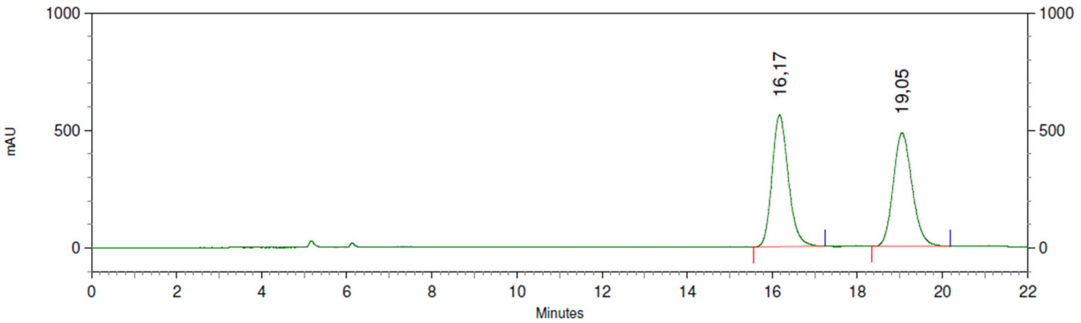

34: 292 nm, 4 nm
Results

Retention Time	Area	Area Percent
16,17	59942412	50,143
19,05	59601500	49,857

Enantioselective reaction

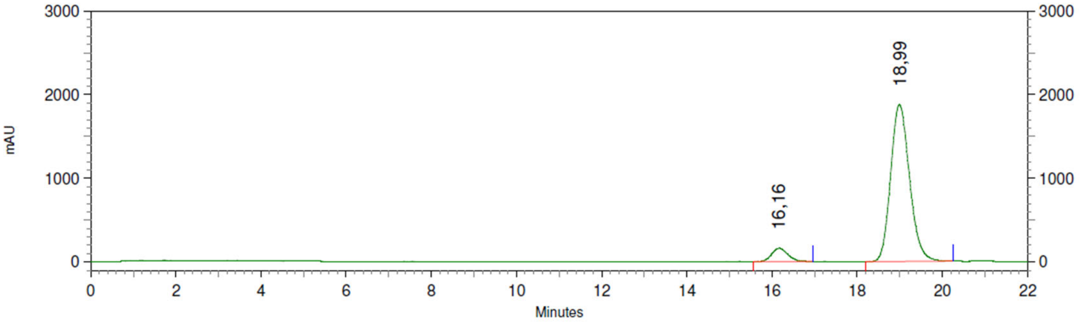

34: 292 nm, 4 nm
Results

Retention Time	Area	Area Percent
16,16	16806302	6,779
18,99	231093923	93,221

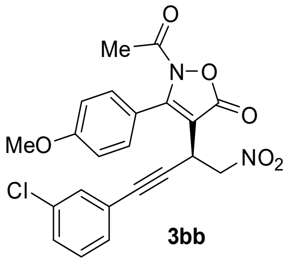

3bb

Non-enantioselective reaction

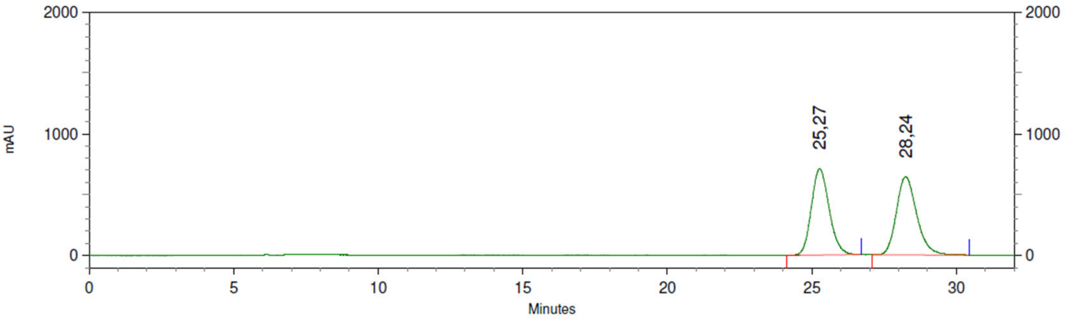

34: 292 nm, 4 nm
Results

Retention Time	Area	Area Percent
25,27	121721231	49,222
28,24	125571039	50,778

Enantioselective reaction

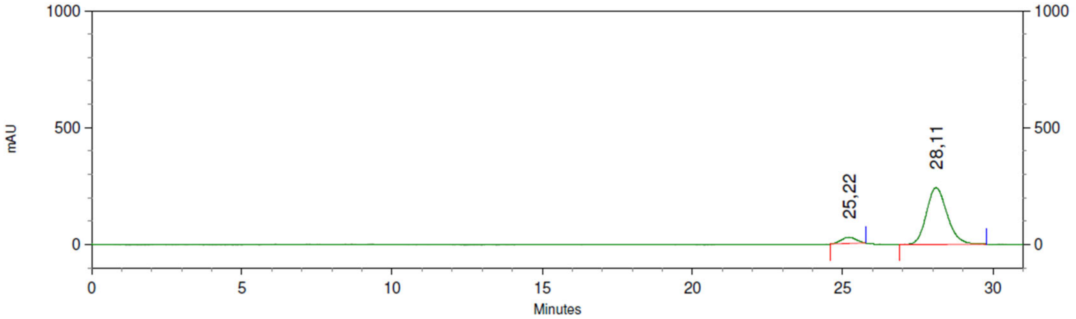

34: 292 nm, 4 nm
Results

Retention Time	Area	Area Percent
25,22	3832859	7,585
28,11	46701459	92,415

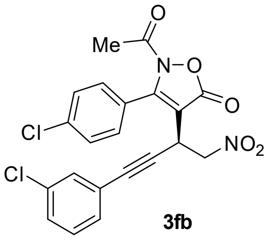

3fb

Non-enantioselective reaction

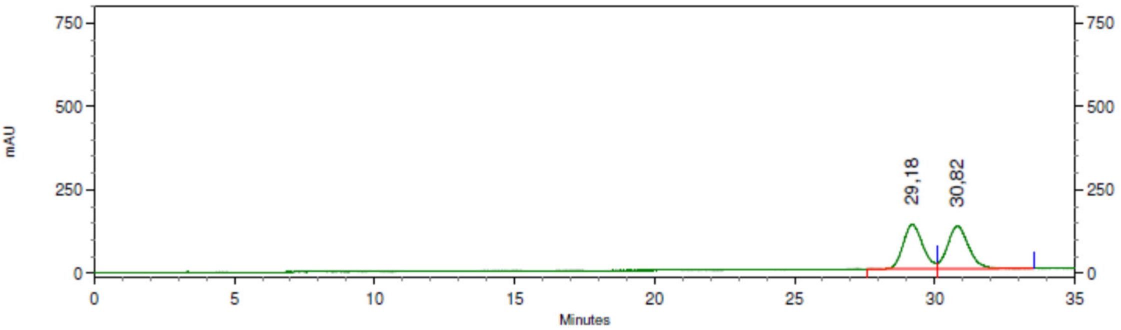

17: 292 nm, 4 nm
Results

Retention Time	Area	Area Percent
29,18	25847381	49,147
30,82	26744869	50,853

Enantioselective reaction

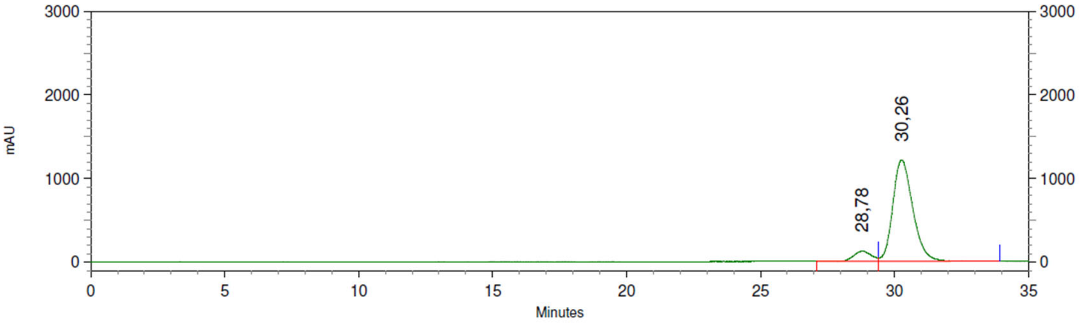

17: 292 nm, 4 nm
Results

Retention Time	Area	Area Percent
28,78	21843833	7,985
30,26	251722590	92,015

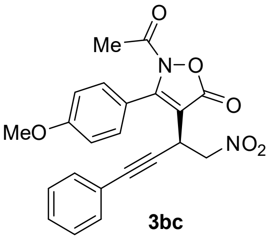

3bc

Non-enantioselective reaction

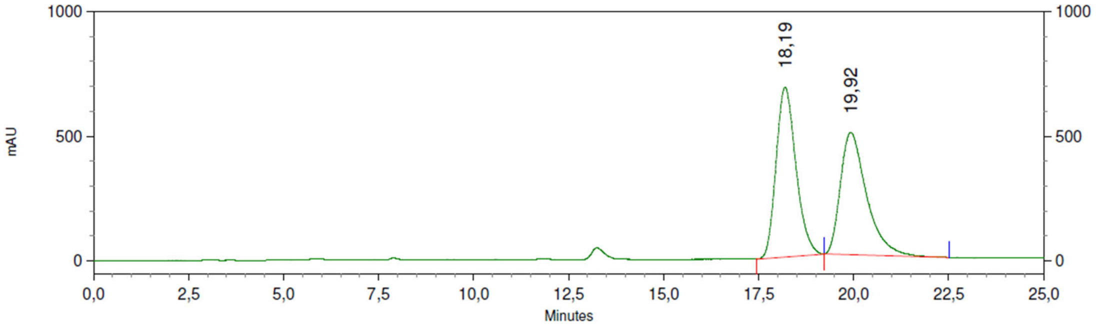

42: 240 nm, 4 nm
Results

Retention Time	Area	Area Percent
18,19	98502510	51,180
19,92	93959555	48,820

Enantioselective reaction

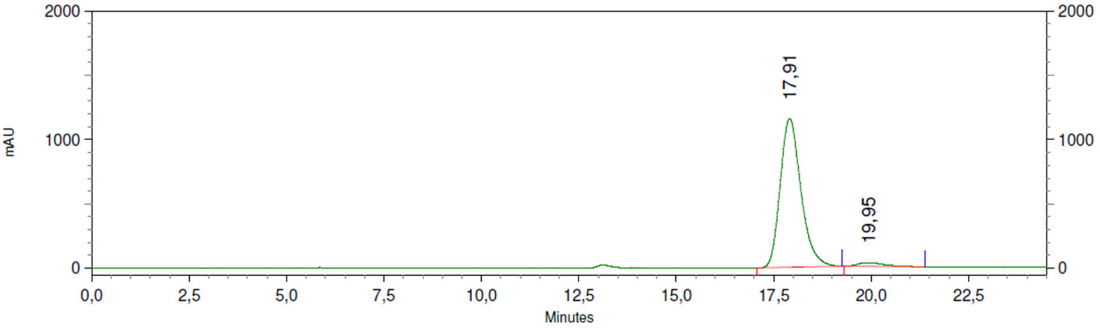

42: 240 nm, 4 nm
Results

Retention Time	Area	Area Percent
17,91	170579659	96,851
19,95	5547094	3,149

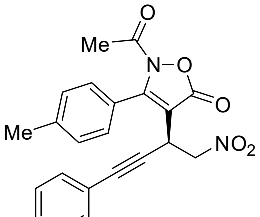

3gc

Non-enantioselective reaction

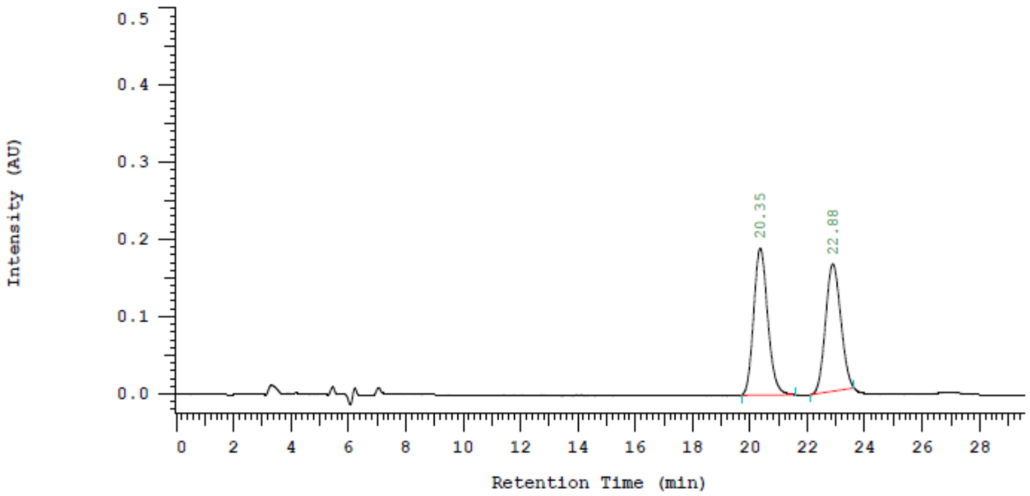

RT	Area	Name	Conc 1
20.35	3277007		52.030
22.88	3021296		47.970
	6298303		100.000

Enantioselective reaction

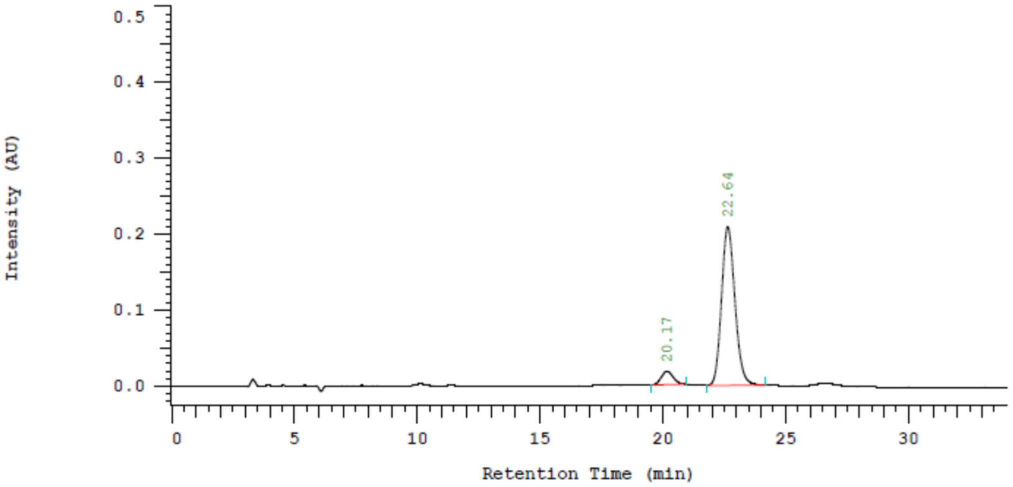

RT	Area	Name	Conc 1
20.17	293687		6.920
22.64	3950213		93.080
	4243900		100.000

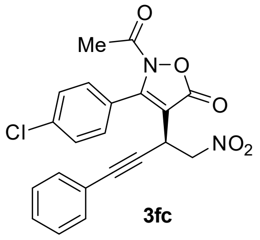

3fc

Non-enantioselective reaction

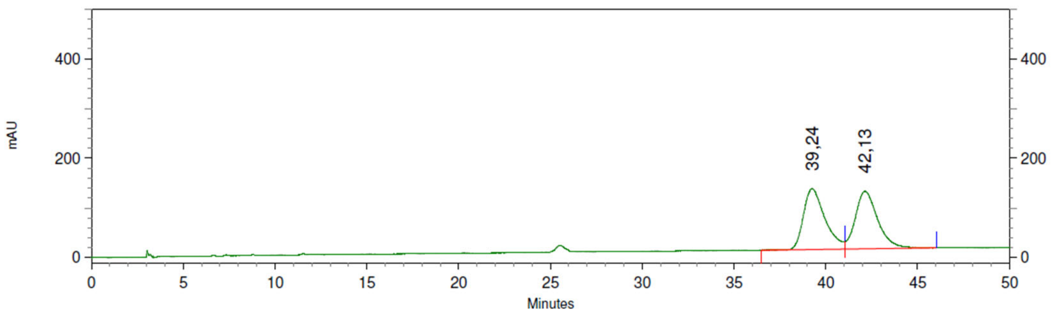

6: 244 nm, 4 nm Results

Retention Time	Area	Area Percent
39,24	39545696	50,248
42,13	39155128	49,752

Enantioselective reaction

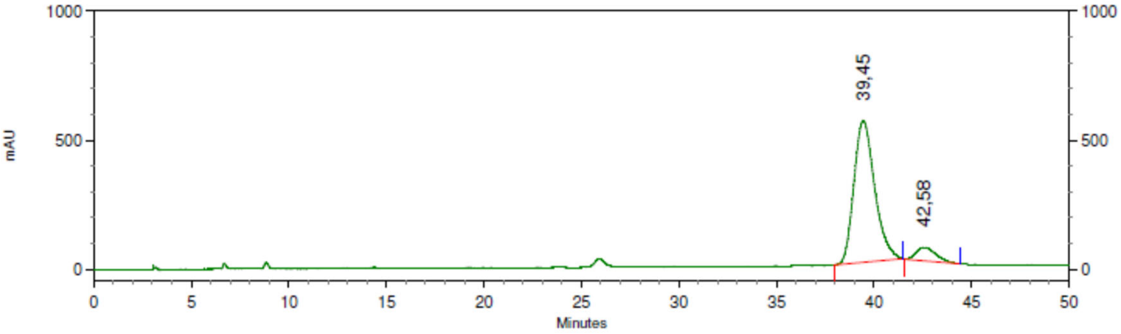

6: 244 nm, 4 nm Results

Retention Time	Area	Area Percent
39,45	162043936	91,611
42,58	14839166	8,389

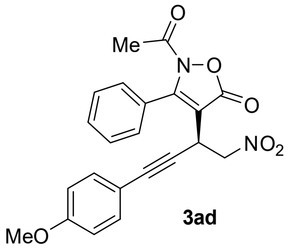

3ad

Non-enantioselective reaction

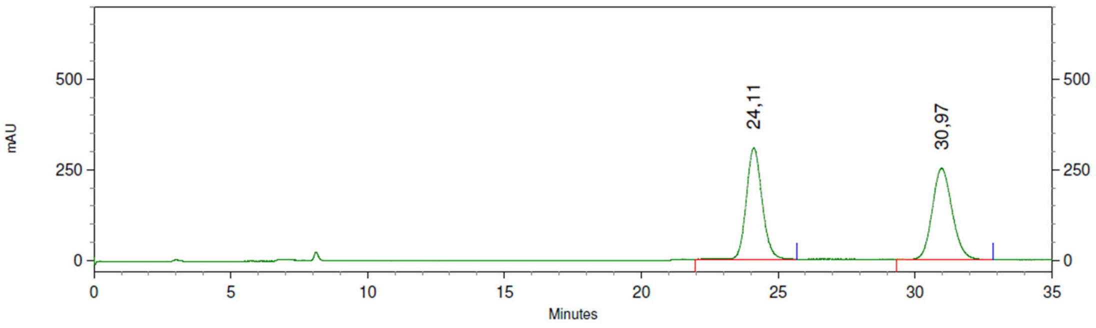

33: 251 nm, 4 nm
Results

Retention Time	Area	Area Percent
24,11	49071437	49,140
30,97	50788585	50,860

Enantioselective reaction

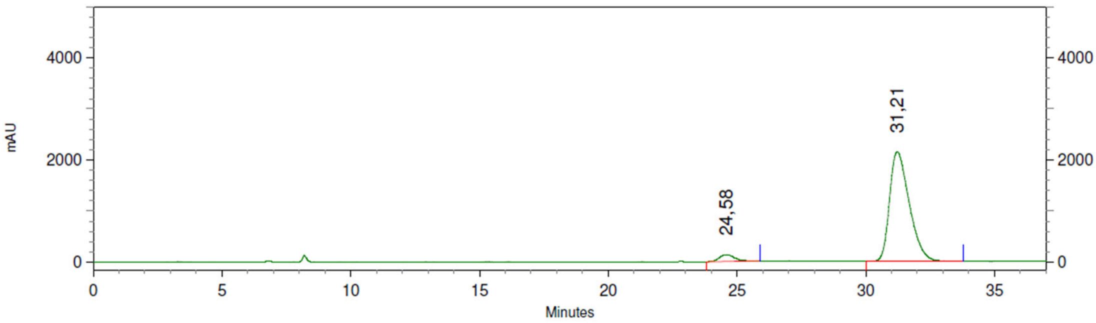

33: 251 nm, 4 nm
Results

Retention Time	Area	Area Percent
24,58	21791017	4,564
31,21	455626855	95,436

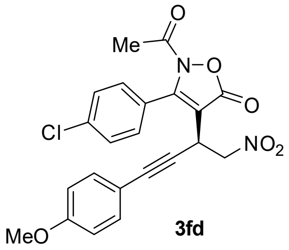

3fd

Non-enantioselective reaction

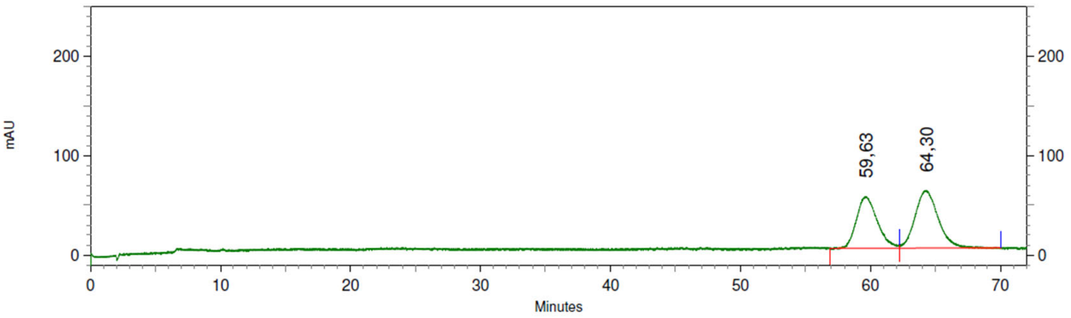

3: 252 nm, 4 nm Results

Retention Time	Area	Area Percent
59,63	22877581	44,832
64,30	28152211	55,168

Enantioselective reaction

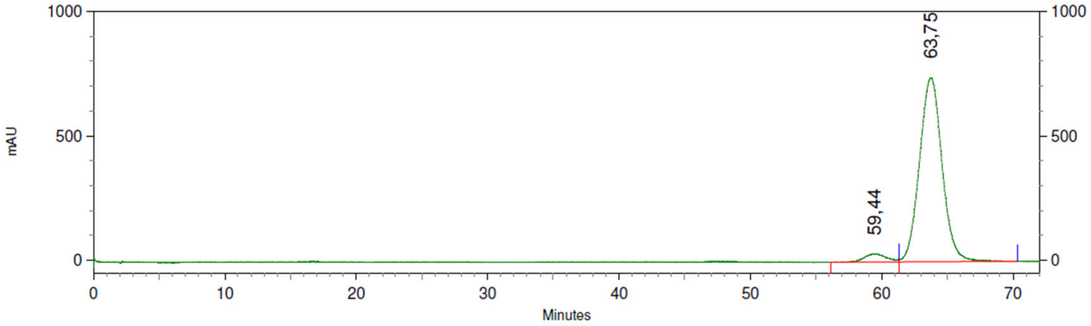

3: 252 nm, 4 nm Results

Retention Time	Area	Area Percent
59,44	15091367	4,103
63,75	352728632	95,897

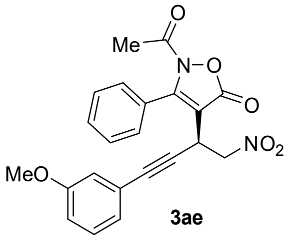

3ae

Non-enantioselective reaction

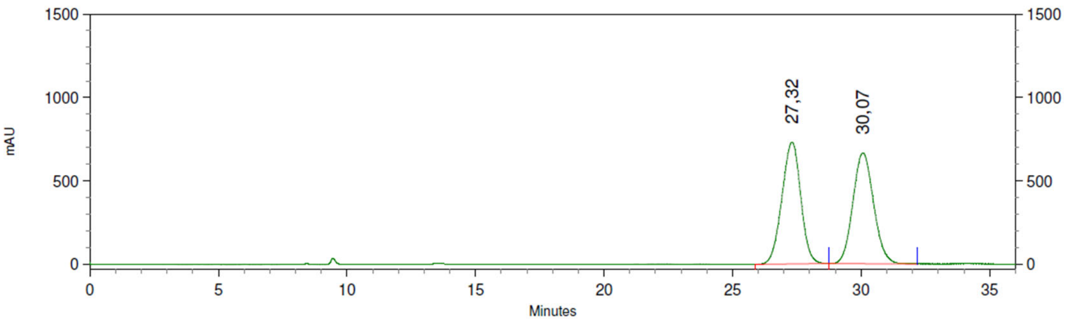

7: 289 nm, 4 nm Results

Retention Time	Area	Area Percent
27,32	148380308	49,977
30,07	148518212	50,023

Enantioselective reaction

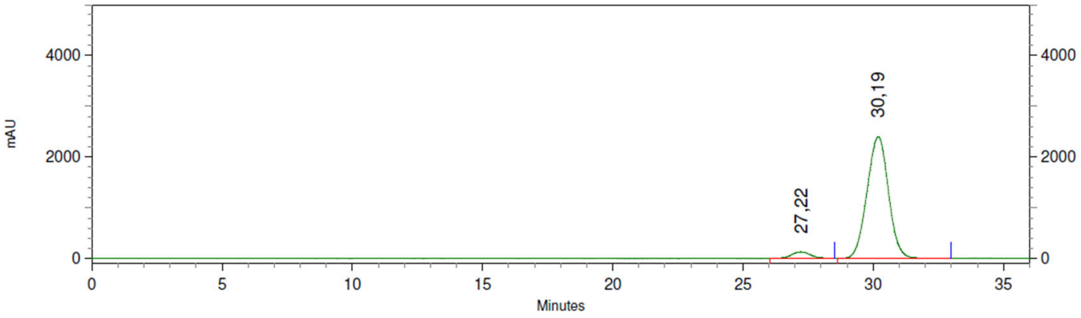

7: 289 nm, 4 nm Results

Retention Time	Area	Area Percent
27,22	26072383	4,559
30,19	545830576	95,441

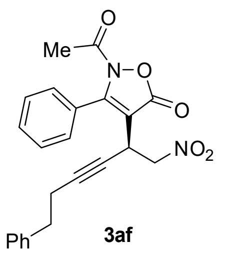

3af

Non-enantioselective reaction

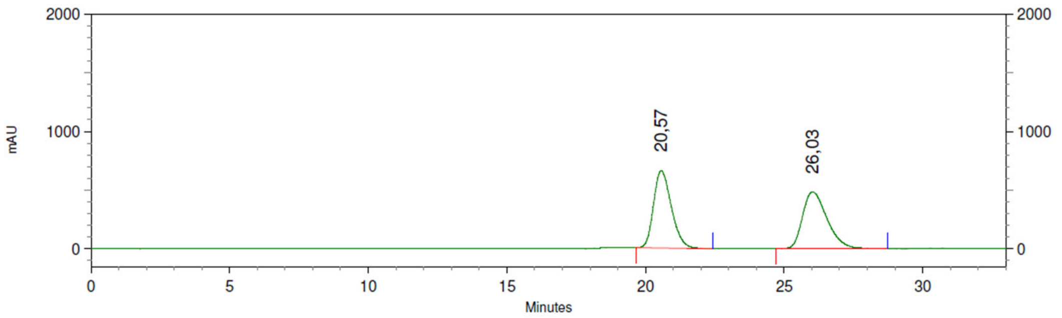

34: 292 nm, 4 nm
Results

Retention Time	Area	Area Percent
20,57	117123817	49,698
26,03	118546850	50,302

Enantioselective reaction

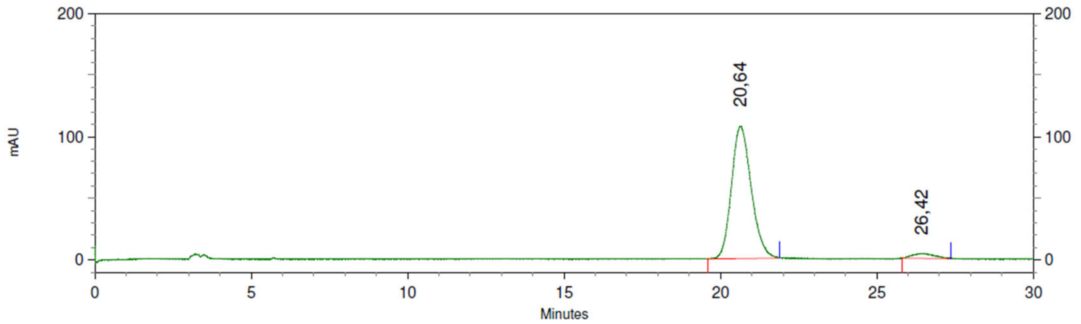

34: 292 nm, 4 nm
Results

Retention Time	Area	Area Percent
20,64	18854065	95,996
26,42	786308	4,004

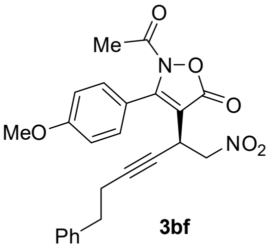

Non-enantioselective reaction

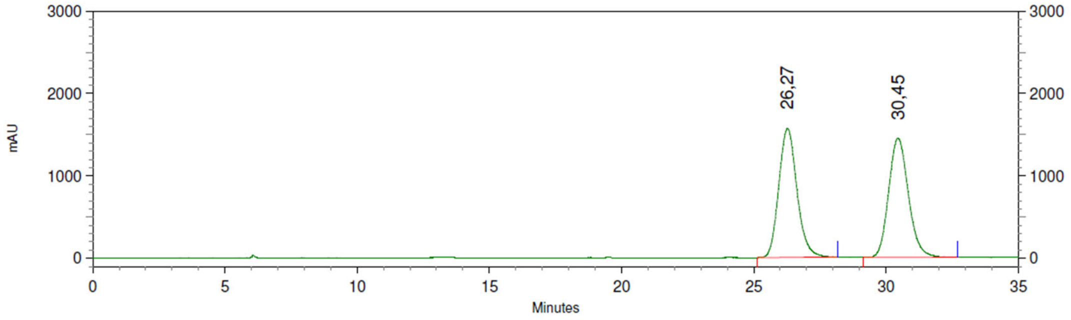

44: 281 nm, 4 nm
Results

Retention Time	Area	Area Percent
26,27	299270525	49,975
30,45	299566712	50,025

Enantioselective reaction

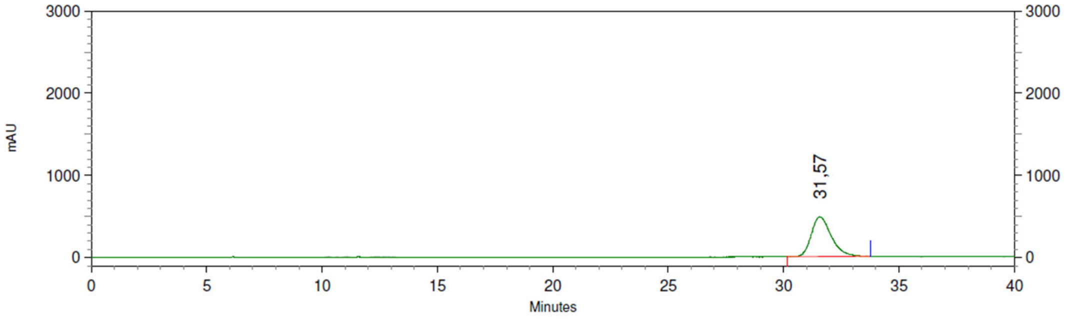

44: 281 nm, 4 nm
Results

Retention Time	Area	Area Percent
31,57	116038652	100,000

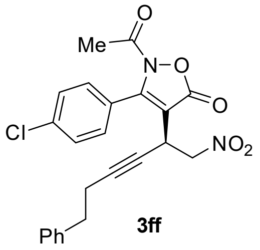

3ff

Non-enantioselective reaction

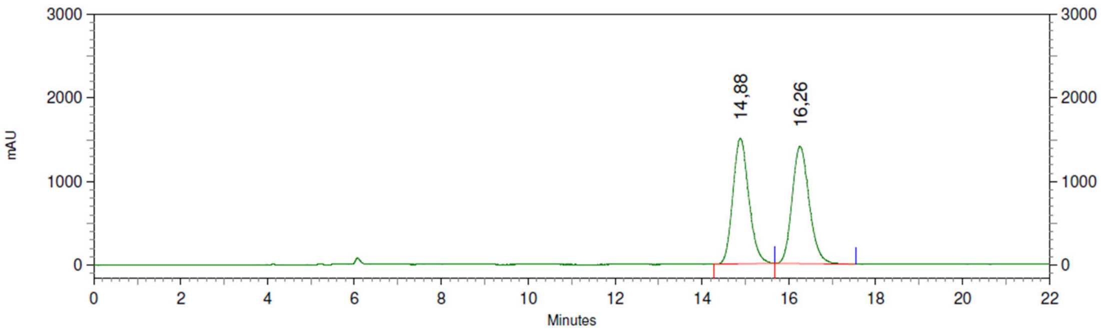

44: 281 nm, 4 nm
Results

Retention Time	Area	Area Percent
14,88	153323387	49,910
16,26	153875101	50,090

Enantioselective reaction

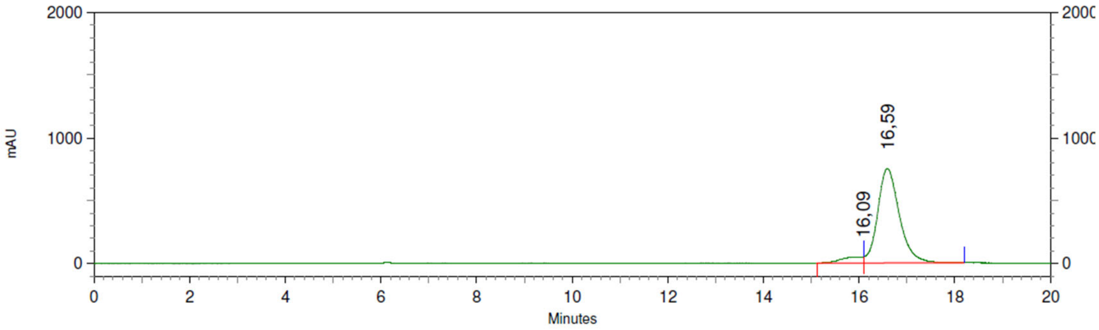

44: 281 nm, 4 nm
Results

Retention Time	Area	Area Percent
16,09	6130546	6,074
16,59	94798362	93,926

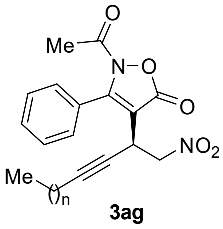

3ag

Non-enantioselective reaction

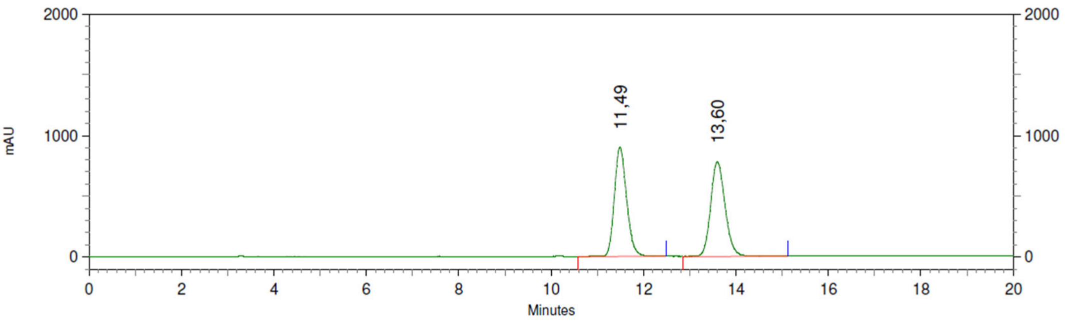

40: 290 nm, 4 nm
Results

Retention Time	Area	Area Percent
11,49	66483715	49,568
13,60	67643750	50,432

Enantioselective reaction

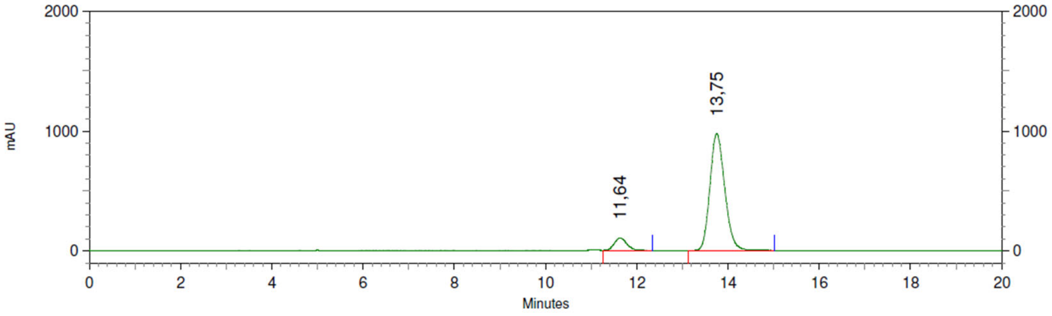

40: 290 nm, 4 nm
Results

Retention Time	Area	Area Percent
11,64	7987141	8,309
13,75	88134832	91,691

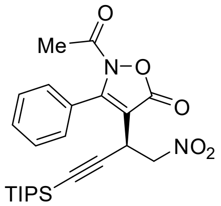

3ah

Non-enantioselective reaction

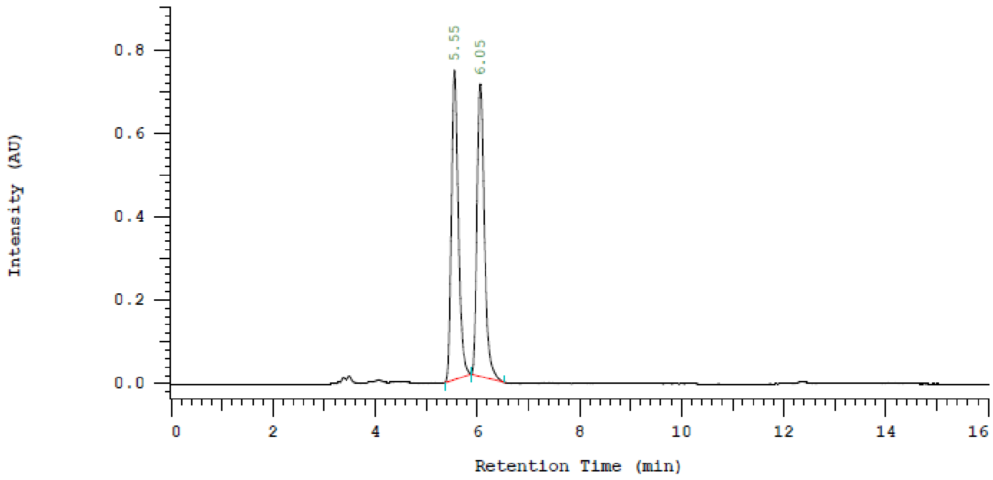

RT	Area	Name	Conc 1
5.55	3533780		50.104
6.05	3519080		49.896
	7052860		100.000

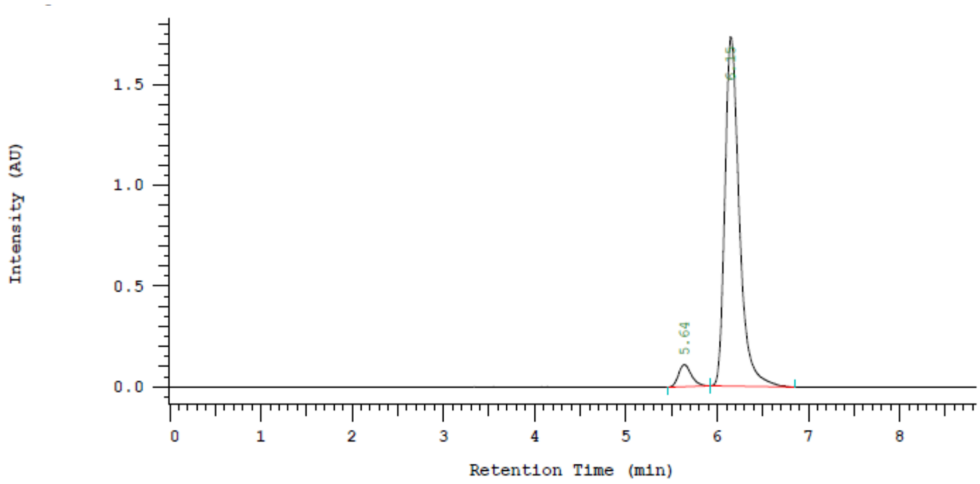

RT	Area	Name	Conc 1
5.64	531590		5.223
6.15	9646220		94.777
	10177810		100.000

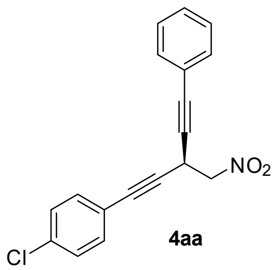

4aa

Non-enantioselective reaction

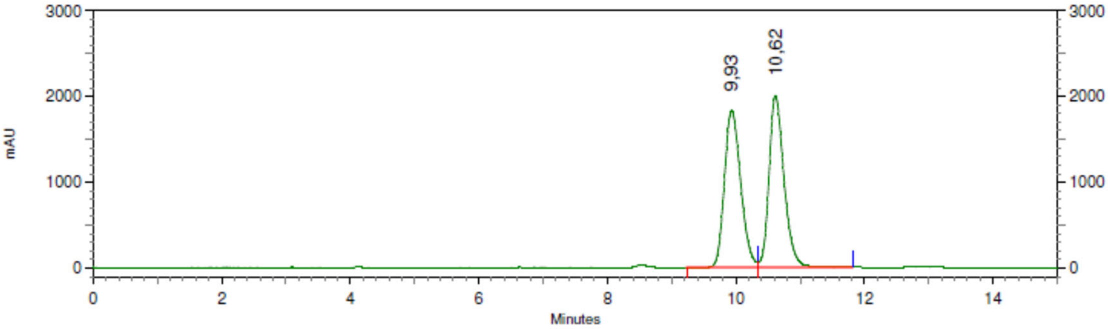

16: 251 nm, 4 nm
Results

Retention Time	Area	Area Percent
9,93	128270242	49,715
10,62	129742608	50,285

Enantioselective reaction

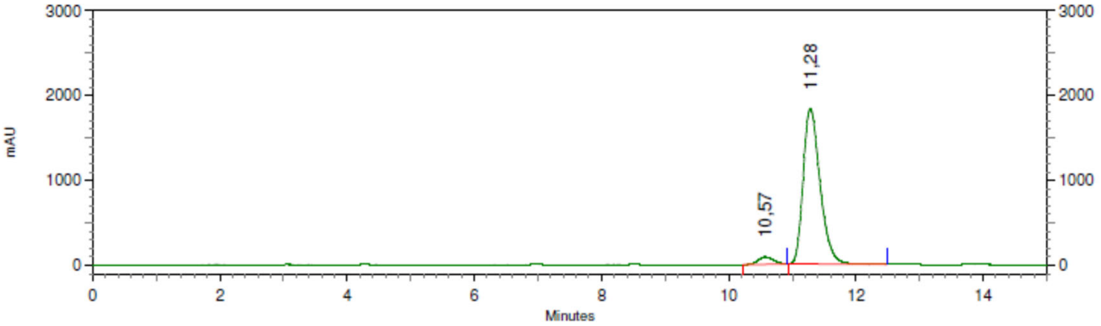

16: 251 nm, 4 nm
Results

Retention Time	Area	Area Percent
10,57	5834718	4,147
11,28	134856216	95,853

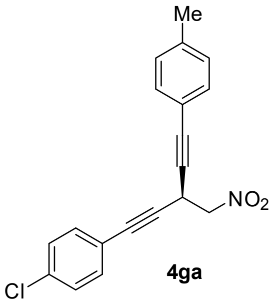

4ga

Non-enantioselective reaction

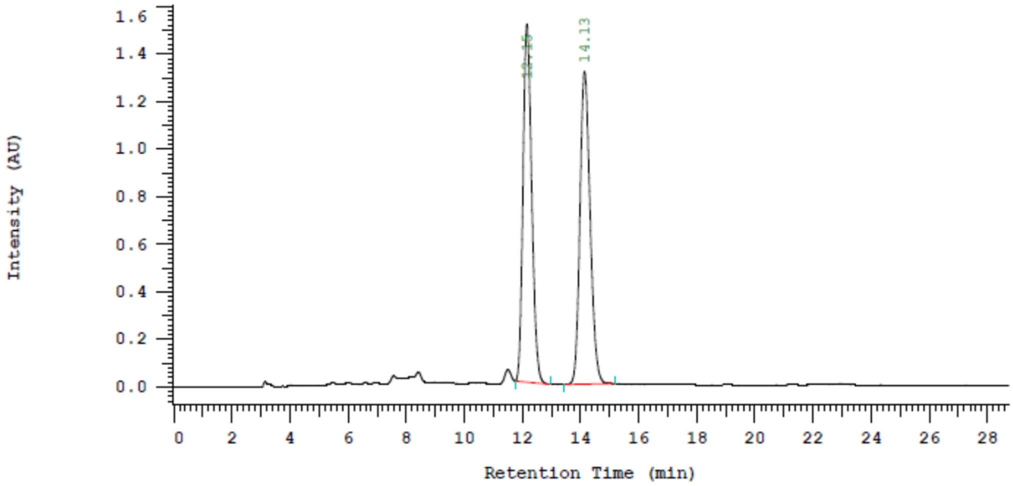

RT	Area	Name	Conc 1
12.15	15521430		49.294
14.13	15966060		50.706
	31487490		100.000

Enantioselective reaction

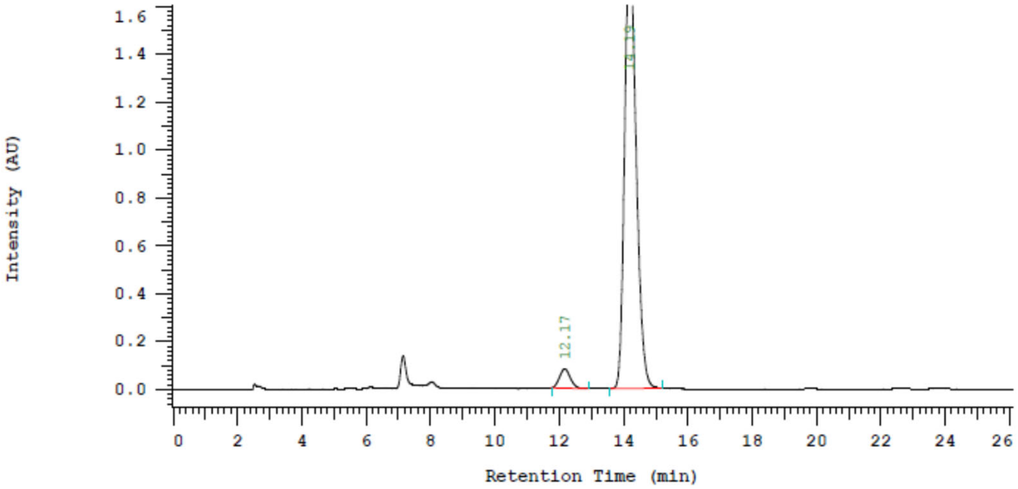

RT	Area	Name	Conc 1
12.17	955860		3.955
14.19	23212750		96.045
	24168610		100.000

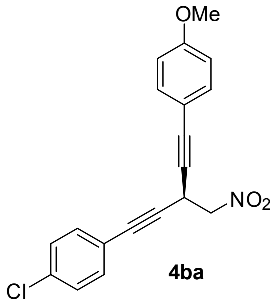

4ba

Non-enantioselective reaction

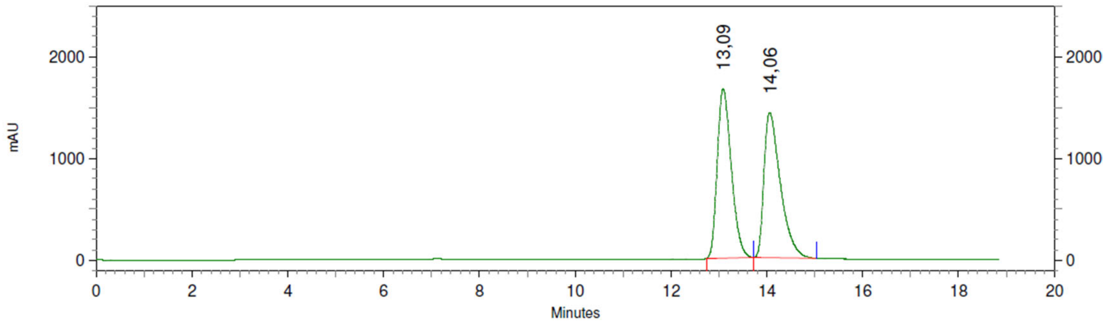

6: 259 nm, 4 nm Results

Retention Time	Area	Area Percent
13,09	135974892	49,980
14,06	136086206	50,020

Enantioselective reaction

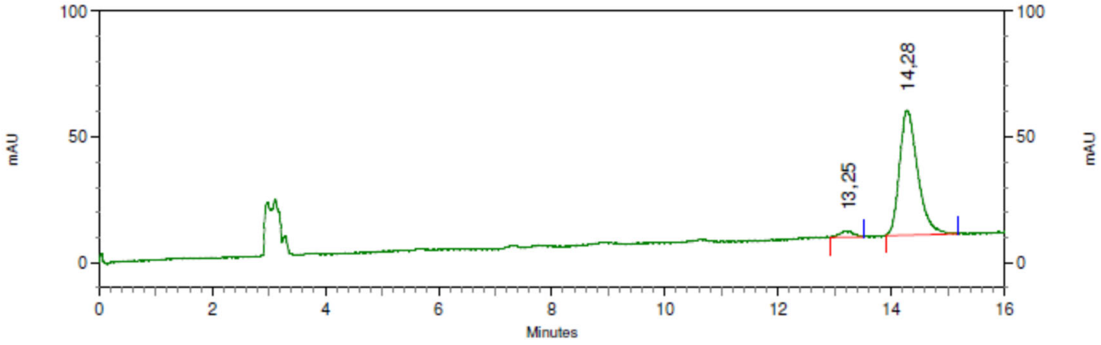

9: 210 nm, 4 nm Results

Retention Time	Area	Area Percent
13,25	172427	3,730
14,28	4449957	96,270

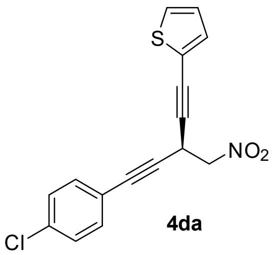

4da

Non-enantioselective reaction

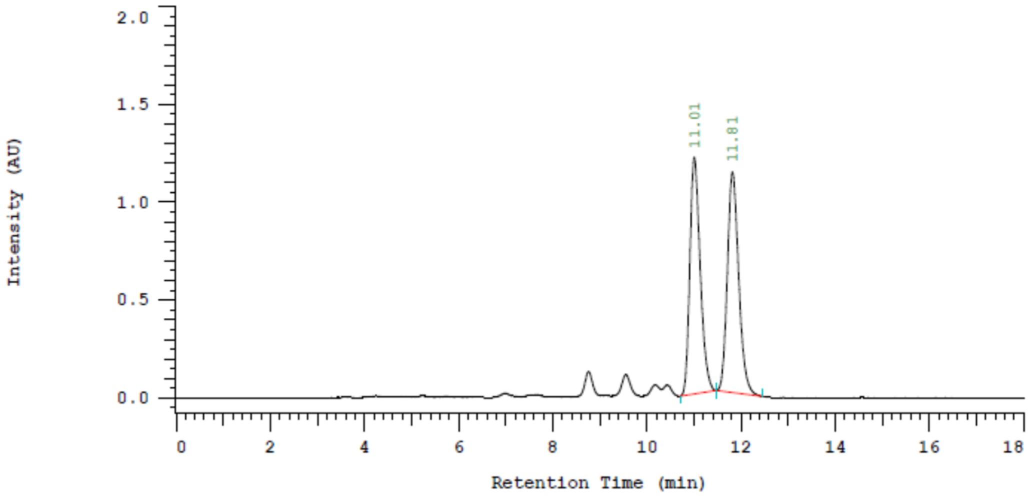

RT	Area	Name	Conc 1
11.01	9209630		49.396
11.81	9434790		50.604
	18644420		100.000

Enantioselective reaction

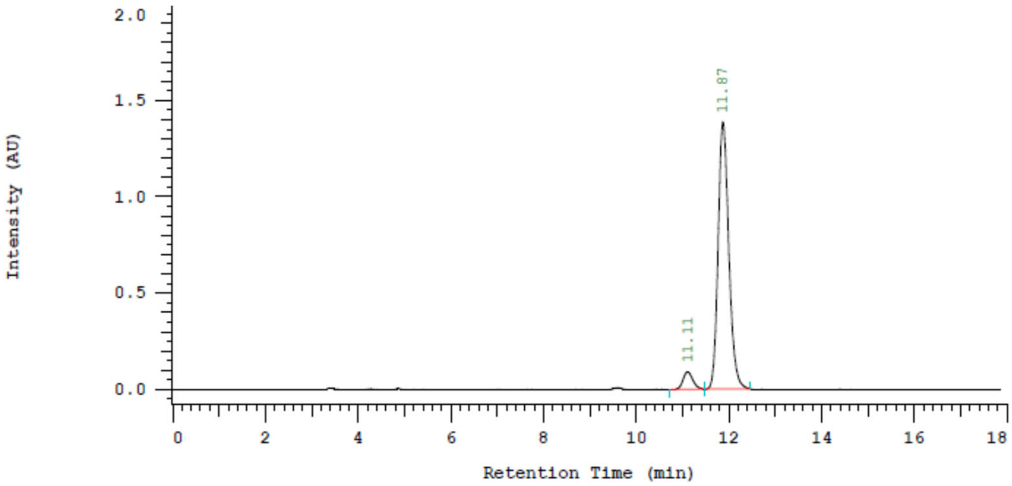

RT	Area	Name	Conc 1
11.11	664090		5.658
11.87	11072800		94.342
	11736890		100.000

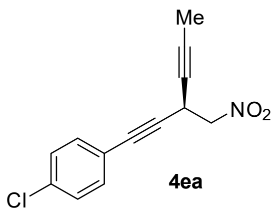

4ea

Non-enantioselective reaction

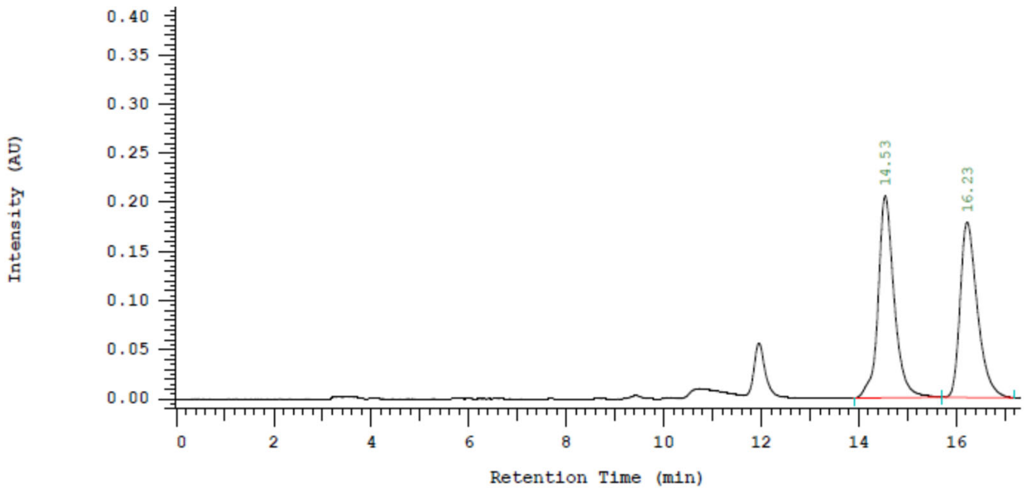

RT	Area	Name	Conc 1
14.53	2344660		51.677
16.23	2192520		48.323
	4537180		100.000

Enantioselective reaction

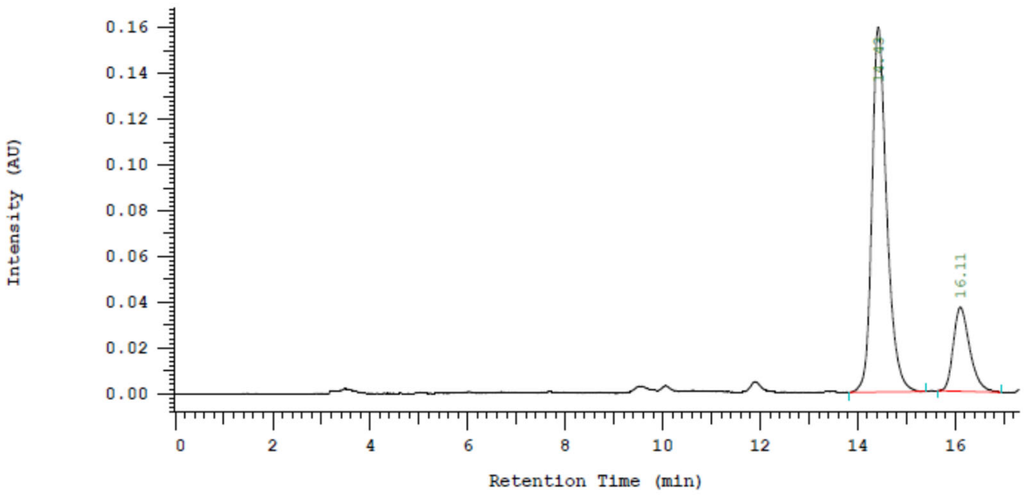

RT	Area	Name	Conc 1
14.43	1680260		79.560
16.11	431680		20.440
	2111940		100.000

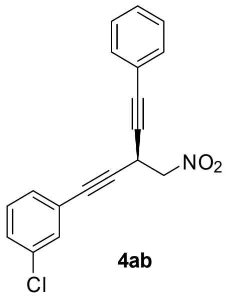

4ab

Non-enantioselective reaction

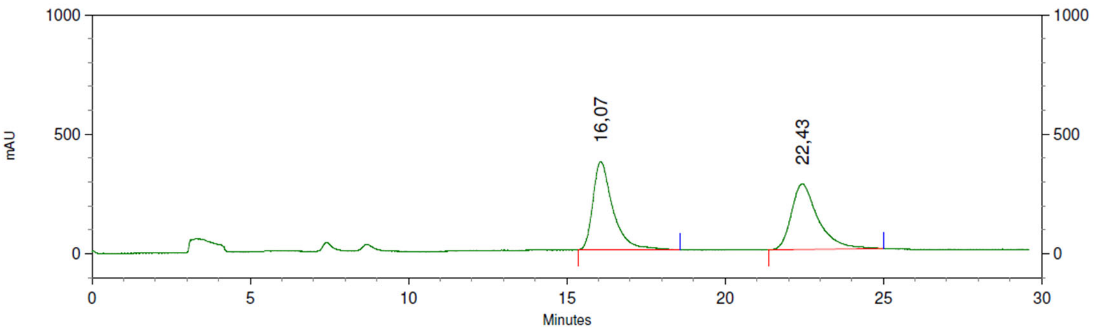

36: 204 nm, 4 nm
Results

Retention Time	Area	Area Percent
16,07	65543935	50,425
22,43	64439348	49,575

Enantioselective reaction

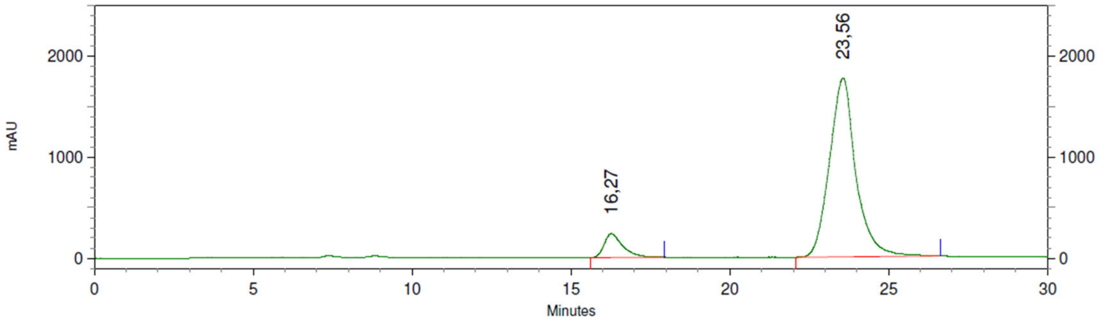

32: 251 nm, 4 nm
Results

Retention Time	Area	Area Percent
16,27	38334374	8,601
23,56	407351649	91,399

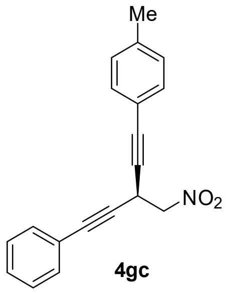

4gc

Non-enantioselective reaction

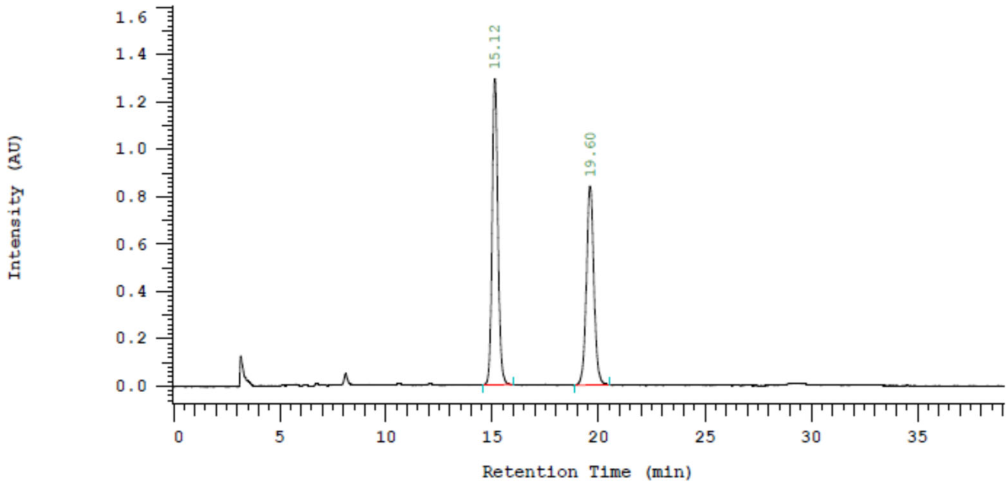

RT	Area	Name	Conc 1
15.12	12224020		54.349
19.60	10267510		45.651
	22491530		100.000

Enantioselective reaction

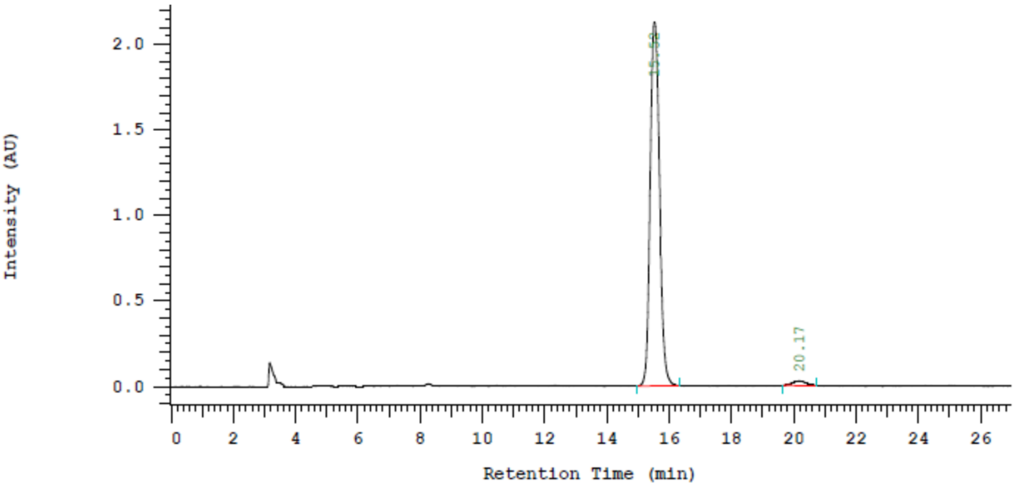

RT	Area	Name	Conc 1
15.52	22337960		98.177
20.17	414710		1.823
	22752670		100.000

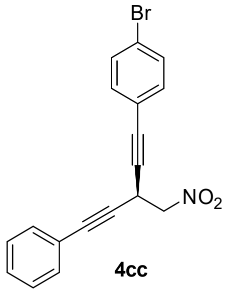

4cc

Non-enantioselective reaction

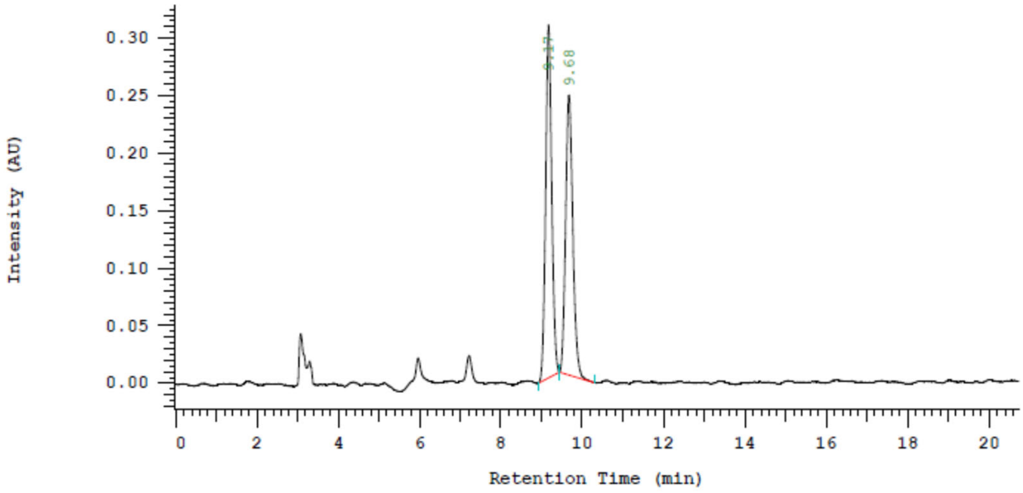

RT	Area	Name	Conc 1
9.17	1583460		50.945
9.68	1524700		49.055
	3108160		100.000

Enantioselective reaction

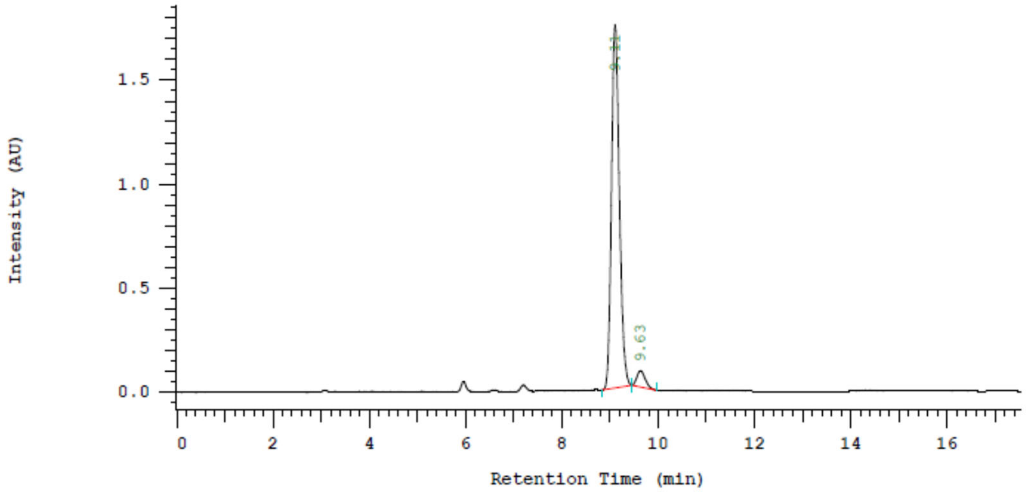

RT	Area	Name	Conc 1
9.11	9899760		95.437
9.63	473300		4.563
	10373060		100.000

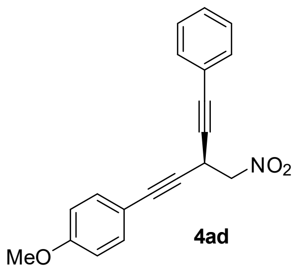

4ad

Non-enantioselective reaction

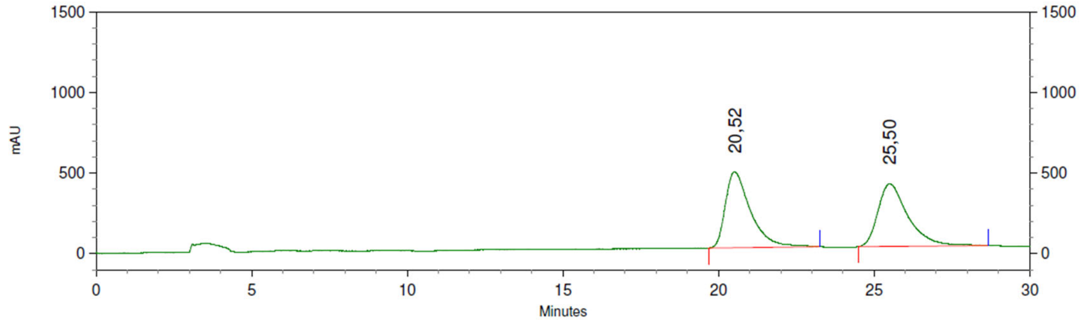

36: 204 nm, 4 nm
Results

Retention Time	Area	Area Percent
20,52	106339512	50,349
25,50	104864601	49,651

Enantioselective reaction

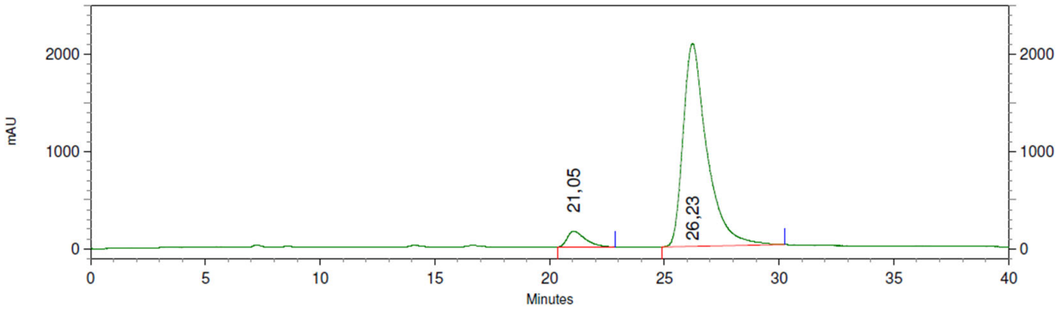

41: 252 nm, 4 nm
Results

Retention Time	Area	Area Percent
21,05	35006964	5,472
26,23	604705001	94,528

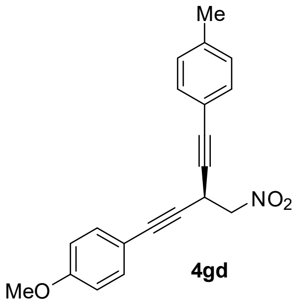

4gd

Non-enantioselective reaction

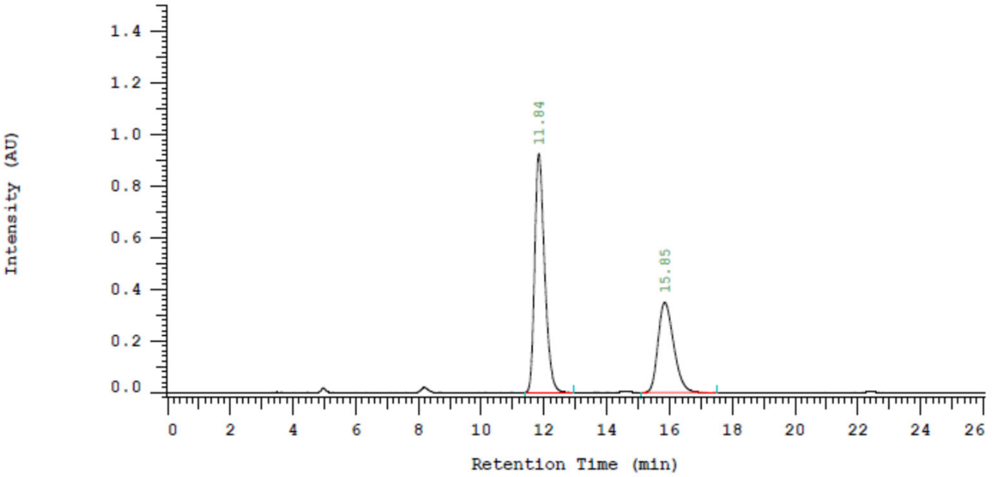

RT	Area	Name	Conc 1
11.84	10192620		62.719
15.85	6058720		37.281
	16251340		100.000

Enantioselective reaction

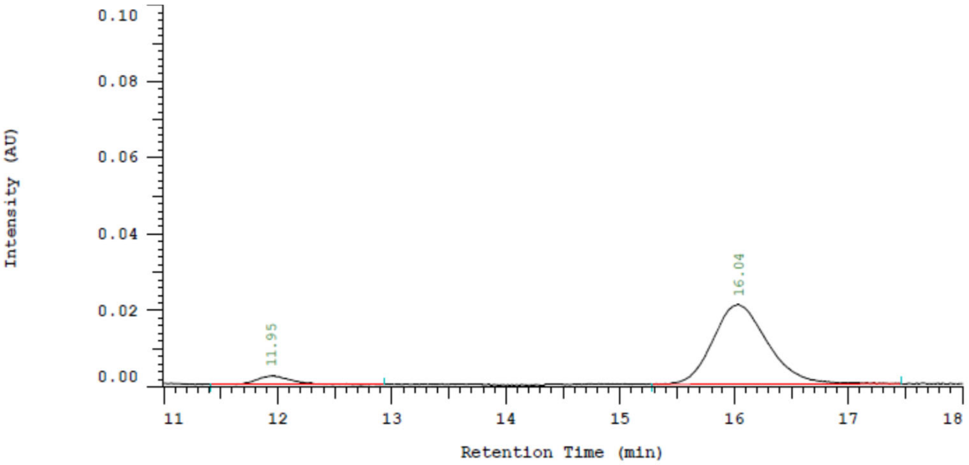

RT	Area	Name	Conc 1
11.95	24980		6.672
16.04	349420		93.328
	374400		100.000

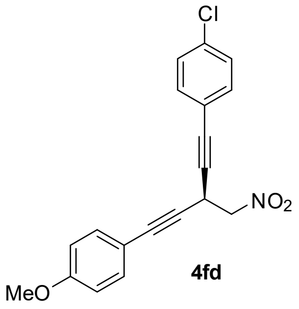

4fd

Non-enantioselective reaction

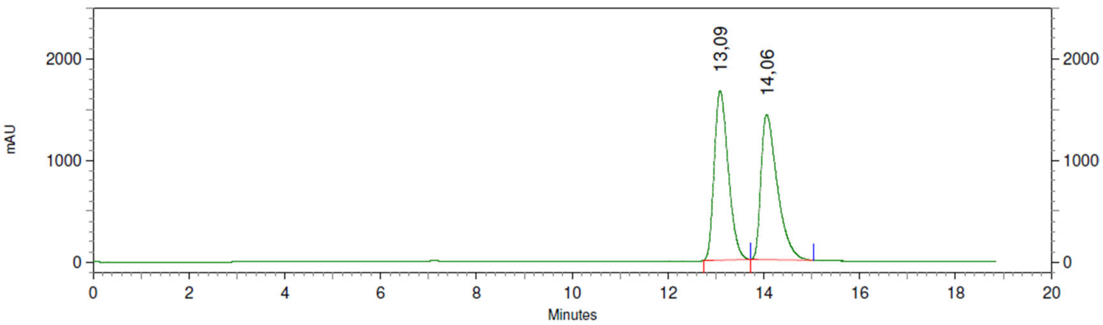

6: 259 nm, 4 nm Results

Retention Time	Area	Area Percent
13,09	135974892	49,980
14,06	136086206	50,020

Enantioselective reaction

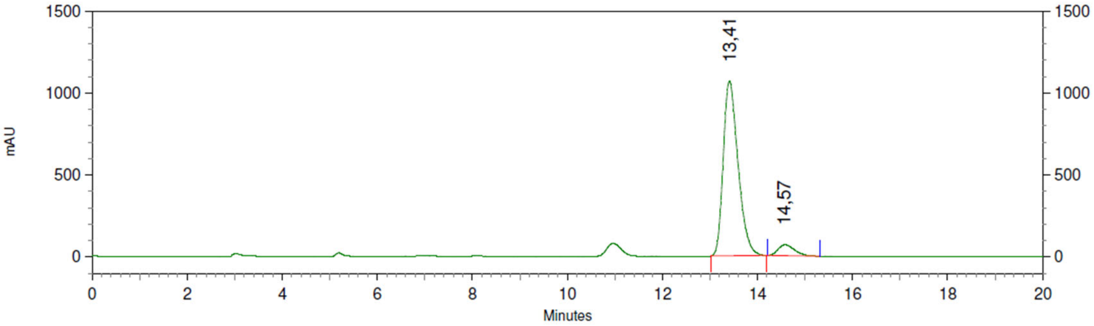

6: 259 nm, 4 nm Results

Retention Time	Area	Area Percent
13,41	92274046	93,369
14,57	6553614	6,631

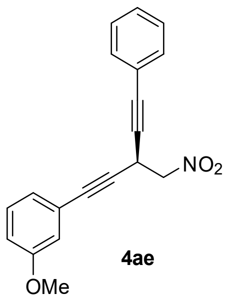

4ae

Non-enantioselective reaction

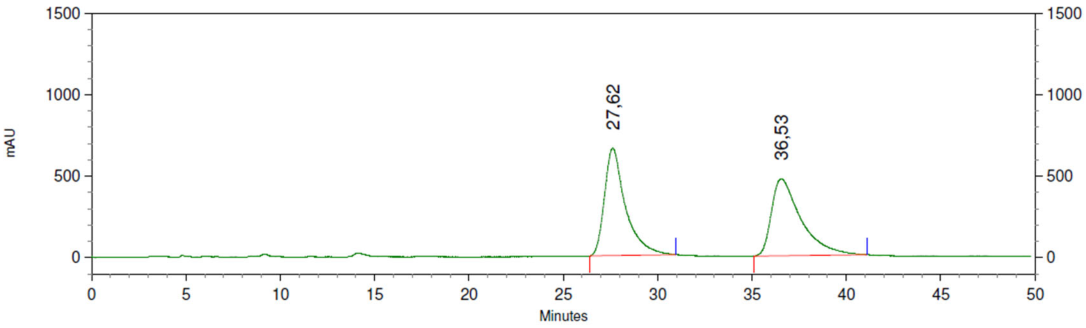

32: 251 nm, 4 nm
Results

Retention Time	Area	Area Percent
27,62	204697087	49,905
36,53	205479518	50,095

Enantioselective reaction

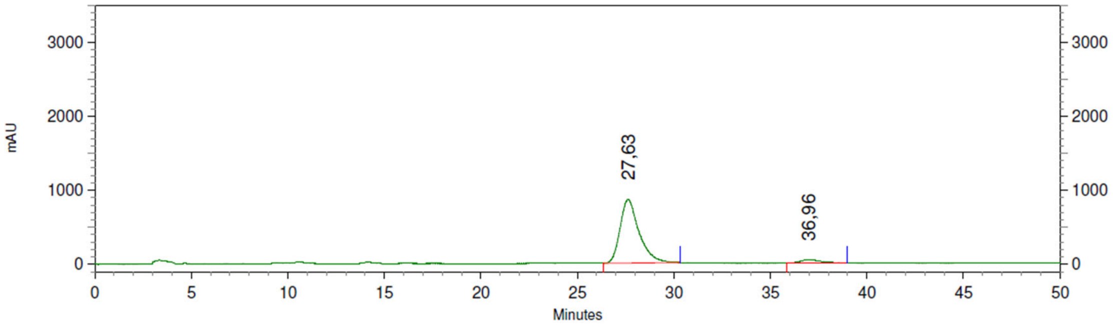

38: 205 nm, 4 nm
Results

Retention Time	Area	Area Percent
27,63	234265049	94,204
36,96	14412255	5,796

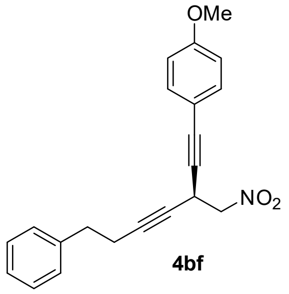

4bf

Non-enantioselective reaction

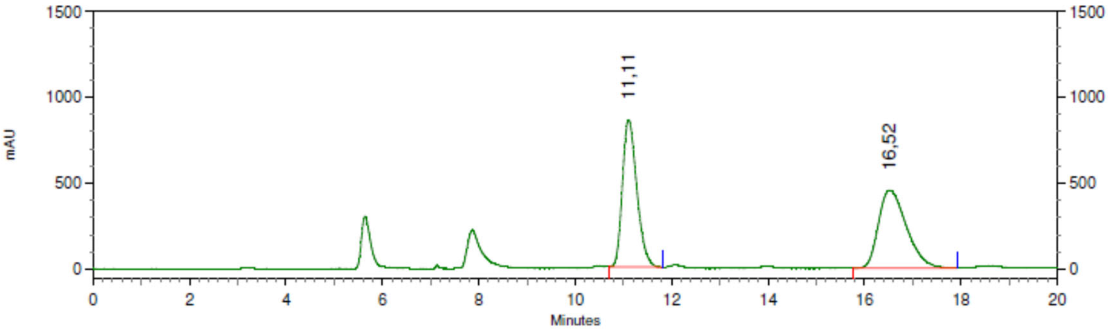

37: 260 nm, 4 nm
Results

Retention Time	Area	Area Percent
11,11	71312828	49,322
16,52	73274614	50,678

Enantioselective reaction

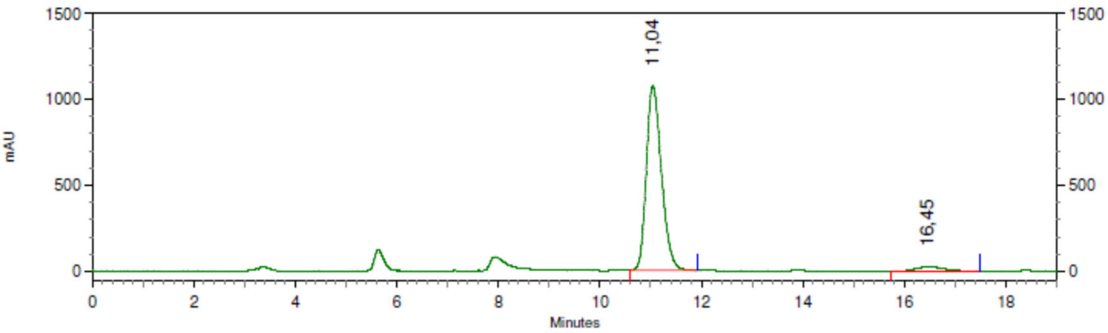

37: 260 nm, 4 nm
Results

Retention Time	Area	Area Percent
11,04	88315961	95,329
16,45	4327449	4,671

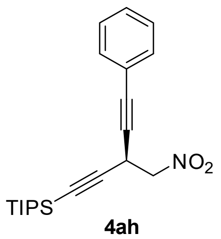

4ah

Non-enantioselective reaction

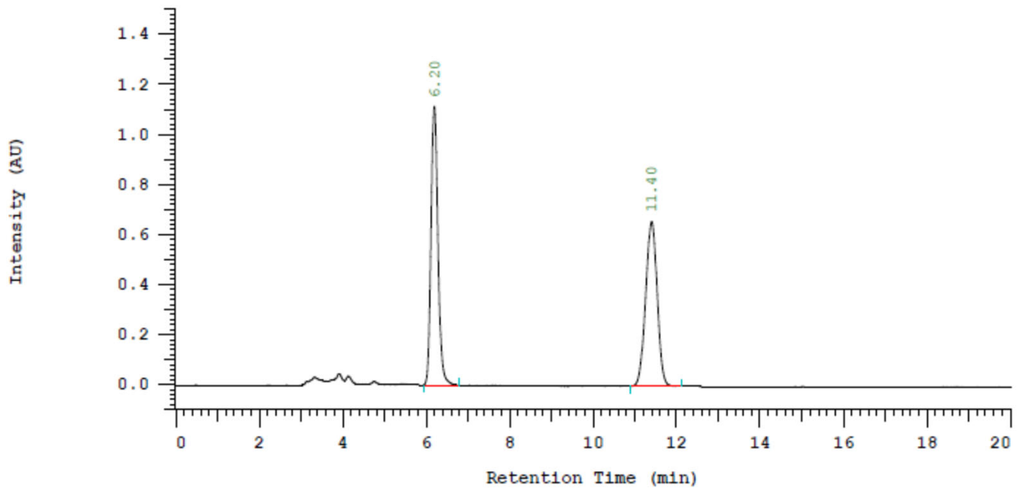

RT	Area	Name	Conc 1
6.20	6413930		50.281
11.40	6342200		49.719
	12756130		100.000

Enantioselective reaction

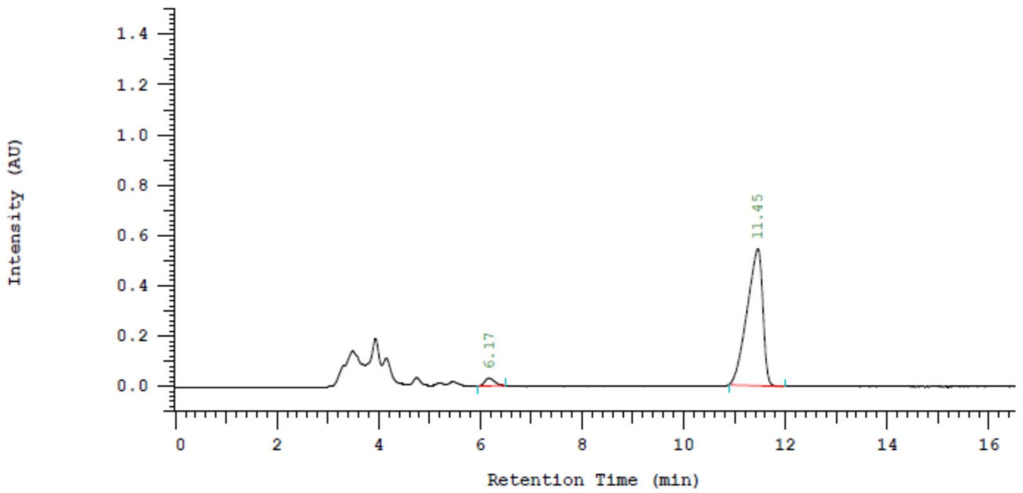

RT	Area	Name	Conc 1
6.17	201520		3.293
11.45	5918890		96.707
	6120410		100.000

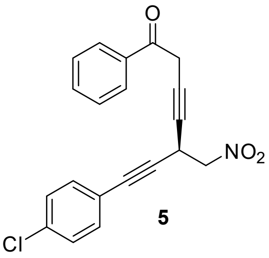

5

Enantioselective reaction

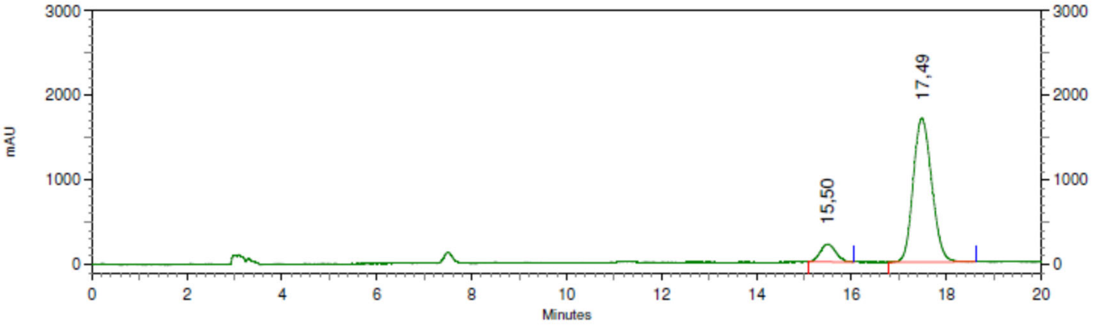

35: 205 nm, 4 nm
Results

Retention Time	Area	Area Percent
15,50	19301380	9,440
17,49	185153251	90,560

Stereochemical model for the enantioselective Michael addition of isoxazolinones to nitroenynes

The stereochemistry of the reaction is determined at the Michael addition step catalyzed by the bi-functional squaramide. According to antecedents reported in the literature,[5] the enantioselective organocatalytic Michael Addition involves the synergistic activation of the isoxazolinone and the nitroenyne by the bifunctional squaramide **V** through the formation of hydrogen bonds between the reaction partners and the squaramide and protonated tertiary amide moieties. Based on computational calculations (see below) we propose a TS model where the nitroenyne is forming a hydrogen bond with the protonated amine while the isoxazolinone enolate is hydrogen-bonded to the squaramide moiety favoring the approach of the *Si* face of the isoxazolinone to the *Re* face of the nitroenyne double bond, to give the corresponding mixture of tautomers. Ulterior acylation with acetic anhydride takes place on the N atom to give compounds 3 with the *S* configuration at the sterogenic center (**Scheme S1**).

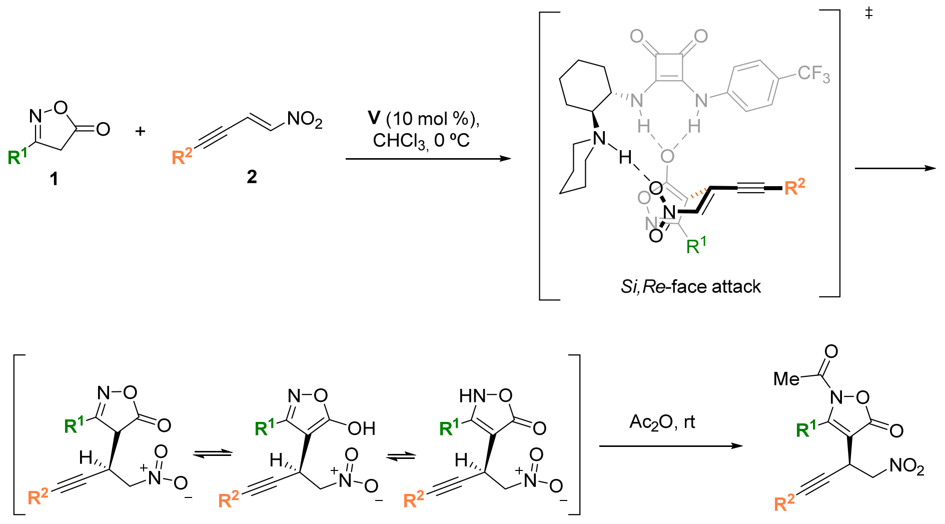

Scheme S1. Stereochemical model for the conjugate addition of isoxazolinones to nitroenynes

Computational methods

All calculations were carried out using density functional theory with the B3LYP-D3(BJ) functional[6] as implemented in the Gaussian 09 package.[7] For geometry optimizations, the 6-31G(d,p) basis set was used. Thermochemical (calculated at 273.15 K) and solvation corrections (calculated with chloroform as solvent and the polarizable

continuum model (PCM) method[8] were added as single points at the same level of theory as the geometry optimization. Optimized structures for the transition states shown below have been represented using CYLview20.[9]

Results and discussion

In order to get insights about the stereochemical model of our system, we have performed preliminary theoretical calculations for the reaction between isoxazol-5(4*H*)-one **1a** and nitroenyne **2a**. For this initial theoretical study, the structure of the squaramide bifunctional catalyst has been simplified: the CF_3 group in the benzene ring was substituted by a hydrogen atom, and the cyclic tertiary amine was modeled as a -$N(Me)_2$ group.[10] For our discussion, we have taken into consideration only the formation of the C-C bond, which we assume is the stereodeterminig step (Scheme S2).

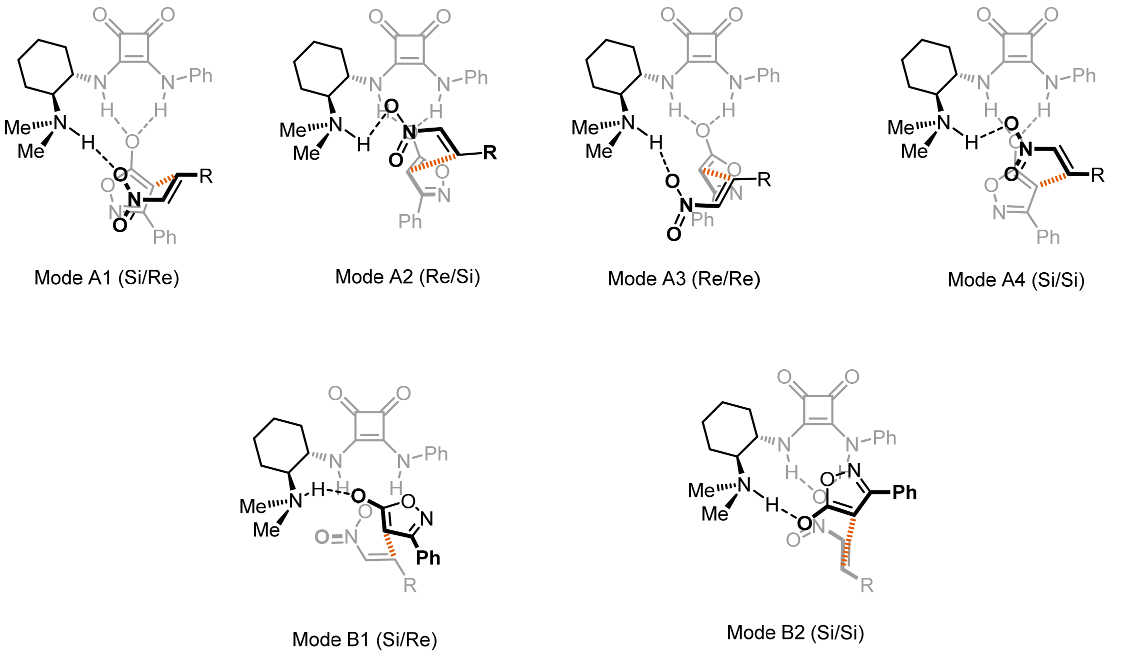

Scheme S2. Different TS considered for computational calculations

We have considered two different models: in Model A, known as Pápai's proposal,[5e] the nucleophile is deprotonated by the tertiary amine of the bifunctional catalyst. Then, the nitro group in the electrophile coordinates to this ammonium moiety, while the deprotonated nucleophile interacts with the NH's of the squaramide. For this proposal, we have been able to locate four different transition states for the four possible approaches of the reactants. The most preferred interaction (isoxazolinone's *Si*-face to nitroenyne's *Re*-face) has been computed to present 11.3 kcal/mol barrier, and would lead

to the *S*-configuration of the addition product, as observed experimentally (Tables S1 and S3). The intermediate previous to this TS is the structure with the lowest energy and serves as a reference for the system. The lowest barrier leading to the *R*-enantiomer has been computed to be 11.9 kcal/mol and corresponds to the approach of the isoxazolinone's *Re*-face to nitroenyne's *Si*-face.

Table S1: Calculated energies for the species folllowing Papai's proposal.

Isoxazolinone/nitroenyne	pre-TS	TS	post-TS
**A1_Si/Re**	0.0	11.3	5.1
**A2_Re/Si**	5.6	11.9	4.9
**A3_Re/Re**	6.4	15.7	10.2
**A4_Si/Si**	5.4	12.7	4.4

On the other hand, in Takemoto's proposal (Model B),[5f,11] nucleophile is activated by the tertiary amine of the bifunctional catalyst, while the nitro group of the electrophile is coordinated to the NH's of the squaramide. Following Takemoto's proposal, we have not been able to locate the desired transition states when the nitroenyne is approached to the *Re*-face of the isoxazolinone. Nitroenyne approaching to the *Si*-face of the isoxazolinone leads to high energy transition states: 18.2 kcal/mol approach to the Re-face of the nitroenyne and 17.1 kcal/mol if the isoxazolinone approaches to the Si-face of the nitroenyne (Table S2 and S3).

Table S2: Calculated energies for the species folllowing Takemoto's proposal.

Isoxazolinone/nitroenyne	pre-TS	TS	post-TS
**B1_Si/Re**	7.2	18.2	13.6
**B2_Si/Si**	13.5	17.1	7.5

Table S3: Calculated energies and energy corrections for all the calculated species.

Species		E_{el} (a.u.)	E_{solv} (a.u.)	G_{corr} (a.u.)	E_{rel} (kcal/mol)
**Pápai Si/Re**	**A1_pre-TS**	-2.157,38543	-2.157,40396	0,61951	0,0
	**A1_TS**	**-2.157,36674**	**-2.157,38455**	**0,61814**	**11,3**
	**A1_post-TS**	-2.157,37896	-2.157,39727	0,620978	5,1
**Pápai Re/Si**	**A2_pre-TS**	-2.157,36824	-2.157,39047	0,615011	5,6
	**A2_TS**	-2.157,36449	-2.157,38353	0,61800	11,9
	**A2_post-TS**	-2.157,37835	-2.157,39706	0,62036	4,9
**Pápai Re/Re**	**A3_pre-TS**	-2.157,36689	-2.157,39057	0,616253	6,4
	**A3_TS**	-2.157,35716	-2.157,37680	0,617407	15,7
	**A3_post-TS**	-2.157,37086	-2.157,38946	0,621294	10,2
**Pápai Si/Si**	**A4_pre-TS**	-2.157,37028	-2.157,39250	0,616575	5,4
	**A4_TS**	-2.157,36128	-2.157,38076	0,616506	12,7
	**A4_post-TS**	-2.157,37650	-2.157,39492	0,617456	4,4

**Takemoto Si/Re**	**B1_pre-TS**	- 2.157,36824	- 2.157,38799	0,615011	7,2
	**B1_TS**	- 2157,35311	- 2.157,37387	0,618379	18,2
	**B1_post-TS**	- 2.157,36334	- 2.157,38446	0,621748	13,6
**Takemoto Si/Si**	**B2_pre-TS**	- 2157,36293	- 2.157,38316	0,62018	13,5
	**B2_TS**	- 2.157,35918	- 2.157,37873	0,62155	17,1
	**B2_post-TS**	- 2.157,37188	- 2.157,39349	0,620995	7,5

A1_TS

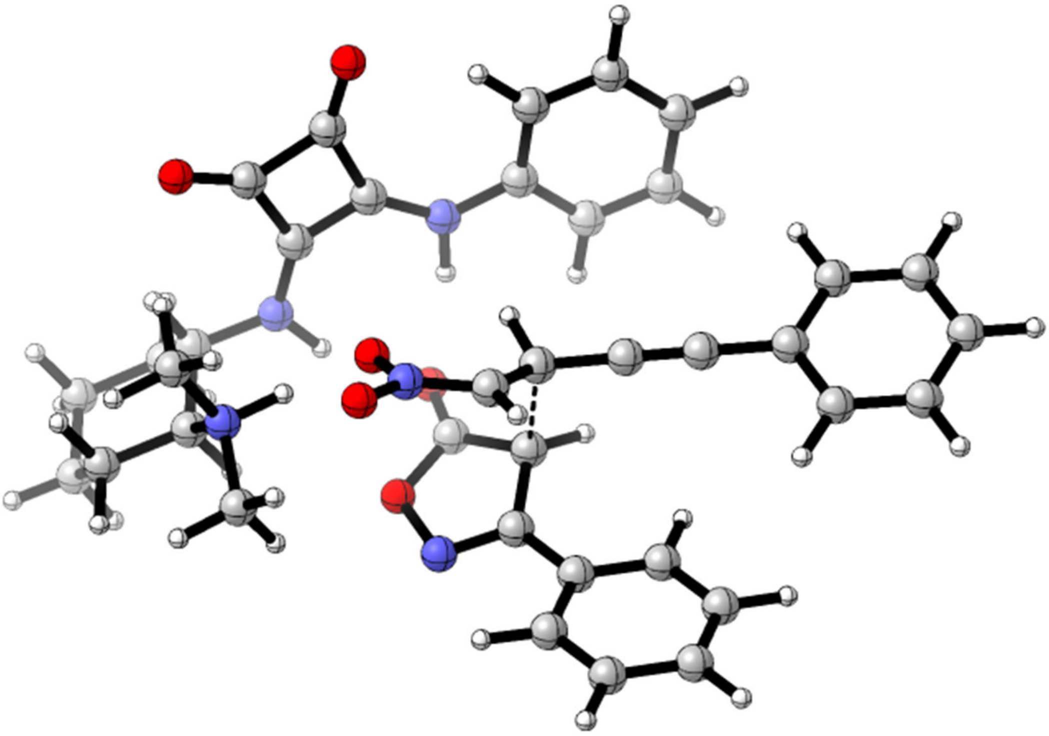

A2_TS

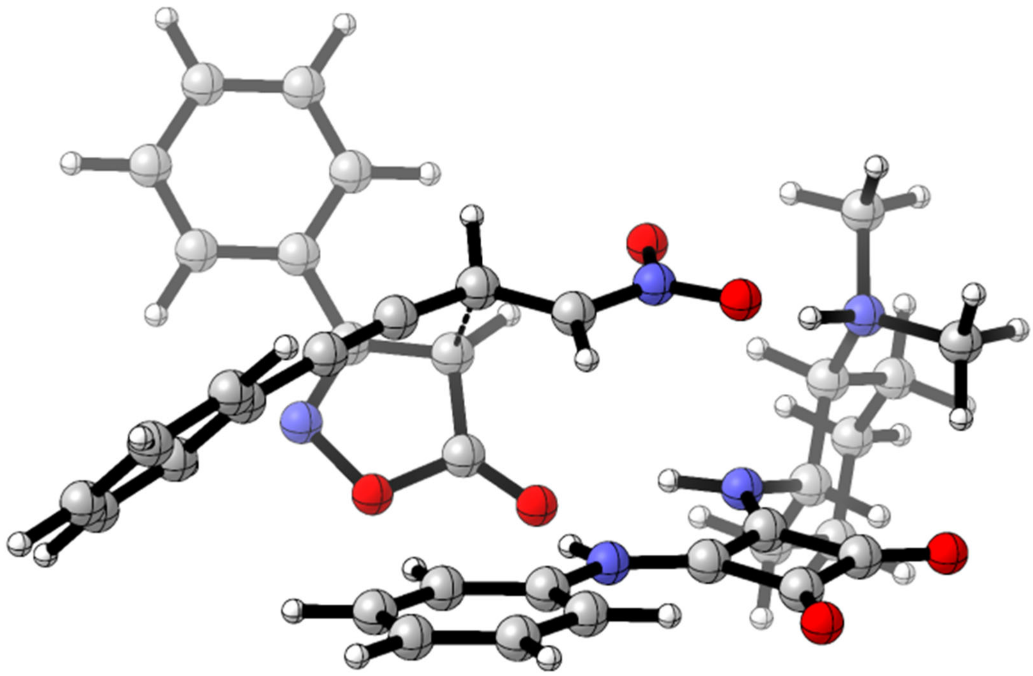

A3_TS

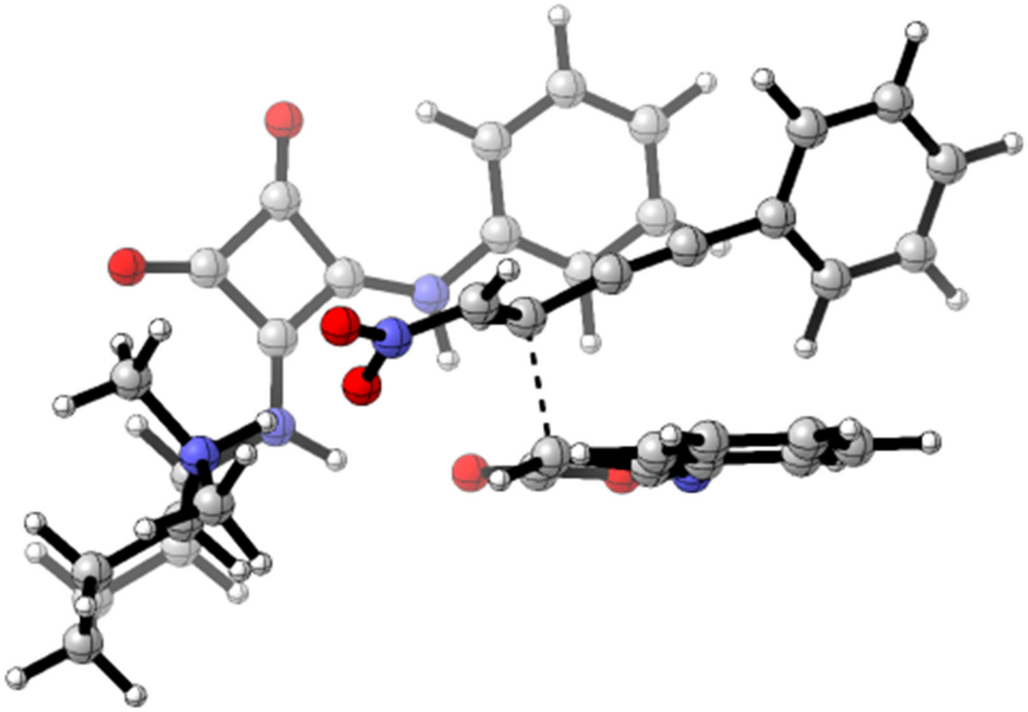

A4_TS

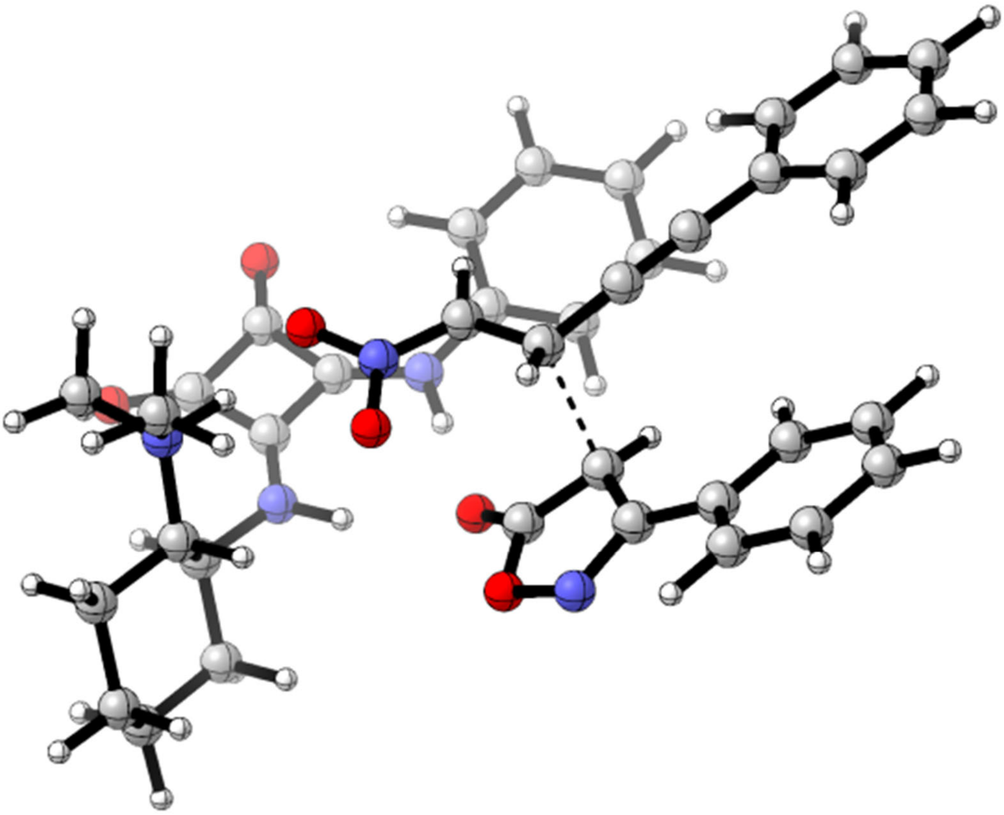

B1_TS

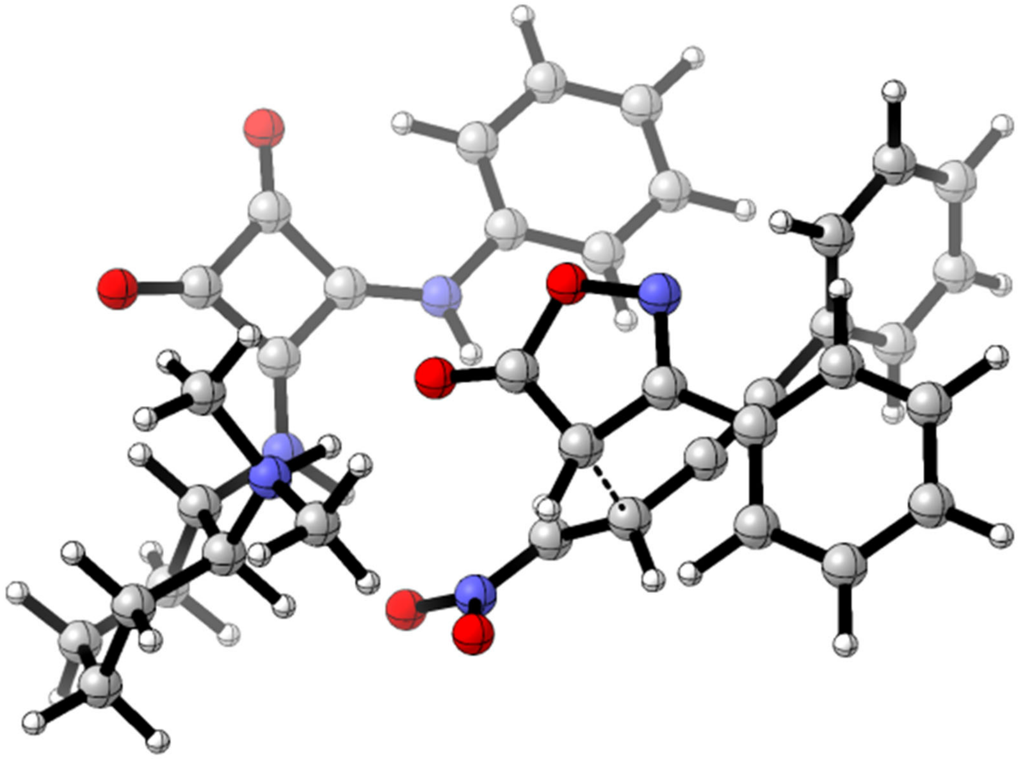

B2_TS

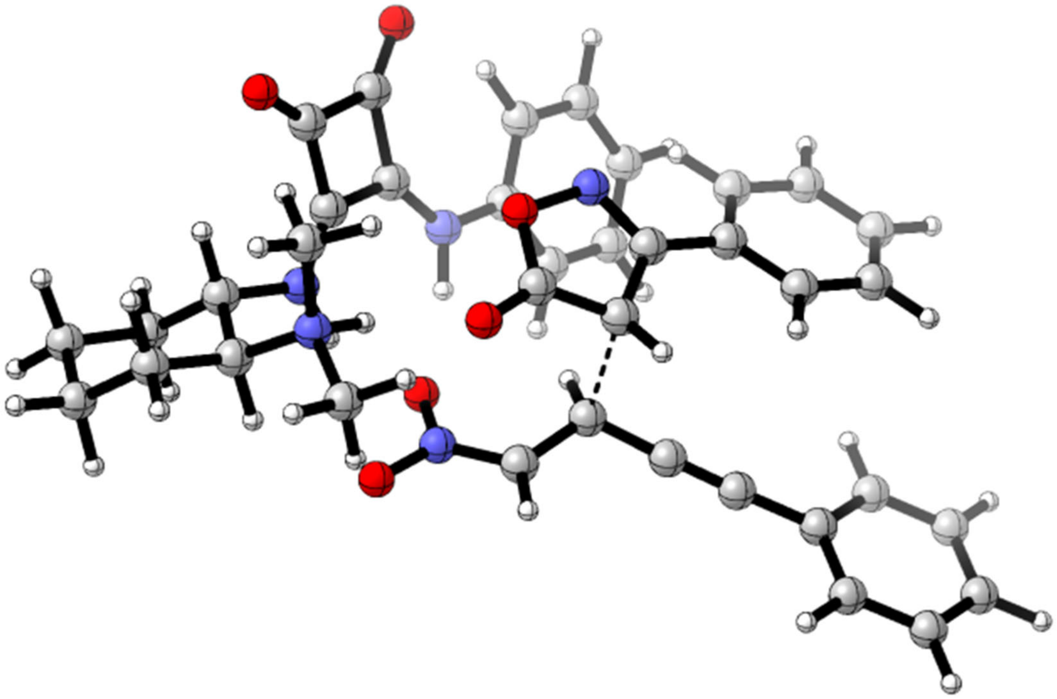

Cartesian Coordinates

A1_pre-TS

C	-1.56443800	2.55075600	0.25202000
C	-2.79305500	1.86854700	0.18118900
C	-3.24567400	2.38585200	1.48154600
C	-1.91826100	3.15748700	1.57584600
O	-1.38237600	3.88847600	2.38606100
O	-4.23463100	2.19038000	2.17859600
N	-0.53803900	2.49979700	-0.62080300
N	-3.29765500	0.99308400	-0.69683900
H	-2.70383900	0.73955600	-1.50685500
H	-0.69782900	1.87732700	-1.42887700
C	0.73096700	3.09064000	-0.55124000
C	1.59376500	2.86274200	-1.63619900

C	1.16482600	3.85467800	0.54185000
C	2.87281300	3.40823400	-1.63392300
C	2.45152300	4.38853500	0.52685700
C	3.31081700	4.17726300	-0.55311300
H	1.24397700	2.26799200	-2.47479200
H	0.50012700	4.03300300	1.38120100
H	3.52679300	3.23420300	-2.48334600
H	2.78185200	4.98281000	1.37338100
H	4.30465000	4.61416000	-0.55582700
C	-4.64108000	0.45362500	-0.59145400
C	-4.60236100	-1.08273300	-0.45593000
C	-6.01087300	-1.67036800	-0.35478100
C	-6.83452600	-1.28558200	-1.59243100
C	-6.88754800	0.23550500	-1.76822800
C	-5.47758800	0.82977900	-1.82641400
H	-5.10481100	0.89237700	0.29685300
H	-4.09193100	-1.48185700	-1.33811800
H	-5.97045500	-2.75939500	-0.25844800
H	-6.50969600	-1.28116900	0.54061000
H	-7.84317900	-1.70067200	-1.50091800
H	-6.38369400	-1.74292800	-2.48282600
H	-7.44215400	0.49482500	-2.67579000
H	-7.43489700	0.67959300	-0.92627300
H	-5.51071300	1.92091400	-1.89420700
H	-4.95561000	0.47125700	-2.72372700
N	-3.70550400	-1.50587900	0.69212500

C	-3.37171700	-2.96544400	0.64770700
H	-4.26698200	-3.54295600	0.87635800
H	-2.97609500	-3.20624300	-0.33597200
H	-2.59699600	-3.14364700	1.39070700
C	-4.18226800	-1.11489800	2.05764400
H	-3.38082400	-1.36614700	2.75039000
H	-4.37225900	-0.04189400	2.09863800
H	-5.08281900	-1.68034800	2.29495600
C	1.38643400	0.06207000	1.09802100
H	0.70319000	0.77957000	0.65832800
C	0.88116400	-0.94791900	1.84667000
H	1.44875300	-1.73089400	2.32339400
N	-0.51792800	-1.10439300	1.96410000
O	-0.94005800	-2.06010800	2.63712800
O	-1.27116800	-0.28631400	1.39361200
H	-2.80322800	-1.00831800	0.56395300
C	2.75388900	0.18904300	0.84669800
C	3.93798700	0.26451700	0.57186300
C	5.31591600	0.29556100	0.24767700
C	6.13369900	-0.81234700	0.55122700
C	5.87589200	1.41946200	-0.39109200
C	7.48213900	-0.79216500	0.21391700
H	5.69066500	-1.67521600	1.03477200
C	7.22679300	1.42851700	-0.71920500
H	5.23634700	2.26554900	-0.61206400
C	8.03107600	0.32568200	-0.42019100

H	8.10867000	-1.64751800	0.44625100
H	7.65604900	2.29647400	-1.20967000
H	9.08504400	0.33763600	-0.67990100
C	-0.64624500	-0.52250300	-1.92746300
O	-1.42548100	-1.52063500	-1.35197900
C	0.64659000	-2.14805200	-1.10241700
C	0.68563000	-0.91195100	-1.78139800
O	-1.22705900	0.48973600	-2.41006400
N	-0.58797100	-2.54493500	-0.82337200
C	1.79371600	-2.94140600	-0.63089400
C	3.07014200	-2.73507000	-1.16985200
C	1.63542100	-3.88028300	0.40101400
C	4.16540600	-3.45294800	-0.69292600
H	3.20373200	-2.01261200	-1.96742300
C	2.73129100	-4.59276600	0.87997000
H	0.64982900	-4.02269900	0.83034500
C	4.00088800	-4.38182000	0.33548700
H	5.14723500	-3.28152800	-1.12284300
H	2.59676400	-5.31161900	1.68268600
H	4.85432100	-4.93952400	0.70943400
H	1.54697300	-0.34750800	-2.09600500

A1_TS

C	-1.83447500	2.70413400	0.02926200
C	-2.99119100	1.90759500	-0.00662300
C	-3.58552800	2.58063800	1.16021400

C	-2.34256000	3.48661500	1.20137900
O	-1.94504600	4.40241500	1.89342500
O	-4.60713000	2.41023200	1.81284000
N	-0.74668900	2.63997700	-0.76717400
N	-3.38720000	0.88319900	-0.77483700
H	-2.72768300	0.51368700	-1.46207900
H	-0.76583700	1.88204500	-1.44998800
C	0.48627600	3.30467100	-0.67001500
C	1.47071200	2.94674600	-1.60517900
C	0.76791000	4.26505100	0.31093900
C	2.72604500	3.54422700	-1.56055400
C	2.03112400	4.85162800	0.33948500
C	3.01582100	4.50149100	-0.58704500
H	1.24003800	2.20200600	-2.36199400
H	0.00939100	4.54421800	1.03507600
H	3.47730100	3.25928400	-2.29104100
H	2.24535700	5.59472500	1.10151900
H	3.99304600	4.97271300	-0.55321000
C	-4.66900200	0.21760900	-0.60353300
C	-4.48164600	-1.26324700	-0.21605100
C	-5.83284100	-1.96877400	-0.06409200
C	-6.64595700	-1.87253100	-1.36151600
C	-6.84420200	-0.41190800	-1.77507300
C	-5.49756300	0.30654200	-1.89785700
H	-5.20169700	0.74503100	0.19264800
H	-3.90417900	-1.74324000	-1.01434200

H	-5.68848900	-3.01835200	0.20712500
H	-6.39882600	-1.49781900	0.74829800
H	-7.61073600	-2.37155800	-1.22572800
H	-6.12017900	-2.41283500	-2.15983200
H	-7.38856800	-0.35189000	-2.72306200
H	-7.46143600	0.09826600	-1.02384600
H	-5.63374800	1.36440700	-2.14025100
H	-4.91414400	-0.13499900	-2.71761600
N	-3.60983600	-1.42688700	1.00845000
C	-3.15699500	-2.84196200	1.18566400
H	-3.99956900	-3.46714400	1.48029500
H	-2.72155000	-3.19028700	0.25023700
H	-2.39165200	-2.83455900	1.96081300
C	-4.17313600	-0.88782500	2.28463300
H	-3.35888400	-0.91774400	3.00892500
H	-4.49510700	0.14463200	2.14626900
H	-5.00512800	-1.51101800	2.61382900
C	1.30398900	-0.08301800	0.39054600
H	0.70712200	0.77714300	0.10291200
C	0.82130400	-0.78222800	1.53897400
H	1.44144400	-1.36928200	2.19682500
N	-0.49807200	-0.80255500	1.79249000
O	-0.97268200	-1.39870600	2.79778300
O	-1.28418800	-0.19337400	0.95637000
H	-2.69645100	-0.87297500	0.87734000
C	2.71558600	0.12724300	0.33791900

C	3.91549700	0.29654800	0.27799000
C	5.32401600	0.48688700	0.21190700
C	6.19777100	-0.55976200	0.56309900
C	5.85682700	1.71975000	-0.20891700
C	7.57445600	-0.37557900	0.48248800
H	5.78087100	-1.50461300	0.89329200
C	7.23522200	1.89387700	-0.28159900
H	5.17615500	2.52500700	-0.46090200
C	8.09677700	0.84882700	0.05965000
H	8.24280000	-1.18684900	0.75426700
H	7.64024100	2.84823100	-0.60366500
H	9.17153800	0.98945800	0.00054900
C	-0.45210800	-0.81666700	-1.67840500
O	-1.17216300	-1.94247700	-1.37719600
C	0.92035500	-2.47742300	-0.95370900
C	0.93516000	-1.06818400	-1.30921600
O	-1.00585100	0.16262200	-2.17879200
N	-0.28725700	-2.97066400	-0.90852200
C	2.06993800	-3.27589600	-0.51378800
C	3.36938400	-2.91826200	-0.89881900
C	1.88094000	-4.39824800	0.30871000
C	4.46052800	-3.67627000	-0.47807700
H	3.52974500	-2.04989600	-1.52631800
C	2.97301400	-5.14848900	0.73062700
H	0.87478500	-4.65913500	0.61647100
C	4.26570700	-4.79030900	0.33835900

H	5.46088800	-3.39230900	-0.78841800
H	2.81884700	-6.00997800	1.37265300
H	5.11660900	-5.37699800	0.67048700
H	1.71461900	-0.61555000	-1.90648400

A1_post-TS

C	-1.41075500	2.85944800	0.15969000
C	-2.63522600	2.17930000	0.03940700
C	-3.22691800	2.88387300	1.19077600
C	-1.90555100	3.65367100	1.33153400
O	-1.46728100	4.51278300	2.06964400
O	-4.30203800	2.81887100	1.77181200
N	-0.29465600	2.73559800	-0.58812000
N	-3.10958700	1.24296700	-0.79518500
H	-2.48470700	0.85759200	-1.49877700
H	-0.33995000	2.01755100	-1.30759600
C	0.97839200	3.30642300	-0.41279100
C	1.98534200	2.89325400	-1.29959100
C	1.27192900	4.23028800	0.59853700
C	3.27691300	3.39306200	-1.17208400
C	2.57017500	4.72406100	0.70713600
C	3.57854600	4.31408200	-0.16762000
H	1.74981600	2.17459500	-2.07935300
H	0.49777100	4.55209100	1.28707400
H	4.04953200	3.05416000	-1.85477100
H	2.79239700	5.44044500	1.49222100

H	4.58486400	4.70921800	-0.07084000
C	-4.44739300	0.68241900	-0.66849300
C	-4.41569900	-0.81635600	-0.29530000
C	-5.83835400	-1.37471000	-0.17650500
C	-6.61447100	-1.18081500	-1.48601900
C	-6.64414100	0.29486000	-1.89426700
C	-5.22641700	0.86534900	-1.98172800
H	-4.95233100	1.24720900	0.11976400
H	-3.88169800	-1.34406400	-1.09452700
H	-5.80909300	-2.43658500	0.08391500
H	-6.36394800	-0.85667600	0.63479000
H	-7.63069200	-1.57132400	-1.37085900
H	-6.13721200	-1.77099500	-2.27967700
H	-7.15790300	0.41691600	-2.85342000
H	-7.21893000	0.86614200	-1.15345600
H	-5.24498700	1.93291200	-2.21935300
H	-4.67374600	0.36813400	-2.79125900
N	-3.58498300	-1.08226200	0.93532500
C	-3.31010500	-2.53690000	1.13091000
H	-4.21324800	-3.05334600	1.45833800
H	-2.94529000	-2.95637600	0.19294200
H	-2.52797000	-2.60401800	1.88828800
C	-4.07971200	-0.47311700	2.20480400
H	-3.25981500	-0.55900200	2.91885100
H	-4.32036100	0.57888100	2.05591500
H	-4.95791400	-1.01524100	2.55942800

C	1.39707400	-0.40747600	0.00709000
H	0.98881900	0.59033000	-0.18806500
C	0.90641800	-0.85830100	1.34915000
H	1.54189300	-1.24245600	2.12974200
N	-0.38804500	-0.81465900	1.57594500
O	-0.95038700	-1.19461700	2.64767800
O	-1.14373500	-0.35231300	0.57835700
H	-2.57259600	-0.67496300	0.77882300
C	2.85051900	-0.28591300	-0.04194900
C	4.04794800	-0.11788500	-0.11400500
C	5.45020300	0.13648700	-0.18478400
C	6.33782800	-0.83604000	-0.67851600
C	5.95755100	1.38000400	0.23677200
C	7.70171000	-0.56823100	-0.75043900
H	5.94690600	-1.79440200	-1.00469500
C	7.32290400	1.63773800	0.16124700
H	5.26877000	2.12898000	0.61056300
C	8.19819400	0.66763400	-0.33153700
H	8.37918200	-1.32490300	-1.13393400
H	7.70598100	2.59944800	0.48846300
H	9.26246800	0.87350900	-0.38884600
C	-0.43047300	-0.74432500	-1.78520200
O	-1.33825500	-1.75300300	-1.87993500
C	0.38714900	-2.68935100	-0.85553100
C	0.84187100	-1.29302500	-1.18504000
O	-0.67274900	0.35810000	-2.24180800

N	-0.81727800	-2.93653900	-1.25241800
C	1.16447100	-3.71731400	-0.15869700
C	2.55524700	-3.59154200	-0.03272200
C	0.52036600	-4.83939500	0.38927400
C	3.28862300	-4.57198400	0.63121300
H	3.06376400	-2.72442600	-0.43681600
C	1.25814200	-5.81497100	1.04809800
H	-0.55654400	-4.92474900	0.30134400
C	2.64437600	-5.68427000	1.17176700
H	4.36371700	-4.46145000	0.73000700
H	0.75287600	-6.67511300	1.47557000
H	3.21694900	-6.44425100	1.69411800
H	1.60057600	-1.30827100	-1.97623200

A2_pre-TS

C	-1.82702900	-2.54485000	0.34100500
C	-2.89409100	-1.62236600	0.40017100
C	-3.89331300	-2.62419400	0.02214500
C	-2.75907000	-3.65105300	-0.07866800
O	-2.66104200	-4.82007700	-0.39127200
O	-5.11173800	-2.60442300	-0.14340400
N	-0.52749400	-2.35113200	0.60900200
N	-2.96268000	-0.29540900	0.63201900
H	-2.08917200	0.16074900	0.93607000
H	-0.31242200	-1.42444800	1.00906200
C	0.56601600	-3.22874100	0.47606300

C	1.81162700	-2.75583200	0.91903800
C	0.45656700	-4.50664000	-0.08768700
C	2.93959600	-3.56174600	0.80058300
C	1.60000400	-5.29594900	-0.20101700
C	2.84271600	-4.83454700	0.23603900
H	1.89234000	-1.76361800	1.35160500
H	-0.50740100	-4.88166400	-0.41386700
H	3.89692500	-3.18643100	1.14571800
H	1.51109800	-6.28698800	-0.63634600
H	3.72548900	-5.45882700	0.13998100
C	-4.21807100	0.35370000	0.97751800
C	-4.52884100	1.50189700	-0.00448300
C	-5.82046800	2.23313500	0.36560300
C	-5.72649600	2.78608500	1.79531800
C	-5.42930000	1.66595500	2.79730300
C	-4.15643400	0.90874100	2.41093700
H	-5.01238500	-0.39593900	0.91964600
H	-3.68427900	2.19947500	0.02285200
H	-6.01978300	3.04779900	-0.33754400
H	-6.66632000	1.53801100	0.30867800
H	-6.66042700	3.29739500	2.04873700
H	-4.93008700	3.54043400	1.84035300
H	-5.32944400	2.07560200	3.80744700
H	-6.27653900	0.96796900	2.82223000
H	-3.97383600	0.07057600	3.08929700
H	-3.28377600	1.57052700	2.49079100

N	-4.52866200	1.00061100	-1.43898200
C	-4.48997600	2.11361100	-2.43276600
H	-5.44429500	2.63901700	-2.43684300
H	-3.66671700	2.77742600	-2.17958200
H	-4.30084800	1.67676300	-3.41307700
C	-5.61237400	0.02661800	-1.77856800
H	-5.42680600	-0.32306700	-2.79468800
H	-5.58241900	-0.83033000	-1.10195400
H	-6.57753700	0.53113200	-1.73682600
C	1.07421400	0.78688400	-1.64354300
H	0.98230700	1.85612200	-1.78764800
C	-0.03026600	-0.00324900	-1.80933500
H	-0.03290700	-1.08131300	-1.77995400
N	-1.28419600	0.57084100	-1.98024700
O	-2.23925100	-0.20519500	-2.27742300
O	-1.46389600	1.79622300	-1.80748400
H	-3.61529000	0.49321600	-1.58476800
C	2.32773800	0.23316200	-1.37795600
C	3.38311000	-0.25857700	-1.01900200
C	4.58524400	-0.88733900	-0.60238400
C	5.09164100	-1.98690200	-1.32092700
C	5.23955600	-0.44837000	0.56433500
C	6.24439900	-2.62855500	-0.88302700
H	4.56718300	-2.33140400	-2.20495200
C	6.38485100	-1.10997800	0.99682900
H	4.80454400	0.37401800	1.12231300

C	6.89132100	-2.19478200	0.27679200
H	6.63210800	-3.47635500	-1.43881400
H	6.88160100	-0.78122000	1.90430500
H	7.78455500	-2.70610800	0.62223700
C	0.56020000	1.00100300	1.36227600
O	1.77169200	0.57764400	1.86422900
C	2.15924700	2.49887700	0.90678400
C	0.77955900	2.23675500	0.73651300
O	-0.44514100	0.26190700	1.50946200
N	2.77433000	1.52805100	1.56406000
C	2.93437000	3.63523400	0.38542400
C	2.37248400	4.49998500	-0.56397800
C	4.25481500	3.85605300	0.80651500
C	3.11322400	5.55975800	-1.08436000
H	1.35284900	4.33955700	-0.90028600
C	4.99277100	4.91333700	0.28437300
H	4.68536900	3.19214400	1.54755200
C	4.42600900	5.76968100	-0.66305100
H	2.66510200	6.21967400	-1.82111900
H	6.01330000	5.07347900	0.61891000
H	5.00393000	6.59463200	-1.06820200
H	0.01659600	2.85182600	0.28998000

A2_TS

C	-1.73594200	-2.66278100	0.20855300
C	-2.80944500	-1.75482600	0.31606000

C	-3.79902000	-2.75181900	-0.11198400
C	-2.64929300	-3.75446100	-0.27281800
O	-2.53509400	-4.90468800	-0.64451300
O	-5.01726900	-2.74387200	-0.26184800
N	-0.43638500	-2.47865300	0.49920400
N	-2.89711700	-0.45251300	0.64139500
H	-2.03684500	0.00951200	0.94442300
H	-0.22383900	-1.58778800	0.95150900
C	0.66792800	-3.33553200	0.33121900
C	1.90525900	-2.86916400	0.80260700
C	0.57665900	-4.58799700	-0.28868700
C	3.04509400	-3.65275100	0.65436000
C	1.73068700	-5.35695600	-0.42959200
C	2.96558600	-4.90033100	0.03390500
H	1.97168100	-1.89699200	1.28168200
H	-0.38113600	-4.95748400	-0.63865900
H	3.99597100	-3.28085400	1.01984600
H	1.65654500	-6.32839400	-0.90940400
H	3.85647000	-5.50859700	-0.08581800
C	-4.16144300	0.18849400	0.97296000
C	-4.46069300	1.38189500	0.03810800
C	-5.75952700	2.08975500	0.43791100
C	-5.69322100	2.56982800	1.89376600
C	-5.40960700	1.40152900	2.84155400
C	-4.12809700	0.67202900	2.43385300
H	-4.94917300	-0.56234300	0.86697300

H	-3.61808400	2.07906200	0.10781500
H	-5.95150200	2.93954600	-0.22353200
H	-6.60295000	1.39717500	0.32997400
H	-6.63261300	3.06603700	2.15738300
H	-4.89941400	3.32223500	1.99124300
H	-5.32651000	1.75653000	3.87382800
H	-6.25329200	0.69918400	2.81632100
H	-3.94799300	-0.19781800	3.07202900
H	-3.26313000	1.33765500	2.55997600
N	-4.45318900	0.96631100	-1.41865700
C	-4.47210200	2.13536800	-2.34495200
H	-5.44499300	2.62570500	-2.31619700
H	-3.66568800	2.80897500	-2.06407500
H	-4.27510200	1.76008800	-3.34926400
C	-5.49670500	-0.02585100	-1.80986500
H	-5.28997300	-0.32621700	-2.83788100
H	-5.45095000	-0.90996600	-1.17260300
H	-6.48285400	0.43785800	-1.75988700
C	1.07860000	1.11662800	-1.25848400
H	1.06878300	2.09948200	-1.72505900
C	-0.01678700	0.26485700	-1.57071900
H	0.05657000	-0.80980900	-1.61005200
N	-1.23740700	0.78222200	-1.79499600
O	-2.16921900	-0.00508500	-2.21280400
O	-1.48640400	2.01018000	-1.57430800
H	-3.48779100	0.51439100	-1.62587000

C	2.34276300	0.48182900	-1.11429600
C	3.37906400	-0.08587900	-0.83643900
C	4.57094200	-0.77421200	-0.47562500
C	5.12347300	-1.74811700	-1.32565700
C	5.16772600	-0.51828100	0.77315900
C	6.25540700	-2.45372200	-0.93108300
H	4.64789800	-1.95220000	-2.27832400
C	6.29684100	-1.23544300	1.15830300
H	4.72083200	0.22658600	1.42295800
C	6.84266700	-2.20274100	0.31088200
H	6.67448100	-3.20848800	-1.58898100
H	6.74998800	-1.04234200	2.12570500
H	7.72109200	-2.76136600	0.61884400
C	0.62379900	0.91059600	1.45141800
O	1.81504600	0.72962300	2.09225800
C	2.06839200	2.56909700	0.91539700
C	0.76914300	2.04015800	0.57083900
O	-0.30977600	0.12006700	1.62089400
N	2.70974600	1.79372800	1.75309400
C	2.71772400	3.74236400	0.32107400
C	2.00934900	4.55965800	-0.57170200
C	4.05790500	4.04459300	0.61164700
C	2.62938800	5.65855400	-1.16417500
H	0.97192300	4.33973500	-0.80300500
C	4.67069600	5.14308800	0.02067500
H	4.60421600	3.40491500	1.29528600

C	3.95979600	5.95326100	-0.86972800
H	2.07197400	6.28290800	-1.85529600
H	5.70783300	5.36775600	0.24918400
H	4.44317900	6.80833500	-1.33187300
H	-0.09145000	2.60321700	0.23842000

A2_post-TS

C	-1.27621500	-2.92911100	0.08007500
C	-2.40871100	-2.11806200	0.28521900
C	-3.34175400	-3.19743200	-0.07338700
C	-2.12678800	-4.09212900	-0.34365600
O	-1.94652700	-5.22706600	-0.73535300
O	-4.56255100	-3.30075600	-0.11533500
N	0.02477900	-2.64083800	0.26944100
N	-2.58212800	-0.83956700	0.66026100
H	-1.75246900	-0.31740700	0.93079500
H	0.20874200	-1.73047400	0.68210100
C	1.16818800	-3.43948800	0.07263400
C	2.38797600	-2.91873500	0.52796100
C	1.12805200	-4.68971800	-0.55661200
C	3.56441700	-3.63920300	0.35012400
C	2.31619200	-5.39902100	-0.72314600
C	3.53538100	-4.88451600	-0.27904500
H	2.40863800	-1.95095600	1.01824300
H	0.18445800	-5.10536600	-0.89208200
H	4.50061700	-3.22235200	0.70439500

H	2.28173900	-6.36933600	-1.20950400
H	4.45238400	-5.44785100	-0.41967000
C	-3.88164700	-0.29768300	1.04160600
C	-4.33265300	0.86019900	0.12074000
C	-5.66800800	1.44555400	0.59683800
C	-5.57151200	1.93653900	2.04655200
C	-5.13564500	0.80302800	2.97714500
C	-3.81731100	0.19044500	2.50003700
H	-4.60250200	-1.11694500	0.97506800
H	-3.55327100	1.62953300	0.14629500
H	-5.97435200	2.27083500	-0.05194300
H	-6.44866500	0.67749900	0.53327300
H	-6.53712300	2.34636700	2.35981600
H	-4.84566200	2.75866300	2.10378600
H	-5.02941700	1.16598100	4.00462600
H	-5.91223900	0.02701100	2.99490800
H	-3.52389900	-0.65643600	3.12720000
H	-3.01341300	0.93579800	2.58161300
N	-4.36844600	0.45257700	-1.33382200
C	-4.57172000	1.61609000	-2.24269100
H	-5.58853800	2.00080700	-2.15624200
H	-3.82426400	2.36936100	-2.00314400
H	-4.39471800	1.26804100	-3.26076400
C	-5.32102000	-0.63921000	-1.67300600
H	-5.13264000	-0.92742700	-2.70836200
H	-5.16301300	-1.50953000	-1.03630500

H	-6.34875300	-0.28202200	-1.58144700
C	1.01340900	1.31296100	-1.01599800
H	1.04831800	2.16256300	-1.71148700
C	0.02356400	0.30799400	-1.51132700
H	0.28323800	-0.71368800	-1.73521700
N	-1.21985800	0.67388900	-1.73062600
O	-2.06917100	-0.18958400	-2.22853200
O	-1.63943900	1.86502700	-1.44152200
H	-3.33792000	0.11568200	-1.62446800
C	2.34159300	0.72783700	-0.86584300
C	3.41394400	0.23208700	-0.60167200
C	4.65288400	-0.37007400	-0.23561400
C	5.44222200	-1.04230900	-1.18393400
C	5.06899600	-0.32869600	1.10792600
C	6.62407000	-1.66365300	-0.79229900
H	5.11124400	-1.08216200	-2.21565000
C	6.25081600	-0.95646600	1.49022500
H	4.45075800	0.18773800	1.83479200
C	7.03042300	-1.62524300	0.54325300
H	7.22589400	-2.18629500	-1.52901000
H	6.56320600	-0.92645600	2.52931400
H	7.95020200	-2.11591400	0.84578200
C	0.70122400	0.98659500	1.49657300
O	1.64262800	1.44324100	2.35491100
C	1.50186900	3.03292500	0.82665500
C	0.57530300	1.95983600	0.36567100

O	0.11286000	-0.06287800	1.69316700
N	2.11509200	2.74011400	1.92662900
C	1.74463700	4.29147700	0.11951000
C	0.85584100	4.70760000	-0.88507700
C	2.85645200	5.08878800	0.43742800
C	1.07966300	5.90720700	-1.55835200
H	-0.00868900	4.10020000	-1.13665700
C	3.07062800	6.28413000	-0.23773500
H	3.54255700	4.75369500	1.20716400
C	2.18398100	6.69597200	-1.23732400
H	0.38979700	6.22385400	-2.33392100
H	3.93329300	6.89484600	0.00875600
H	2.35753000	7.62817300	-1.76578000
H	-0.46200500	2.27780400	0.20515600

A3_pre-TS

C	-1.58973700	-2.52868400	-0.19484400
C	-2.78058600	-1.79799200	-0.02530500
C	-3.39517100	-2.35188600	-1.23751200
C	-2.10746800	-3.16503200	-1.45440800
O	-1.68238200	-3.92434900	-2.30137200
O	-4.45983700	-2.16316100	-1.81828700
N	-0.48501500	-2.50421300	0.57203000
N	-3.14049100	-0.83123300	0.83449400
H	-2.45006000	-0.58191700	1.57568200
H	-0.57738100	-1.92968800	1.42340900

C	0.80695600	-2.98826800	0.29691500
C	1.80561600	-2.70575600	1.24269000
C	1.12740100	-3.66945100	-0.88549400
C	3.11597600	-3.10219600	1.00182900
C	2.44857000	-4.05402700	-1.10761300
C	3.44883500	-3.77280400	-0.17650300
H	1.55702900	-2.14838000	2.14005100
H	0.35554100	-3.89849000	-1.61269100
H	3.88459500	-2.86425600	1.72972900
H	2.69373600	-4.57837800	-2.02641500
H	4.47563700	-4.06810300	-0.36650100
C	-4.50053300	-0.32543400	0.89322800
C	-4.52096800	1.18167400	0.57175000
C	-5.93306800	1.76290500	0.63593100
C	-6.52684900	1.53397500	2.03379700
C	-6.52523600	0.04395100	2.39310300
C	-5.11770700	-0.55102200	2.28333400
H	-5.08986000	-0.86289800	0.14385300
H	-3.88706400	1.68094900	1.31364200
H	-5.92648900	2.83205800	0.39826300
H	-6.56799000	1.26654300	-0.10681200
H	-7.54258500	1.93979300	2.06758600
H	-5.93817000	2.09174000	2.77378700
H	-6.91477600	-0.10232700	3.40543700
H	-7.20128800	-0.49276500	1.71455800
H	-5.12756100	-1.62667600	2.48033600

H	-4.45650100	-0.09898800	3.03466200
N	-3.82722800	1.45469000	-0.74941800
C	-3.33835200	2.86231300	-0.85695700
H	-4.18878100	3.54159200	-0.91030300
H	-2.73037100	3.08940300	0.01980300
H	-2.73247600	2.93497800	-1.76010200
C	-4.58384400	1.05149500	-1.97958100
H	-3.90045500	1.18275900	-2.81829500
H	-4.85771400	-0.00269300	-1.90671900
H	-5.45744400	1.69204600	-2.09243600
C	1.13355300	-0.05934500	-1.37644200
H	0.46332600	-0.58018200	-0.70292400
C	0.58550400	0.71783500	-2.34848900
H	1.11972700	1.33160900	-3.05655800
N	-0.80607000	0.89972700	-2.36586100
O	-1.29077900	1.74711100	-3.13988600
O	-1.51270700	0.23948400	-1.55943500
H	-2.96721800	0.86510900	-0.78237400
C	2.49709500	-0.15534300	-1.11959900
C	3.64123200	-0.19710800	-0.70278400
C	4.92962800	-0.22567800	-0.11715600
C	5.03202000	-0.13500100	1.28721500
C	6.09193900	-0.33476500	-0.90180800
C	6.28769500	-0.15801300	1.88398100
H	4.12515300	-0.01054400	1.87128200
C	7.33940800	-0.37229800	-0.28757700

H	6.00174400	-0.39930800	-1.98066100
C	7.44000000	-0.28367700	1.10287700
H	6.36815200	-0.07756400	2.96341100
H	8.23525700	-0.46639000	-0.89309800
H	8.41639100	-0.30783000	1.57689100
C	-0.11785300	0.31439200	2.15024100
O	1.10573000	0.23486800	2.78123500
C	1.30881800	1.80538600	1.28706500
C	-0.02160900	1.32899500	1.19456400
O	-1.01653300	-0.51442900	2.48506200
N	2.00109700	1.17417800	2.22287400
C	1.96904700	2.81284700	0.44114000
C	1.25936000	3.47492000	-0.57017000
C	3.33607400	3.09134500	0.59715100
C	1.89841600	4.38238500	-1.41345300
H	0.20210300	3.27292600	-0.70644300
C	3.97294700	3.99732100	-0.24408200
H	3.88787400	2.57857600	1.37580300
C	3.25896500	4.64547600	-1.25531900
H	1.33279200	4.88132800	-2.19489600
H	5.03276700	4.19517400	-0.11478100
H	3.75932300	5.34927400	-1.91342800
H	-0.79737900	1.62269500	0.50958300

A3_TS

C	-1.70738000	-2.48472600	-0.09093200

C	-2.89058300	-1.72833100	0.01754900
C	-3.56510300	-2.48712700	-1.04774900
C	-2.27909400	-3.30936300	-1.20875900
O	-1.89509200	-4.20058600	-1.93768200
O	-4.67622900	-2.43621100	-1.56362300
N	-0.57762100	-2.40913600	0.63715100
N	-3.27595500	-0.69238000	0.78070500
H	-2.56377500	-0.21956300	1.33790100
H	-0.60591700	-1.73550600	1.40295700
C	0.68717600	-2.99134200	0.40963700
C	1.69566100	-2.69880200	1.33970200
C	0.96826100	-3.78595700	-0.70778700
C	2.98213800	-3.18828100	1.14386400
C	2.26444100	-4.26677100	-0.88545100
C	3.27763200	-3.96973100	0.02653400
H	1.47764800	-2.06715100	2.19491400
H	0.18672400	-4.03111600	-1.41823500
H	3.76137500	-2.93631300	1.85518800
H	2.48013100	-4.87869800	-1.75620500
H	4.28704600	-4.33369100	-0.13388300
C	-4.66132400	-0.24550800	0.82029300
C	-4.82693700	1.21810300	0.35836500
C	-6.29651200	1.64883200	0.41487900
C	-6.85800600	1.49207800	1.83336000
C	-6.69687600	0.05271000	2.32943900
C	-5.23500600	-0.39531000	2.24168100

H	-5.22078800	-0.90074500	0.14662800
H	-4.24207000	1.84585100	1.04262200
H	-6.40261300	2.68664500	0.08518900
H	-6.88137900	1.02815200	-0.27435500
H	-7.91088800	1.79108300	1.84499500
H	-6.32847100	2.17572200	2.51036400
H	-7.05593600	-0.04043700	3.35939700
H	-7.31794700	-0.61416800	1.71715000
H	-5.12467600	-1.44180900	2.53959600
H	-4.61992300	0.19689700	2.93330700
N	-4.20718300	1.48069800	-0.99321500
C	-4.09518500	2.93831600	-1.29310000
H	-5.06705500	3.34645600	-1.57125500
H	-3.71452700	3.45195300	-0.40909800
H	-3.37854300	3.03929900	-2.10897900
C	-4.81236500	0.75705700	-2.14962100
H	-4.13641500	0.90525500	-2.99309500
H	-4.89168700	-0.30856500	-1.93260800
H	-5.79393800	1.17488700	-2.37803500
C	0.84056800	0.40777400	-0.77744300
H	0.15696400	-0.33180900	-0.37360700
C	0.31493400	1.12413600	-1.89591000
H	0.90255600	1.54870900	-2.69394700
N	-1.01150300	1.33685800	-1.94217800
O	-1.58619300	1.90371500	-2.90989400
O	-1.69944400	0.94632700	-0.90213700

H	-3.16093100	1.16439000	-0.96119900
C	2.21014900	0.03368700	-0.77178700
C	3.37602300	-0.25908500	-0.60763900
C	4.72584700	-0.61322500	-0.33548100
C	5.23250200	-0.42798700	0.96558900
C	5.55064800	-1.16677200	-1.32929900
C	6.54250400	-0.79495600	1.25724000
H	4.58255200	-0.00232100	1.72302200
C	6.85824400	-1.53269500	-1.02370200
H	5.15250600	-1.31433800	-2.32714800
C	7.35770800	-1.34873700	0.26722200
H	6.92844400	-0.65091600	2.26179300
H	7.48935100	-1.96419200	-1.79448500
H	8.37828800	-1.63606500	0.50051800
C	0.28393700	0.65877300	1.92972200
O	1.42042500	0.46926000	2.66814000
C	1.93492400	2.06008500	1.23822500
C	0.60437200	1.61373100	0.88463500
O	-0.73125300	0.00004000	2.16777900
N	2.44257100	1.36916000	2.22520800
C	2.74474800	3.04432800	0.51309400
C	2.17945000	3.78122400	-0.53684300
C	4.09973900	3.23227300	0.83066900
C	2.95377200	4.68776400	-1.25840900
H	1.13694300	3.63829900	-0.79833100
C	4.86786400	4.13800800	0.10896700

H	4.53792000	2.65338900	1.63505000
C	4.29861000	4.86862300	-0.93797000
H	2.50577800	5.24987500	-2.07182600
H	5.91600100	4.27161900	0.35764000
H	4.90272800	5.57285700	-1.50152500
H	-0.18024000	2.23253800	0.47852900

A3_post-TS

C	-1.03482400	-2.40071800	-0.26369200
C	-2.28639200	-1.75196400	-0.30071500
C	-2.71375400	-2.47053500	-1.51261200
C	-1.35584800	-3.18488300	-1.50969900
O	-0.79723500	-4.00163500	-2.21082700
O	-3.72935200	-2.46613900	-2.19506600
N	-0.03903900	-2.30686400	0.63585500
N	-2.91178300	-0.80665700	0.43318300
H	-2.39383300	-0.36835000	1.19108700
H	-0.22504100	-1.69106100	1.42295400
C	1.24552400	-2.88924000	0.64164800
C	2.08243700	-2.56042900	1.71756000
C	1.70009800	-3.74643200	-0.36629900
C	3.36848300	-3.08586400	1.78147800
C	2.99135200	-4.26405800	-0.28132500
C	3.83195100	-3.94300900	0.78381900
H	1.73202900	-1.88216600	2.48955900
H	1.05643300	-4.00445000	-1.19939800

H	4.01275100	-2.81242000	2.61084800
H	3.34316100	-4.92304000	-1.06910600
H	4.84198200	-4.33550700	0.82417700
C	-4.37519000	-0.77022100	0.46052900
C	-4.95182400	0.65497600	0.36588300
C	-6.48548300	0.60502700	0.35502900
C	-7.02582900	-0.09692700	1.60786900
C	-6.44304100	-1.50537100	1.74839900
C	-4.91256600	-1.46480900	1.72516300
H	-4.71030600	-1.34077900	-0.40997400
H	-4.62000000	1.21851900	1.24812300
H	-6.89414600	1.61726100	0.29249600
H	-6.82160100	0.06499400	-0.53874800
H	-8.11907500	-0.13243600	1.56440300
H	-6.76544000	0.49734400	2.49409000
H	-6.79346200	-1.97499800	2.67320500
H	-6.79995200	-2.13229700	0.92086400
H	-4.49319800	-2.47484900	1.76099600
H	-4.53696500	-0.93193400	2.60989500
N	-4.38551100	1.42880800	-0.79327600
C	-4.84210900	2.84721800	-0.82869900
H	-5.85913700	2.91807900	-1.21692300
H	-4.79607200	3.25872700	0.18069000
H	-4.13677500	3.38124800	-1.46710900
C	-4.52708200	0.80718900	-2.13966900
H	-3.89995200	1.39112900	-2.81470100

H	-4.18668800	-0.22699500	-2.12738900
H	-5.56990600	0.84457400	-2.46233000
C	0.41439800	1.04523400	-0.34130100
H	-0.34836500	0.27602200	-0.17753100
C	-0.09570900	1.92192000	-1.43885200
H	0.46181300	2.17259300	-2.32673800
N	-1.35626000	2.30368900	-1.37536300
O	-1.98171300	2.90909100	-2.29802400
O	-2.00104500	2.02264100	-0.23788800
H	-3.29028100	1.57207000	-0.58560400
C	1.68840800	0.40353900	-0.63493400
C	2.77548400	-0.12130300	-0.72308300
C	4.06417500	-0.72572100	-0.77400500
C	4.97386300	-0.50055600	0.27501700
C	4.43351300	-1.56162100	-1.84018200
C	6.22841900	-1.10052000	0.25114200
H	4.67191800	0.12910500	1.10520100
C	5.69105600	-2.15710000	-1.85582100
H	3.72306400	-1.74544900	-2.63816800
C	6.59046200	-1.92983900	-0.81267300
H	6.92408200	-0.92566200	1.06621400
H	5.96815500	-2.80542900	-2.68128400
H	7.56988100	-2.39813900	-0.82787800
C	0.33973500	0.75337800	2.16813900
O	1.44836500	0.76664300	2.93922900
C	1.83851600	2.33400700	1.42349700

C	0.50050700	1.76990100	1.07354800
O	-0.57244200	-0.03281700	2.36696500
N	2.37488700	1.76983900	2.45647300
C	2.55750000	3.36431700	0.67179900
C	1.85939300	4.22601300	-0.18627700
C	3.95242200	3.48369500	0.78822900
C	2.54719300	5.19254400	-0.91726000
H	0.78410400	4.13792500	-0.28708800
C	4.63205000	4.45029800	0.05765100
H	4.48750800	2.80301400	1.44020500
C	3.93169000	5.30625200	-0.79768700
H	1.99944400	5.85307000	-1.58147900
H	5.71085300	4.53117500	0.14438000
H	4.46677700	6.05558100	-1.37269400
H	-0.33633000	2.47322300	1.10184400

A4_pre-TS

C	-2.14640800	2.50018000	-0.69640800
C	-3.19565200	1.56221000	-0.61310600
C	-4.17950700	2.58205900	-0.22428300
C	-3.05438900	3.62504900	-0.30061400
O	-2.94589600	4.81700100	-0.08762100
O	-5.37022300	2.55403200	0.07469700
N	-0.85744200	2.27956800	-1.00949000
N	-3.22161100	0.22737000	-0.73774800
H	-2.35229000	-0.22692400	-1.07757800

H	-0.64629300	1.31470100	-1.30824500
C	0.24674000	3.13818800	-0.92530200
C	1.48561800	2.61060800	-1.32449100
C	0.16946700	4.45156100	-0.43949400
C	2.63521900	3.38844400	-1.24302000
C	1.33343100	5.21393300	-0.36061300
C	2.56871100	4.69555700	-0.75603500
H	1.53230300	1.59375900	-1.70020700
H	-0.78825100	4.86916200	-0.14614900
H	3.58475700	2.96838000	-1.55900000
H	1.26807400	6.23136200	0.01321800
H	3.46500300	5.30499500	-0.69501900
C	-4.44069100	-0.55925800	-0.72153700
C	-4.43692900	-1.52337000	0.48348200
C	-5.68168800	-2.41054100	0.50918100
C	-5.77486300	-3.22103200	-0.79229000
C	-5.78217300	-2.29836800	-2.01484900
C	-4.55655100	-1.38080700	-2.01749500
H	-5.29132400	0.12518500	-0.64877300
H	-3.53499200	-2.13996300	0.40301500
H	-5.65867100	-3.08905200	1.36762500
H	-6.57916600	-1.78793200	0.60717700
H	-6.67599000	-3.84193200	-0.76890900
H	-4.91887800	-3.90522400	-0.85278700
H	-5.80791900	-2.88933500	-2.93586200
H	-6.69604500	-1.68926200	-2.00528900

H	-4.58653300	-0.68273000	-2.85894000
H	-3.63990400	-1.97453100	-2.13053700
N	-4.23237100	-0.76148800	1.78520800
C	-3.91343300	-1.66851900	2.92834800
H	-4.81091400	-2.20849300	3.22731500
H	-3.12141100	-2.34857100	2.62493000
H	-3.55802500	-1.05138500	3.75341900
C	-5.33263600	0.18246400	2.15518500
H	-5.01139600	0.72518100	3.04483100
H	-5.50995600	0.89953200	1.35170300
H	-6.23367700	-0.38769900	2.37962700
C	1.33470900	-0.34061100	1.30314200
H	1.16039200	-1.40907800	1.37801200
C	0.28358500	0.50586200	1.48851000
H	0.32709500	1.58342400	1.47956100
N	-0.99684000	-0.02046300	1.71467600
O	-1.91606700	0.79593500	1.99867200
O	-1.20551400	-1.24341400	1.63702400
H	-3.36967900	-0.18013900	1.65964700
C	2.64275600	0.12268200	1.12797500
C	3.78863800	0.50774000	0.98000100
C	5.11936500	0.95461600	0.79147400
C	5.40404900	2.33364000	0.71881800
C	6.16805900	0.02014700	0.67146000
C	6.71183400	2.76136900	0.51970500
H	4.59126700	3.04514200	0.80986200

C	7.47131800	0.46199400	0.47408600
H	5.93961300	-1.03780000	0.73269600
C	7.74615700	1.82985800	0.39605200
H	6.92726400	3.82353400	0.46130200
H	8.27634300	-0.26018300	0.38131000
H	8.76524400	2.16965600	0.24018100
C	-0.00958400	-1.34843800	-1.35699000
O	-0.56033800	-2.54103700	-0.93735600
C	1.60084300	-2.81086000	-0.85437700
C	1.38038200	-1.48946500	-1.31151100
O	-0.77864400	-0.39818600	-1.66535600
N	0.46741600	-3.45503700	-0.61367400
C	2.89263700	-3.46778200	-0.59208100
C	4.09808900	-2.80483600	-0.86258000
C	2.93915200	-4.76521300	-0.05855100
C	5.32058200	-3.42730100	-0.61363200
H	4.08137700	-1.80001000	-1.26970900
C	4.16075600	-5.38125600	0.19521500
H	2.00528300	-5.27472700	0.15083200
C	5.35771000	-4.71630300	-0.08148600
H	6.24484500	-2.90546400	-0.84446900
H	4.18065600	-6.38519600	0.60869400
H	6.30993600	-5.20048800	0.11254000
H	2.09943400	-0.74739600	-1.61064400

A4_TS

C	-2.39189600	2.47803400	-0.74747600
C	-3.42359400	1.53368200	-0.61472200
C	-4.39563600	2.54374100	-0.16688200
C	-3.27841800	3.59755200	-0.30508300
O	-3.15941300	4.78973000	-0.10441200
O	-5.56280600	2.50346400	0.20540000
N	-1.12722800	2.22798900	-1.13405300
N	-3.44815900	0.20185200	-0.78127500
H	-2.59637700	-0.26860500	-1.09272900
H	-0.99280000	1.23607600	-1.33641500
C	0.01014000	3.04109800	-1.08981700
C	1.22052000	2.47838400	-1.52362000
C	-0.01940200	4.35854800	-0.61066000
C	2.39569300	3.22012000	-1.46367600
C	1.16685200	5.08753200	-0.56291300
C	2.37812600	4.52971000	-0.97962200
H	1.22052000	1.46587700	-1.91081900
H	-0.95729100	4.80124400	-0.29000700
H	3.32717600	2.77136400	-1.79478600
H	1.13998400	6.10771400	-0.19218700
H	3.29373000	5.11121300	-0.93759800
C	-4.62034000	-0.62178900	-0.53863800
C	-4.42154400	-1.54130600	0.68805700
C	-5.64519300	-2.43511300	0.91220100
C	-5.92328000	-3.29002600	-0.33170400
C	-6.11811600	-2.40971500	-1.56920400

C	-4.91556200	-1.48562700	-1.77659600
H	-5.46324400	0.05317800	-0.36442900
H	-3.53512900	-2.15654900	0.49885300
H	-5.49373800	-3.08382300	1.77966900
H	-6.52262400	-1.81090500	1.12181000
H	-6.80568500	-3.91347600	-0.15607300
H	-5.07945300	-3.97254800	-0.49612200
H	-6.27047900	-3.02930000	-2.45875500
H	-7.02645300	-1.80477000	-1.44691400
H	-5.07377500	-0.81578800	-2.62663200
H	-4.02108800	-2.08170300	-2.00342900
N	-4.04158300	-0.75213400	1.92418800
C	-3.59634700	-1.63353800	3.04334400
H	-4.44423800	-2.18365300	3.45085200
H	-2.82147600	-2.29830600	2.66836200
H	-3.16568800	-0.99237400	3.81275500
C	-5.06469200	0.22065500	2.40688400
H	-4.60160400	0.81629400	3.19452500
H	-5.38011500	0.88768900	1.60486600
H	-5.92044500	-0.31922600	2.81431500
C	1.42869900	-0.63430400	0.88210800
H	1.29112600	-1.62422500	1.31020800
C	0.35727400	0.27057200	1.12682100
H	0.45847000	1.34309600	1.12595100
N	-0.87198100	-0.21418200	1.38853200
O	-1.79865100	0.61099700	1.74540900

O	-1.12323100	-1.45342900	1.29021800
H	-3.14645000	-0.18896100	1.69460000
C	2.73647400	-0.07034500	0.96295200
C	3.83310800	0.44965100	0.96794800
C	5.12701400	1.03984700	0.95594200
C	5.27353200	2.42421300	0.74351100
C	6.27341700	0.24297600	1.13962400
C	6.54338700	2.99160400	0.70874400
H	4.38702200	3.03305700	0.60440600
C	7.53783800	0.82226700	1.10356800
H	6.15332100	-0.82184800	1.30547700
C	7.67691800	2.19516400	0.88691100
H	6.65008500	4.05922500	0.54354100
H	8.41777400	0.20270100	1.24656600
H	8.66530600	2.64308900	0.85898000
C	0.04229700	-1.40985800	-1.40782500
O	-0.34518700	-2.71636000	-1.35361200
C	1.82001100	-2.74449900	-0.96909700
C	1.44383600	-1.34382000	-1.04661700
O	-0.75532700	-0.51609000	-1.69381900
N	0.78841600	-3.54023900	-1.08611600
C	3.15982800	-3.28918100	-0.70912200
C	4.29437900	-2.47568700	-0.83909000
C	3.32195500	-4.63426700	-0.33802400
C	5.56579200	-2.99634100	-0.60474900
H	4.18713700	-1.43545800	-1.12027900

C	4.59200700	-5.14866900	-0.10241400
H	2.44188600	-5.25923500	-0.23598000
C	5.71871400	-4.33209800	-0.23429200
H	6.43435500	-2.35489500	-0.71532700
H	4.70556700	-6.18849900	0.18799500
H	6.70908400	-4.73674700	-0.04971400
H	2.07356200	-0.57542200	-1.46858000

A4_post-TS

C	-1.78899500	2.88822100	-0.37291200
C	-2.90996000	2.03901000	-0.42251000
C	-3.80515200	3.05999300	0.14289800
C	-2.60265500	4.01337100	0.19125200
O	-2.40594700	5.15881800	0.54722700
O	-4.98614700	3.07564700	0.47168500
N	-0.51623800	2.62058400	-0.72140400
N	-3.08011400	0.76816700	-0.80888500
H	-2.28337600	0.27497300	-1.20620600
H	-0.34730400	1.67344500	-1.05324800
C	0.64142000	3.40312200	-0.58846400
C	1.84886100	2.82108500	-1.00623000
C	0.63594200	4.70093800	-0.06024400
C	3.04039200	3.53113700	-0.90059900
C	1.84036900	5.39447600	0.04248300
C	3.04517400	4.82292300	-0.37166400
H	1.84701200	1.81309900	-1.40898100

H	-0.29508600	5.15627400	0.26112300
H	3.96538500	3.06945700	-1.22863300
H	1.83062000	6.39975700	0.45307700
H	3.97476900	5.37687500	-0.28776500
C	-4.33193800	0.03999900	-0.68989800
C	-4.24150600	-1.07349500	0.37991900
C	-5.55361800	-1.85999000	0.46469600
C	-5.90107500	-2.48134000	-0.89487400
C	-5.98589300	-1.40664100	-1.98213300
C	-4.69174900	-0.59199900	-2.04552300
H	-5.10556700	0.76225500	-0.41424700
H	-3.42014300	-1.73748300	0.09101200
H	-5.48035100	-2.64629300	1.22151800
H	-6.36577700	-1.18901700	0.77115500
H	-6.84559500	-3.02947300	-0.81668400
H	-5.12939200	-3.21433100	-1.16373800
H	-6.18852700	-1.86314700	-2.95647600
H	-6.82849100	-0.73630900	-1.76626900
H	-4.76638400	0.21045200	-2.78515200
H	-3.85933700	-1.23795800	-2.35710600
N	-3.79750200	-0.53164200	1.71978900
C	-3.46064800	-1.61683100	2.68498300
H	-4.36213700	-2.13947900	3.00572200
H	-2.74782300	-2.28552600	2.20672900
H	-2.97763900	-1.14978200	3.54362400
C	-4.71690000	0.45602500	2.34971200

H	-4.20093600	0.87840600	3.21320300
H	-4.95712100	1.26358600	1.65894200
H	-5.62921400	-0.04281100	2.68133400
C	1.56766700	-1.00158200	0.51686100
H	1.56764200	-1.83367400	1.23538400
C	0.63522300	0.05280600	1.03023500
H	0.94858100	1.05020600	1.29121300
N	-0.61618700	-0.28135700	1.23916000
O	-1.45764700	0.55435900	1.78322400
O	-1.03765400	-1.45014200	0.86828400
H	-2.81926000	-0.03532800	1.58948800
C	2.91990600	-0.45284800	0.42236200
C	4.02649500	0.03173100	0.33054100
C	5.31677900	0.63503000	0.24179800
C	5.54103600	1.90331200	0.80983200
C	6.37326500	-0.01919000	-0.41646300
C	6.79505600	2.49881900	0.71438200
H	4.72247200	2.41230400	1.30539200
C	7.62435200	0.58405300	-0.50522100
H	6.20031200	-0.99643100	-0.85507200
C	7.83906900	1.84307000	0.05866600
H	6.95746100	3.47867000	1.15236500
H	8.43329000	0.07168600	-1.01659300
H	8.81548100	2.31171200	-0.01364200
C	-0.15472500	-1.23324300	-1.47000900
O	-0.87808900	-2.33060500	-1.81037100

C	0.90965800	-3.16217800	-0.82218300
C	1.15146600	-1.67966100	-0.86477700
O	-0.52691800	-0.11377500	-1.77362500
N	-0.21125900	-3.51348600	-1.35846600
C	1.81715300	-4.16576100	-0.25758600
C	3.15199000	-3.83808800	0.02512600
C	1.35848000	-5.46913300	0.00077100
C	4.01207500	-4.79861700	0.55419500
H	3.52294600	-2.83652600	-0.16321200
C	2.22243000	-6.42234500	0.52520400
H	0.32355400	-5.71288900	-0.21053600
C	3.55166800	-6.09029500	0.80464800
H	5.04177300	-4.53366700	0.77182300
H	1.85970700	-7.42582900	0.72410900
H	4.22217200	-6.83597700	1.22025200
H	1.93792900	-1.44680500	-1.59228100

B1_pre-TS

C	2.32176600	2.48648000	-0.43064200
C	3.36480300	1.55349100	-0.30175600
C	4.13163800	2.13935900	-1.41414000
C	3.06584300	3.25159900	-1.48864500
O	2.91547300	4.28265600	-2.10917500
O	5.11212400	1.77824700	-2.05261000
N	1.11466000	2.47410600	0.17202500
N	3.60151000	0.52286000	0.52174000

H	2.94873900	0.30702700	1.27062200
H	0.81722100	1.53499800	0.43240100
C	0.12199200	3.46802500	0.19491300
C	-1.07752700	3.16889100	0.85903600
C	0.30686800	4.73246900	-0.37825400
C	-2.07767500	4.13148200	0.95582200
C	-0.70961900	5.68063400	-0.27797900
C	-1.90133000	5.39483900	0.38884100
H	-1.22647900	2.18111400	1.28403100
H	1.21908600	4.96102200	-0.91811300
H	-2.99787200	3.89054000	1.47931700
H	-0.56153300	6.65647600	-0.73060200
H	-2.68211600	6.14463600	0.46558900
C	4.68696200	-0.42192600	0.30930300
C	4.13492200	-1.83209100	0.01714300
C	5.27222000	-2.80374500	-0.29842500
C	6.23455200	-2.87773800	0.89806900
C	6.77022500	-1.49108300	1.27213200
C	5.62739700	-0.50078700	1.52216400
H	5.26036700	-0.05781900	-0.54822500
H	3.59651700	-2.17182700	0.90803700
H	4.88359100	-3.80251600	-0.51783000
H	5.81924900	-2.46141900	-1.18565000
H	7.05829800	-3.55898600	0.66232900
H	5.70153700	-3.30793700	1.75566500
H	7.40748700	-1.55946000	2.15965400

H	7.40416400	-1.11515700	0.45801300
H	6.01542700	0.50274300	1.71927900
H	5.04387800	-0.80340100	2.39989300
N	3.06244500	-1.78708200	-1.05574200
C	2.35398500	-3.09213200	-1.19523400
H	3.02090300	-3.84346000	-1.61683700
H	2.00059400	-3.39277000	-0.20887300
H	1.50165300	-2.94009700	-1.85703900
C	3.51131300	-1.27859700	-2.38416300
H	2.62177800	-1.10762200	-2.99144500
H	4.05876400	-0.34353700	-2.27651000
H	4.14646400	-2.02351800	-2.86518900
C	-1.06987600	-1.47981500	2.15339600
H	-1.04263100	-2.55038200	1.97343900
C	0.09775900	-0.85907600	2.45821800
H	0.21314600	0.18610700	2.69845700
N	1.31865400	-1.56395500	2.38139500
O	2.35908100	-0.93294900	2.67782100
O	1.34031500	-2.75350900	2.02203800
C	-2.29604700	-0.81955300	2.03257700
C	-3.35578800	-0.30970700	1.70940800
C	-4.59729000	0.25342700	1.31696100
C	-5.80090100	-0.41606900	1.60564200
C	-4.62528200	1.45127700	0.57671500
C	-7.01148200	0.11143900	1.16950800
H	-5.76954900	-1.34835000	2.15833400

C	-5.84136400	1.96530000	0.14165100
H	-3.69231800	1.93057400	0.31065400
C	-7.03486800	1.30072700	0.43762100
H	-7.93709600	-0.40943400	1.39272200
H	-5.85694200	2.88173000	-0.43944900
H	-7.98105200	1.70596500	0.09250700
C	-0.18569700	-0.67530900	-0.61256300
O	-1.01654900	0.39166700	-0.84155600
C	-2.22982100	-1.38707700	-1.14950800
C	-0.92756000	-1.83755500	-0.81043300
O	0.99778400	-0.41308300	-0.22143400
N	-2.30541300	-0.06585400	-1.17196000
H	-0.57906900	-2.84745800	-0.67815200
H	2.28027600	-1.13429700	-0.69932800
C	-3.44611400	-2.18508600	-1.37234100
C	-4.68334500	-1.55109000	-1.57003900
C	-3.39879600	-3.58519500	-1.35060000
C	-5.84146000	-2.30360500	-1.73263300
H	-4.72323200	-0.46884000	-1.57938100
C	-4.56029900	-4.33717600	-1.51783500
H	-2.45003400	-4.09028800	-1.20035000
C	-5.78629600	-3.69926000	-1.70692100
H	-6.79157200	-1.79737200	-1.87241600
H	-4.50731400	-5.42157300	-1.49781700
H	-6.69199800	-4.28477000	-1.83264800

B1_TS

C	1.94906800	2.62355400	-0.25684200
C	3.06499700	1.78213100	-0.09498500
C	3.83853800	2.48896900	-1.13003200
C	2.68167100	3.49247100	-1.24028000
O	2.47103400	4.52184600	-1.84680200
O	4.89422300	2.26054000	-1.70973200
N	0.71358400	2.50565200	0.26869100
N	3.34358500	0.71086100	0.66101800
H	2.71549200	0.36492900	1.39247500
H	0.49849200	1.57950900	0.61952600
C	-0.39466000	3.36995100	0.15897500
C	-1.61543000	2.91760200	0.67771000
C	-0.30315200	4.64312000	-0.41608700
C	-2.73987800	3.73622400	0.62783400
C	-1.44077400	5.44645300	-0.46307200
C	-2.65858500	5.00703100	0.05774600
H	-1.68582700	1.92391700	1.10919500
H	0.63112000	4.98785700	-0.84393600
H	-3.67830200	3.37501500	1.03284800
H	-1.36707100	6.43066800	-0.91587800
H	-3.53555900	5.64510900	0.01705500
C	4.59724100	-0.02204400	0.52797500
C	4.32003000	-1.47976200	0.11377300
C	5.62327600	-2.24021800	-0.12448600
C	6.45473000	-2.23402300	1.16891100

C	6.71784700	-0.80527500	1.65893800
C	5.40894200	-0.02569100	1.83171800
H	5.18328700	0.48410000	-0.24417800
H	3.74644800	-1.94808400	0.91981700
H	5.42546100	-3.27256300	-0.43005800
H	6.19777800	-1.76254900	-0.92788900
H	7.39774700	-2.76272800	0.99738900
H	5.91191500	-2.79173200	1.94224900
H	7.26818500	-0.82724400	2.60501800
H	7.35781200	-0.28502200	0.93378400
H	5.60732600	1.01194000	2.11613000
H	4.79911700	-0.47397000	2.62418300
N	3.36749600	-1.51482000	-1.07066300
C	2.82184500	-2.88336600	-1.31384700
H	3.61275800	-3.53394000	-1.68527300
H	2.41125600	-3.24874400	-0.37196700
H	2.03173900	-2.80056200	-2.06076300
C	3.89005000	-0.90641500	-2.33205700
H	3.05318800	-0.81677300	-3.02490300
H	4.30624800	0.08198600	-2.13870700
H	4.65420400	-1.55890500	-2.75416600
C	-1.08524200	-1.63866400	1.36873700
H	-1.19750600	-2.70234100	1.57327900
C	0.06820300	-1.02203500	1.93946400
H	0.08218100	-0.00517900	2.29905000
N	1.24893300	-1.67898000	1.97192800

O	2.27512700	-1.09299600	2.45961500
O	1.34234400	-2.85400700	1.49648900
C	-2.28889200	-0.86804500	1.40917300
C	-3.29078900	-0.19661100	1.27610600
C	-4.43609500	0.62382600	1.06332300
C	-5.20193700	1.09644000	2.14326500
C	-4.77629300	1.00096400	-0.25009400
C	-6.28872100	1.93486800	1.91085600
H	-4.93152600	0.80794000	3.15323900
C	-5.86691700	1.83652000	-0.46866400
H	-4.16198600	0.64776800	-1.07112300
C	-6.62326100	2.30700000	0.60688800
H	-6.87368100	2.30136900	2.74857200
H	-6.11938300	2.13235500	-1.48198200
H	-7.46861700	2.96487700	0.43084100
C	-0.19800300	-0.77995300	-1.05333100
O	-1.16628400	-0.08687700	-1.71305400
C	-2.09455300	-2.01349300	-1.19325500
C	-0.78703800	-2.00864600	-0.56534400
O	0.91888500	-0.28895600	-0.87090100
N	-2.34591100	-0.89094200	-1.81338200
H	-0.16329800	-2.87845500	-0.41545100
H	2.53110000	-0.95095800	-0.79423300
C	-3.12584800	-3.04561100	-1.05157700
C	-4.44696000	-2.79860400	-1.45937900
C	-2.80799900	-4.28159300	-0.47123300

C	-5.42334200	-3.77313100	-1.29179000
H	-4.69372000	-1.83619700	-1.89214300
C	-3.79082300	-5.25546600	-0.30405800
H	-1.79097800	-4.48569700	-0.15185000
C	-5.09951000	-5.00429000	-0.71410000
H	-6.44288700	-3.57173700	-1.60508800
H	-3.53340800	-6.20838100	0.14731300
H	-5.86595900	-5.76148600	-0.58169600

B1_post-TS

C	1.29973300	2.66525500	-0.13510500
C	2.54000900	2.00053900	-0.10103600
C	3.06431300	2.77423400	-1.24106600
C	1.74383100	3.55225200	-1.26071300
O	1.27549500	4.46179000	-1.91544100
O	4.08985400	2.72150100	-1.91067300
N	0.19253100	2.43579000	0.59878000
N	3.05528700	1.00913900	0.63613100
H	2.57177700	0.57610400	1.44270000
H	0.19996500	1.57263600	1.13101400
C	-0.99261700	3.19359700	0.69880600
C	-1.86279100	2.89105000	1.75407700
C	-1.31042000	4.22109400	-0.19821000
C	-3.05063300	3.59886400	1.90438600
C	-2.50005700	4.92588500	-0.02579800
C	-3.37774000	4.62131500	1.01521900

H	-1.61588800	2.09080700	2.44451300
H	-0.63437900	4.46855700	-1.00924300
H	-3.72534300	3.34057400	2.71355000
H	-2.74268500	5.72029700	-0.72515700
H	-4.30938900	5.16557600	1.12632600
C	4.41043400	0.51113500	0.42619900
C	4.36342700	-0.97201200	0.01634700
C	5.76055900	-1.52082600	-0.26244800
C	6.62406900	-1.37638800	1.00101500
C	6.67455600	0.08056800	1.47515700
C	5.26562100	0.64318600	1.69579200
H	4.86277700	1.10900500	-0.36967800
H	3.88389500	-1.52327100	0.83248600
H	5.71578000	-2.57274200	-0.56181600
H	6.22702900	-0.96344600	-1.08423100
H	7.63205900	-1.75191800	0.79797100
H	6.20194100	-2.00536900	1.79472800
H	7.25537400	0.15570000	2.40009900
H	7.19751500	0.68856100	0.72463800
H	5.30592500	1.70010100	1.97578200
H	4.76242200	0.10586800	2.50757700
N	3.40261100	-1.15487600	-1.15088600
C	3.05944500	-2.59171100	-1.37261500
H	3.93529000	-3.11843500	-1.74909000
H	2.71938900	-2.99767300	-0.41983800
H	2.26176900	-2.63611500	-2.11465800

C	3.80800300	-0.49639600	-2.43184500
H	2.95598800	-0.55047200	-3.10930500
H	4.06327800	0.54989800	-2.26319300
H	4.65683500	-1.03414900	-2.85347900
C	-0.95138700	-1.59698600	1.12351300
H	-1.23369000	-2.51325700	1.66279200
C	0.17458800	-0.90107200	1.82134500
H	0.00768300	-0.01097300	2.40778700
N	1.38456400	-1.43819100	1.91211800
O	2.33361300	-0.78927300	2.50544100
O	1.64743100	-2.58578300	1.38466200
C	-2.11351600	-0.71894700	1.00071100
C	-3.05066100	-0.00899900	0.70940900
C	-4.11330400	0.86082800	0.32748000
C	-5.26001300	1.00531200	1.12630200
C	-3.99657000	1.60532500	-0.85936400
C	-6.26668500	1.88771200	0.74597200
H	-5.34480200	0.42913100	2.04152700
C	-5.00676600	2.48578700	-1.22919300
H	-3.10572100	1.49303600	-1.46673900
C	-6.14222700	2.63031300	-0.42968300
H	-7.14925300	1.99828000	1.36851900
H	-4.90359800	3.06752200	-2.13937500
H	-6.92717300	3.32149000	-0.72080300
C	-0.20107100	-1.01449600	-1.27701800
O	-1.13755700	-0.95792600	-2.25524100

C	-1.74706000	-2.69814600	-1.03997200
C	-0.57290800	-2.11636900	-0.32713900
O	0.76091500	-0.26787000	-1.27792900
N	-2.07818000	-2.04153300	-2.10247900
H	0.27525300	-2.78784300	-0.16396300
H	2.51456700	-0.69990400	-0.86010700
C	-2.51866900	-3.85689600	-0.58663400
C	-3.82863000	-4.06659900	-1.04714500
C	-1.94958600	-4.76434200	0.32026200
C	-4.55110700	-5.17015300	-0.60947100
H	-4.26490200	-3.35060500	-1.73440100
C	-2.68071200	-5.86748500	0.75639000
H	-0.93537300	-4.61425700	0.67737400
C	-3.98019500	-6.07218400	0.29327000
H	-5.56506500	-5.32490300	-0.96422900
H	-2.23437400	-6.56549000	1.45724000
H	-4.55013600	-6.92958100	0.63731900

B2_pre-TS

C	-1.97437200	2.21197400	-0.44951600
C	-3.04130600	1.32831500	-0.22265300
C	-3.70714600	2.23811900	0.71850200
C	-2.59991300	3.26364200	0.42630500
O	-2.33668600	4.39243300	0.78393100
O	-4.68875100	2.13973800	1.44512700
N	-0.86696600	2.02693900	-1.19591600

N	-3.33415200	0.07711100	-0.62239300
H	-2.72112900	-0.36562600	-1.30819600
H	-0.75535100	1.08378400	-1.55336400
C	0.25939200	2.84735600	-1.36549700
C	1.33282300	2.30954900	-2.09092300
C	0.34365700	4.15217500	-0.86178900
C	2.48235200	3.06252000	-2.30501600
C	1.49853200	4.89614300	-1.09647800
C	2.57203900	4.36504900	-1.81409900
H	1.25590400	1.30235000	-2.49174500
H	-0.47542000	4.57063300	-0.28862100
H	3.30923000	2.62756500	-2.85744300
H	1.55658100	5.90734800	-0.70483600
H	3.46621400	4.95534400	-1.98555500
C	-4.66376000	-0.48493600	-0.43888500
C	-4.57399100	-1.87659100	0.20120000
C	-5.97642800	-2.44430300	0.44489500
C	-6.74885800	-2.53328500	-0.87999400
C	-6.82447800	-1.17067800	-1.57777000
C	-5.42500100	-0.57908900	-1.77074600
H	-5.21218300	0.18504900	0.23009800
H	-4.01631600	-2.53086900	-0.47735400
H	-5.92220200	-3.43462000	0.90495600
H	-6.52264300	-1.79162700	1.13725200
H	-7.75255900	-2.92722400	-0.69006600
H	-6.24644300	-3.25408100	-1.53853400

H	-7.33390500	-1.26370100	-2.54225000
H	-7.42494900	-0.48299000	-0.96733200
H	-5.47537500	0.42386800	-2.20528000
H	-4.84454500	-1.20204100	-2.46474500
N	-3.72189000	-1.82189700	1.44882300
C	-3.53452600	-3.17161600	2.06430100
H	-4.45331500	-3.50481300	2.54542600
H	-3.23504100	-3.86661700	1.27953000
H	-2.72469800	-3.08993100	2.78601500
C	-4.14582900	-0.82432400	2.47270400
H	-3.34765700	-0.76136800	3.21192800
H	-4.29434400	0.15333000	2.01729900
H	-5.07226900	-1.15678500	2.94238100
C	1.42339900	-1.68557200	-1.21987500
H	1.24060900	-0.63195300	-1.39921700
C	0.37710200	-2.55668000	-1.30573300
H	0.41751800	-3.61697900	-1.11373200
N	-0.91066900	-2.07366600	-1.59895300
O	-1.87166100	-2.85990800	-1.57298000
O	-1.04832600	-0.86846100	-1.93552100
C	2.76152100	-2.07401800	-1.05198400
C	3.94396500	-2.31922200	-0.89759200
C	5.30666500	-2.56570400	-0.59230400
C	6.21823200	-1.49154800	-0.54270100
C	5.76387900	-3.87383900	-0.33593500
C	7.55420100	-1.72832200	-0.24206100

H	5.85704900	-0.48619300	-0.72025800
C	7.10322000	-4.09768900	-0.03660500
H	5.05938500	-4.69757900	-0.37424700
C	8.00054800	-3.02798100	0.01099400
H	8.24986700	-0.89614500	-0.20132100
H	7.44936300	-5.10737900	0.16035900
H	9.04504200	-3.20657300	0.24621300
C	-0.35128000	-0.88231800	1.42828400
O	-0.84305400	0.40306200	1.43912700
C	1.33097400	0.59087100	1.52777900
C	1.04352600	-0.79740900	1.50720600
O	-1.14100700	-1.85672900	1.27042800
N	0.23068100	1.32880100	1.51287500
H	1.71543700	-1.63358800	1.58066100
H	-2.71087100	-1.57091500	1.17518300
C	2.65487600	1.23107500	1.50639700
C	2.78293700	2.58069000	1.14856600
C	3.80888100	0.49021500	1.79881900
C	4.03964500	3.17535300	1.09337500
H	1.89456800	3.14637300	0.89416300
C	5.06586200	1.09004200	1.74616600
H	3.72438600	-0.55750600	2.06496500
C	5.18551600	2.43523000	1.39258800
H	4.12097800	4.21691400	0.79905000
H	5.94964400	0.50134600	1.97113500
H	6.16516700	2.90196600	1.34598600

B2_TS

C	-2.10511300	2.20838300	-0.34569600
C	-3.16201600	1.29949900	-0.17552700
C	-3.84751900	2.14756100	0.80601700
C	-2.74747000	3.20093200	0.58422900
O	-2.50819200	4.32067500	0.98564300
O	-4.83078300	1.99092900	1.52200200
N	-0.98230400	2.06538500	-1.07552500
N	-3.42960900	0.07000600	-0.65177300
H	-2.74733400	-0.35750800	-1.29219000
H	-0.83844500	1.12755300	-1.44640400
C	0.09491500	2.94575200	-1.25283700
C	1.15570200	2.49799700	-2.05538200
C	0.14337700	4.22428000	-0.68176900
C	2.25403700	3.31982000	-2.28734000
C	1.24902100	5.03569200	-0.92997600
C	2.30712800	4.59796400	-1.73009100
H	1.10492600	1.50950300	-2.50450900
H	-0.66516800	4.56939700	-0.04652700
H	3.06935500	2.95710400	-2.90544900
H	1.28033800	6.02555300	-0.48457700
H	3.16128400	5.24177700	-1.91343700
C	-4.75035000	-0.52114100	-0.51462500
C	-4.62268700	-1.96608500	-0.01145400
C	-5.99568900	-2.59498100	0.22167600

C	-6.79149300	-2.58867900	-1.09313400
C	-6.91336500	-1.17168500	-1.66498800
C	-5.53524700	-0.52191600	-1.83729100
H	-5.30077400	0.08328100	0.21256800
H	-4.06628300	-2.53060500	-0.76469300
H	-5.90117000	-3.62099400	0.59086900
H	-6.54551900	-2.02275700	0.97865000
H	-7.78207600	-3.02164100	-0.92161700
H	-6.28437000	-3.23530200	-1.82091100
H	-7.44095900	-1.19449100	-2.62397600
H	-7.51991500	-0.55758200	-0.98628900
H	-5.62856600	0.51172200	-2.18337500
H	-4.95222800	-1.06420000	-2.59314700
N	-3.71906500	-2.02070100	1.20410000
C	-3.27258400	-3.41424800	1.50826100
H	-4.13118700	-4.01827700	1.79922400
H	-2.78828300	-3.80328000	0.61258500
H	-2.54603500	-3.36085600	2.31574200
C	-4.23672900	-1.31876600	2.41831000
H	-3.42093700	-1.28837100	3.13952500
H	-4.52912500	-0.29831000	2.16483600
H	-5.08297600	-1.87213800	2.82463200
C	1.32566800	-1.37912500	-0.54759200
H	1.16820000	-0.41093300	-1.01377500
C	0.29957500	-2.33160200	-0.79556300
H	0.35849800	-3.36504800	-0.49424600

N	-0.89718600	-1.94458000	-1.29064500
O	-1.86129800	-2.76291100	-1.35789300
O	-1.05835600	-0.74526000	-1.73300000
C	2.67952900	-1.83034000	-0.55617900
C	3.84102800	-2.18374900	-0.55325400
C	5.21626100	-2.54921900	-0.55146300
C	6.20451500	-1.57019700	-0.77309700
C	5.60818700	-3.88120800	-0.32091500
C	7.55044900	-1.92154000	-0.75936000
H	5.90008400	-0.54435900	-0.94347300
C	6.95704900	-4.22206100	-0.31029200
H	4.84615300	-4.63460700	-0.15271900
C	7.93109900	-3.24536200	-0.52828200
H	8.30546400	-1.16049600	-0.93053200
H	7.25008500	-5.25192700	-0.13182200
H	8.98251200	-3.51511100	-0.51894100
C	-0.25255600	-0.63183700	1.60268100
O	-0.57830700	0.69100600	1.63849100
C	1.61312200	0.65646200	1.47307900
C	1.17960600	-0.73050600	1.39926200
O	-1.11946000	-1.50386400	1.64155200
N	0.60761400	1.48634100	1.57073000
H	1.73032800	-1.55341500	1.83048900
H	-2.83801800	-1.52178900	0.96560000
C	2.98412000	1.17145000	1.36486100
C	3.20193100	2.49911700	0.96443400

C	4.08276200	0.34820200	1.64734400
C	4.49824200	2.98734400	0.84641700
H	2.35252300	3.12870700	0.73131800
C	5.37987600	0.84505600	1.53595500
H	3.92670300	-0.67995900	1.95186200
C	5.59067200	2.16423100	1.13189900
H	4.65313900	4.01035700	0.51895800
H	6.22252100	0.19760800	1.75525400
H	6.60196100	2.54866200	1.03801200

B2_post-TS

C	-1.79316000	1.93762500	-0.96531900
C	-3.02503300	1.28902600	-0.76280300
C	-3.62106400	2.45997000	-0.09978900
C	-2.30472200	3.21326600	-0.36186400
O	-1.87730500	4.33107300	-0.16074700
O	-4.68957700	2.66763600	0.46423000
N	-0.66190700	1.42600200	-1.49049000
N	-3.46473700	0.04616600	-1.01744600
H	-2.78645100	-0.63541700	-1.40975800
H	-0.71733900	0.41951100	-1.69003200
C	0.60424900	2.01308300	-1.65132500
C	1.62060300	1.19657300	-2.17483700
C	0.88504400	3.33873500	-1.29629900
C	2.90894900	1.69815600	-2.33103600
C	2.17797100	3.82737900	-1.47397300

C	3.19614600	3.01965000	-1.98639600
H	1.39629000	0.17194900	-2.45658300
H	0.10710800	3.96978400	-0.87975900
H	3.68513100	1.05028000	-2.72651500
H	2.38864800	4.85692200	-1.20022900
H	4.19945200	3.41388300	-2.11249900
C	-4.84158600	-0.35445800	-0.77799900
C	-4.88078800	-1.50279800	0.24627200
C	-6.31129300	-1.91878600	0.57474000
C	-7.01129300	-2.37911400	-0.71423600
C	-6.97047500	-1.28621100	-1.78987100
C	-5.53441900	-0.82358800	-2.06593200
H	-5.37372200	0.51816700	-0.38668700
H	-4.31822800	-2.33718400	-0.18249200
H	-6.32374200	-2.72413600	1.31690900
H	-6.86217900	-1.07152000	1.00053800
H	-8.04540000	-2.65824800	-0.48874300
H	-6.51261800	-3.28187600	-1.08933200
H	-7.43140200	-1.64905000	-2.71417300
H	-7.56830300	-0.42760900	-1.45599300
H	-5.51898900	-0.00147700	-2.78737700
H	-4.94797900	-1.64379000	-2.49974200
N	-4.06083300	-1.12370800	1.46642700
C	-3.63348400	-2.31800600	2.25795900
H	-4.51267500	-2.79902100	2.68640200
H	-3.09486500	-2.97709000	1.57399700

H	-2.96878400	-1.96978700	3.04685200
C	-4.65776700	-0.05916700	2.33122100
H	-3.90026300	0.22736300	3.05869700
H	-4.91354100	0.81267900	1.72657900
H	-5.53711600	-0.46080100	2.83346200
C	0.85820000	-1.12794700	0.32856500
H	0.59748100	-0.41261100	-0.44797400
C	-0.21191900	-2.16671000	0.34720100
H	-0.27442900	-2.91946900	1.11854400
N	-1.20183100	-2.15437200	-0.52918800
O	-2.20410400	-2.94708400	-0.40865100
O	-1.19841000	-1.33635100	-1.55649000
C	2.18810600	-1.63177600	0.00350500
C	3.30050600	-1.96884100	-0.33693500
C	4.64820900	-2.23548500	-0.71534600
C	5.51287800	-1.15293700	-0.96655200
C	5.13780100	-3.54784000	-0.83051900
C	6.83732900	-1.38497100	-1.32205000
H	5.13165100	-0.14339900	-0.86577700
C	6.46481900	-3.76978100	-1.18860400
H	4.47006200	-4.38081500	-0.63824400
C	7.31750500	-2.69176200	-1.43427900
H	7.49771400	-0.54396100	-1.51023700
H	6.83513600	-4.78653700	-1.27636600
H	8.35167600	-2.86966700	-1.71257800
C	-0.45491900	0.41582400	1.84076800

O	-0.22021300	1.74607700	1.79713300
C	1.80774100	0.86444400	1.73270300
C	0.86633900	-0.30794900	1.69135500
O	-1.57884700	-0.02978400	1.94822500
N	1.19611900	2.00184600	1.77689600
H	1.02232800	-1.01181800	2.51328700
H	-3.18527500	-0.72431500	1.09513900
C	3.27463000	0.83568800	1.75077700
C	3.99675900	1.93136800	1.25309500
C	3.96400600	-0.25679200	2.29526300
C	5.38723000	1.92833000	1.30321700
H	3.45739500	2.75959200	0.81110200
C	5.35483500	-0.25324100	2.34389400
H	3.41624200	-1.11338400	2.67064200
C	6.06908000	0.83877400	1.84960400
H	5.93973500	2.77445400	0.90691900
H	5.88099500	-1.10778800	2.75586700
H	7.15414400	0.83373100	1.87811200

References

(1) (a) Capreti, N.; Jurberg, I. D. Michael Addition of Soft Carbon Nucleophiles to Alkylidene Isoxazol-5-ones: A Divergent Entry to β-Branched Carbonyl Compounds *Org. Lett.* **2015**, *17*, 2490–2493; (b) Hellmuth, T.; Frey, W.; Peters, R. Regioselective Catalytic Asymmetric C-Alkylation of Isoxazolinones by a Base-Free Palladacycle-Catalyzed Direct 1,4-Addition *Angew. Chem. Int. Ed.* **2015**, *54*, 2788–2791.

(2) Belot, S.; Vogt, K. A.; Besnard, C.; Krause, N.; Alexakis, A. Enantioselective One-Pot Organocatalytic Michael Addition/Gold-Catalyzed Tandem Acetalization/Cyclization *Angew. Chem. Int. Ed.* **2009**, *48*, 8923-8926.

(3) Z. A. Ignatiuk, M. J. Janicki, R. W. Góra, K. Konieczny, R. Kowalczyk, "Applications of Thermal Activation, Ball-milling and Aqueous Medium in Stereoselective Michael Addition of Nitromethane to Enynones Catalyzed by Chiral Squaramides" *Adv. Synth. Catal.* **2019**, *361*, 1108-1116.

(4) (a) Zard, S. Z. New Syntheses of Alkynes: A Tale of Serendipity and Design *Chem. Commun.* **2002**, *2*, 1555-1563; (b) Da Silva, A. F.; Fernandes, A. A. G.; Thurow, S.; Stivanin, M. L.; Jurberg, I. D. Isoxazol-5-Ones as Strategic Building Blocks in Organic Synthesis *Synthesis* **2018**, *50*, 2473-2489.

(5) For reviews in hydrogen bond catalysis, see: (a) Pihko, P. M. *Hydrogen Bonding in Organic Synthesis*; Wiley-VCH Verlag GmbH & Co. KGaA: Weinheim, Germany, 2009; b) Schreiner, P. R. Metal-free Organocatalysis through Explicit Hydrogen Bonding Interactions. *Chem. Soc. Rev*. **2003**, *32*, 289-296; (c) Doyle, A. G.; Jacobsen, E. N. Small-Molecule H-Bond Donors in Asymmetric Catalysis. *Chem. Rev.* **2007**, *107*, 5713-5743; (d) McGilvra, J. D.; Gondi, V. D.; Rawal, V. H. In *Enantioselective Organocatalysis*; D alko, P. I., Ed.; Wiley-VCH: Weinheim, 2007; pp 189−254. Papai's model: (e) Hamza, A.; Schubert, G.; Sóos, T.; Pápai, I. Theoretical Studies on the Bifunctionality of Chiral Thiourea-Based Organocatalysts: Competing Routes to C−C Bond Formation. *J. Am. Chem. Soc.* **2006**, *128*, 13151-13160. Takemoto's model: (f) Okino, T.; Hoashi, Y.; Furukawa, T.; Xu, X. N.; Takemoto, Y. Enantio- and Diastereoselective Michael Reaction of 1,3-Dicarbonyl Compounds to Nitroolefins Catalyzed by a Bifunctional Thiourea. *J. Am. Chem. Soc.* **2005**, *127*, 119-125.

(6) a) Becke, A. D. Density-functional Thermochemistry. III. The Role of Exact Exchange. *J. Chem. Phys.* **1993**, *98*, 5648−5652. (b) Grimme, S.; Antony, J.; Ehrlich, S.; Krieg, H. A Consistent and Accurate Ab Initio Parametrization of Density Functional Dispersion Correction (DFT-D) for the 94 Elements H-Pu. *J. Chem. Phys*. 2010, 132, No. 154104. (c) Grimme, S.; Ehrlich, S.; Goerigk, L. Effect of the Damping Function in Dispersion Corrected Density Functional Theory. *J. Comput. Chem.* **2011**, 32, 1456−1465.

(7) Frisch, M. J.; Trucks, G. W.; Schlegel, H. B.; Scuseria, G. E.; Robb, M. A.; Cheeseman, J. R.; Scalmani, G.; Barone, V.; Mennucci, B.; Petersson, G. A.; Nakatsuji, H.; Caricato, M.; Li, X.; Hratchian, H. P.; Izmaylov, A. F.; Bloino, J.; Zheng, G.; Sonnenberg, J. L.; Hada, M.; Ehara, M.; Toyota, K.; Fukuda, R.; Hasegawa, J.; Ishida, M.; Nakajima, T.; Honda, Y.; Kitao, O.; Nakai, H.; Vreven, T.; Montgomery, J. A., Jr.; Peralta, J. E.; Ogliaro, F.; Bearpark, M.; Heyd, J. J.; Brothers, E.; Kudin, K. N.; Staroverov, V. N.; Kobayashi, R.; Normand, J.; Raghavachari, K.; Rendell, A.; Burant, J. C.; Iyengar, S. S.; Tomasi, J.; Cossi, M.; Rega, N.; Millam, J. M.; Klene, M.; Knox, J. E.; Cross, J. B.; Bakken, V.; Adamo, C.; Jaramillo, J.; Gomperts, R.; Stratmann, R. E.; Yazyev, O.; Austin, A. J.; Cammi, R.; Pomelli, C.; Ochterski, J. W.; Martin, R. L.; Morokuma, K.; Zakrzewski, V. G.; Voth, G. A.; Salvador, P.; Dannenberg, J. J.; Dapprich, S.; Daniels, A. D.; Farkas, Ö.; Foresman, J. B.; Ortiz, J. V.; Cioslowski, J.; Fox, D. J., Gaussian, Inc., Wallingford CT, 2001.

(8) Tomasi, J.; Persico, M. Molecular Interactions in Solution: An Overview of Methods Based on Continuous Distributions of the Solvent. *Chem. Rev.* **1994**, *94*, 2027–2094.

(9) CYLview20; Legault, C. Y. Université de Sherbrooke, **2020** (http://www.cylview.org).

(10) Frias, M.; Mas-Ballesté, R.; Arias, S.; Alvarado, C.; Alemán, J. Asymmetric Synthesis of Rauhut–Currier-Type Products by a Regioselective Mukaiyama Reaction under Bifunctional Catalysis. *J. Am. Chem. Soc.* **2017**, *139*, 672–679.

(11) Kaya, U.; Chauhan, P.; Deckers, K.; Puttreddy, R.; Rissanen, K.; Raabe, G.; Enders, D. Asymmetric synthesis of tetrahydrobenzofurans and annulated dihydropyrans via cooperative one-pot organo- and silver-catalysis. *Synthesis* **2016**, *48*, 3207-3216.
